# Supplementary material for: Epigenetic cell memory: The gene’s inner chromatin modification circuit
Source: PLoS Comput Biol. 2022 Apr 6;18(4):e1009961. doi: 10.1371/journal.pcbi.1009961 (PMC8985953; doi:10.1371/journal.pcbi.1009961)
Supplement: S1 File — Fig A: establishment, erasure, and maintenance of DNA methylation. Fig B: competitive interactions between activating histone modifications DA and DNA methylation D1R. Fig C: cooperative interactions between DNA methylation D1R and repressive histone modification D2R. Fig D: competition between activating and repressive histone modifications. Fig E: trajectories of D¯R and D¯A of the full and the reduced system. Figs F-I: effect of system parameters on the stochastic behavior of the histone modification circuit. Fig J: trajectories of D¯12R and D¯A of the full and the reduced system. Figs K-O: effect of system parameters on the deterministic behavior of the chromatin modification circuit. Figs P-W: effect of system parameters on the stochastic behavior of the chromatin modification circuit. Fig X: time trajectories of D¯A. Fig Y: effect of ϵ and μ′ on the silencing and reactivation processes. Table A: reactions and parameter values used to generate the plots in Fig 4A, 4B. Table B: reactions and parameter values used to generate the plots in Fig 4C, 4D. Table C: reactions and parameter values used to generate the plots in Fig 4F. Table D: parameter values relative to Fig D. Table E: parameter values relative to Fig E. Table F: reactions and parameter values used to generate the plots in Fig F. Table G: reactions and parameter values used to generate the plots in Fig G. Table H: reactions and parameter values used to generate the plots in Fig H. Table I: reactions and parameter values used to generate the plots in Fig I. Table J: parameter values relative to Fig J. Table K: reactions and parameter values used to generate the plots in Fig P. Table L: reactions and parameter values used to generate the plots in Fig Q. Table M: reactions and parameter values used to generate the plots in Fig R. Table N: reactions and parameter values used to generate the plots in Fig S. Table O: reactions and parameter values used to generate the plots in Fig T. Table P: reactions and p [file pcbi.1009961.s001.pdf]

Epigenetic cell memory:
The gene's inner chromatin modification circuit

Simone Bruno[1], Ruth J. Williams[2], and Domitilla Del Vecchio[1]

[1] *Department of Mechanical Engineering, Massachusetts Institute of Technology, 77 Massachusetts Avenue, Cambridge, MA 02139. Emails:* (sbruno,ddv)@mit.edu

[2] *Department of Mathematics, University of California, San Diego, 9500 Gilman Drive, La Jolla CA 92093-0112. Email:* rjwilliams@ucsd.edu

Supporting information: S1 File

1 Derivation of the chromatin modification circuit models

1.1 Single histone modification: reactions and model

In order to realize the model, we make the following assumptions:

- we lump together the two methylation states (me2 and me3) for simplicity because both of them are associated with gene repression. This will not affect the type of qualitative predictions that we seek to make in this paper;
- a nucleosome cannot be characterized by more than one modified histone simultaneously;
- D represents an unmodified nucleosome;
- $\mathrm{D^M}$ represents a modified nucleosome (methylated or acetylated);
- For a species X, we use n^X to denote the number of such a species and use italics, X, to denote concentration when appropriate (defining the reaction volume as Ω, $X = \frac{n^X}{\Omega}$). When working with concentrations, $D_{tot} = \frac{\mathrm{D_{tot}}}{\Omega}$ represents the total concentration of nucleosomes that can be modified within a gene of interest.

Then, we model the *establishment*, the *catalysis* and the *erasure* mechanisms as follows.

***De novo* establishment**: the writers of histone modifications are usually found in multi-protein complexes (denoted here by W) that contribute to stabilizing their enzymatic activity and help in recruiting these writers to specific loci on DNA. These writer enzymes can be recruited to DNA by TFs [1](Chapter 6). In particular, based on previous works (Chapter 6 of [1] and [2]), we assume that the TF binds to DNA first and then recruits W. Furthermore, even if it is less effective, the writers can still modify the histone even without being recruited by the TF [3, 4] and then we introduce in the model also the possibility that the enzyme W binds directly to D. By modeling the *de novo* establishment of a histone mark by an enzymatic reaction, the reactions characterizing

this phase are the following:

$$\begin{aligned}
&\mathrm{P}+\mathrm{P}+... \underset{d_P}{\overset{a_P}{\rightleftharpoons}} \mathrm{P}_n, \qquad \mathrm{P}_n+\mathrm{D} \underset{\bar{d}_P}{\overset{\bar{a}_P}{\rightleftharpoons}} \bar{\mathrm{C}}_\mathrm{P}, \\
&\mathrm{D}+\mathrm{W} \underset{d_W}{\overset{a_{W0}}{\rightleftharpoons}} \mathrm{C}_{\mathrm{W0}} \xrightarrow{\kappa_\mathrm{W}} \mathrm{D}^\mathrm{M}+\mathrm{W}, \qquad \bar{\mathrm{C}}_\mathrm{P}+\mathrm{W} \underset{d_W}{\overset{a_{W0}+a_W}{\rightleftharpoons}} \mathrm{C}_\mathrm{W} \xrightarrow{\kappa_\mathrm{W}} \mathrm{D}^\mathrm{M}+\mathrm{P}_n+\mathrm{W},
\end{aligned} \tag{1}$$

in which the first enzymatic reaction represents the basal *de novo* establishment and the second enzymatic reaction represents the recruited *de novo* establishment. Furthermore, P is a sequence-specific TF that can form multimer with n copies (P_n), W denotes the complex containing the specific enzyme that writes the modification, $\bar{\mathrm{C}}_\mathrm{P}$ is the complex between D and P_n, C_{W0} is the complex between D and W, C_W is the complex between $\bar{\mathrm{C}}_\mathrm{P}$ and W, a_P, a_1, a_{W0}, a_W and d_P, d_1, d_W are association and dissociation rate constants, respectively, and κ_W is the catalytic rate constant of the enzymatic reaction.

Auto-catalysis: As well explained in Section "Models", histone modification can be quickly restored on unmodfied histones through a read-write mechanism where a modified histone is recognized by "readers", proteins that bind the modified histone, which recruit writer enzymes for the same modification, thus enabling the modification of nearby unmodified histones [5](Chapter 22), [6, 7, 8, 9, 10]. Consistent with early work that modeled this auto-catalysis mechanism as a recruited modification [11, 12], we model the auto-catalysis phase with the following enzymatic reactions:

$$\mathrm{D}^\mathrm{M}+\mathrm{V} \underset{d_M}{\overset{a_M}{\rightleftharpoons}} \mathrm{M}, \qquad \mathrm{D}+\mathrm{M} \underset{\bar{d}}{\overset{\bar{a}}{\rightleftharpoons}} \mathrm{C}_\mathrm{M} \xrightarrow{\kappa_\mathrm{M}} \mathrm{D}^\mathrm{M}+\mathrm{M}, \qquad \bar{\mathrm{C}}_\mathrm{P}+\mathrm{M} \underset{\bar{d}}{\overset{\bar{a}}{\rightleftharpoons}} \mathrm{C}_{\mathrm{M1}} \xrightarrow{\kappa_\mathrm{M}} \mathrm{D}^\mathrm{M}+\mathrm{P}_n+\mathrm{M} \tag{2}$$

in which V is a multi-protein complex containing the reader and the writer, M denotes the complex between D^M and V, C_M denotes the complex between D and M, C_{M1} denotes the complex between $\bar{\mathrm{C}}_\mathrm{P}$ and M, a_M, $\bar{a}$ and d_M, $\bar{d}$ are the association and dissociation rate constants, respectively, and κ_M is the catalytic rate constant. Furthermore, the complex M, containing V, can bind also to $\bar{\mathrm{C}}_\mathrm{P}$ (D bound to P_n) to introduce in the model the fact that, as the sequence-specific TF does not sequester D from the writer enzyme recruited the *de novo* establishment phase [1](Chapter 6),[3, 4], in the same way the sequence-specific TF does not sequester D from the writer enzyme recruited through the read-write (auto-catalysis) mechanism. In particular, we assume that the association and dissociation rate constants of M with D are independent of weather D is bound to P_n or free. This reaction model assumes that (see Fig 1C) a modified nucleosome can recruit the writer enzyme to any other unmodified nucleosome with equal probability, which increases with the concentration of modified nucleosomes. This is plausible given higher-order chromatin structure, such as by DNA-looping [13], which allows in principle any nucleosome to move close to any other nucleosome.

Active and passive erasure: active erasure of the mark through eraser enzymes can be modeled through a similar enzymatic reaction similar to (1):

$$\mathrm{D}^\mathrm{M}+\mathrm{E} \underset{d_E}{\overset{a_E}{\rightleftharpoons}} \mathrm{C}_\mathrm{E} \xrightarrow{\kappa_\mathrm{E}} \mathrm{D}+\mathrm{E} \tag{3}$$

in which E is a multi-protein complex containing the eraser enzyme, C_E denotes the complex between D^M and E, a_E and d_E are the association and dissociation rate constants, respectively, and κ_E is the catalytic rate constant. In addition to being removed by suitable enzymes, the mark can be passively removed through dilution due to DNA replication during S phase [5](Chapter

22). Therefore, calling δ the rate constant of cell division (and DNA replication), we will have the passive erasure reaction

$$\mathrm{D^M} \xrightarrow{\delta} \mathrm{D}. \tag{4}$$

Derivation of the model: the ODE model associated with reactions (1-4) is given by

$$\begin{aligned}
\dot{P}_n &= a_P P^n - d_P P_n + \kappa_W C_W + \kappa_M C_{M1} - \delta P_n \\
\dot{\bar{C}}_P &= \bar{a}_P P_n D - \bar{d}_P \bar{C}_P - (a_{W0} + a_W)\bar{C}_P W + d_W C_W - \bar{a}\bar{C}_P M + \bar{d} C_{M1} \\
\dot{C}_{W0} &= a_{W0} D W - d_W C_{W0} - \kappa_W C_{W0} \\
\dot{C}_W &= (a_{W0} + a_W)\bar{C}_P W - d_W C_W - \kappa_W C_W \\
\dot{M} &= a_M D^M V - d_M M - \bar{a} D M + \bar{d} C_M + \kappa_M C_M - \bar{a}\bar{C}_P M + \bar{d} C_{M1} + \kappa_M C_{M1} \\
\dot{C}_M &= \bar{a} D M - \bar{d} C_M - \kappa_M C_M \\
\dot{C}_{M1} &= \bar{a}\bar{C}_P M - \bar{d} C_{M1} - \kappa_M C_{M1} \\
\dot{C}_E &= a_E D^M E - d_E C_E - \kappa_E C_E \\
\dot{D}^M &= \kappa_W (C_{W0} + C_W) + \kappa_M (C_M + C_{M1}) - a_E D^M E + d_E C_E - \delta D^M.
\end{aligned} \tag{5}$$

Since the binding reactions are much faster than the other reactions, we set the complexes to their quasi-steady state (QSS) values by setting $\dot{P}_n = \dot{\bar{C}}_P = \dot{C}_{W0} = \dot{C}_W = \dot{M} = \dot{C}_M = \dot{C}_{M1} = \dot{C}_E = 0$, obtaining

$$\begin{aligned}
&P_n = \frac{P^n}{K_P}, \quad \bar{C}_P = \frac{P^n}{K_P \bar{K}_P} D, \quad C_{W0} = \frac{W}{K_{W0}} D, \quad C_W = (\frac{1}{K_W} + \frac{1}{K_{W0}})\frac{P^n W}{K_P \bar{K}_P} D, \quad M = \frac{D^M V}{K_{MM}} \\
&C_M = \frac{V}{K_{MM}\bar{K}_M} D^M D = \frac{V}{K_M} D^M D, \quad C_{M1} = \frac{V}{K_M} D^M \bar{C}_P = \frac{V P^n}{K_M K_P \bar{K}_P} D^M D, \quad C_E = \frac{E}{K_E} D^M,
\end{aligned}$$

in which we introduce the dissociation constants of the first two reactions in (1) and of the first reaction in (2) ($K_P = \frac{d_P}{a_P}$, $\bar{K}_P = \frac{\bar{d}_P}{\bar{a}_P}$ and $K_{MM} = \frac{d_M}{a_M}$ respectively), the Michaelis-Menten (M-M) constants of enzymatic reactions in (1), (2) and (3) ($K_{W0} = \frac{d_W + \kappa_W}{a_{w0}}$, $\bar{K}_M = \frac{\bar{d} + \kappa_M}{\bar{a}}$, $K_W = \frac{d_W + \kappa_W}{a_W}$ and $K_E = \frac{d_E + \kappa_E}{a_E}$, respectively) and we define $K_M = K_{MM}\bar{K}_M$.

Substituting these values in the last ODE of (5), we obtain

$$\begin{aligned}
\dot{D}^M &= \kappa_W C_{W0} + \kappa_W C_W + \kappa_M (C_M + C_{M1}) - \kappa_E C_E - \delta D^M \\
&= \kappa_W \frac{W}{K_{W0}} D + \kappa_W (\frac{1}{K_W} + \frac{1}{K_{W0}}) \frac{P^n W}{K_P \bar{K}_P} D + \kappa_M \left(\frac{V}{K_{MM}\bar{K}_M} D^M D + \frac{V P^n}{K_M K_P \bar{K}_P} D^M D \right) \\
&- \kappa_E \frac{E}{K_E} D^M - \delta D^M \\
&= \left(\kappa_W \frac{W}{K_{W0}} \left(1 + \frac{P^n}{K_P \bar{K}_P} \right) + \kappa_W \frac{W}{K_W} \frac{P^n}{K_P \bar{K}_P} + \kappa_M \left(1 + \frac{P^n}{K_P \bar{K}_P} \right) \frac{V}{K_M} D^M \right) D - (\kappa_E \frac{E}{K_E} + \delta) D^M.
\end{aligned} \tag{6}$$

D can be obtained by the DNA conservation law $D_{tot} = D + D^M + \bar{C}_P + C_{W0} + C_W + M + C_M + C_{M1} + C_E$ that, by considering the complexes dynamics to the QSS, can be written as

$$\begin{aligned}
D_{tot} &= D \left(1 + \frac{P^n}{K_P \bar{K}_P} + \frac{W}{K_{W0}} \left(1 + \frac{P^n}{K_P \bar{K}_P} \right) + \frac{P^n W}{K_P \bar{K}_P K_W} + \frac{V D^M}{K_M} + \frac{V D^M P^n}{K_M K_P \bar{K}_P} \right) \\
&+ D^M \left(1 + \frac{V}{K_{MM}} + \frac{E}{K_E} \right).
\end{aligned}$$

By solving for D the conservation law, we obtain

$$D = \frac{D_{tot} - D^M(1 + \frac{V}{K_{MM}} + \frac{E}{K_E})}{1 + \frac{P^n}{K_P\bar{K}_P} + \frac{W}{K_{W0}}\left(1 + \frac{P^n}{K_P\bar{K}_P}\right) + \frac{P^nW}{K_P\bar{K}_PK_W} + \frac{VD^M}{K_M} + \frac{VD^MP^n}{K_MK_P\bar{K}_P}}.$$

Now, if we assume that the sum of the complexes C_W, C_{W0}, M, C_M, C_{M1} and C_E is negligible with respect to $D + D^M + \bar{C}_P$ (that is, $D\left(\frac{W}{K_{W0}}\left(1 + \frac{P^n}{K_P\bar{K}_P}\right) + \frac{P^nW}{K_P\bar{K}_PK_W} + \frac{VD^M}{K_M} + \frac{VD^MP^n}{K_MK_P\bar{K}_P}\right) + D^M(\frac{E}{K_E} + \frac{V}{K_{MM}}) \ll D\left(1 + \frac{P^n}{K_P\bar{K}_P}\right) + D^M$), D can be written as

$$D \approx \frac{D_{tot} - D^M}{\left(1 + \frac{P^n}{K_P\bar{K}_P}\right)}.$$

Then, equation (6) becomes

$$\begin{aligned}\dot{D}^M &= \left(\kappa_W\frac{W}{K_{W0}} + \kappa_W\frac{W}{K_W}\frac{\frac{P^n}{K_P\bar{K}_P}}{\left(1 + \frac{P^n}{K_P\bar{K}_P}\right)} + \kappa_M\frac{V}{K_M}D^M\right)(D_{tot} - D^M) - (\delta + \kappa_E\frac{E}{K_E})D^M \\ &= \left(k_{W0} + k_W + k_MD^M\right)(D_{tot} - D^M) - (\delta + \bar{k}_E)D^M.\end{aligned}$$

1.2 Activating and repressive histone modifications: reactions and model

In this paper, we will refer to H3K4me3 and H3K4ac as activating histone modifications while we will refer to H3K9me3 as repressive histone modifications. H3K4me3 and H3/H4 acetylation co-exist at promoters and TSS of active genes and H3K4me3 may, in turn, promote downstream acetylation through recruitment of HATs by the Thryotorax complex [8]. Therefore, there is a synergy and mutual reinforcement between H3K4 methylation and histone acetylation. We then denote by D^A a nucleosome carrying activating histone modifications, lumping together H3K4 methylation and H3/H4 acetylation. We instead let D^R denote a nucleosome carrying H3K9me3.

***De novo* establishment**: transcriptional activators, while recruiting the TrX complex for H3K4 methylation and/or HATs for acetylation, compete for binding to DNA with PRC2 recruited there by transcriptional repressors (see [14](Chapter 7) and also [1] (Chapter 6)). This competitive binding scenario is well captured by the two following reactions, in which activators and repressors are assumed not to be able to bind concurrently to DNA to enable a modification.

Activating modifications: H3K acetylation and H3K4me3/1. Based on [5](Chapter 21), sequence-specific transcriptional activators bind DNA and recruit histone acetylases (HATs) such as the SAGA complex to the promoter, which becomes acetylated. Examples of transcriptional activators that recruit HATs include Myc, GATA.1,and Gal4 [14](Chapter 7). The deposition of H3K4me3 then can occur co-transcriptionally as RNAPol II recruits SETs, which methylate H3K4 [15],[5](Chapter 3), or through the recruitment of SETs and MLL1/2 to chromatin by the CxxC binding domain that specifically recognizes unmethylated DNA. Due to this binding domain, H3K4 methylation writers may be hardly recruited to regions with DNA methylation [14](Chapter 7). Finally, MLLs can be recruited to specific promoters by transcriptional activators such as Oct4, which recruits WRD5 to self-renewal associated gene promoters thus facilitating the recruitment of MLLs through the Trythorax complex [16]. We abstract these mechanisms by letting W^A denote the writer enzyme, V^A denote the reader-writer multiprotein complex, that is, TrX for H3K4 methylation and p300/CBP for acetylation, and A denote a sequence-specific transcriptional

activator that can form multimer with n copies (A_n). Therefore, the establishment of *de novo* activating modifications can be modeled by the reactions (1) in which we substitute W with W^A and P with A, obtaining

$$\begin{aligned} &A + A + ... \underset{d_A}{\overset{a_A}{\rightleftharpoons}} A_n, \qquad A_n + D \underset{\bar{d}_A}{\overset{\bar{a}_A}{\rightleftharpoons}} \bar{C}_A, \\ &D + W^A \underset{d_W^A}{\overset{a_{W0}^A}{\rightleftharpoons}} C_{W0}^A \xrightarrow{\kappa_W^A} D^A + W^A, \qquad \bar{C}_A + W^A \underset{d_W^A}{\overset{a_{W0}^A + a_W^A}{\rightleftharpoons}} C_W^A \xrightarrow{\kappa_W^A} D^A + A_n + W^A. \end{aligned} \tag{7}$$

Furthermore, denoting with R a sequence-specific transcriptional repressor that can form multimer with n copies (R_n) and with $\bar{C}^R$ the complex between D and the R_n, we assume that the repressor allows W^A to still bind to the nucleosome at a small rate (a_{W0}^A) and then we introduce the following reactions:

$$\bar{C}_R + W^A \underset{d_W^A}{\overset{a_{W0}^A}{\rightleftharpoons}} C_{W2}^A \xrightarrow{\kappa_W^A} D^A + R_n + W^A. \tag{8}$$

Repressing modifications: H3K9 methylation. H3K9me3 is established by the writer action of Suv39H1, which can be recruited to D by sequence-specific TFs. An example of this is the recruitment of this enzyme to GATA.1 targets by the PU.1 TF, as a means to silence GATA.1 targets and promote the myeloid lineage [17]. In this example, PU.1 binding to GATA.1 targets results in the exclusion of CBP histone acetyltransferase (although not of GATA.1 itself), which is recruited to D by GATA.1. Let thus W^R denote the writer enzyme and V^R denote the reader-writer multiprotein complex. Then, the *de novo* establishment of H3K9me3 can be modeled by the enzymatic reaction (1) with W and P substituted by W^R and R, respectively. The obtained reactions are the following:

$$\begin{aligned} &R + R + ... \underset{d_R}{\overset{a_R}{\rightleftharpoons}} R_n, \qquad R_n + D \underset{\bar{d}_R}{\overset{\bar{a}_R}{\rightleftharpoons}} \bar{C}_R, \\ &D + W^R \underset{d_W^R}{\overset{a_{W0}^R}{\rightleftharpoons}} C_{W0}^R \xrightarrow{\kappa_W^R} D^R + W^R, \qquad \bar{C}_R + W^R \underset{d_W^R}{\overset{a_{W0}^R + a_W^R}{\rightleftharpoons}} C_W^R \xrightarrow{\kappa_W^R} D^R + R_n + W^R. \end{aligned} \tag{9}$$

Furthermore, we assume that the activator allows W^R to still bind to the nucleosome at a small rate (a_{W0}^R) and then we introduce the following reactions:

$$\bar{C}_A + W^R \underset{d_W^R}{\overset{a_{W0}^R}{\rightleftharpoons}} C_{W2}^R \xrightarrow{\kappa_W^R} D^R + A_n + W^R. \tag{10}$$

Other reactions that could potentially occur include W_A adding activating histone marks on nucleosome with repressive histone marks D^R and W^R adding repressive histone marks on nucleosome with activating histone marks D^A. Both of these reactions are excluded in light of the following observations. The first one is excluded because it is known that the Set1/Ash2 HMT that methylates H3K4 does not do so if the neighboring K9 residue is already methylated [18]. The second reaction is also excluded because *in vitro* studies complemented by *in vivo* experiments showed that histones with H3K4me3 tend to lack H3K9me2/3 and that H3K4me3 prevents SUV39H1 and other KMTs for H3K9 from binding H3 [19].

Auto-catalysis: in addition to (2) for both D^A and D^R, we have to introduce the following reactions:

$$\bar{C}_R + M^A \underset{\bar{d}^A}{\overset{\bar{a}^A}{\rightleftharpoons}} C_{M2}^A \xrightarrow{\kappa_M^A} D^A + R_n + M^A, \qquad \bar{C}_A + M^R \underset{\bar{d}^R}{\overset{\bar{a}^R}{\rightleftharpoons}} C_{M2}^R \xrightarrow{\kappa_M^R} D^R + A_n + M^R, \tag{11}$$

in which C^A_{M2} denotes the complex between $\bar{C}_R$ and M^A and C^R_{M2} denotes the complex between $\bar{C}_A$ and M^R. The reason is that, even if the DNA wrapped around a nucleosome is bound by A_n (or R_n), D is still accessible to the repressive (or activating) modification auto-catalytic process. In this way, as we did for the single histone modification model, we introduce in the model the fact that any sequence-specific TF does not sequester D from the auto-catalysis.

Then, the reactions that we have to introduce in order to model the activating histone modification auto-catalysis are reactions (2) and the first enzymatic reaction in (11):

$$D^A + V^A \underset{d^A_M}{\overset{a^A_M}{\rightleftharpoons}} M^A, \qquad D + M^A \underset{\bar{d}^A}{\overset{\bar{a}^A}{\rightleftharpoons}} C^A_M \xrightarrow{\kappa^A_M} D^A + M^A, \tag{12}$$

$$\bar{C}_A + M^A \underset{\bar{d}^A}{\overset{\bar{a}^A}{\rightleftharpoons}} C^A_{M1} \xrightarrow{\kappa^A_M} D^A + A_n + M^A, \qquad \bar{C}_R + M^A \underset{\bar{d}^A}{\overset{\bar{a}^A}{\rightleftharpoons}} C^A_{M2} \xrightarrow{\kappa^A_M} D^A + R_n + M^A, \tag{13}$$

and the reactions that we have to introduce in order to model the repressive histone modification auto-catalysis are reactions (2) and the second enzymatic reaction in (11):

$$D^R + V^R \underset{d^R_M}{\overset{a^R_M}{\rightleftharpoons}} M^R, \qquad D + M^R \underset{\bar{d}^R}{\overset{\bar{a}^R}{\rightleftharpoons}} C^R_M \xrightarrow{\kappa^R_M} D^R + M^R, \tag{14}$$

$$\bar{C}_R + M^R \underset{\bar{d}^R}{\overset{\bar{a}^R}{\rightleftharpoons}} C^R_{M1} \xrightarrow{\kappa^R_M} D^R + R_n + M^R, \qquad \bar{C}_A + M^R \underset{\bar{d}^R}{\overset{\bar{a}^R}{\rightleftharpoons}} C^R_{M2} \xrightarrow{\kappa^R_M} D^R + A_n + M^R, \tag{15}$$

in which we assume that the association and dissociation rate constants of M with D are independent of weather D is bound to A_n (or R_n) or free.

Active and passive erasure: just like a histone mark can recruit, through a reader, protein writers for the same mark to nearby histones, an activating (repressing) histone mark can recruit erasers for a repressing (activating) histone mark [20]. Specifically, JMJD2A is an erasers for H3K9me3/2 and de-methylates H3K9me3/2 through its Jumonji domain while being able to bind H3K4me3 through the Tudor domain. Therefore, H3K4me3 helps recruit this eraser so that it can demethylate neighboring H3K9me3 marks. In turn, JARID is an erasers of H3K4me3 and does so through one of its PHD domains. Through a different PHD domain, it binds H3K9me3. Therefore, H3K9me3 helps recruit this eraser so that it can demethylate neighboring H3K4me3 marks. Furthermore, the CHD4 subunit of the NuRD (nucleosome remodeling and de-acetylase) complex contains a domain (a PHD domain) that recognizes H3K9me3 and prefers unmethylated H3K4. This can give a mechanism through which H3K9me3 recruits HDACs that de-acetylated nearby histones.

Letting D^M and $D^{\bar{M}}$ represent a nucleosome characterized by a histone modification and a nucleosome characterized by the opposing histone modification, such as H3K9me3 and H3K4me3, within a gene of interest, we can therefore model the effective erasure as two enzymatic reactions as follows:

$$D^M + E \underset{d_E}{\overset{a_E}{\rightleftharpoons}} C_E \xrightarrow{\kappa_E} D + E, \tag{16}$$

$$D^{\bar{M}} + E \underset{d_e}{\overset{a_e}{\rightleftharpoons}} E_{act}, \qquad D^M + E_{act} \underset{d_E}{\overset{a_E}{\rightleftharpoons}} C_{E_{act}} \xrightarrow{\kappa_E} D + E_{act} \tag{17}$$

in which E is a multi-protein complex containing the eraser enzyme, C_E denotes the complex between D^M and E, E_{act} denotes the complex between $D^{\bar{M}}$ and E, $C_{E_{act}}$ denotes the complex between D^M and E_{act}, a_E, a_e and d_E, d_e are the association and dissociation rate constants, respectively, and κ_E is the catalytic rate constant. In particular, reaction (16) captures the basal erasure, while

reactions (17) capture the active erasure of $D^{\bar{M}}$ on D^M.

Additional chemical reactions: there is the possibility that the sequence-specific transcriptional activator multimer A_n binds to the actively modified nucleosome D^A and this will lead to more efficient recruitment of the basal transcription machinery [1](Chapter 4). Furthermore, R_n can bind to D^A and block the formation of the pre-initiation complex, independent of its ability to recruit repressive histone modifiers, that is R_n can inhibit transcription even without the need to recruit epigenetic modifiers. In light of this, we can write the additional set of chemical reactions that can occur, noting that if some of them are not present we can simply set the corresponding rate constants to zero:

$$A_n + D^A \underset{\bar{d}'_A}{\overset{\bar{a}'_A}{\rightleftharpoons}} \bar{C}^A_A,\ R_n + D^A \underset{\bar{d}'_R}{\overset{\bar{a}'_R}{\rightleftharpoons}} \bar{C}^A_R, \tag{18}$$

in which $\bar{C}^A_A$ is the complex between D^A and A_n, $\bar{C}^A_R$ is the complex between D^A and R_n and $\bar{a}'_A$, $\bar{a}'_R$ and $\bar{d}'_A$, $\bar{d}'_R$ are association and dissociation rate constants, respectively. Note that in this set of reactions, we have not allowed for both A_n and R_n to bind with closed chromatin D^R [21]. As for the D^A, also the marks contained in the complexes $\bar{C}^A_A$ and $\bar{C}^A_R$ can be actively removed (through erasers recruited by the repressive histone mark) and passively removed (through dilution due to DNA replication during S phase [5](Chapter 22). Then,

$$\bar{C}^A_A + E^A \underset{d^A_E}{\overset{a^A_E}{\rightleftharpoons}} \bar{C}^A_{AE} \xrightarrow{\kappa^A_E} D + A_n + E^A, \quad D^R + E^A \underset{d^A_e}{\overset{a^A_e}{\rightleftharpoons}} E^A_{act}, \quad \bar{C}^A_A + E^A_{act} \underset{d^A_E}{\overset{a^A_E}{\rightleftharpoons}} \bar{C}^A_{AE_{act}} \xrightarrow{\kappa^A_E} D + A_n + E^A_{act}, \tag{19}$$

$$\bar{C}^A_R + E^A \underset{d^A_E}{\overset{a^A_E}{\rightleftharpoons}} \bar{C}^A_{RE} \xrightarrow{\kappa^A_E} D + A_n + E^A, \qquad \bar{C}^A_R + E^A_{act} \underset{d^A_E}{\overset{a^A_E}{\rightleftharpoons}} \bar{C}^A_{RE_{act}} \xrightarrow{\kappa^A_E} D + R_n + E^A_{act}, \tag{20}$$

$$\bar{C}^A_A \xrightarrow{\delta} D + A_n,\ \bar{C}^A_R \xrightarrow{\delta} D + R_n, \tag{21}$$

in which $\bar{C}^A_{AE}$ is the complex between $\bar{C}^A_A$ and E^A, $\bar{C}^A_{AE_{act}}$ is the complex between $\bar{C}^A_A$ and E^A_{act}, $\bar{C}^A_{RE}$ is the complex between $\bar{C}^A_R$ and E^A, $\bar{C}^A_{RE_{act}}$ is the complex between $\bar{C}^A_R$ and E^A_{act} and a_E, a_e, d_E, d_e, κ_E are defined as it was done in (16). It is important to point out that for simplicity we consider that the binding of R_n or A_n to D^A does not affect the erasure rate of the mark and this will not affect the qualitative results we will obtain (if the binding of R_n or A_n to D^A would affect the erasure rate of the mark, we could consider as erasure rate of D^A_{tot} an average of the different erasure rates of D^A).

Derivation of the model: the species involved are the following: D (unmodified nucleosome), D^R (nucleosome with a repressive histone modification, H3K9me3), and D^A (nucleosome with a activating histone modification, H3K4me3 or H3Kac). In terms of notation, for a species X, we use n^X to denote the number of such a species and use italics, X, to denote concentration (defining the reaction volume as Ω, $X = \frac{n^X}{\Omega}$). Furthermore, let us distinguish the parameters, the complexes and the enzymes related to the activating and repressive histone marks with the subscripts "A" and "R" respectively. By introducing ${D^A}_{tot} = D^A + \bar{C}^A_A + \bar{C}^A_R$ = "positively modified nucleosome free or bound by A_n or R_n", $C^A_{Etot} = C^A_E + \bar{C}^A_{AE} + \bar{C}^A_{RE}$ = "positively modified nucleosome, free or bound by A_n or R_n, bound by the erasure enzyme E^A" and $C^A_{E_{act}tot} = C^A_{E_{act}} + \bar{C}^A_{AE_{act}} + \bar{C}^A_{RE_{act}}$ = "positively modified nucleosome, free or bound by A_n or R_n, bound by the complex E^A_{act}", the

ODE model associated with reactions (7,8,9,10,12-21) is given by

$$
\begin{aligned}
\dot{A_n} &= a_A A^n - d_A A_n - \delta A_n - \bar{a}_A A_n D + \bar{d}_A \bar{C}_A + \kappa_W^A C_W^A + \kappa_M^A C_{M1}^A + \kappa_M^R C_{M2}^R \\
&\quad - \bar{a}_A^{'} A_n D^A + \bar{d}_A^{'} \bar{C}_A^A + \kappa_E^A \bar{C}_{AE}^A + \kappa_E^A \bar{C}_{AE_{act}}^A + \delta \bar{C}_A^A \\
\dot{\bar{C}}_A &= \bar{a}_A A_n D - \bar{d}_A \bar{C}_A - (a_{W0}^A + a_W^A)\bar{C}_A W^A + d_W^A C_W^A - a_{W0}^R \bar{C}_A W^R + d_W^R C_W^{R,2} \\
&\quad - \bar{a}^A \bar{C}_A M^A + \bar{d}^A C_{M1}^A - \bar{a}^R \bar{C}_A M^R + \bar{d}^R C_{M2}^R \\
\dot{C}_{W0}^A &= a_{W0}^A D W^A - d_W^A C_{W0}^A - \kappa_W^A C_{W0}^A \\
\dot{C}_W^A &= (a_{W0}^A + a_W^A)\bar{C}_A W^A - d_W^A C_W^A - \kappa_W^A C_W^A \\
\dot{C}_W^{A,2} &= a_{W0}^A \bar{C}_R W^A - d_W^A C_W^{A,2} - \kappa_W^A C_W^{A,2} \\
\dot{M}^A &= a_M^A D_{tot}^A V^A - d_M^A M^A - \bar{a}^A D M^A + \bar{d}^A C_M^A + \kappa_M^A C_M^A - \bar{a}^A \bar{C}_A M^A + \bar{d}^A C_{M1}^A + \kappa_M^A C_{M1}^A \\
&\quad - \bar{a}^A \bar{C}_R M^A + \bar{d}^A C_{M2}^A + \kappa_M^A C_{M2}^A \\
\dot{C}_M^A &= \bar{a}^A D M^A - \bar{d}^A C_M^A - \kappa_M^A C_M^A \\
\dot{C}_{M1}^A &= \bar{a}^A \bar{C}_A M^A - \bar{d}^A C_{M1}^A - \kappa_M^A C_{M1}^A \\
\dot{C}_{M2}^A &= \bar{a}^A \bar{C}_R M^A - \bar{d}^A C_{M2}^A - \kappa_M^A C_{M2}^A \\
\dot{E}_{act}^A &= a_e^A D^R E^A - d_e^A E_{act}^A - a_E^A D_{tot}^A E_{act}^A + d_E^A C_{E_{act}tot}^A + \kappa_E^A C_{E_{act}tot}^A \\
\dot{\bar{C}}_{AE}^A &= a_E^A \bar{C}_A^A E^A - d_E^A \bar{C}_{AE}^A - \kappa_E^A \bar{C}_{AE}^A \\
\dot{\bar{C}}_{RE}^A &= a_E^A \bar{C}_R^A E^A - d_E^A \bar{C}_{RE}^A - \kappa_E^A \bar{C}_{RE}^A \\
\dot{C}_{Etot}^A &= a_E^A D_{tot}^A E^A - d_E^A C_{Etot}^A - \kappa_E^A C_{Etot}^A \\
\dot{\bar{C}}_{AE_{act}}^A &= a_E^A \bar{C}_A^A E_{act}^A - d_E^A \bar{C}_{AE_{act}}^A - \kappa_E^A \bar{C}_{AE_{act}}^A \\
\dot{\bar{C}}_{RE_{act}}^A &= a_E^A \bar{C}_R^A E_{act}^A - d_E^A \bar{C}_{RE_{act}}^A - \kappa_E^A \bar{C}_{RE_{act}}^A \\
\dot{C}_{E_{act}tot}^A &= a_E^A D_{tot}^A E_{act}^A - d_E^A C_{E_{act}tot}^A - \kappa_E^A C_{E_{act}tot}^A \\
\dot{\bar{C}}_A^A &= \bar{a}_A^{'} A_n D^A - \bar{d}_A^{'} \bar{C}_A^A - a_E^A \bar{C}_A^A E^A + d_E^A \bar{C}_{AE}^A - a_E^A \bar{C}_A^A E_{act}^A + d_E^A \bar{C}_{AE_{act}}^A - \delta \bar{C}_A^A \\
\dot{\bar{C}}_R^A &= \bar{a}_R^{'} R_n D^A - \bar{d}_R^{'} \bar{C}_R^A - a_E^A \bar{C}_R^A E^A + d_E^A \bar{C}_{RE}^A - a_E^A \bar{C}_R^A E_{act}^A + d_E^A \bar{C}_{RE_{act}}^A - \delta \bar{C}_A^R \\
\dot{R}_n &= a_R R^n - d_R R_n - \delta R_n - \bar{a}_R R_n D + \bar{d}_R \bar{C}_R + \kappa_W^R C_W^R + \kappa_W^R C_W^{R,2} + \kappa_M^R C_{M1}^R + \kappa_M^A C_{M2}^A \\
&\quad - \bar{a}_R^{'} R_n D^A + \bar{d}_R^{'} \bar{C}_R^A + \kappa_E^A \bar{C}_{RE}^A + \kappa_E^A \bar{C}_{RE_{act}}^A + \delta \bar{C}_R^A \\
\dot{\bar{C}}_R &= \bar{a}_R R_n D - \bar{d}_R \bar{C}_R - (a_{W0}^R + a_W^R)\bar{C}_R W^R + d_W^R C_W^R - a_{W0}^A \bar{C}_R W^A + d_W^A C_W^{A,2} \\
&\quad - \bar{a}^R \bar{C}_R M^R + \bar{d}^R C_{M1}^R - \bar{a}^A \bar{C}_R M^A + \bar{d}^A C_{M2}^A \\
\dot{C}_{W0}^R &= a_{W0}^R D W^R - d_W^R C_{W0}^R - \kappa_W^R C_{W0}^R \\
\dot{C}_W^R &= (a_{W0}^R + a_W^R)\bar{C}_R W^R - d_W^R C_W^R - \kappa_W^R C_W^R \\
\dot{C}_W^{R,2} &= a_{W0}^R \bar{C}_A W^R - d_W^R C_W^{R,2} - \kappa_W^R C_W^{R,2} \\
\dot{M}^R &= a_M^R D^R V^R - d_M^R M^R - \bar{a}^R D M^R + \bar{d}^R C_M^R + \kappa_M^R C_M^R - \bar{a}^R \bar{C}_R M^R + \bar{d}^R C_{M1}^R + \kappa_M^R C_{M1}^R \\
&\quad - \bar{a}^R \bar{C}_A M^R + \bar{d}^R C_{M2}^R + \kappa_M^R C_{M2}^R \\
\dot{C}_M^R &= \bar{a}^R D M^R - \bar{d}^R C_M^R - \kappa_M^R C_M^R \\
\dot{C}_{M1}^R &= \bar{a}^R \bar{C}_R M^R - \bar{d}^R C_{M1}^R - \kappa_M^R C_{M1}^R \\
\dot{C}_{M2}^R &= \bar{a}^R \bar{C}_A M^R - \bar{d}^R C_{M2}^R - \kappa_M^R C_{M2}^R \\
\dot{E}_{act}^R &= a_e^R D_{tot}^A E^R - d_e^R E_{act}^A - a_E^R D^R E_{act}^R + d_E^R C_E^R + \kappa_E^R C_E^R \\
\dot{C}_E^R &= a_E^R D^R E^R - d_E^R C_E^R - \kappa_E^R C_E^R \\
\dot{C}_{E_{act}}^R &= a_E^R D^R E_{act}^R - d_E^R C_E^R - \kappa_E^R C_E^R
\end{aligned}
\qquad (22)
$$

$$\begin{aligned}\dot{D}^A_{tot} &= \kappa^A_W(C^A_{W0} + C^{A,2}_W) + \kappa^A_W C^A_W + \kappa^A_M(C^A_M + C^A_{M1} + C^A_{M2}) - a^A_E D^A_{tot}(E^A + E^A_{act}) \\ &\quad + d^A_E(C^A_{Etot} + C^A_{E_{act}tot}) - \delta D^A_{tot} \\ \dot{D}^R &= \kappa^R_W(C^R_{W0} + C^{R,2}_W) + \kappa^R_W C^R_W + \kappa^R_M(C^R_M + C^R_{M1} + C^R_{M2} - a^R_E D^R(E^R + E^R_{act}) \\ &\quad + d^R_E(C^R_E + C^R_{E_{act}}) - \delta D^R.\end{aligned}$$

Since the binding reactions are much faster than the other reactions, we set the complexes to their QSS values by setting $\dot{A}_n = \dot{\bar{C}}_A = \dot{C}^A_{W0} = \dot{C}^A_W = \dot{C}^{A,2}_W = \dot{M}^A = \dot{C}^A_M = \dot{C}^A_{M1} = \dot{C}^A_{M2} = \dot{E}^A_{act} = \dot{C}^A_{Etot} = \dot{C}^A_{E_{act}tot} = \dot{R}_n = \dot{\bar{C}}_R = \dot{C}^R_{W0} = \dot{C}^R_W = \dot{C}^{R,2}_W \dot{M}^R = \dot{C}^R_M = \dot{C}^R_{M1} = \dot{C}^R_{M2} = \dot{E}^R_{act} = \dot{C}^R_E = \dot{C}^R_{E_{act}} = 0$, obtaining

$$\begin{aligned}
&A_n = \frac{A^n}{K_A}, \quad \bar{C}_A = \frac{A^n}{K_A\bar{K}_A}D, \quad C^A_{W0} = \frac{W^A}{K^A_{W0}}D, \quad C^A_W = (\frac{1}{K^A_{W0}} + \frac{1}{K^A_W})\frac{A^nW^A}{K_A\bar{K}_A}D, \quad M^A = \frac{D^A_{tot}V^A}{K^A_{MM}}, \\
&C^A_M = \frac{V^AD^A_{tot}D}{K^A_{MM}\bar{K}^A_M} = \frac{V^AD^A_{tot}}{K^A_M}D, \quad C^A_{M1} = \frac{V^AD^A_{tot}}{K^A_M}\bar{C}_A = \frac{V^AA^nD^A_{tot}}{K^A_MK_A\bar{K}_A}D, \quad C^A_{M2} = \frac{V^AR^nD^A_{tot}}{K^A_MK_R\bar{K}_R}D, \\
&E^A_{act} = \frac{D^RE^A}{K^A_{EE}}, \quad C^A_{Etot} = \frac{E^A}{K^A_E}D^A_{tot}, \quad C^A_{E_{act}tot} = \frac{E^AD^R}{K^A_{EE}K^A_E}D^A_{tot} = \frac{E^AD^R}{K^{A*}_E}D^A_{tot}, \\
&R_n = \frac{R^n}{K_R}, \quad \bar{C}_R = \frac{R^n}{K_R\bar{K}_R}D, \quad C^R_{W0} = \frac{W^R}{K^R_{W0}}D, \quad C^R_W = (\frac{1}{K^R_{W0}} + \frac{1}{K^R_W})\frac{R^nW^R}{K_R\bar{K}_R}D, \quad M^R = \frac{D^RV^R}{K^R_{MM}}, \\
&C^R_M = \frac{V^RD^RD}{K^R_{MM}\bar{K}^R_M} = \frac{V^RD^R}{K^R_M}D, \quad C^R_{M1} = \frac{V^RD^R}{K^R_M}\bar{C}_R = \frac{V^RR^nD^R}{K^R_MK_R\bar{K}_R}D, \quad C^R_{M2} = \frac{V^RA^nD^R}{K^R_MK_A\bar{K}_A}D, \\
&E^R_{act} = \frac{D^A_{tot}E^R}{K^R_{EE}}, \quad C^R_E = \frac{E^R}{K^R_E}D^R, \quad C^R_{E_{act}} = \frac{E^RD^A_{tot}}{K^R_{EE}K^R_E}D^R = \frac{E^RD^A_{tot}}{K^{R*}_E}D^R, \\
&C^A_{W2} = \frac{R^nW^A}{K_R\bar{K}_RK^A_W}D, \quad C^R_{W2} = \frac{A^nW^R}{K_A\bar{K}_AK^R_W}D,
\end{aligned} \tag{23}$$

in which we introduce the dissociation constants of the first two reactions in (7), of the first reaction in (12), of the first two reactions in (9), of the first reaction in (14) and of the first reaction in (17) ($K^A = \frac{d^A}{a^A}$, $\bar{K}^A = \frac{\bar{d}^A}{\bar{a}^A}$, $K^A_{MM} = \frac{d^A_M}{a^A_M}$, $K^R = \frac{d^R}{a^R}$, $\bar{K}^R = \frac{\bar{d}^R}{\bar{a}^R}$, $K^R_{MM} = \frac{d^R_M}{a^R_M}$, $K^A_{EE} = \frac{d^A_e}{d^A_e}$ and $K^R_{EE} = \frac{d^R_e}{d^R_e}$, respectively), the M-M constant of the enzymatic reactions in (7), (12), (9), (14) and in (17) ($K^A_{W0} = \frac{d^A_W+\kappa^A_W}{a^A_{W0}}$, $\bar{K}^A_M = \frac{\bar{d}^A+\kappa^A_M}{\bar{a}^A}$, $K^R_{W0} = \frac{d^R_W+\kappa^R_W}{a^R_{W0}}$, $\bar{K}^R_M = \frac{\bar{d}^R+\kappa^R_M}{\bar{a}^R}$, $K^A_E = \frac{d^A_E+\kappa^A_E}{a^A_E}$ and $K^R_E = \frac{d^R_E+\kappa^R_E}{a^R_E}$) and the constants $K^A_W = \frac{d^A_W+\kappa^A_W}{a^A_W}$ and $K^R_W = \frac{d^R_W+\kappa^R_W}{a^R_W}$. Substituting these values in the last two ODEs of (22), we obtain

$$\begin{aligned}
\dot{D}^A_{tot} &= \kappa^A_{W0}(C^A_{W0} + C^{A,2}_W) + \kappa^A_W C^A_W + \kappa^A_M(C^A_M + C^A_{M1} + C^A_{M2}) - \kappa^A_E(C^A_{Etot} + C^A_{E_{act}tot}) - \delta D^A_{tot} \\
&= \kappa^A_W\frac{W^A}{K^A_{W0}}D + \kappa^A_W\frac{R^nW^A}{K_R\bar{K}_RK^A_{W0}}D + \kappa^A_W(\frac{1}{K^A_{W0}} + \frac{1}{K^A_W})\frac{A^nW^A}{K_A\bar{K}_A}D \\
&\quad + \kappa^A_M\left(\frac{V^A}{K^A_M}D^A_{tot}D + \frac{V^AA^n}{K^A_MK_A\bar{K}_A}D^A_{tot}D + \frac{V^AR^n}{K^A_MK_R\bar{K}_R}D^A_{tot}D\right) \\
&\quad - \kappa^A_E\frac{E^A}{K^A_E}D^A_{tot} - \kappa^A_E\frac{E^A}{K^{A*}_E}D^RD^A_{tot} - \delta D^A_{tot} \\
&= \left(\kappa^A_W\frac{W^A}{K^A_{W0}}\left(1 + \frac{A^n}{K_A\bar{K}_A} + \frac{R^n}{K_R\bar{K}_R}\right) + \kappa^A_W\frac{W^A}{K^A_W}\frac{A^n}{K_A\bar{K}_A} + \kappa^A_M\left(1 + \frac{A^n}{K_A\bar{K}_A} + \frac{R^n}{K_R\bar{K}_R}\right)\frac{V^A}{K^A_M}D^A_{tot}\right)D
\end{aligned}$$

$$
\begin{aligned}
&- \left(\kappa_E^A \frac{E^A}{K_E^A} + \kappa_E^A \frac{E^A}{K_E^{A*}} D^R + \delta\right) D_{tot}^A \\
\dot{D}^R &= \kappa_W^R (C_{W0}^R + C_W^{R,2}) + \kappa_W^R C_W^R + \kappa_M^R (C_M^R + C_{M1}^R + C_{M2}^R) - \kappa_E^R (C_E^R + C_{E_{act}}^R) - \delta D^R \\
&= \kappa_W^R \frac{W^R}{K_{W0}^R} D + \kappa_W^R \frac{A^n W^R}{K_A \bar{K}_A K_{W0}^R} D + \kappa_W^R \left(\frac{1}{K_{W0}^R} + \frac{1}{K_W^R}\right) \frac{R^n W^R}{K_R \bar{K}_R} D \\
&+ \kappa_M^R \left(\frac{V^R}{K_M^R} D^R D + \frac{V^R R^n}{K_M^R K_R \bar{K}_R} D^R D + \frac{V^R A^n}{K_M^R K_A \bar{K}_A} D^R D\right) \\
&- \kappa_E^R \frac{E^R}{K_E^R} D^R - \kappa_E^R \frac{E^R}{K_E^{R*}} D_{tot}^A D^R - \delta D^R \\
&= \left(\kappa_W^R \frac{W^R}{K_{W0}^R}\left(1 + \frac{A^n}{K_A \bar{K}_A} + \frac{R^n}{K_R \bar{K}_R}\right) + \kappa_W^R \frac{W^R}{K_W^R} \frac{R^n}{K_R \bar{K}_R} + \kappa_M^R \left(1 + \frac{R^n}{K_R \bar{K}_R} + \frac{A^n}{K_A \bar{K}_A}\right) \frac{V^R}{K_M^R} D^R\right) D \\
&- \left(\kappa_E^R \frac{E^R}{K_E^R} + \kappa_E^R \frac{E^R}{K_E^{R*}} D_{tot}^A + \delta\right) D^R.
\end{aligned} \tag{24}
$$

D can be obtained by the DNA conservation law

$$
\begin{aligned}
D_{tot} = D &+ D_{tot}^A + \bar{C}_A + C_{W0}^A + C_W^{A,2} + C_W^A + M^A + C_M^A + C_{M1}^A + C_{M2}^A + C_{Etot}^A + C_{E_{act}tot}^A + E_{act}^A \\
&+ D^R + \bar{C}_R + C_{W0}^R + C_W^{R,2} + C_W^R + M^R + C_M^R + C_{M1}^R + C_{M2}^R + C_E^R + C_{E_{act}}^R + E_{act}^R.
\end{aligned} \tag{25}
$$

In particular, if we assume that the sum of the complexes C_{W0}^A, $C_W^{A,2}$, C_W^A, M^A, C_M^A, C_{M1}^A, C_{M2}^A, C_{W0}^R, $C_W^{R,2}$, C_W^R, M^R, C_M^R, C_{M1}^R, C_{M2}^R, E_{act}^A, C_{Etot}^A, $C_{E_{act}tot}^A$, E_{act}^R, C_E^R, $C_{E_{act}}^R$ is negligible with respect to $D + D_{tot}^A + D^R + \bar{C}_A + \bar{C}_R$ (that is $C_{W0}^A + C_W^{A,2} + C_W^A + M^A + C_M^A + C_{M1}^A + C_{M2}^A + C_{W0}^R + C_W^{R,2} + C_W^R + C_M^R + C_{M1}^R + C_{M2}^R + E_{act}^A + C_{Etot}^A + C_{E_{act}tot}^A + E_{act}^R + C_E^R + C_{E_{act}}^R \ll D + D_{tot}^A + D^R + \bar{C}_A + \bar{C}_R$), (25) can be approximated as

$$
D_{tot} \approx D + D_{tot}^A + \bar{C}_A + D^R + \bar{C}_R. \tag{26}
$$

Then, by considering the complexes dynamics to the QSS, D can be written as

$$
D \approx \frac{D_{tot} - D_{tot}^A - D^R}{1 + \frac{A^n}{K_A \bar{K}_A} + \frac{R^n}{K_R \bar{K}_R}}. \tag{27}
$$

By substituting (27) in equations (24), the ODE model becomes

$$
\begin{aligned}
\dot{D}^A &= \left(\kappa_W^A \frac{W^A}{K_{W0}^A} + \kappa_W^A \frac{W^A}{K_W^A} \frac{\frac{A^n}{K_A \bar{K}_A}}{(1 + \frac{A^n}{K_A \bar{K}_A} + \frac{R^n}{K_R \bar{K}_R})} + \kappa_M^A \frac{V^A}{K_M^A} D^A\right) (D_{tot} - D^A - D^R) \\
&- \left(\delta + \kappa_E^A \frac{E^A}{K_E^A} + \kappa_E^A \frac{E^A}{K_E^{A*}} D^R\right) D^A \\
&= \left(k_{W0}^A + k_W^A + k_M^A D^A\right) (D_{tot} - D^A - D^R) - (\delta + \bar{k}_E^A + k_E^A D^R) D^A \\
\dot{D}^R &= \left(\kappa_W^R \frac{W^R}{K_{W0}^R} + \kappa_W^R \frac{W^R}{K_W^R} \frac{\frac{R^n}{K_R \bar{K}_R}}{(1 + \frac{A^n}{K_A \bar{K}_A} + \frac{R^n}{K_R \bar{K}_R})} + \kappa_M^R \frac{V^R}{K_M^R} D^R\right) (D_{tot} - D^A - D^R) \\
&- \left(\delta + \kappa_E^R \frac{E^R}{K_E^R} + \kappa_E^R \frac{E^R}{K_E^{R*}} D_{tot}^A\right) D^R \\
&= \left(k_{W0}^R + k_W^R + k_M^R D^R\right) (D_{tot} - D^A - D^R) - (\delta + \bar{k}_E^R + k_E^R D^A) D^R
\end{aligned} \tag{28}
$$

in which, with abuse of notation, we indicate D^A_{tot} with D^A.

Now, let us define $\bar{D}^A = D^A/D_{tot} = n^A/\mathrm{D_{tot}}$, $\bar{D}^R = D^R/D_{tot} = n^R/\mathrm{D_{tot}}$ and $\bar{D} = D/D_{tot} = n^D/\mathrm{D_{tot}}$, the normalized time $\tau = tk^A_M D_{tot}$, the normalized inputs $\bar{u}^A = u^A_0 + u^A$ with $u^A_0 = k^A_{W0}/(k^A_M D_{tot})$, $u^A = k^A_W/(k^A_M D_{tot})$, $\bar{u}^R = u^R_0 + u^R$ with $u^R_0 = k^R_{W0}/(k^A_M D_{tot})$ and $u^R = k^R_W/(k^A_M D_{tot})$ and the non-dimensional parameters $\epsilon = (\delta + \bar{k}^A_E)/(k^A_M D_{tot})$, $\mu = k^R_E/k^A_E$, with a constant b such that $(\delta + \bar{k}^R_E)/(\delta + \bar{k}^A_E) = b\mu$, $\alpha = k^R_M/k^A_M$ and $\epsilon' = k^A_E/k^A_M$. With these definitions and letting $\dot{x} := dx/d\tau$, we can rewrite the system model in terms of non-dimensional variables and non-dimensional parameters as follows:

$$\begin{aligned}\dot{\bar{D}}^A &= (\bar{u}^A + \bar{D}^A)(1 - \bar{D}^A - \bar{D}^R) - (\epsilon + \epsilon' \bar{D}^R)\bar{D}^A \\ \dot{\bar{D}}^R &= (\bar{u}^R + \alpha\bar{D}^R)(1 - \bar{D}^A - \bar{D}^R) - \mu(b\epsilon + \epsilon' \bar{D}^A)\bar{D}^R.\end{aligned} \tag{29}$$

1.3 DNA methylation: reactions and models

Here, we provide reaction rate and ODE models of DNA methylation, based on the molecular mechanisms described in [5](Chapter 15) and [14](Chapter 17), using published DNA methylation models as a starting point [22, 23, 24, 25]. We then amend these models in order to reconcile inconsistencies between measured *in vivo* DNA de-methylation time scales [26] and *in vitro* data on the kinetics of TET enzymes [27] in light of recent new experimental data uncovering the role of MBD proteins on the *in vivo* activity of TET enzymes [28]. **Establishment, erasure, and maintenance**: we first start by writing a simple reaction and ODE model of DNA methylation, including *de novo* establishment by DNMT3, passive de-methylation through DNA replication, active de-methylation through TET enzymes, and maintenance methylation through DNMT1 enzyme [5](Chapter 15). The model starts with the definition of the molecular species involved, with the chemical reactions modeling the above processes, the corresponding ODEs, and is consistent with early models of DNA methylation proposed in the literature [22, 23, 24, 25], but contains a major addition. Specifically, we introduce a major addition to the model, which has not been considered before, in light of recent experimental evidence according to which the effective modification rate by TET enzymes is impacted by MBD proteins [29, 30, 28]. This addition is central to being able to predict the experimentally observed effects of MBDs knock down on important processes such as iPSC reprogramming efficiency and kinetics [29, 30].

The objective of the model is to capture the temporal dynamics of the total number of (single stranded) methylated CpGs within a given gene of interest. In particular, we assume for simplicity that the DNA wrapped around each nucleosome can have only one CpG and this means that the total modifiable (single stranded) CpGs coincide with the total number of modifiable nucleosomes, that is $\mathrm{D_{tot}}$. Therefore, let us then define D as a nucleosome with unmethylated single CpG in a gene of interest, $\mathrm{D^M}$ as a nucleosome with a methylated single CpG in a gene of interest and consider the model proposed by Laird et al.[31]. In this paper, they described the dynamics of $\mathrm{D^M}$ assuming *de novo* methylation, maintenance methylation and dilution due to DNA replication/ cell division. Based on this and introducing R, that is a sequence-specific repressor that can form multimer with n copies (R_n), which binds to D, creating the complex $\bar{\mathrm{C}}^0_R$ and then recruits DNMT3 writer of DNA methylation denoted by W^d to D, we write the following chemical reactions:

$$\mathrm{R} + \mathrm{R} + ... \underset{d_R}{\overset{a_R}{\rightleftharpoons}} \mathrm{R}_n, \qquad \mathrm{R}_n + \mathrm{D} \underset{\bar{d}_R}{\overset{\bar{a}_R}{\rightleftharpoons}} \bar{\mathrm{C}}^0_{\mathrm{R}}, \tag{30}$$

$$\mathrm{D} + \mathrm{W}^d \underset{d^d_W}{\overset{a^d_{W0}}{\rightleftharpoons}} \mathrm{C}^d_{\mathrm{W0}} \xrightarrow{\kappa^d_W} \mathrm{D^M} + \mathrm{W}^d, \qquad \bar{\mathrm{C}}^0_{\mathrm{R}} + \mathrm{W}^d \underset{d^d_W}{\overset{a^d_{W0} + a^d_W}{\rightleftharpoons}} \mathrm{C}^d_{\mathrm{W}} \xrightarrow{\kappa^d_W} \mathrm{D^M} + \mathrm{W}^d + \mathrm{R}_n \tag{31}$$

$$\mathrm{D^M} \xrightarrow{\delta'} \mathrm{D}, \tag{32}$$

in which C_W^d represents the complex between D and W^d, a_{W0}^d a_W^d and d_W^d are the association and dissociation rate constants, respectively, and κ_W^d is the catalytic rate constant of the enzymatic reactions (31). Furthermore, W^d can represent either the DNMT3 enzyme or a complex of the DNMT3 enzyme with a factor recruiting it to DNA, such as DNMT3L. Concerning reactions (30) and (31), they represent the DNA methylation *de novo* establishment. The DNMT3 enzyme can either bind directly to DNA or can be recruited to specific loci on DNA by sequence-specific TF [32, 33]. Reaction (32) represents passive demethylation, that is, the process by which DNA methylation is lost due to cell division. Rate constant δ' represents the effective passive erasure rate constant resulting from the balance between the dilution of DNA methylation due to DNA replication (occurring every T= $\frac{\ln(2)}{\delta}$) and the maintenance process by which DNMT1 copies CpG methylation on the newly generated DNA strand following the pattern on the mother strand [5](Chapter 15), [34],[35]). As it was derived in Laird et al [31], one can write δ' as follows:

$$\delta' = \delta\eta. \tag{33}$$

If there is not maintenance methylation, $\eta = 1$ and then $\delta' = \delta$, that is CpGs will halve at every cell division, while $\eta = 0$ if the maintenance process through DNMT1 enzyme is 100% efficient (all the CpG methylations are copied on the daughter DNA strand). Therefore, if we have a more efficient maintenance mechanism, this model leads to a larger half life of $\mathrm{D^M}$. As we did for the histone modification model, we can consider the complexes at their QSS and, defining $K^R = \frac{d^R}{a^R}$, $\bar{K}^R = \frac{\bar{d}^R}{\bar{a}^R}$ the dissociation constants of the first two reactions in (31), $K_{W0}^d = \frac{d_W^d + \kappa_{W0}^d}{a_W^d}$ and $K_W^d = \frac{d_W^d + \kappa_W^d}{a_W^d}$ the M-M constants of the enzymatic reactions in (31), the ODE model corresponding to these reactions is given by

$$\dot{D}^M = (k_{W0} + k_W)(D_{tot} - D^M) - \delta' D^M, \tag{34}$$

in which we define $k_{W0} = \kappa_W^d \frac{W^d}{K_{W0}^d}$ and $k_W = \kappa_W^d \frac{W^d}{K_W^d} \frac{\frac{R^n}{K_R \bar{K}_R}}{1 + \frac{R^n}{K_R \bar{K}_R}}$ (full derivation below). From this ODE we can represent the system through the following chemical reactions (depicted in the diagram of Fig AA in black):

$$\mathrm{D} \xrightarrow{\mathrm{kw_0}} \mathrm{D^M}, \quad k_{W0} = \kappa_W^d \frac{W^d}{K_{W0}^d}; \tag{35}$$

$$\mathrm{D} \xrightarrow{\mathrm{kw}} \mathrm{D^M}, \quad k_W = \kappa_W^d \frac{W^d}{K_W^d} \frac{\frac{R^n}{K_R \bar{K}_R}}{1 + \frac{R^n}{K_R \bar{K}_R}}; \tag{36}$$

$$\mathrm{D^M} \xrightarrow{\delta'} \mathrm{D}, \quad \delta' = \delta\eta; \tag{37}$$

Derivation of model (34)

The ODE model corresponding to reactions (30)-(32) is given by

$$\begin{aligned}
\dot{R}_n &= a_R R^n - d_R R_n - \delta R_n - \bar{a}_R R_n D + \bar{d}_R \bar{C}_R^0 + \kappa_W^d C_W^d \\
\dot{C}_{W0}^d &= a_{W0}^d D W^d - d_W^d C_{W0}^d - \kappa_W^d C_{W0}^d \\
\dot{C}_W^d &= (a_{W0}^d + a_W^d)\bar{C}_R^0 W^d - d_W^d C_W^d - \kappa_W^d C_W^d \\
\dot{\bar{C}}_R^0 &= \bar{a}_R R_n D - \bar{d}_R \bar{C}_R^0 - a_W^d \bar{C}_R^0 W^d + d_W^d C_W^d \\
\dot{D}^M &= \kappa_W^d C_{W0}^d + \kappa_W^d C_W^d - \delta' D^M.
\end{aligned} \tag{38}$$

As we did for the histone modification model, since the binding reactions are much faster than the other reactions, we set R_n, $\bar{C}_R^0$, C_{W0}^d and C_W^d to their QSS ($\dot{R}_n = \dot{\bar{C}}_R^0 = \dot{C}_{W0}^d = \dot{C}_W^d = 0$), obtaining

$$R_n = \frac{R^n}{K_R}, \quad \bar{C}_R^0 = \frac{R^n}{K_R \bar{K}_R} D, \quad C_{W0}^d = \frac{W^d}{K_{W0}^d} D,$$
$$C_W^d = (\frac{1}{K_{W0}^d} + \frac{1}{K_W^d}) W^d \bar{C}_R^0 = (\frac{1}{K_{W0}^d} + \frac{1}{K_W^d}) \frac{R^n W^d}{K_R \bar{K}_R K_W^d} D.$$

in which $K_{W0}^d = \frac{d_W^d + \kappa_W^d}{a_W^d}$ and $K_W^d = \frac{d_W^d + \kappa_W^d}{a_W^d}$, with $d_W^d, a_W^d, a_{W0}^d, \kappa_W^d$ defined as done in (31). Then, substituting these values in the last ODE of (38), we obtain

$$\begin{aligned} \dot{D}^M &= \kappa_W^d C_{W0}^d + \kappa_W^d C_W^d - \delta^{'} D^M \\ &= \left(\kappa_W^d \frac{W^d}{K_{W0}^d} + \kappa_W^d (\frac{1}{K_{W0}^d} + \frac{1}{K_W^d}) \frac{R^n W^d}{K_R \bar{K}_R} \right) D - \delta^{'} D^M \end{aligned} \tag{39}$$

D can be obtained by the DNA conservation law $D_{tot} = D + D^M + \bar{C}_R^0 + C_{W0}^d + C_W^d$ that, if we assume that the sum of the complexes C_{W0}^d and C_W^d is negligible with respect to $D + D^M + \bar{C}_R^0$ as we did for the previous models, can be approximated as

$$D_{tot} \approx D + D^M + \bar{C}_R^0. \tag{40}$$

Then, by considering the complexes dynamics to the QSS, D can be written as

$$D \approx \frac{D_{tot} - D^M}{1 + \frac{R^n}{K_R \bar{K}_R}}. \tag{41}$$

By substituting (41) in (39), the ODE model becomes

$$\begin{aligned} \dot{D}^M &= \left(\kappa_W^d \frac{W^d}{K_{W0}^d} + \kappa_W^d \frac{W^d}{K_W^d} \frac{\frac{R^n}{K_R \bar{K}_R}}{1 + \frac{R^n}{K_R \bar{K}_R}} \right) (D_{tot} - D^M) - \delta^{'} D^M \\ &= (k_{W0} + k_W)(D_{tot} - D^M) - \delta^{'} D^M. \end{aligned} \tag{42}$$

Here, we provide some estimate of the value of η, and hence of $\delta^{'}$. Specifically, we can estimate these parameters by comparing the kinetics of DNA demethylation in the absence ($\eta = 1$) or presence ($\eta < 1$) of DNMT1, both in the absence of DNMT3 ($k_W = k_{W0} = 0$). Specifically in [23], the authors transitioned mES cells from serum to 2i conditions and at that time, they also induced the deletion of DNMT1. Under these perturbations, which largely suppress DNMT3, we can estimate the half life of methylated CpGs, $\mathrm{D^M}$, to be about 1.7 cell divisions with the reported doubling time of 16 hours, corresponding to $\delta \approx 0.024\mathrm{hr}^{-1}$. This is in contrast to the kinetics observed in experiments with serum growth conditions and DNMT3a/b knocked off, corresponding to no *de novo* establishment ($k_{W0} = k_W = 0$) but presence of DNA methylation maintenance through DNMT1 enzyme, leading to $\delta' \approx 0.001\ \mathrm{hr}^{-1}$, giving $\eta \approx 0.04$, corresponding about 96% efficiency of the maintenance process. Note that the slightly larger half life observed in experiments than the time of cell division when both DNMT3a/b and DNMT1 are knocked off is potentially due to residual DNMT1 and DNMT3 and to the fact that DNMT3 can also act as a methylation maintenance enzyme [36].

In addition to passive erasure, DNA methylation can be actively removed through the TET enzymatic pathway [14](Chapter 17). TET1 is an enzyme that binds CpG-rich regions through its CxxC binding motif, which has a slight preference for unmethylated CpGs. TET2, instead, specifically recognizes CpG dinucleotides with a substrate preference for 5mC. Both have catalytic activity and are able to convert 5mC to 5hmC (hydroxilmethylated CpG), then to 5fc (formylcytosine), and finally to 5caC (carbolxylcytosine) [37, 38]. None of these modified forms are recognized by DNMT1 and therefore they are subject to dilution through DNA replication [14](Chapter 17). Here, we lump all these three different modified versions of CpGs into a species that we call $\mathrm{D_h^M}$, which is not subject to the maintenance reaction and only subject to dilution:

Active erasure of DNA methylation:

$$\mathrm{D^M} + \mathrm{T} \underset{d_T^d}{\overset{a_T^d}{\rightleftharpoons}} \mathrm{C_T^d} \xrightarrow{\kappa_\mathrm{T}^d} \mathrm{D}_h^\mathrm{M} + \mathrm{T}, \quad \mathrm{D}_h^\mathrm{M} \xrightarrow{\delta} \mathrm{D} \tag{43}$$

in which T represents the TET enzyme or a complex of the TET enzyme with a factor recruiting it to DNA, C_T^d represents the complex between $\mathrm{D^M}$ and T, a_T^d and d_T^d are the association and dissociation rate constants, respectively, and κ_T^d is the catalytic rate constant of the enzymatic reaction. If we introduce reaction (43), the ODE model (34) becomes

$$\begin{aligned} \dot{D}^M &= (k_{W0} + k_W)(D_{tot} - D^M - D_h^M) - k_T D^M - \delta' D^M \\ \dot{D}_h^M &= k_T D^M - \delta D_h^M, \end{aligned} \tag{44}$$

in which we defined $k_T = \kappa_T^d \frac{T}{K_T^d}$, with $K_T^d = ((d_T^d + \kappa_T^d)/a_T^d)$ the M-M constants of (43) and k_{W0} and k_W are defined as it was done in (34) (full derivation below). From this ODE we can represent the system through reactions (35), (36), (37) and the following ones corresponding to the TET pathway (depicted in the diagram of Fig AA in blue):

$$\begin{aligned} &\mathrm{D^M} \xrightarrow{\mathrm{k_T}} \mathrm{D}_h^\mathrm{M}, \quad k_T = \kappa_T^d \frac{T}{K_T^d}; \\ &\mathrm{D}_h^\mathrm{M} \xrightarrow{\delta} \mathrm{D^0}. \end{aligned} \tag{45}$$

Derivation of model (44)

The ODE model corresponding to reactions (30)-(43) is given by

$$\begin{aligned} \dot{R} &= -a_R R^n + d_R R_n \\ \dot{C}_{W0}^d &= a_{W0}^d D W^d - d_W^d C_{W0}^d - \kappa_W^d C_{W0}^d \\ \dot{C}_W^d &= (a_W^d + a_{W0}^d)\bar{C}_R^0 W^d - d_W^d C_W^d - \kappa_W^d C_W^d \\ \dot{\bar{C}}_R^0 &= \bar{a}_R R_n D - \bar{d}_R \bar{C}_R^0 - a_W^d \bar{C}_R^0 W^d + d_W^d C_W^d \\ \dot{C}_T^d &= a_T^d D^M T - d_T^d C_T^d - \kappa_T^d C_T^d \\ \dot{D}^M &= \kappa_W^d C_{W0}^d + \kappa_W^d C_W^d - \delta' D^M - a_T^d D^M T + d_T^d C_T^d \\ \dot{D}_h^M &= \kappa_T^d C_T^d - \delta D_h^M. \end{aligned} \tag{46}$$

Since the binding reactions are much faster than the other reactions, we set R_n, $\bar{C}_R^0$, C_{W0}^d, C_W^d and

C_T^d to their QSS ($\dot{R}_n = \dot{\bar{C}}_R^0 = \dot{C}_{W0}^d = \dot{C}_W^d = \dot{C}_M^d = \dot{C}_T^d = 0$), obtaining

$$
\begin{aligned}
&R_n = \frac{R^n}{K_R}, \quad \bar{C}_R^0 = \frac{R^n}{K_R \bar{K}_R} D, \quad C_{W0}^d = \frac{W^d}{K_{W0}^d} D, \\
&C_W^d = (\frac{1}{K_{W0}^d} + \frac{1}{K_W^d}) W^d \bar{C}_R^0 = (\frac{1}{K_{W0}^d} + \frac{1}{K_W^d}) \frac{R^n W^d}{K_R \bar{K}_R} D, \quad C_T^d = \frac{T}{K_T^d} D^M.
\end{aligned}
$$

in which $K_T^d = ((d_T^d + \kappa_T^d)/a_T^d)$ is the M-M constant (43), $K_{W0}^d = \frac{d_W^d + \kappa_{W0}^d}{a_W^d}$, $K_W^d = \frac{d_W^d + \kappa_W^d}{a_W^d}$ with $d_W^d, a_W^d, a_{W0}^d, \kappa_W^d$ defined as done in (31). Then, substituting these values in the last two ODEs of (46), we obtain

$$
\begin{aligned}
\dot{D}^M &= \kappa_W^d C_{W0}^d + \kappa_W^d C_W^d - \delta' D^M - \kappa_T C_T^d \\
&= \left(\kappa_W^d \frac{W^d}{K_{W0}^d} + \kappa_W^d (\frac{1}{K_{W0}^d} + \frac{1}{K_W^d}) \frac{R^n W^d}{K_R \bar{K}_R} \right) D - \delta' D^M - \kappa_T^d \frac{T}{K_T^d} D^M \\
\dot{D}_h^M &= \kappa_T^d C_T^d - \delta D_h^M \\
&= \kappa_T^d \frac{T}{K_T^d} D^M - \delta D_h^M.
\end{aligned} \tag{47}
$$

D can be obtained by the DNA conservation law $D_{tot} = D + D^M + D_h^M + \bar{C}_R^0 + C_{W0}^d + C_W^d + C_T^d$ that, if we assume that the sum of the complexes C_{W0}^d, C_W^d and C_T^d is negligible with respect to $D + D^M + D_h^M + \bar{C}_R^0$ as we did before, can be approximated as

$$
D_{tot} \approx D + D^M + D_h^M + \bar{C}_R^0. \tag{48}
$$

Then, by considering the complexes dynamics to the QSS, D can be written as

$$
D \approx \frac{D_{tot} - D^M - D_h^M}{1 + \frac{R^n}{K_R \bar{K}_R}}. \tag{49}
$$

By substituting (49) in (47), the ODE model becomes

$$
\begin{aligned}
\dot{D}^M &= \left(\kappa_W^d \frac{W^d}{K_{W0}^d} + \kappa_W^d \frac{W^d}{K_W^d} \frac{\frac{R^n}{K_R \bar{K}_R}}{1 + \frac{R^n}{K_R \bar{K}_R}} \right) (D_{tot} - D^M - D_h^M) - \left(\delta' + \kappa_T \frac{T}{K_T^d} \right) D^M \\
&= (k_{W0} + k_W) (D_{tot} - D^M - D_h^M) - (\delta' + k_T) D^M \\
\dot{D}_h^M &= \kappa_T^d \frac{T}{K_T^d} D^M - \delta D_h^M = k_T D^M - \delta D_h^M.
\end{aligned} \tag{50}
$$

The first decay term in the equation of D^M in (44) represents active de-methylation through the action of TET, while the second term is due to inefficient maintenance of the methylation marks through DNA replication. *In vitro* characterization of the kinetics of DNMT1 and TET enzymes have been performed and, although they are not necessarily the same as the kinetics encountered *in vivo*, they nevertheless provide a starting point to estimate the order of magnitude of k_T.

Specifically, *in vivo* experimental studies have shown that in the absence of DNMT3 ($k_{W0} = k_W = 0$) DNA methylation in ES cells goes from 22% to 0.6% in 216 cell divisions [39], which corresponds to $\delta' + k_T = 0.024\delta$. This would require a value of $k_T < 0.024\delta$. This order of magnitude is consistent with studies in ES cells estimating the ratio of hydroxilmethylated to methylated

DNA (k_T/δ in our model) to be in the range of $0.018 - 0.04$, depending on the TET level [40]. However, this is largely inconsistent with *in vitro* estimates of the TET enzyme catalytic constant (κ_T), estimated to be greater than $15.6\text{hr}^{-1} \approx 312\delta$ [27], which cannot lead to $k_T < 0.024\delta$ unless TET level in ES cells are at least 10,000X smaller than TET's M-M constant. This, in turn is highly unlikely given that ES cells are characterized by considerably high amounts of TET (see [14](Chapter 17), [41]).

This basic model therefore does not reflect the low effective values of k_T encountered *in vivo*. More recent experimental data demonstrated that the ability of TET enzymes *in vivo* to convert methylated DNA to hydorxilmethylated DNA (that, in our model considering the DNA wrapped around a nucleosome, coverting $\mathrm{D^M}$ to $\mathrm{D^M_h}$) is hampered by the binding of MBD proteins, denoted with B, to methylated DNA. MBD proteins MBD2/1 and MeCP2 recognize single methylated CpG dinucleotides and recruit both histone modifying and chromatin remodeling complexes to the methylated sites (see Section 1.4). It was shown that the binding of MBD2 or MeCP2 protein to methylated DNA protects it from binding by TET1 and that MBD2 KD leads to an increase in $\mathrm{D^M_h}$ level [28]. Reversely, MBD proteins cannot bind hydroxylmethylated DNA wrapped around a nucleosome, $\mathrm{D^M_h}$ [37]. To reflect these observations, we have to add the following reactions:

Reversible binding of MBD proteins B *to* $\mathrm{D^M}$*:*

$$\mathrm{D^M} + \mathrm{B} \underset{d^d_B}{\overset{a^d_B}{\rightleftharpoons}} \mathrm{C^d_B} \xrightarrow{\kappa^d_\mathrm{B}} \mathrm{C^0} + \mathrm{B} \tag{51}$$

in which B represents the MBD protein, C^d_B represents the complex between $\mathrm{D^M}$ and B, a^d_B and d^d_B are the association and dissociation rate constants, respectively, and κ^d_B is the catalytic rate constant of the enzymatic reaction.

Unbinding reaction:

$$\mathrm{C^0} \xrightarrow{d} \mathrm{D^M} \tag{52}$$

where d is the unbinding rate constant.

Removal of the methylation mark from $\mathrm{C^0}$ *through dilution:*

$$\mathrm{C^0} \xrightarrow{\delta'} \mathrm{D} \tag{53}$$

in which δ' is defined as it was done in (32).

The ODE model in equations (44) then modifies to

$$\begin{aligned}
\dot{D}^M &= (k_{W0} + k_W)\,(D_{tot} - D^M - D^M_h - C^0) - (\delta' + k_T + k_B)D^M + dC^0 \\
\dot{D}^M_h &= k_T D^M - \delta D^M_h \\
\dot{C}^0 &= k_B D^M - dC^0 - \delta' C^0.
\end{aligned} \tag{54}$$

in which we defined $k_B = \kappa^d_B \frac{B}{K^d_B}$ with $K^d_B = \frac{d^d_B + \kappa^d_B}{a^d_B}$, that is the M-M constant of reaction (51) and k_{W0}, k_W and k_T are defined as it was done in (44) (full derivation below).

Derivation of model (54)

The ODE model corresponding to reactions (30), (31), (32), (43), (51), (52), (53) is given by

$$\begin{aligned}
\dot{R} &= -a_R R^n + d_R R_n \\
\dot{C}^d_{W0} &= a^d_{W0} D W^d - d^d_W C^d_{W0} - \kappa^d_W C^d_{W0} \\
\dot{C}^d_W &= (a^d_{W0} + a^d_W)\bar{C}^0_R W^d - d^d_W C^d_W - \kappa^d_W C^d_W \\
\dot{\bar{C}}^0_R &= \bar{a}_R R_n D - \bar{d}_R \bar{C}^0_R - a^d_W \bar{C}^0_R W^d + d^d_W C^d_W \\
\dot{C}^d_T &= a^d_T D^M T - d^d_T C^d_T - \kappa^d_T C^d_T \\
\dot{C}^d_B &= a^d_B D^M B - d^d_B C^d_B - \kappa^d_B C^d_B \\
\dot{D}^M &= \kappa^d_W C^d_{W0} + \kappa^d_W C^d_W - \delta' D^M - a^d_T D^M T + d^d_T C^d_T - a^d_B D^M B + d^d_B C^d_B + dC^0 \\
\dot{C}^0 &= \kappa^d_B C^d_B - dC^0 - \delta' C^0 \\
\dot{D}^M_h &= \kappa^d_T C^d_T - \delta D^M_h .
\end{aligned} \tag{55}$$

Since the binding reactions are much faster than the other reactions, we set R_n, $\bar{C}^0_R$, C^d_{W0}, C^d_W, C^d_T and C^d_B to their QSS ($\dot{R}_n = \dot{\bar{C}}^0_R = \dot{C}^d_{W0} = \dot{C}^d_W = \dot{C}^d_M = \dot{C}^d_T = \dot{C}^d_B = 0$), obtaining

$$\begin{aligned}
&R_n = \frac{R^n}{K_R}, \quad \bar{C}^0_R = \frac{R^n}{K_R \bar{K}_R} D, \\
&C^d_W = (\frac{1}{K^d_{W0}} + \frac{1}{K^d_W}) W^d \bar{C}^0_R = (\frac{1}{K^d_{W0}} + \frac{1}{K^d_W}) \frac{R^n W^d}{K_R \bar{K}_R} D, \\
&C^d_{W0} = \frac{W^d}{K^d_{W0}} D, \quad C^d_T = \frac{T}{K^d_T} D^M, \quad C^d_B = \frac{B}{K^d_B} D^M
\end{aligned}$$

in which $K^d_T = \frac{d^d_T + \kappa^d_T}{a^d_T}$ and $K^d_B = \frac{d^d_B + \kappa^d_B}{a^d_B}$, that are the M-M constants of (43) and (51), respectively, and $K^d_{W0} = \frac{d^d_W + \kappa^d_{W0}}{a^d_W}$, $K^d_W = \frac{d^d_W + \kappa^d_W}{a^d_W}$, with $d^d_W, a^d_W, a^d_{W0}, \kappa^d_W$ defined as done in (31). Then, the model (55) becomes

$$\begin{aligned}
\dot{D}^M &= \kappa^d_W C^d_{W0} + \kappa^d_W C^d_W - \delta' D^M - \kappa_T C^d_T - \kappa^d_B C^d_B + dC^0 \\
&= \left(\kappa^d_W \frac{W^d}{K^d_{W0}} + \kappa^d_W (\frac{1}{K^d_{W0}} + \frac{1}{K^d_W}) \frac{R^n W^d}{K_R \bar{K}_R} \right) D - \delta' D^M - \kappa^d_T \frac{T}{K^d_T} D^M - \kappa^d_B \frac{B}{K^d_B} D^M + dC^0 \\
\dot{D}^M_h &= \kappa^d_T C^d_T - \delta D^M_h = \kappa^d_T \frac{T}{K^d_T} D^M - \delta D^M_h \\
\dot{C}^0 &= \kappa^d_B C^d_B - dC^0 - \delta' C^0 = \kappa^d_B \frac{B}{K^d_B} D^M - dC^0 - \delta' C^0 .
\end{aligned} \tag{56}$$

D can be obtained by the DNA conservation law $D_{tot} = D + D^M + D^M_h + \bar{C}^0_R + C^d_{W0} + C^d_W + C^d_T + C^d_B + C^0$ that, if we assume that the sum of the complexes C^d_{W0}, C^d_W, C^d_T and C^d_B is negligible with respect to $D + D^M + D^M_h + \bar{C}^0_R + C^0$ as we did before, can be approximated as

$$D_{tot} \approx D + D^M + D^M_h + \bar{C}^0_R + C^0 . \tag{57}$$

Then, by considering the complexes dynamics to the QSS, D can be written as

$$D \approx \frac{D_{tot} - D^M - D^M_h - C^0}{1 + \frac{R^n}{K_R \bar{K}_R}} . \tag{58}$$

By substituting (58) in (56), the ODE model becomes

$$\begin{aligned}
\dot{D}^M &= \left(\kappa_W^d \frac{W^d}{K_{W0}^d} + \kappa_W^d \frac{W^d}{K_W^d} \frac{\frac{R^n}{K_R \bar{K}_R}}{1+\frac{R^n}{K_R \bar{K}_R}}\right)(D_{tot} - D^M - D_h^M - C^0) \\
&- \left(\delta' + \kappa_T \frac{T}{K_T^d} + \kappa_B \frac{B}{K_B^d}\right) D^M + dC^0 \\
&= (k_{W0} + k_W)(D_{tot} - D^M - D_h^M - C^0) - (\delta' + k_T + k_B) D^M + dC^0 \\
\dot{D}_h^M &= \kappa_T^d \frac{T}{K_T^d} D^M - \delta D_h^M = k_T D^M - \delta D_h^M \\
\dot{C}^0 &= \kappa_B^d \frac{B}{K_B^d} D^M - dC^0 - \delta' C^0 = k_B D^M - dC^0 - \delta' C^0.
\end{aligned} \tag{59}$$

By studying these ODEs, we can simplify the MBD proteins binding reactions as follows:

$$\begin{aligned}
&\mathrm{D^M} \xrightarrow{\mathrm{k_B}} \mathrm{C^0}; \quad k_B = \kappa_B^d \frac{B}{K_B^d}; \\
&\mathrm{C^0} \xrightarrow{d} \mathrm{D^M}; \quad \mathrm{C^0} \xrightarrow{\delta'} \mathrm{D^0}.
\end{aligned} \tag{60}$$

The simplified reaction system representing the reactions making up the DNA methylation system, which account for the mutual protection mechanism between MBD proteins and TET binding to DNA is shown in Fig AB. Looking at the ODE model (54), we note that $k_B \gg d$ since MBD proteins are highly expressed in somatic tissues and about half of it is expressed in ES cells [30], suggesting a large B compared to K_B^d, and MBD and MeCP2 proteins stay bound even to mitotic chromosomes [42], suggesting $d < \delta$.

Calling the total single methylated CpGs $D_{tot}^M = C^0 + D^M$, letting $\bar{\delta} = d + \delta'$, substituting $D^M = D_{tot}^M - C^0$ and setting C^0 to its QSS, assuming d sufficiently larger than δ, we obtain the following ODE model:

$$\begin{aligned}
\dot{D}_{tot}^M &= (k_{W0} + k_W)(D_{tot} - D_{tot}^M - D_h^M) - (\delta' + k_T')D_{tot}^M \\
\dot{D}_h^M &= k_T' D_{tot}^M - \delta D_h^M,
\end{aligned} \tag{61}$$

with k_T' defined as follows:

$$k_T' = k_T \frac{\bar{\delta}}{k_B + \bar{\delta}}. \tag{62}$$

Letting $\mathrm{D_{tot}^M}$ represent any nucleosome with methylated CpG, with or without B bound to it, model (61) corresponds to the simplified diagram in Fig AC, whose reactions are the following:

$$\begin{aligned}
&\mathrm{D} \xrightarrow{\mathrm{k_{W0}}} \mathrm{D_{tot}^M}, \quad k_{W0} = \kappa_W^d \frac{W^d}{K_{W0}^d}; \\
&\mathrm{D} \xrightarrow{\mathrm{k_W}} \mathrm{D_{tot}^M}, \quad k_W = \kappa_W^d \frac{W^d}{K_W^d} \frac{\frac{R^n}{K_R \bar{K}_R}}{1+\frac{R^n}{K_R \bar{K}_R}}; \\
&\mathrm{D_{tot}^M} \xrightarrow{\mathrm{k_T'}} \mathrm{D}_h^\mathrm{M}, \quad k_T' = k_T \frac{\bar{\delta}}{k_B + \bar{\delta}}, k_T = \kappa_T^d \frac{T}{K_T^d}, k_B = \kappa_B^d \frac{B}{K_B^d}; \\
&\mathrm{D_{tot}^M} \xrightarrow{\delta'} \mathrm{D}; \\
&\mathrm{D}_h^\mathrm{M} \xrightarrow{\delta} \mathrm{D}.
\end{aligned} \tag{63}$$

From here, we observe that the effective active erasure rate constant $k_T^{'}$ is now decreased compared to k_T and it can be very small if k_B is large and $\bar{\delta}$ is small. Then, depending on the value of k_B, this model can now be consistent with experimental data demonstrating that knock down of MBD2 (decreased k_B) leads to global increase in hydroxylmethylation D_h^M [28] due to an increased $k_T^{'}$.

Model (61) can be further simplified. Specifically, since $\delta^{'} + k_T^{'} \ll \delta$, we can set D_h^M at the QSS, obtaining $D_h^M = \frac{k_T^{'}}{\delta} D_{tot}^M$. Furthermore, since $k_T^{'} \ll \delta$, we have that $D_h^M \ll D_{tot}^M$. Then, the final DNA methylation model can be written as follows:

$$\dot{D}_{tot}^M = (k_{W0} + k_W)(D_{tot} - D_{tot}^M) - (\delta' + k_T^{'})D_{tot}^M \tag{64}$$

and it is depicted in the diagram of Fig AD.

1.4 Cooperative interactions between DNA methylation and repressive H3K9me3 histone modifications: reactions

In order to develop a model that captures how DNA methylation and repressive histone modifications cooperate, we assume for simplicity that the DNA wrapped around each nucleosome can have only one CpG. Also, we assume that each nucleosome can have only one histone modification. Therefore, each nucleosome can either be unmodified, denoted D, or modified with H3K9me3, denoted $\mathrm{D_2^R}$, or modified with both H3K9me3 and DNA methylation, denoted $\mathrm{D_{12}^R}$, or modified only with DNA methylation, denoted by $\mathrm{D_1^R}$.

Now, let us describe the two possible pathways through which D can be modified. Pathway (A) represents the case in which DNA methylation is initially recruited to D to lead to nucleosome with DNA methylation, $\mathrm{D_1^R}$. Then, $\mathrm{D_1^R}$ recruites, *via* MBD and MeCP2, proteins Suv39H leading to a nucleosome characterized by both DNA methylation and H3K9me3, $\mathrm{D_{12}^R}$. DNA methylation is positively correlated with H3K9 methylation [5](Chapters 6, 22). In fact, MBD proteins recognize single methylated CpG dinucleotides and recruit both histone modifying and chromatin remodeling complexes to the methylated sites. MBD1, in particular, binds to methylated CpG sites and recruits histone methyltransferases for H3K9, SETDB1 and Suv39H1, which bring H3K9me3 about [43]. Similarly, MeCP2 binds methylated CpGs and recruits histone methylases that lead to H3K9me3 [44]. On the other hand, DNMT3/1 binds to HP1 protein, which is recruited to D by H3K9me3, suggesting that H3K9me3 recruits DNA methylation enzymes through HP1 protein [45]. Pathway (B) is also possible: it represents the case wherein H3K9me3 is specifically recruited to the nucleosome first and then DNA methylation is non-specifically recruited by H3K9me3. In practice, both pathways co-exist, that is, although the initial stimulus may be applied through one of these two pathways only, once there is some of $\mathrm{D_{12}^R}$ in the system, it can be converted back to D through either pathway. The diagram in which pathway (A) and (B) are concurrently present is represented in Fig C.

The reactions representing this cross-catalysis mechanism between repressive epigenetic marks described above are the following:

H3K9me3 recruits DNA methylation. Letting $\mathrm{C_{12}^0}$ represent a nucleosome with both a repressive histone modification (H3K9me3) and methylated DNA, bound to MBD; $\mathrm{D_{12h}^R}$ represent a nucleosome with both a repressive histone modification (H3K9me3) and hydroximethylated DNA; $\mathrm{W^d}$ represent the DNMT3 writer of DNA methylation and introducing R, that is a sequence-specific repressor that can form multimer with n copies (R_n), which binds to D, creating the complex $\mathrm{\bar{C}_R^0}$,

we have the following reactions:

$$
\begin{aligned}
&\mathrm{D}_2^{\mathrm{R}}+\mathrm{W}^d \underset{d_M^d}{\overset{a_M^d}{\rightleftharpoons}} \mathrm{M}_2^{'}, \quad \mathrm{D}_{12h}^{\mathrm{R}}+\mathrm{W}^d \underset{d_M^d}{\overset{a_M^d}{\rightleftharpoons}} \mathrm{M}_{12h}^{'}, \quad \mathrm{D}_{12}^{\mathrm{R}}+\mathrm{W}^d \underset{d_M^d}{\overset{a_M^d}{\rightleftharpoons}} \mathrm{M}_{12a}^{'}, \quad \mathrm{C}_{12}^{0}+\mathrm{W}^d \underset{d_M^d}{\overset{a_M^d}{\rightleftharpoons}} \mathrm{M}_{12b}^{'},\\
&\mathrm{D}+\mathrm{M}_2^{'} \underset{\bar{d}_1}{\overset{\bar{a}_1}{\rightleftharpoons}} \mathrm{C}_{\mathrm{R1}_1}^{'} \xrightarrow{\kappa_{\mathrm{M}}^{'}} \mathrm{D}_1^{\mathrm{R}}+\mathrm{M}_2^{'}, \qquad \bar{\mathrm{C}}_{\mathrm{R}}+\mathrm{M}_2^{'} \underset{\bar{d}_1}{\overset{\bar{a}_1}{\rightleftharpoons}} \mathrm{C}_{\mathrm{R1_{c1}}}^{'} \xrightarrow{\kappa_{\mathrm{M}}^{'}} \mathrm{D}_1^{\mathrm{R}}+\mathrm{M}_2^{'}+\mathrm{R}_n,\\
&\mathrm{D}+\mathrm{M}_{12h}^{'} \underset{\bar{d}_1}{\overset{\bar{a}_1}{\rightleftharpoons}} \mathrm{C}_{\mathrm{R1}_2}^{'} \xrightarrow{\kappa_{\mathrm{M}}^{'}} \mathrm{D}_1^{\mathrm{R}}+\mathrm{M}_{12h}^{'}, \qquad \bar{\mathrm{C}}_{\mathrm{R}}+\mathrm{M}_{12h}^{'} \underset{\bar{d}_1}{\overset{\bar{a}_1}{\rightleftharpoons}} \mathrm{C}_{\mathrm{R1c2}}^{'} \xrightarrow{\kappa_{\mathrm{M}}^{'}} \mathrm{D}_1^{\mathrm{R}}+\mathrm{M}_{12h}^{'}+\mathrm{R}_n,\\
&\mathrm{D}+\mathrm{M}_{12a}^{'} \underset{\bar{d}_1}{\overset{\bar{a}_1}{\rightleftharpoons}} \mathrm{C}_{\mathrm{R1_{3a}}}^{'} \xrightarrow{\kappa_{\mathrm{M}}^{'}} \mathrm{D}_1^{\mathrm{R}}+\mathrm{M}_{12a}^{'}, \qquad \bar{\mathrm{C}}_{\mathrm{R}}+\mathrm{M}_{12a}^{'} \underset{\bar{d}_1}{\overset{\bar{a}_1}{\rightleftharpoons}} \mathrm{C}_{\mathrm{R1_{c3a}}}^{'} \xrightarrow{\kappa_{\mathrm{M}}^{'}} \mathrm{D}_1^{\mathrm{R}}+\mathrm{M}_{12a}^{'}+\mathrm{R}_n,\\
&\mathrm{D}+\mathrm{M}_{12b}^{'} \underset{\bar{d}_1}{\overset{\bar{a}_1}{\rightleftharpoons}} \mathrm{C}_{\mathrm{R1_{3b}}}^{'} \xrightarrow{\kappa_{\mathrm{M}}^{'}} \mathrm{D}_1^{\mathrm{R}}+\mathrm{M}_{12b}^{'}, \qquad \bar{\mathrm{C}}_{\mathrm{R}}+\mathrm{M}_{12b}^{'} \underset{\bar{d}_1}{\overset{\bar{a}_1}{\rightleftharpoons}} \mathrm{C}_{\mathrm{R1_{c3b}}}^{'} \xrightarrow{\kappa_{\mathrm{M}}^{'}} \mathrm{D}_1^{\mathrm{R}}+\mathrm{M}_{12b}^{'}+\mathrm{R}_n,\\
&\mathrm{D}_2^{\mathrm{R}}+\mathrm{M}_2^{'} \underset{\bar{d}_1}{\overset{\bar{a}_1}{\rightleftharpoons}} \mathrm{C}_{\mathrm{R12}_1}^{'} \xrightarrow{\kappa_{\mathrm{M}}^{'}} \mathrm{D}_{12}^{\mathrm{R}}+\mathrm{M}_2^{'}, \qquad \mathrm{D}_2^{\mathrm{R}}+\mathrm{M}_{12h}^{'} \underset{\bar{d}_1}{\overset{\bar{a}_1}{\rightleftharpoons}} \mathrm{C}_{\mathrm{R12}_2}^{'} \xrightarrow{\kappa_{\mathrm{M}}^{'}} \mathrm{D}_{12}^{\mathrm{R}}+\mathrm{M}_{12h}^{'},\\
&\mathrm{D}_2^{\mathrm{R}}+\mathrm{M}_{12a}^{'} \underset{\bar{d}_1}{\overset{\bar{a}_1}{\rightleftharpoons}} \mathrm{C}_{\mathrm{R12_{3a}}}^{'} \xrightarrow{\kappa_{\mathrm{M}}^{'}} \mathrm{D}_{12}^{\mathrm{R}}+\mathrm{M}_{12a}^{'}, \qquad \mathrm{D}_2^{\mathrm{R}}+\mathrm{M}_{12b}^{'} \underset{\bar{d}_1}{\overset{\bar{a}_1}{\rightleftharpoons}} \mathrm{C}_{\mathrm{R12_{3b}}}^{'} \xrightarrow{\kappa_{\mathrm{M}}^{'}} \mathrm{D}_{12}^{\mathrm{R}}+\mathrm{M}_{12b}^{'},
\end{aligned}
\tag{65}
$$

in which $\mathrm{M}_2^{'}$ is the complex between $\mathrm{D}_2^{\mathrm{R}}$ and W^d, $\mathrm{M}_{12h}^{'}$ denotes the complex between D_{12h}^{R} and W^d, $\mathrm{M}_{12a}^{'}$ denotes the complex between $\mathrm{D}_{12}^{\mathrm{R}}$ and W^d, $\mathrm{M}_{12b}^{'}$ denotes the complex between C_{12}^{0} and W^d, $\mathrm{C}_{\mathrm{R1}_1}^{'}$ denotes the complex between D and $\mathrm{M}_2^{'}$, $\mathrm{C}_{\mathrm{R1}_2}^{'}$ denotes the complex between D and $\mathrm{M}_{12h}^{'}$, $\mathrm{C}_{R1_{3a}}^{'}$ denotes the complex between D and $\mathrm{M}_{12a}^{'}$, $\mathrm{C}_{R1_{3b}}^{'}$ denotes the complex between D and $\mathrm{M}_{12b}^{'}$, $\mathrm{C}_{R1_{c1}}^{'}$ denotes the complex between $\bar{\mathrm{C}}_R$ and $\mathrm{M}_2^{'}$, $\mathrm{C}_{R1_{c2}}^{'}$ denotes the complex between $\bar{\mathrm{C}}_R$ and $\mathrm{M}_{12h}^{'}$, $\mathrm{C}_{R1_{c3a}}^{'}$ denotes the complex between $\bar{\mathrm{C}}_R$ and $\mathrm{M}_{12a}^{'}$, $\mathrm{C}_{R1_{c3b}}^{'}$ denotes the complex between $\bar{\mathrm{C}}_R$ and $\mathrm{M}_{12b}^{'}$, $\mathrm{C}_{R12_1}^{'}$ denotes the complex between D_2^{R} and $\mathrm{M}_2^{'}$, $\mathrm{C}_{R12_2}^{'}$ denotes the complex between D_2^{R} and $\mathrm{M}_{12h}^{'}$, $\mathrm{C}_{R12_{3a}}^{'}$ denotes the complex between D_2^{R} and $\mathrm{M}_{12a}^{'}$, $\mathrm{C}_{R12_{3b}}^{'}$ denotes the complex between D_2^{R} and $\mathrm{M}_{12b}^{'}$, a_M^d, $\bar{a}_1$ and d_M^d, $\bar{d}_1$ are the association and dissociation rate constants, respectively, and $\kappa_M^{'}$ is the catalytic rate constant of the enzymatic reactions.

DNA methylation recruits H3K9me3. Letting C_1^0 represent a nucleosome without any histone modification but with methylated DNA, bound to MBD; $\mathrm{D}_{\mathrm{1h}}^{\mathrm{R}}$ represent a nucleosome without any histone modification but with hydroximethylated DNA; W^{R} represent the writer enzyme of H3K9me3, we have the following reactions:

$$
\begin{aligned}
&\mathrm{C}_1^0+\mathrm{W}^{\mathrm{R}} \underset{d_M^R}{\overset{a_M^R}{\rightleftharpoons}} \bar{\mathrm{M}}_{\mathrm{1tot}}, \quad \mathrm{C}_{12}^0+\mathrm{W}^{\mathrm{R}} \underset{d_M^R}{\overset{a_M^R}{\rightleftharpoons}} \bar{\mathrm{M}}_{\mathrm{12tot}},\\
&\mathrm{D}+\bar{\mathrm{M}}_{\mathrm{1tot}} \underset{\bar{d}_2}{\overset{\bar{a}_2}{\rightleftharpoons}} \bar{\mathrm{C}}_{\mathrm{R2}_1} \xrightarrow{\bar{\kappa}_{\mathrm{M}}} \mathrm{D}_2^{\mathrm{R}}+\bar{\mathrm{M}}_{\mathrm{1tot}}, \qquad \bar{\mathrm{C}}_{\mathrm{R}}+\bar{\mathrm{M}}_{\mathrm{1tot}} \underset{\bar{d}_2}{\overset{\bar{a}_2}{\rightleftharpoons}} \bar{\mathrm{C}}_{\mathrm{R2c1}} \xrightarrow{\bar{\kappa}_{\mathrm{M}}} \mathrm{D}_2^{\mathrm{R}}+\bar{\mathrm{M}}_{\mathrm{1tot}}+\mathrm{R}_n,\\
&\mathrm{D}+\bar{\mathrm{M}}_{\mathrm{12tot}} \underset{\bar{d}_2}{\overset{\bar{a}_2}{\rightleftharpoons}} \bar{\mathrm{C}}_{\mathrm{R2}_2} \xrightarrow{\bar{\kappa}_{\mathrm{M}}} \mathrm{D}_2^{\mathrm{R}}+\bar{M}_{\mathrm{12tot}}, \qquad \bar{\mathrm{C}}_{\mathrm{R}}+\bar{\mathrm{M}}_{\mathrm{12tot}} \underset{\bar{d}_2}{\overset{\bar{a}_2}{\rightleftharpoons}} \bar{\mathrm{C}}_{\mathrm{R2_{c2}}} \xrightarrow{\bar{\kappa}_{\mathrm{M}}} \mathrm{D}_2^{\mathrm{R}}+\bar{M}_{\mathrm{12tot}}+\mathrm{R}_n,\\
&\mathrm{D}_1^{\mathrm{R}}+\bar{\mathrm{M}}_{\mathrm{1tot}} \underset{\bar{d}_2}{\overset{\bar{a}_2}{\rightleftharpoons}} \bar{\mathrm{C}}_{\mathrm{R12}_1} \xrightarrow{\bar{\kappa}_{\mathrm{M}}} \mathrm{D}_{12}^{\mathrm{R}}+\bar{\mathrm{M}}_{\mathrm{1tot}}, \qquad \mathrm{D}_1^{\mathrm{R}}+\bar{\mathrm{M}}_{\mathrm{12tot}} \underset{\bar{d}_2}{\overset{\bar{a}_2}{\rightleftharpoons}} \bar{\mathrm{C}}_{\mathrm{R12}_2} \xrightarrow{\bar{\kappa}_{\mathrm{M}}} \mathrm{D}_{12}^{\mathrm{R}}+\bar{\mathrm{M}}_{\mathrm{12tot}},\\
&\mathrm{C}_1^0+\bar{\mathrm{M}}_{\mathrm{1tot}} \underset{\bar{d}_2}{\overset{\bar{a}_2}{\rightleftharpoons}} \bar{\mathrm{C}}_{\mathrm{R12}_1}^0 \xrightarrow{\bar{\kappa}_{\mathrm{M}}} \mathrm{C}_{12}^0+\bar{\mathrm{M}}_{\mathrm{1tot}}, \qquad \mathrm{C}_1^0+\bar{\mathrm{M}}_{\mathrm{12tot}} \underset{\bar{d}_2}{\overset{\bar{a}_2}{\rightleftharpoons}} \bar{\mathrm{C}}_{\mathrm{R12}_2}^0 \xrightarrow{\bar{\kappa}_{\mathrm{M}}} \mathrm{C}_{12}^0+\bar{\mathrm{M}}_{\mathrm{12tot}},\\
&\mathrm{D}_{1h}^{\mathrm{R}}+\bar{\mathrm{M}}_{\mathrm{1tot}} \underset{\bar{d}_2}{\overset{\bar{a}_2}{\rightleftharpoons}} \bar{\mathrm{C}}_{\mathrm{R12h}_1} \xrightarrow{\bar{\kappa}_{\mathrm{M}}} \mathrm{D}_{12h}^{\mathrm{R}}+\bar{\mathrm{M}}_{\mathrm{1tot}}, \qquad \mathrm{D}_{1h}^{\mathrm{R}}+\bar{\mathrm{M}}_{\mathrm{12tot}} \underset{\bar{d}_2}{\overset{\bar{a}_2}{\rightleftharpoons}} \bar{\mathrm{C}}_{\mathrm{R12h}_2} \xrightarrow{\bar{\kappa}_{\mathrm{M}}} \mathrm{D}_{12h}^{\mathrm{R}}+\bar{\mathrm{M}}_{\mathrm{12tot}},
\end{aligned}
\tag{66}
$$

in which $\bar{\mathrm{M}}_{1tot}$ denotes the complex between C_1^0 and W^R, $\bar{\mathrm{M}}_{12tot}$ denotes the complex between C_{12}^0 and W^R, $\bar{\mathrm{C}}_{R2_1}$ denotes the complex between D and $\bar{\mathrm{M}}_{1tot}$, $\bar{\mathrm{C}}_{R2_2}$ denotes the complex between D and $\bar{\mathrm{M}}_{12tot}$, $\bar{\mathrm{C}}_{R2_{c1}}$ denotes the complex between $\bar{\mathrm{C}}_R$ and $\bar{\mathrm{M}}_{1tot}$, $\bar{\mathrm{C}}_{R2_{c2}}$ denotes the complex between $\bar{\mathrm{C}}_R$ and $\bar{\mathrm{M}}_{12tot}$, $\bar{\mathrm{C}}_{R12_1}$ denotes the complex between D_1^R and $\bar{\mathrm{M}}_{1tot}$, $\bar{\mathrm{C}}_{R12_2}$ denotes the complex between D_1^R and $\bar{\mathrm{M}}_{12tot}$, $\bar{\mathrm{C}}^0_{R12_1}$ denotes the complex between C_1^0 and $\bar{\mathrm{M}}_{1tot}$, $\bar{\mathrm{C}}^0_{R12_2}$ denotes the complex between C_1^0 and $\bar{\mathrm{M}}_{12tot}$, $\bar{\mathrm{C}}_{R12h_1}$ denotes the complex between D_{1h}^R and $\bar{\mathrm{M}}_{1tot}$, $\bar{\mathrm{C}}_{R12h_2}$ denotes the complex between D_{1h}^R and $\bar{\mathrm{M}}_{12tot}$, a_M^R, $\bar{a}_2$ and d_M^R, $\bar{d}_2$ are the association and dissociation rate constants, respectively, and $\bar{\kappa}_M$ is the catalytic rate constant of the enzymatic reactions.

1.5 Competitive interactions between activating histone modifications and DNA methylation: reactions

As shown in Fig B, at a high level DNA methylation is correlated with the absence of H3K4 methylation [5](Chapter 6), as there is a mutual antagonism between these two modifications as follows. The Cfp1 protein specifically recognizes unmethylated CXXC DNA binding motif and recruits H3K4-specific lisyne methylases SET1, bringing about H3K4me3 [5](Chapter 1). Similarly, DNMT3L recognizes the absence of H3K4me3 and docks to the nucleosome DNMT3, bringing about de novo DNA methylation [5](Chapter 6, Section 3.1). In turn, it is known that proteins that contain the ADD (ATRX-DNMT3-DNMT3L) domain, that is, proteins of the DNMT3 family, do not associate with H3K4me3, which thus inhibits *de novo* DNA methylation [46]. Therefore, we model these two marks as mutually exclusive and let D represent an unmodified nucleosome, let $\mathrm{D^R}$ represent a nucleosome without any histone modification but with CpGme and $\mathrm{D^A}$ represent a nucleosome with a activating histone modification (H3K4me3 or H3Kac).

Active erasure of DNA methylation is enhanced by the presence of active marks. TET1 has enhanced propensity to bind to unmethylated CpGs through the CXXC domain [14](Chapter 17), [37]). This suggests a potential mechanism by which H3K4me3 in $\mathrm{D^A}$ recruits TET1, denoted T, to nearby methylated CpGs, enhancing the erasure of DNA methylation. Denoting the complex between and with T_{act}, this process can be described as follows:

$$\mathrm{D^A} + \mathrm{T} \underset{d_t^d}{\overset{a_t^d}{\rightleftharpoons}} \mathrm{T_{act}}, \qquad \mathrm{D^R} + \mathrm{T_{act}} \underset{d_T^d}{\overset{a_T^d}{\rightleftharpoons}} \mathrm{C}^d_{\mathrm{T_{act}}} \xrightarrow{\kappa_{\mathrm{T}}^d} \mathrm{D}_h^{\mathrm{R}} + \mathrm{T_{act}}. \tag{67}$$

These reactions have to be added to the DNA methylation erasure process without the presence of active marks described in (43) and rewritten here with the current notation of the nucleosome with DNA methylation, D^R:

$$\mathrm{D^R} + \mathrm{T} \underset{d_T^d}{\overset{a_T^d}{\rightleftharpoons}} \mathrm{C}^d_{\mathrm{T}} \xrightarrow{\kappa_{\mathrm{T}}^d} \mathrm{D}_h^{\mathrm{R}} + \mathrm{T}, \qquad \mathrm{D}_h^{\mathrm{R}} \xrightarrow{\delta} \mathrm{D}^0, \tag{68}$$

Active erasure of active histone modifications is enhanced by the presence of DNA methylation. Methylated CpGs recruit MeCP2 proteins, which associate with HDACs to establish histone de-acetylation and further chromatin compaction [5](Chapter 15), [43, 47, 48]. Similarly, methylated CpGs also recruit MBD2, which interacts with the NuRD complex to promote de-acetylation [43], [5](Chapter 21). These interactions can be modeled by a recruitment of erasers of the activating histone marks by methylated CpGs. That is, similar to what done for the competition between opposing histone marks, we have the following reactions for the active erasure of $\mathrm{D^R}$ on $\mathrm{D^A}$:

$$\mathrm{D^R} + \mathrm{E^A} \underset{d_e^A}{\overset{a_e^A}{\rightleftharpoons}} \mathrm{E^{Ad}_{act}}, \qquad \mathrm{D^A} + \mathrm{E^{Ad}_{act}} \underset{d_E^A}{\overset{a_E^A}{\rightleftharpoons}} \mathrm{C^{Ad}_E} \xrightarrow{\kappa_{\mathrm{E}}^{\mathrm{A}}} \mathrm{D} + \mathrm{E^{Ad}_{act}}. \tag{69}$$

1.6 Derivation of the complete model

By combining the competition interactions between activating and repressive marks of Figs 1C and B with the cooperation pathways among repressive marks of Fig C, we obtain the overall chromatin modification interactions, whose pictorial representation and interaction diagram are shown in Figs 1D and 3A, respectively. To realize the model, we assume that the DNA wrapped around each nucleosome can have only one CpG and that each nucleosome cannot be characterized by more than one modified histone simultaneously. Furthermore, as we saw in Section 1.5, activating histone modifications are anti-correlated with DNA methylation and then we assume that a nucleosome characterized by a activating histone modification cannot acquire CpG methylation. Conversely, as we saw in Section 1.4, repressive histone modifications are positively correlated with DNA methylation and then we assume that a nucleosome characterized by a repressive histone modification can acquire CpG methylation, and *viceversa*. Therefore, the species involved are the following: D (unmodified nucleosome), $\mathrm{D}_1^{\mathrm{R}}$ (nucleosome without any histone modification but with CpGme), $\mathrm{D}_2^{\mathrm{R}}$ (nucleosome with a repressive histone modification, H3K9me3, but without methylated CpG), $\mathrm{D}_{12}^{\mathrm{R}}$ (nucleosome with both H3K9me3 and CpGme) and $\mathrm{D}_{\mathrm{A}}^{\mathrm{R}}$ (nucleosome with a activating histone modification, H3K4me3 or H3Kac). In terms of notation, for a species X, we use n^X to denote the number of such a species and use italics, X, to denote concentration (defining the reaction volume as Ω, $X = \frac{n^X}{\Omega}$).

The reactions considered are the following:

- reactions (1), (16), (4) and the first reaction in (14), in which we substitute P, P_n, $\bar{\mathrm{C}}_P$, W, C_{W0}, C_W, D^M, M, E and C_E with R, R_n, $\bar{\mathrm{C}}_{R1}^0$, W^R, C_{W2}^R, D_2^R, M^R, E^R and C_{E2}^R, respectively;
- reactions (10), in which we substitute D^{R} with $\mathrm{D}_2^{\mathrm{R}}$, respectively;
- reactions (17), in which we substitute D^M, E, E_{act}, $\mathrm{C}_{E_{act}}$ and $\bar{\mathrm{D}}^M$ with D_2^R, E^R, $\mathrm{E}_{act_1}^R$, $\mathrm{C}_{E2_{act_1}}^R$ and D^A;
- reactions (17), in which we substitute D^M, E, E_{act}, $\mathrm{C}_{E_{act}}$ and $\bar{\mathrm{D}}^M$ with D_2^R, E^R, $\mathrm{E}_{act_2}^R$, $\mathrm{C}_{E2_{act_2}}^R$ and $\bar{\mathrm{C}}_A^A$, respectively;
- reactions (17), in which we substitute D^M, E, E_{act}, $\mathrm{C}_{E_{act}}$ and $\bar{\mathrm{D}}^M$ with D_2^R, E^R, $\mathrm{E}_{act_3}^R$, $\mathrm{C}_{E2_{act_3}}^R$ and $\bar{\mathrm{C}}_R^A$, respectively;
- reactions (16), (4) and the first reaction in (14), in which we substitute D, D^M, M, E and C_E with D_1^R, D_{12}^R, M_{12}^R, E^R and C_{E12}^R, respectively;
- reactions (17), in which we substitute D^M, E, E_{act}, $\mathrm{C}_{E_{act}}$ and $\bar{\mathrm{D}}^M$ with D_1^R, E^R, $\mathrm{E}_{act_1}^R$, $\mathrm{C}_{E12_{act_1}}^R$ and D^A, respectively;
- reactions (17), in which we substitute D^M, E, E_{act}, $\mathrm{C}_{E_{act}}$ and $\bar{\mathrm{D}}^M$ with D_1^R, E^R, $\mathrm{E}_{act_2}^R$, $\mathrm{C}_{E12_{act_2}}^R$ and $\bar{\mathrm{C}}_A^A$, respectively;
- reactions (17), in which we substitute D^M, E, E_{act}, $\mathrm{C}_{E_{act}}$ and $\bar{\mathrm{D}}^M$ with D_1^R, E^R, $\mathrm{E}_{act_3}^R$, $\mathrm{C}_{E12_{act_3}}^R$ and $\bar{\mathrm{C}}_R^A$, respectively;

- reactions (16), (4) and the first reaction in (14), in which we substitute D, D^M, M, E and C_E with D^R_{1h}, D^R_{12h}, M^R_{12h}, E^R and C^R_{E12h}, respectively;

- reaction (17), in which we substitute D^M, E, E_{act}, $C_{E_{act}}$ and $\bar{D}^M$ with D^R_{1h}, E^R, $E^R_{act_1}$, $C^R_{E12h_{act_1}}$ and D^A, respectively;

- reactions (17), in which we substitute D^M, E, E_{act}, $C_{E_{act}}$ and $\bar{D}^M$ with D^R_{1h}, E^R, $E^R_{act_2}$, $C^R_{E12h_{act_2}}$ and $\bar{C}^A_A$, respectively;

- reactions (17), in which we substitute D^M, E, E_{act}, $C_{E_{act}}$ and $\bar{D}^M$ with D^R_{1h}, E^R, $E^R_{act_3}$, $C^R_{E12h_{act_3}}$ and $\bar{C}^A_R$, respectively;

- reactions (16), (4) and the first reaction in (14), in which we substitute D, D^M, M, E and C_E with C^0_1, C^0_{12}, M^{R0}_{12}, E^R and C^{R0}_{E12}, respectively;

- reactions (17), in which we substitute D^M, E, E_{act}, $C_{E_{act}}$ and $\bar{D}^M$ with C^0_1, E^R, $E^R_{act_1}$, $C^{R0}_{E12_{act_1}}$ and D^A, respectively;

- reactions (17), in which we substitute D^M, E, E_{act}, $C_{E_{act}}$ and $\bar{D}^M$ with C^0_1, E^R, $E^R_{act_2}$, $C^{R0}_{E12_{act_2}}$ and $\bar{C}^A_A$, respectively;

- reactions (17), in which we substitute D^M, E, E_{act}, $C_{E_{act}}$ and $\bar{D}^M$ with C^0_1, E^R, $E^R_{act_3}$, $C^{R0}_{E12_{act_3}}$ and $\bar{C}^A_R$, respectively;

- reactions (14) and (15), in which we substitute D^R, M^R, C^R_M, $\bar{C}_R$, C^R_{M1}, $\bar{C}_A$ and C^R_{M2} with D^R_2, M^R, $C^R_{M2_1}$, $\bar{C}^0_{R1}$, $C^R_{M2_{c1}}$, $\bar{C}_A$ and $C^R_{M2_{ac1}}$, respectively;

- reactions (14) and (15), in which we substitute D^R, M^R, C^R_M, $\bar{C}_R$, C^R_{M1}, $\bar{C}_A$ and C^R_{M2} with D^R_2, M^R_{12}, $C^R_{M2_2}$, $\bar{C}^0_{R1}$, $C^R_{M2_{c2}}$, $\bar{C}_A$ and $C^R_{M2_{ac2}}$, respectively;

- reactions (14) and (15), in which we substitute D^R, M^R, C^R_M, $\bar{C}_R$, C^R_{M1}, $\bar{C}_A$ and $C^R_{M2}$with D^R_2, M^R_{12h}, $C^R_{M2_3}$, $\bar{C}^0_{R1}$, $C^R_{M2_{c3}}$, $\bar{C}_A$ and $C^R_{M2_{ac3}}$, respectively;

- reactions (14) and (15), in which we substitute D^R, M^R, C^R_M, $\bar{C}_R$, C^R_{M1}, $\bar{C}_A$ and C^R_{M2} with D^R_2, M^{R0}_{12}, $C^R_{M2_4}$, $\bar{C}^0_{R1}$, $C^R_{M2_{c4}}$, $\bar{C}_A$ and $C^R_{M2_{ac4}}$, respectively;

- the second reaction in (14), in which we substitute D, D^R, M^R and C^R_M with D^R_1, D^R_{12}, M^R and $C^R_{M12_1}$, respectively;

- the second reaction in (14), in which we substitute D, D^R, M^R and C^R_M with D^R_1, D^R_{12}, M^R_{12} and $C^R_{M12_2}$, respectively;

- the second reaction in (14), in which we substitute D, D^R, M^R and C^R_M with D^R_1, D^R_{12}, M^R_{12h} and $C^R_{M12_3}$, respectively;

- the second reaction in (14), in which we substitute D, D^R, M^R and C^R_M with D^R_1, D^R_{12}, M^{R0}_{12} and $C^R_{M12_4}$, respectively;

- the second reaction in (14), in which we substitute D, D^R, M^R and C^R_M with D^R_{1h}, D^R_{12h}, M^R and $C^R_{M12h_1}$, respectively;

- the second reaction in (14), in which we substitute D, D^R, M^R and C^R_M with D^R_{1h}, D^R_{12h}, M^R_{12} and $C^R_{M12h_2}$, respectively;
- the second reaction in (14), in which we substitute D, D^R, M^R and C^R_M with D^R_{1h}, D^R_{12h}, M^R_{12h} and $C^R_{M12h_3}$, respectively;
- the second reaction in (14), in which we substitute D, D^R, M^R and C^R_M with D^R_{1h}, D^R_{12h}, M^{R0}_{12} and $C^R_{M12h_4}$, respectively;
- the second reaction in (14), in which we substitute D, D^R, M^R and C^R_M with C^0_1, C^0_{12}, M^R and $C^{R0}_{M12_1}$, respectively;
- the second reaction in (14), in which we substitute D, D^R, M^R and C^R_M with C^0_1, C^0_{12}, M^R_{12} and $C^{R0}_{M12_2}$, respectively;
- the second reaction in (14), in which we substitute D, D^R, M^R and C^R_M with C^0_1, C^0_{12}, M^R_{12h} and $C^{R0}_{M12_3}$, respectively;
- the second reaction in (14), in which we substitute D, D^R, M^R and C^R_M with C^0_1, C^0_{12}, M^{R0}_{12} and $C^{R0}_{M12_4}$, respectively;
- reactions (30), (31), (32), (51), (52), (53) and (68), in which we substitute D^M, $\bar{C}^0_R$,C^d_{W0}, C^d_W, C^d_T, C^d_{B1}, D^M_h and C^0 with D^R_1, $\bar{C}^0_{R1}$, C^d_{W10}, C^d_{W1}, C^d_{T1}, C^d_{B1}, D^R_{1h} and C^0_1, respectively;
- reactions (10), in which we substitute W^R, $C^{R,2}_W$, and $\mathrm{D^R}$ with W^d, $C^{d,2}_{W1}$ and $\mathrm{D^R_1}$, respectively;
- reactions (67), in which we substitute T_{act}, D^R, $C^d_{T_{act}}$ and D^R_h with T_{act_1}, D^R_1, $C^d_{T1_{act_1}}$ and D^R_{1h}, respectively;
- reactions (67), in which we substitute D^A, T_{act}, D^R, $C^d_{T_{act}}$ and D^R_h with $\bar{C}^A_A$, T_{act_2}, D^R_1, $C^d_{T1_{act_2}}$ and D^R_{1h}, respectively;
- reactions (67), in which we substitute D^A, T_{act}, D^R, $C^d_{T_{act}}$ and D^R_h with $\bar{C}^A_R$, T_{act_3}, D^R_1, $C^d_{T1_{act_3}}$ and D^R_{1h}, respectively;
- reactions (32), (51), (52), (53) and (68) in which we substitute D, D^M, C^d_T, C^d_{B1}, D^M_h and C^0 with D^R_2, D^R_{12}, $\bar{C}^0_{R112}$, C^d_{T12}, C^d_{B12}, D^R_{12h} and C^0_{12}, respectively;
- reactions (67), in which we substitute T_{act}, D^R, $C^d_{T_{act}}$ and D^R_h with T_{act_1}, D^R_{12}, $C^d_{T12_{act_1}}$ and D^R_{12h}, respectively;
- reactions (67), in which we substitute D^A, T_{act}, D^R, $C^d_{T_{act}}$ and D^R_h with $\bar{C}^A_A$, T_{act_2}, D^R_{12}, $C^d_{T12_{act_2}}$ and D^R_{12h}, respectively;
- reactions (67), in which we substitute D^A, T_{act}, D^R, $C^d_{T_{act}}$ and D^R_h with $\bar{C}^A_R$, T_{act_3}, D^R_{12}, $C^d_{T12_{act_3}}$ and D^R_{12h}, respectively;
- reactions (65), (66);
- reactions (1), (16) and (4), in which we substitute P, P_n, $\bar{C}_P$, W, C_W, D^M, E and C_E with A, A_n, $\bar{C}_A$, W^A, C^A_W, D^A, E^A and C^A_E, respectively;

- reactions (65), (66);
- reactions (8), in which we substitute $\bar{C}_R$ with $\bar{C}^0_{R1}$, respectively;
- reactions (17), in which we substitute D^M, E, E_{act}, $C_{E_{act}}$ and $\bar{D}^M$ with D^A, E^A, $E^A_{act_1}$, $C^A_{E_{act_1}}$ and D^R_2;
- reactions (17), in which we substitute D^M, E, E_{act}, $C_{E_{act}}$ and $\bar{D}^M$ with D^A, E^A, $E^A_{act_2}$, $C^A_{E_{act_2}}$ and D^R_{12};
- reactions (17), in which we substitute D^M, E, E_{act}, $C_{E_{act}}$ and $\bar{D}^M$ with D^A, E^A, $E^A_{act_3}$, $C^A_{E_{act_3}}$ and D^R_{12h};
- reactions (17), in which we substitute D^M, E, E_{act}, $C_{E_{act}}$ and $\bar{D}^M$ with D^A, E^A, $E^A_{act_4}$, $C^A_{E_{act_4}}$ and C^0_{12};
- reactions (12) and (13), in which we substitute M^A, C^A_M, $\bar{C}_A$, C^A_{M1}, $\bar{C}_R$ and C^A_{M2} with M^A_1, $C^A_{M_1}$, $\bar{C}_A$, $C^A_{M1_1}$, $\bar{C}^0_{R1}$ and $C^A_{M2_1}$, respectively;
- reactions (12) and (13), in which we substitute D^A, M^A, C^A_M, $\bar{C}_A$, C^A_{M1}, $\bar{C}_R$ and C^A_{M2} with $\bar{C}^A_A$, M^A_2, $C^A_{M_2}$, $\bar{C}_A$, $C^A_{M1_2}$, $\bar{C}^0_{R1}$ and $C^A_{M2_2}$, respectively;
- reactions (12) and (13), in which we substitute D^A, M^A, C^A_M, $\bar{C}_A$, C^A_{M1}, $\bar{C}_R$ and C^A_{M2} with $\bar{C}^A_R$, M^A_3, $C^A_{M_3}$, $\bar{C}_A$, $C^A_{M1_3}$, $\bar{C}^0_{R1}$ and $C^A_{M2_3}$, respectively;
- reactions (69), in which we substitute D^R, E^{Ad}_{act}, D^A and C^{Ad}_E with D^R_1, $E^{Ad}_{act_1}$, D^A and $C^{Ad}_{E_1}$, respectively;
- reactions (69), in which we substitute D^R, E^{Ad}_{act}, D^A and C^{Ad}_E with C^0_1, $E^{Ad}_{act_2}$, D^A and $C^{Ad}_{E_2}$, respectively;
- reactions (69), in which we substitute D^R, E^{Ad}_{act}, D^A and C^{Ad}_E with D^R_{12}, $E^{Ad}_{act_3}$, D^A and $C^{Ad}_{E_3}$, respectively;
- reactions (69), in which we substitute D^R, E^{Ad}_{act}, D^A and C^{Ad}_E with C^0_{12}, $E^{Ad}_{act_4}$, D^A and $C^{Ad}_{E_4}$, respectively;
- reactions (69), in which we substitute D^R, E^{Ad}_{act}, D^A and C^{Ad}_E with D^R_1, $E^{Ad}_{act_1}$, $\bar{C}^A_A$ and $C^{Ad}_{AE_1}$, respectively;
- reactions (69), in which we substitute D^R, E^{Ad}_{act}, D^A and C^{Ad}_E with C^0_1, $E^{Ad}_{act_2}$, $\bar{C}^A_A$ and $C^{Ad}_{AE_2}$, respectively;
- reactions (69), in which we substitute D^R, E^{Ad}_{act}, D^A and C^{Ad}_E with D^R_{12}, $E^{Ad}_{act_3}$, $\bar{C}^A_A$ and $C^{Ad}_{AE_3}$, respectively;
- reactions (69), in which we substitute D^R, E^{Ad}_{act}, D^A and C^{Ad}_E with C^0_{12}, $E^{Ad}_{act_4}$, $\bar{C}^A_A$ and $C^{Ad}_{AE_4}$, respectively;
- reactions (69), in which we substitute D^R, E^{Ad}_{act}, D^A and C^{Ad}_E with D^R_1, $E^{Ad}_{act_1}$, $\bar{C}^A_R$ and $C^{Ad}_{RE_1}$, respectively;

- reactions (69), in which we substitute D^R, E^{Ad}_{act}, D^A and C^{Ad}_E with C^0_1, $E^{Ad}_{act_2}$, $\bar{C}^A_R$ and $C^{Ad}_{RE_2}$, respectively;
- reactions (69), in which we substitute D^R, E^{Ad}_{act}, D^A and C^{Ad}_E with D^R_{12}, $E^{Ad}_{act_3}$, $\bar{C}^A_R$ and $C^{Ad}_{RE_3}$, respectively;
- reactions (69), in which we substitute D^R, E^{Ad}_{act}, D^A and C^{Ad}_E with C^0_{12}, $E^{Ad}_{act_4}$, $\bar{C}^A_R$ and $C^{Ad}_{RE_4}$, respectively;

Defining $D^A_{tot} = D^A + \bar{C}^A_A + \bar{C}^A_R$, $D^R_{1tot} = D^R_1 + C^0_1$, $D^R_{12tot} = D^R_{12} + C^0_{12}$, $C^R_{M2} = C^R_{M2_1} + C^R_{M2_2} + C^R_{M2_3} + C^R_{M2_4}$, $C^R_{M12} = C^R_{M12_1} + C^R_{M12_2} + C^R_{M12_3} + C^R_{M12_4}$, $C^R_{M12h} = C^R_{M12h_1} + C^R_{M12h_2} + C^R_{M12h_3} + C^R_{M12h_4}$, $C^{R0}_{M12} = C^{R0}_{M12_1} + C^{R0}_{M12_2} + C^{R0}_{M12_3} + C^{R0}_{M12_4}$, $C^R_{M2_c} = C^R_{M2_{c1}} + C^R_{M2_{c2}} + C^R_{M2_{c3}} + C^R_{M2_{c4}}$, $C^{'}_{R1} = C^{'}_{R1_1} + C^{'}_{R1_2} + C^{'}_{R1_{3a}} + C^{'}_{R1_{3b}}$, $C^{'}_{R1_c} = C^{'}_{R1_{c1}} + C^{'}_{R1_{c2}} + C^{'}_{R1_{c3a}} + C^{'}_{R1_{c3b}}$, $C^{'}_{R12} = C^{'}_{R12_1} + C^{'}_{R12_2} + C^{'}_{R12_3}$, $\bar{C}_{R2} = \bar{C}_{R2_1} + \bar{C}_{R2_2}$, $\bar{C}_{R2_c} = \bar{C}_{R2_{c1}} + \bar{C}_{R2_{c2}}$, $\bar{C}_{R12} = \bar{C}_{R12_1} + \bar{C}_{R12_2}$, $\bar{C}^0_{R12} = \bar{C}^0_{R12_1} + \bar{C}^0_{R12_2}$, $\bar{C}_{R12h} = \bar{C}_{R12h_1} + \bar{C}_{R12h_2}$, $M^R_{tot} = M^R + M^R_{12} + M^R_{12h} + M^{R0}_{12}$, $M^{'} = M^{'}_2 + M^{'}_{12h} + M^{'}_{12a} + M^{'}_{12b}$, $\bar{M} = \bar{M}_{1tot} + \bar{M}_{12tot}$, $E^R_{act} = E^R_{act_1} + E^R_{act_2} + E^R_{act_3}$, $C^R_{E2_{act}} = C^R_{E2_{act_1}} + C^R_{E2_{act_2}} + C^R_{E2_{act_3}}$, $C^R_{E12_{act}} = C^R_{E12_{act_1}} + C^R_{E12_{act_2}} + C^R_{E12_{act_3}}$, $C^R_{E12h_{act}} = C^R_{E12h_{act_1}} + C^R_{E12h_{act_2}} + C^R_{E12h_{act_3}}$, $C^{R0}_{E12_{act}} = C^{R0}_{E12_{act_1}} + C^{R0}_{E12_{act_2}} + C^{R0}_{E12_{act_3}}$, $C^R_{M2_{ac}} = C^R_{M2_{ac1}} + C^R_{M2_{ac2}} + C^R_{M2_{ac3}} + C^R_{M2_{ac4}}$, $T_{act} = T_{act_1} + T_{act_2} + T_{act_3}$, $C^d_{T1_{act}} = C^d_{T1_{act_1}} + C^d_{T1_{act_2}} + C^d_{T1_{act_3}}$, $C^d_{T12_{act}} = C^d_{T12_{act_1}} + C^d_{T12_{act_2}} + C^d_{T12_{act_3}}$, $E^A_{act} = E^A_{act_1} + E^A_{act_2} + E^A_{act_3} + E^A_{act_4}$, $C^A_{E_{act}} = C^A_{E_{act_1}} + C^A_{E_{act_2}} + C^A_{E_{act_3}} + C^A_{E_{act_4}}$, $C^A_M = C^A_{M_1} + C^A_{M_2} + C^A_{M_3}$, $C^A_{M1} = C^A_{M1_1} + C^A_{M1_2} + C^A_{M1_3}$, $C^A_{M2} = C^A_{M2_1} + C^A_{M2_2} + C^A_{M2_3}$, $E^{Ad}_{act} = E^{Ad}_{act_1} + E^{Ad}_{act_2} + E^{Ad}_{act_3} + E^{Ad}_{act_4}$, $C^{Ad}_E = C^{Ad}_{E_1} + C^{Ad}_{E_2} + C^{Ad}_{E_3} + C^{Ad}_{E_4}$, $C^{Ad}_{AE} = C^{Ad}_{AE_1} + C^{Ad}_{AE_2} + C^{Ad}_{AE_3} + C^{Ad}_{AE_4}$, $C^{Ad}_{RE} = C^{Ad}_{RE_1} + C^{Ad}_{RE_2} + C^{Ad}_{RE_3} + C^{Ad}_{RE_4}$, $C^A_{E_{tot}} = C^A_E + \bar{C}^A_{AE} + \bar{C}^A_{RE}$, $C^A_{E_{act}tot} = C^A_{E_{act}} + \bar{C}^A_{AE_{act}} + \bar{C}^A_{RE_{act}}$, $C^{Ad}_{E_{tot}} = C^{Ad}_E + C^{Ad}_{AE} + C^{Ad}_{RE}$, the ODE system associated with the reaction system described above and

shown in Fig 3A is given by

$$
\begin{aligned}
\dot{A}_n &= a_A^n - d_A A_n - \delta A_n - \bar{a}_A A_n D + \bar{d}_A \bar{C}_A \\
&+ \kappa_W^A C_W^A + \kappa_W^A C_W^{A,2} + \kappa_W^A C_W^{A,2} + \kappa_M^A C_{M1}^A + \kappa_M^{'} C_{R1_{ac}}^{'} + \bar{\kappa}_M \bar{C}_{R2_{ac}} + \kappa_M C_{M2_{ac}}^R \\
&- \bar{a}_A^{'} A_n D^A + \bar{d}_A^{'} \bar{C}_A^A + \kappa_E^A \bar{C}_{AE}^A + \kappa_E^A \bar{C}_{AE_{act}}^A + \kappa_E^A \bar{C}_{AE}^{Ad} + \delta \bar{C}_A^A \\
\dot{R}_n &= a_R R^n - d_R R_n - \delta R_n - \bar{a}_R R_n D + \bar{d}_R \bar{C}_{R1}^0 + \kappa_W^d C_{W1}^d + \kappa_W^d C_W^{d,2} \\
&+ \kappa_W^2 C_{W2}^R + \kappa_W^2 C_W^{R,2} + \kappa_M C_{M2_c}^R + \kappa_M^{'} C_{R1_c}^{'} + \bar{\kappa}_M \bar{C}_{R2_c} + \kappa_M^A C_{M2}^A \\
&- \bar{a}_R^{'} R_n D^A + \bar{d}_R^{'} \bar{C}_R^A + \kappa_E^A \bar{C}_{RE}^A + \kappa_E^A \bar{C}_{RE_{act}}^A + \kappa_E^A \bar{C}_{RE}^{Ad} + \delta \bar{C}_R^A \\
\dot{\bar{C}}_{R1}^0 &= \bar{a}_R R_n D - \bar{d}_R \bar{C}_{R1}^0 - [(a_{W0}^d + a_W^d) \bar{C}_{R1}^0 W^d - d_W^d C_{W1}^d \\
&+ a_{W0}^A \bar{C}_{R1}^0 W^A - d_W^A C_W^{A,2} + (a_{W0}^R + a_W^R) \bar{C}_{R1}^0 W^R - d_W^R C_{W2}^R \\
&+ \bar{a}^R M_{tot}^R \bar{C}_{R1}^0 - \bar{d}^R C_{M2_c}^R + \bar{a}_1 \bar{C}_{R1}^0 M^{'} - \bar{d}_1 C_{R1_c}^{'} + \bar{a}_2 \bar{C}_{R1}^0 \bar{M} - \bar{d}_2 \bar{C}_{R2_c} \\
&+ \bar{a}^A M^A \bar{C}_{R1}^0 - \bar{d}^A C_{M2}^A + \bar{a}_1 \bar{C}_{R1}^0 M^{'} - \bar{d}_1 C_{R1_c}^{'} + \bar{a}_2 \bar{C}_{R1}^0 \bar{M} - \bar{d}_2 \bar{C}_{R2_c}] \\
\dot{C}_{W10}^d &= a_{W0}^d D W^d - d_W^d C_{W10}^d - \kappa_W^d C_{W10}^d \\
\dot{C}_{W1}^d &= (a_{W0}^d + a_W^d) \bar{C}_{R1}^0 W^d - d_W^d C_{W1}^d - \kappa_W^d C_{W1}^d \\
\dot{C}_{W1}^{d,2} &= a_{W0}^d \bar{C}_A W^d - d_W^d C_{W1}^{d,2} - \kappa_W^d C_{W1}^{d,2} \\
\dot{T}_{act} &= a_t^d D_{tot}^A T - d_t^d T_{act} - [a_T^d D_1^R T_{act} - d_T^d C_{T1_{act}}^d - \kappa_T^d C_{T1_{act}}^d \\
&+ a_T^d D_{12}^R T_{act} - d_T^d C_{T12_{act}}^d - \kappa_T^d C_{T12_{act}}^d] \\
\dot{C}_{T1}^d &= a_T^d D_1^R T - d_T^d C_{T1}^d - \kappa_T^d C_{T1}^d \\
\dot{C}_{T1_{act}}^d &= a_T^d D_1^R T_{act} - d_T^d C_{T1_{act}}^d - \kappa_T^d C_{T1_{act}}^d \\
\dot{C}_{B1}^d &= a_B^d D_1^R B - d_B^d C_{B1}^d - \kappa_B^d C_{B1}^d \\
\dot{C}_{W20}^R &= a_{W0}^R D W^R - d_W^R C_{W20}^R - \kappa_W^2 C_{W20}^R \\
\dot{C}_{W2}^R &= a_W^R \bar{C}_{R1}^0 W^R - d_W^R C_{W2}^R - \kappa_W^2 C_{W2}^R \\
\dot{C}_W^{R,2} &= a_W^R \bar{C}_A W^R - d_W^R C_W^{R,2} - \kappa_W^2 C_W^{R,2} \\
\dot{M}_{tot}^R &= a_M^R (D_2^R + D_{12h}^R + D_{12tot}^R) V^R - d_M^R M_{tot}^R - [\bar{a}^R M_{tot}^R D - \bar{d}^R C_{M2}^R - \kappa_M C_{M2}^R \\
&+ \bar{a}^R M_{tot}^R \bar{C}_{R1}^0 - \bar{d}^R C_{M2_c}^R - \kappa_M C_{M2_c}^R + \bar{a}^R D_1^R M_{tot}^R - \bar{d}^R C_{M12}^R - \kappa_M C_{M12}^R \\
&+ \bar{a}^R C_1^0 M_{tot}^R - \bar{d}^R C_{M12}^{R0} - \kappa_M C_{M12}^{R0} + \bar{a}^R D_{1h}^R M_{tot}^R - \bar{d}^R C_{M12h}^R - \kappa_M C_{M12h}^R] \\
\dot{C}_{M2}^R &= \bar{a}^R M_{tot}^R D - \bar{d}^R C_{M2}^R - \kappa_M C_{M2}^R \\
\dot{C}_{M2_c}^R &= \bar{a}^R M_{tot}^R \bar{C}_{R1}^0 - \bar{d}^R C_{M2_c}^R - \kappa_M C_{M2_c}^R \\
\dot{C}_{M2_{ac}}^R &= \bar{a}^R M_{tot}^R \bar{C}_A - \bar{d}^R C_{M2_{ac}}^R - \kappa_M C_{M2_{ac}}^R \\
\dot{E}_{act}^R &= a_e^R D_{tot}^A E^R - d_e^R E_{act}^A - [a_E^R D_2^R E_{act}^R - d_E^R C_{E2}^R - \kappa_E^R C_{E2}^R \\
&+ a_E^R D_{12}^R E_{act}^R - d_E^R C_{E12_{act}}^R - \kappa_E^R C_{E12_{act}}^R + a_E^R C_{12}^0 E_{act}^R - d_E^R C_{E12_{act}}^{R0} - \kappa_E^R C_{E12_{act}}^{R0} \\
&+ a_E^R D_{12h}^R E_{act}^R - d_E^R C_{E12h_{act}}^R - \kappa_E^R C_{E12h_{act}}^R] \\
\dot{C}_{E2}^R &= a_E^R D_2^R E^R - d_E^R C_{E2}^R - \kappa_E^R C_{E2}^R \\
\dot{C}_{E2_{act}}^R &= a_E^R D_2^R E_{act}^R - d_E^R C_{E2}^R - \kappa_E^R C_{E2_{act}}^R; \\
\dot{C}_{W120}^d &= a_{W0}^d D_2^R W^d - d_W^d C_{W120}^d - \kappa_W^d C_{W120}^d \\
\dot{C}_{T12}^d &= a_T^d D_{12}^R T - d_T^d C_{T12}^d - \kappa_T^d C_{T12}^d \\
\dot{C}_{T12_{act}}^d &= a_T^d D_{12}^R T_{act} - d_T^d C_{T12_{act}}^d - \kappa_T^d C_{T12_{act}}^d \\
\dot{C}_{B12}^d &= a_B^d D_{12}^R B - d_B^d C_{B12}^d - \kappa_B^d C_{B12}^d \\
\dot{C}_{W120}^R &= a_{W0}^R D_1^R W^R - d_W^R C_{W120}^R - \kappa_W^2 C_{W120}^R
\end{aligned}
$$

$$
\begin{aligned}
\dot{C}^{R}_{M12} &= \bar{a}^{R} D^{R}_{1} M^{R}_{tot} - \bar{d}^{R} C^{R}_{M12} - \kappa_M C^{R}_{M12} \\
\dot{C}^{R}_{E12} &= a^{R}_{E} D^{R}_{12} E^{R} - d^{R}_{E} C^{R}_{E12} - \kappa^{R}_{E} C^{R}_{E12} \\
\dot{C}^{R}_{E12_{act}} &= a^{R}_{E} D^{R}_{12} E^{R}_{act} - d^{R}_{E} C^{R}_{E12_{act}} - \kappa^{R}_{E} C^{R}_{E12_{act}} \\
\dot{C}^{R0}_{W120} &= a^{R}_{W0} C^{0}_{1} W^{R} - d^{R}_{W} C^{R0}_{W120} - \kappa^{2}_{W} C^{R0}_{W120} \\
\dot{C}^{R0}_{M12} &= \bar{a}^{R} C^{0}_{1} M^{R}_{tot} - \bar{d}^{R} C^{R0}_{M12} - \kappa_M C^{R0}_{M12} \\
\dot{C}^{R0}_{E12} &= a^{R}_{E} C^{0}_{12} E^{R} - d^{R}_{E} C^{R0}_{E12} - \kappa^{R}_{E} C^{R0}_{E12} \\
\dot{C}^{R0}_{E12_{act}} &= a^{R}_{E} C^{0}_{12} E^{R}_{act} - d^{R}_{E} C^{R0}_{E12_{act}} - \kappa^{R}_{E} C^{R0}_{E12_{act}} \\
\dot{C}^{R}_{W12h0} &= a^{R}_{W0} D^{R}_{1h} W^{R} - d^{R}_{W} C^{R}_{W12h0} - \kappa^{2}_{W} C^{R}_{W12h0} \\
\dot{C}^{R}_{M12h} &= \bar{a}^{R} D^{R}_{1h} M^{R}_{tot} - \bar{d}^{R} C^{R}_{M12h} - \kappa_M C^{R}_{M12h} \\
\dot{C}^{R}_{E12h} &= a^{R}_{E} D^{R}_{12h} E^{R} - d^{R}_{E} C^{R}_{E12h} - \kappa^{R}_{E} C^{R}_{E12h} \\
\dot{C}^{R}_{E12h_{act}} &= a^{R}_{E} D^{R}_{12h} E^{R}_{act} - d^{R}_{E} C^{R}_{E12h_{act}} - \kappa^{R}_{E} C^{R}_{E12h_{act}} \\
\dot{M}' &= a^{d}_{M}(D^{R}_{2} + D^{R}_{12h} + D^{R}_{12tot}) W^{d} - d^{d}_{M} M' \\
&\quad - [\bar{a}_1 D M' - \bar{d}_1 C'_{R1} - \kappa'_M C'_{R1} \\
&\quad + \bar{a}_1 \bar{C}^{0}_{R1} M' - \bar{d}_1 C'_{R1_c} - \kappa'_M C'_{R1_c} + \bar{a}_1 D^{R}_{2} M' - \bar{d}_1 C'_{R12} - \kappa'_M C'_{R12} \\
&\quad + \bar{a}_1 \bar{C}_A M' - \bar{d}_1 C'_{R1_{ac}} - \kappa'_M C'_{R1_{ac}}] \\
\dot{\bar{M}} &= a^{R}_{M}(C^{0}_{1} + C^{0}_{12}) W^{R} - d^{R}_{M} \bar{M} - [\bar{a}_2 D \bar{M} - \bar{d}_2 \bar{C}_{R2} - \kappa_M \bar{C}_{R2} \\
&\quad + \bar{a}_2 \bar{C}^{0}_{R1} \bar{M} - \bar{d}_2 \bar{C}_{R2_c} - \bar{\kappa}_M \bar{C}_{R2_c} + \bar{a}_2 D^{R}_{1} \bar{M} - \bar{d}_2 \bar{C}_{R12} - \bar{\kappa}_M \bar{C}_{R12} \\
&\quad + \bar{a}_2 C^{0}_{1} \bar{M} - \bar{d}_2 \bar{C}^{0}_{R12} - \bar{\kappa}_M \bar{C}^{0}_{R12} + \bar{a}_2 D^{R}_{1h} \bar{M} - \bar{d}_2 \bar{C}_{R12h} - \bar{\kappa}_M \bar{C}_{R12h} \\
&\quad + \bar{a}_2 \bar{C}_A \bar{M} - \bar{d}_2 \bar{C}_{R2_{ac}} - \bar{\kappa}_M \bar{C}_{R2_{ac}}] \\
\dot{C}'_{R1} &= \bar{a}_1 D M' - \bar{d}_1 C'_{R1} - \kappa'_M C'_{R1} \\
\dot{C}'_{R1_c} &= \bar{a}_1 \bar{C}^{0}_{R1} M' - \bar{d}_1 C'_{R1_c} - \kappa'_M C'_{R1_c} \\
\dot{C}'_{R1_{ac}} &= \bar{a}_1 \bar{C}_A M' - \bar{d}_1 C'_{R1_{ac}} - \kappa'_M C'_{R1_{ac}} \\
\dot{\bar{C}}_{R2} &= \bar{a}_2 D \bar{M} - \bar{d}_2 \bar{C}_{R2} - \bar{\kappa}_M \bar{C}_{R2} \\
\dot{\bar{C}}_{R2_c} &= \bar{a}_2 \bar{C}^{0}_{R1} \bar{M} - \bar{d}_2 \bar{C}_{R2_c} - \bar{\kappa}_M \bar{C}_{R2_c} \\
\dot{\bar{C}}_{R2_{ac}} &= \bar{a}_2 \bar{C}_A \bar{M} - \bar{d}_2 \bar{C}_{R2_{ac}} - \bar{\kappa}_M \bar{C}_{R2_{ac}} \\
\dot{C}'_{R12} &= \bar{a}_1 D^{R}_{2} M' - \bar{d}_1 C'_{R12} - \kappa'_M C'_{R12} \\
\dot{\bar{C}}_{R12} &= \bar{a}_2 D^{R}_{1} \bar{M} - \bar{d}_2 \bar{C}_{R12} - \bar{\kappa}_M \bar{C}_{R12} \\
\dot{\bar{C}}^{0}_{R12} &= \bar{a}_2 C^{0}_{1} \bar{M} - \bar{d}_2 \bar{C}^{0}_{R12} - \bar{\kappa}_M \bar{C}^{0}_{R12} \\
\dot{\bar{C}}_{R12h} &= \bar{a}_2 D^{R}_{1h} \bar{M} - \bar{d}_2 \bar{C}_{R12h} - \bar{\kappa}_M \bar{C}_{R12h} \\
\dot{\bar{C}}_{A} &= \bar{a}_A A_n D - \bar{d}_A \bar{C}_A - [(a^{A}_{W0} + a^{A}_{W}) \bar{C}_A W^{A} - d^{A}_{W} C^{A}_{W} + \bar{a}^{A} \bar{C}_A M^{A} - \bar{d}^{A} C^{A}_{M1} \\
&\quad + a^{R}_{W} \bar{C}_A W^{R} - d^{R}_{W} C^{R,2}_{W} + a^{d}_{W0} \bar{C}_A W^{d} - d^{d}_{W} C^{d,2}_{W1} \\
&\quad + \bar{a}^{R} \bar{C}_A M^{R}_{tot} - \bar{d}^{R} C^{R}_{M2_{ac}} + \bar{a}_1 \bar{C}_A M' - \bar{d}_1 C'_{R1_{ac}} + \bar{a}_2 \bar{C}_A \bar{M} - \bar{d}_2 \bar{C}_{R2_{ac}}] \\
\dot{C}^{A}_{W0} &= a^{A}_{W0} D W^{A} - d^{A}_{W} C^{A}_{W0} - \kappa^{A}_{W} C^{A}_{W0} \\
\dot{C}^{A}_{W} &= (a^{A}_{W0} + a^{A}_{W}) \bar{C}_A W^{A} - d^{A}_{W} C^{A}_{W} - \kappa^{A}_{W} C^{A}_{W} \\
\dot{C}^{A,2}_{W} &= a^{A}_{W0} \bar{C}^{0}_{R1} W^{A} - d^{A}_{W} C^{A,2}_{W} - \kappa^{A}_{W} C^{A,2}_{W} \\
\dot{M}^{A} &= a^{A}_{M} D^{A}_{tot} V^{A} - d^{A}_{M} M^{A} - [\bar{a}^{A} D M^{A} - \bar{d}^{A} C^{A}_{M} - \kappa^{A}_{M} C^{A}_{M} \\
&\quad + \bar{a}^{A} \bar{C}_A M^{A} - \bar{d}^{A} C^{A}_{M1} - \kappa^{A}_{M} C^{A}_{M1} + \bar{a}^{A} \bar{C}_R M^{A} - \bar{d}^{A} C^{A}_{M2} - \kappa^{A}_{M} C^{A}_{M2}
\end{aligned}
\tag{70}
$$

$$
\begin{aligned}
\dot{C}^A_M &= \bar{a}^A D M^A - \bar{d}^A C^A_M - \kappa^A_M C^A_M \\
\dot{C}^A_{M1} &= \bar{a}^A \bar{C}_A M^A - \bar{d}^A C^A_{M1} - \kappa^A_M C^A_{M1} \\
\dot{C}^A_{M2} &= \bar{a}^A \bar{C}^0_{R1} M^A - \bar{d}^A C^A_{M2} - \kappa^A_M C^A_{M2} \\
\dot{E}^A_{act} &= a^A_e (D^R_2 + D^R_{12h} + D^R_{12tot}) E^A - d^A_e E^A_{act} - [a^A_E D^A_{tot} E^A_{act} - d^A_E C^A_{E_{act}tot} - \kappa^A_E C^A_{E_{act}tot}] \\
\dot{E}^{Ad}_{act} &= a^A_e (D^R_{1tot} + D^R_{12tot}) E^A - d^A_e E^{Ad}_{act} - [a^A_E D^A_{tot} E^{Ad}_{act} - d^A_E C^{Ad}_{E_{tot}} - \kappa^A_E C^{Ad}_{E_{tot}}] \\
\dot{\bar{C}}^A_{AE} &= a^A_E \bar{C}^A_A E^A - d^A_E \bar{C}^A_{AE} - \kappa^A_E \bar{C}^A_{AE} \\
\dot{\bar{C}}^A_{RE} &= a^A_E \bar{C}^A_R E^A - d^A_E \bar{C}^A_{RE} - \kappa^A_E \bar{C}^A_{RE} \\
\dot{C}^A_{Etot} &= a^A_E D^A_{tot} E^A - d^A_E C^A_{Etot} - \kappa^A_E C^A_{Etot} \\
\dot{\bar{C}}^A_{AE_{act}} &= a^A_E \bar{C}^A_A E^A_{act} - d^A_E \bar{C}^A_{AE_{act}} - \kappa^A_E \bar{C}^A_{AE_{act}} \\
\dot{\bar{C}}^A_{RE_{act}} &= a^A_E \bar{C}^A_R E^A_{act} - d^A_E \bar{C}^A_{RE_{act}} - \kappa^A_E \bar{C}^A_{RE_{act}} \\
\dot{C}^A_{E_{act}tot} &= a^A_E D^A_{tot} E^A_{act} - d^A_E C^A_{E_{act}tot} - \kappa^A_E C^A_{E_{act}tot} \\
\dot{\bar{C}}^{Ad}_{AE} &= a^A_E \bar{C}^A_A E^{Ad}_{act} - d^A_E \bar{C}^{Ad}_{AE} - \kappa^A_E \bar{C}^{Ad}_{AE} \\
\dot{\bar{C}}^{Ad}_{RE} &= a^A_E \bar{C}^A_R E^{Ad}_{act} - d^A_E \bar{C}^{Ad}_{RE} - \kappa^A_E \bar{C}^{Ad}_{RE} \\
\dot{C}^{Ad}_{E_{tot}} &= a^A_E D^A_{tot} E^{Ad}_{act} - d^A_E C^{Ad}_{E_{tot}} - \kappa^A_E C^{Ad}_{E_{tot}} \\
\dot{\bar{C}}^A_A &= \bar{a}'_A A_n D^A - \bar{d}'_A \bar{C}^A_A - a^A_E \bar{C}^A_A E^A + d^A_E \bar{C}^A_{AE} - a^A_E \bar{C}^A_A E^A_{act} + d^A_E \bar{C}^A_{AE_{act}} - a^A_E \bar{C}^A_A E^{Ad}_{act} + d^A_E \bar{C}^{Ad}_{AE} - \delta \bar{C}^A_A \\
\dot{\bar{C}}^A_R &= \bar{a}'_R R_n D^A - \bar{d}'_R \bar{C}^A_R - a^A_E \bar{C}^A_R E^A + d^A_E \bar{C}^A_{RE} - a^A_E \bar{C}^A_R E^A_{act} + d^A_E \bar{C}^A_{RE_{act}} - a^A_E \bar{C}^A_R E^{Ad}_{act} + d^A_E \bar{C}^{Ad}_{RE} - \delta \bar{C}^R_A \\
\dot{D}^R_1 &= \kappa^d_W (C^d_{W10} + C^d_{W1} + C^{d,2}_{W1}) + \kappa'_M (C'_{R1} + C'_{R1_c} + C'_{R1_{ac}}) \\
&\quad - \delta' D^R_1 - a^d_B D^R_1 B + d^d_B C^d_{B1} + dC^0_1 - a^d_T D^R_1 (T + T_{act}) + d^d_T (C^d_{T1} + C^d_{T1_{act}}) - a^A_e D^R_1 E^A + d^A_e E^{Ad}_{act_1} \\
&\quad - [a^R_{W0} D^R_1 W^R - d^R_W C^R_{W120} + \bar{a}^R D^R_1 M^R_{tot} - \bar{d}^R C^R_{M12} + \bar{a}_2 D^R_1 \bar{M} - \bar{d}_2 \bar{C}_{R12} - \kappa^R_E (C^R_{E12} + C^R_{E12_{act}}) - \delta D^R_{12}] \\
\dot{C}^0_1 &= \kappa^d_B C^d_{B1} - dC^0_1 - \delta' C^0_1 - a^R_M C^0_1 W^R + d^R_M \bar{M}_{1tot} - a^A_e C^0_1 E^A + d^A_e E^{Ad}_{act_2} \\
&\quad - [a^R_{W0} C^0_1 W^R - d^R_W C^{R0}_{W120} + \bar{a}^R C^0_1 M^R_{tot} - \bar{d}^R C^{R0}_{M12} + \bar{a}_2 C^0_1 \bar{M} - \bar{d}_2 \bar{C}^0_{R12} - \kappa^R_E (C^{R0}_{E12} + C^{R0}_{E12_{act}}) - \delta C^0_{12}] \\
\dot{D}^R_{1h} &= \kappa^d_T (C^d_{T1} + C^d_{T1_{act}}) - \delta D^R_{1h} \\
&\quad - [a^R_{W0} D^R_{1h} W^R - d^R_W C^R_{W12h0} + \bar{a}^R D^R_{1h} M^R_{tot} - \bar{d}^R C^R_{M12h} \\
&\quad + \bar{a}_2 D^R_{1h} \bar{M} - \bar{d}_2 \bar{C}_{R12h} - \kappa^R_E (C^R_{E12h} + C^R_{E12h_{act}}) - \delta D^R_{12h}] \\
\dot{D}^R_2 &= \kappa^2_W (C^R_{W20} + C^R_{W2} + C^{R,2}_W) + \kappa_M (C^R_{M2} + C^R_{M2_c} + C^R_{M2_{ac}}) + \bar{\kappa}_M (\bar{C}_{R2} + \bar{C}_{R2_c} + \bar{C}_{R2_{ac}}) \\
&\quad - a^R_E D^R_2 (E^R + E^R_{act}) + d^R_E (C^R_{E2} + C^R_{E2_{act}}) - \delta D^R_2 - a^A_e D^R_2 E^A + d^A_e E^A_{act_1} \\
&\quad - [a^d_{W0} D^R_2 W^d - d^d_W C^d_{W120} + a^R_M D^R_2 W^R - d^R_M M^R + \bar{a}_1 D^R_2 M' - \bar{d}_1 C'_{R12} - \delta' D^R_{12} - \delta D^R_{12h} - \delta' C^0_{12}] \\
\dot{D}^R_{12} &= \kappa^2_W C^R_{W120} + \kappa_M C^R_{M12} + \bar{\kappa}_M \bar{C}_{R12} - a^R_E D^R_{12} (E^R + E^R_{act}) + d^R_E (C^R_{E12} + C^R_{E12_{act}}) - \delta D^R_{12} \\
&\quad + \kappa^d_W C^d_{W120} + \kappa'_M C'_{R12} - \delta' D^R_{12} - a^d_B D^R_{12} B + d^d_B C^d_{B12} + dC^0_{12} \\
&\quad - a^A_e D^R_{12} E^A + d^A_e E^A_{act_2} - a^A_e D^R_{12} E^A + d^A_e E^{Ad}_{act_4} \\
&\quad - a^d_T D^R_{12} (T + T_{act}) - d^d_T (C^d_{T12} + C^d_{T12_{act}}) - a^R_M D^R_{12} W^R + d^R_M M^R_{12} \\
\dot{C}^0_{12} &= \kappa^d_B C^d_{B12} - dC^0_{12} - \delta' C^0_{12} - a^R_M C^0_{12} W^R + d^R_M \bar{M}_{12tot} \\
&\quad - a^A_e C^0_{12} E^A + d^A_e E^A_{act_4} - a^A_e C^0_{12} E^A + d^A_e E^{Ad}_{act_3} \\
&\quad + [\kappa^2_W C^{R0}_{W120} + \kappa_M C^{R0}_{M12} + \bar{\kappa}_M \bar{C}^0_{R12} - a^R_E C^0_{12} (E^R + E^R_{act}) + d^R_E (C^{R0}_{E12} + C^{R0}_{E12_{act}}) \\
&\quad - \delta C^0_{12} - a^R_M C^0_{12} W^R + d^R_M M^{R0}_{12}] \\
\dot{D}^R_{12h} &= \kappa^d_T (C^d_{T12} + C^d_{T12_{act]}}) - \delta D^R_{12h} \\
&\quad + [\kappa^2_W C^R_{W12h0} + \kappa_M C^R_{M12h} + \bar{\kappa}_M \bar{C}_{R12h} - a^R_E D^R_{12h} (E^R + E^R_{act}) + d^R_E (C^R_{E12h} + C^R_{E12h_{act}}) \\
&\quad - \delta D^R_{12h} - a^R_M D^R_{12h} W^R + d^R_M M^R_{12h}] - a^A_e D^R_{12h} E^A + d^A_e E^A_{act_3}
\end{aligned}
$$

$$\dot{D}^A_{tot} = \kappa^A_W(C^A_{W0} + C^A_W + C^{A,2}_W) + \kappa^A_M(C^A_M + C^A_{M1} + C^A_{M2}) - a^A_M D^A_{tot} W^A + d^A_M M^A - a^R_e D^A_{tot} E^R + d^R_e E^A_{act} - a^A_E D^A_{tot}(E^A + E^A_{act} + E^{Ad}_{act}) + d^A_E(C^A_{E_{tot}} + C^A_{E_{act}tot} + C^{Ad}_{E_{tot}}) - \delta D^A.$$

All the reaction rate constants involved are defined as it was done in (1), (4), (12), (13), (14), (15), (16), (17), (30), (31), (32), (51), (52), (53) and (68), (67), (69). Since the binding reactions are much faster than the other reactions, we set the complexes dynamics to the QSS, that is we set $\dot{A}_n = \dot{R}_n = \dot{\bar{C}}^0_{R1} = \dot{C}^{d,2}_{W1} = \dot{C}^d_{W10} = \dot{C}^d_{W1} = \dot{T}_{act} = \dot{C}^d_{T1} = \dot{C}^d_{T1_{act}} = \dot{C}^d_{B1} = \dot{C}^R_{W20} = \dot{C}^R_{W2} = \dot{C}^{R,2}_W = \dot{M}^R_{tot} = \dot{C}^R_{M2} = \dot{C}^R_{M2_c} = \dot{C}^R_{M2_{ac}} = \dot{E}^R_{act} = \dot{C}^R_{E2} = \dot{C}^R_{E2_{act}} = \dot{C}^d_{W120} = \dot{C}^d_{T12} = \dot{C}^d_{T12_{act}} = \dot{C}^d_{B12} = \dot{C}^R_{W120} = \dot{C}^R_{M12} = \dot{C}^R_{E12} = \dot{C}^R_{E12_{act}} = \dot{C}^{R0}_{W120} = \dot{C}^{R0}_{M12} = \dot{C}^{R0}_{E12} = \dot{C}^{R0}_{E12_{act}} = \dot{C}^R_{W12h0} = \dot{C}^R_{M12h} = \dot{C}^R_{E12h} = \dot{C}^R_{E12h_{act}} = \dot{M}' = \dot{M} = \dot{C}'_{R1} = \dot{C}'_{R1_c} = \dot{C}'_{R1_{ac}} = \dot{\bar{C}}_{R2} = \dot{\bar{C}}_{R2_c} = \dot{\bar{C}}_{R2_{ac}} = \dot{C}'_{R12} = \dot{\bar{C}}_{R12} = \dot{\bar{C}}^0_{R12} = \dot{\bar{C}}_{R12h} = \dot{\bar{C}}_A = \dot{C}^A_{W0} = \dot{C}^A_{W2} = \dot{C}^A_W = \dot{M}^A = \dot{C}^A_M = \dot{C}^A_{M1} = \dot{C}^A_{M2} = \dot{E}^A_{act} = \dot{E}^{Ad}_{act} = \dot{C}^A_{Etot} = \dot{C}^A_{E_{act}tot} = \dot{C}^{Ad}_{E_{tot}} = 0$, obtaining

$$R_n = \frac{R^n}{K_R}, \quad \bar{C}^0_{R1} = \frac{R^n}{K_R\bar{K}_R}D, \quad A_n = \frac{A^n}{K_A}, \quad \bar{C}_A = \frac{A^n}{K_A\bar{K}_A}D, \quad C^d_{W10} = \frac{a^d_{w0}W^dD}{d^d_W + \kappa^d_W} = \frac{W^d}{K^d_{W0}}D,$$

$$C^d_{W1} = \frac{(a^d_{W0} + a^d_W)W^d\bar{C}^0_{R1}}{d^d_W + \kappa^d_W} = (\frac{1}{K^d_{W0}} + \frac{1}{K^d_W})W^d\bar{C}^0_{R1} = (\frac{1}{K^d_{W0}} + \frac{1}{K^d_W})\frac{W^dR^n}{K_R\bar{K}_R}D,$$

$$C^{d,2}_{W1} = \frac{a^d_{W0}W^d\bar{C}_A}{d^d_W + \kappa^d_W} = \frac{W^d}{K^d_{W0}}\bar{C}_A = \frac{W^dA^n}{K^d_{W0}K_A\bar{K}_A}D,$$

$$C^R_{W20} = \frac{a^R_{W0}W^RD}{d^R_W + \kappa^2_W} = \frac{W^R}{K^R_{W0}}D, \quad T_{act} = \frac{a^d_tD^A_{tot}T}{d^d_t} = \frac{D^A_{tot}T}{K_{TT}},$$

$$C^R_{W2} = \frac{(a^R_{W0} + a^R_W)W^R\bar{C}^0_{R1}}{d^R_W + \kappa^2_W} = (\frac{1}{K^R_{W0}} + \frac{1}{K^R_W})W^R\bar{C}^0_{R1} = (\frac{1}{K^R_{W0}} + \frac{1}{K^R_W})\frac{W^RR^n}{K_R\bar{K}_R}D,$$

$$C^{R,2}_W = \frac{a^R_{W0}W^R\bar{C}_A}{d^R_W + \kappa^2_W} = \frac{W^R}{K^R_{W0}}\bar{C}_A = \frac{W^RA^n}{K^R_{W0}K_A\bar{K}_A}D,$$

$$C^d_{B1} = \frac{a^d_BBD^R_1}{d^d_B + \kappa^d_B} = \frac{B}{K^d_B}D^R_1, \quad C^d_{T1} = \frac{a^d_TTD^R_1}{d^d_T + \kappa^d_T} = \frac{T}{K^d_T}D^R_1, \quad C^d_{T1_{act}} = \frac{T_{act}}{K^d_T}D^R_1,$$

$$C^d_{W120} = \frac{a^d_{w0}W^dD^R_2}{d^d_W + \kappa^d_W} = \frac{W^d}{K^d_{W0}}D^R_2, \quad C^d_{B12} = \frac{a^d_BBD^R_{12}}{d^d_B + \kappa^d_B} = \frac{B}{K^d_B}D^R_{12}, \quad C^d_{T12} = \frac{a^d_TTD^R_{12}}{d^d_T + \kappa^d_T} = \frac{T}{K^d_T}D^R_{12},$$

$$C^d_{T1_{act}} = \frac{T_{act}}{K^d_T}D^R_1 = \frac{TD^A_{tot}}{K_{TT}K^d_T}D^R_1 = \frac{TD^A_{tot}}{K^*_T}D^R_1, \quad C^d_{T12_{act}} = \frac{T_{act}}{K^d_T}D^R_{12} = \frac{TD^A_{tot}}{K^*_T}D^R_{12},$$

$$M^R_{tot} = \frac{a^R_MV^R(D^R_2 + D^R_{12h} + D^R_{12tot})}{d^R_M} = \frac{V^R(D^R_2 + D^R_{12h} + D^R_{12tot})}{K^R_{MM}}$$

$$C^R_{M2} = \frac{\bar{a}^RM^R_{tot}D}{\bar{d}^R + \kappa_M} = \frac{V^R(D^R_2 + D^R_{12h} + D^R_{12tot})}{\bar{K}^R_MK^R_{MM}}D = \frac{V^R(D^R_2 + D^R_{12h} + D^R_{12tot})}{K^R_M}D,$$

$$C^R_{M2_c} = \frac{\bar{a}^RM^R_{tot}\bar{C}^0_{R1}}{\bar{d}^R + \kappa_M} = \frac{V^R(D^R_2 + D^R_{12h} + D^R_{12tot})}{\bar{K}^R_MK^R_{MM}}\bar{C}^0_{R1} = \frac{R^nV^R(D^R_2 + D^R_{12h} + D^R_{12tot})}{K_R\bar{K}_RK^R_M}D,$$

$$C^R_{M2_{ac}} = \frac{\bar{a}^RM^R_{tot}\bar{C}_A}{\bar{d}^R + \kappa_M} = \frac{V^R(D^R_2 + D^R_{12h} + D^R_{12tot})}{\bar{K}^R_MK^R_{MM}}\bar{C}_A = \frac{A^nV^R(D^R_2 + D^R_{12h} + D^R_{12tot})}{K_A\bar{K}_AK^R_M}D,$$

$$E^R_{act}=\frac{a^R_eD^A_{tot}E^R}{d^R_e}=\frac{D^A_{tot}E^R}{K^R_{EE}},\;\;C^R_{E2}=\frac{a^R_EE^RD^R_2}{d^R_E+\kappa^R_E}=\frac{E^R}{K^R_E}D^R_2,\;\;C^R_{E12}=\frac{a^R_EE^RD^R_{12}}{d^R_E+\kappa^R_E}=\frac{E^R}{K^R_E}D^R_{12},$$

$$C^R_{E2_{act}}=\frac{E^R_{act}}{K^R_E}D^R_2=\frac{E^RD^A_{tot}}{K^R_{EE}K^R_E}D^R_2=\frac{E^RD^A_{tot}}{K^{R*}_E}D^R_2,\;\;C^R_{E12_{act}}=\frac{E^R_{act}}{K^R_E}D^R_{12}=\frac{E^RD^A_{tot}}{K^{R*}_E}D^R_{12},$$

$$C^R_{W120}=\frac{a^R_{W0}W^RD^R_1}{d^R_W+\kappa^2_W}=\frac{W^R}{K^R_{W0}}D^R_1,\;\;C^{R0}_{W120}=\frac{a^R_{W0}W^RC^0_1}{d^R_W+\kappa^2_W}=\frac{W^R}{K^R_{W0}}C^0_1,$$

$$C^R_{M12}=\frac{\bar{a}^RM^R_{tot}D^R_1}{\bar{d}^R_M+\kappa_M}=\frac{V^R(D^R_2+D^R_{12h}+D^R_{12tot})}{\bar{K}^R_MK^R_{MM}}D^R_1=\frac{V^R(D^R_2+D^R_{12h}+D^R_{12tot})}{K^R_M}D^R_1,$$

$$C^{R0}_{M12}=\frac{\bar{a}^RM^R_{tot}C^0_1}{\bar{d}^R_M+\kappa_M}=\frac{V^R(D^R_2+D^R_{12h}+D^R_{12tot})}{\bar{K}^R_MK^R_{MM}}C^0_1=\frac{V^R(D^R_2+D^R_{12h}+D^R_{12tot})}{K^R_M}C^0_1,$$

$$C^{R0}_{E12}=\frac{a^R_EE^RC^0_{12}}{d^R_E+\kappa^R_E}=\frac{E^R}{K^R_E}C^0_{12},\;\;C^R_{E12h}=\frac{a^R_EE^RD^R_{12h}}{d^R_E+\kappa^R_E}=\frac{E^R}{K^R_E}D^R_{12h},\;\;C^R_{W12h0}=\frac{a^R_{W0}W^RD^R_{1h}}{d^R_W+\kappa^2_W}=\frac{W^R}{K^R_{W0}}D^R_{1h},$$

$$C^{R0}_{E12}=\frac{E^R_{act}}{K^R_E}C^0_{12}=\frac{E^RD^A_{tot}}{K^R_{EE}K^R_E}C^0_{12}=\frac{E^RD^A_{tot}}{K^{R*}_E}C^0_{12},\;\;C^R_{E12h}=\frac{E^R_{act}}{K^R_E}D^R_{12h}=\frac{E^RD^A_{tot}}{K^{R*}_E}D^R_{12h},$$

$$C^R_{M12h}=\frac{\bar{a}^RM^R_{tot}D^R_{1h}}{d^R_M+\kappa_M}=\frac{V^R((D^R_2+D^R_{12h}+D^R_{12tot})}{\bar{K}^R_MK^R_{MM}}D^R_{1h}=\frac{V^R(D^R_2+D^R_{12h}+D^R_{12tot})}{K^R_M}D^R_{1h},$$

$$M^{'}=\frac{a^d_MW^d(D^R_2+D^R_{12h}+D^R_{12tot})}{d^d_M}=\frac{W^d(D^R_2+D^R_{12h}+D^R_{12tot})}{K^d_{MM}},$$

$$C^{'}_{R1}=\frac{\bar{a}_1DM^{'}}{\bar{d}_1+\kappa^{'}_M}=\frac{W^d(D^R_2+D^R_{12h}+D^R_{12tot})}{\bar{K}^R_1K^d_{MM}}D=\frac{W^d(D^R_2+D^R_{12h}+D^R_{12tot})}{K^{'}_M}D,$$

$$C^{'}_{R1_c}=\frac{\bar{a}_1\bar{C}^0_{R1}M^{'}}{\bar{d}_1+\kappa^{'}_M}=\frac{W^d(D^R_2+D^R_{12h}+D^R_{12tot})}{\bar{K}^R_1K^d_{MM}}\bar{C}^0_{R1}=\frac{R^nW^d(D^R_2+D^R_{12h}+D^R_{12tot})}{K_R\bar{K}_RK^{'}_M}D,$$

$$C^{'}_{R1_{ac}}=\frac{\bar{a}_1\bar{C}_AM^{'}}{\bar{d}_1+\kappa^{'}_M}=\frac{W^d(D^R_2+D^R_{12h}+D^R_{12tot})}{\bar{K}^R_1K^d_{MM}}\bar{C}_A=\frac{A^nW^d(D^R_2+D^R_{12h}+D^R_{12tot})}{K_A\bar{K}_AK^{'}_M}D,$$

$$\bar{M}=\frac{a^R_MW^R(C^0_1+C^0_{12})}{d^R_M}=\frac{W^R(C^0_1+C^0_{12})}{K^R_{MM}},$$

$$\bar{C}_{R2}=\frac{\bar{a}_2D\bar{M}}{\bar{d}_2+\bar{\kappa}_M}=\frac{W^R(C^0_1+C^0_{12})}{K^R_2K^R_{MM}}D=\frac{W^R(C^0_1+C^0_{12})}{\bar{K}_M}D,$$

$$\bar{C}_{R2_c}=\frac{\bar{a}_2\bar{C}^0_{R1}\bar{M}}{\bar{d}_2+\bar{\kappa}_M}=\frac{W^R(C^0_1+C^0_{12})}{K^R_2K^R_{MM}}\bar{C}^0_{R1}=\frac{R^nW^R(C^0_1+C^0_{12})}{K_R\bar{K}_R\bar{K}_M}D,$$

$$\bar{C}_{R2_c}=\frac{\bar{a}_2\bar{C}_A\bar{M}}{\bar{d}_2+\bar{\kappa}_M}=\frac{W^R(C^0_1+C^0_{12})}{K^R_2K^R_{MM}}\bar{C}_A=\frac{A^nW^R(C^0_1+C^0_{12})}{K_A\bar{K}_A\bar{K}_M}D,$$

$$C^{'}_{R12}=\frac{\bar{a}_1D^R_2M^{'}}{\bar{d}_1+\kappa^{'}_M}=\frac{W^d(D^R_2+\bar{C}^0_{R112}+D^R_{12h}+D^R_{12tot})}{\bar{K}^R_1K^d_{MM}}D^R_2=\frac{W^d(D^R_2+\bar{C}^0_{R112}+D^R_{12h}+D^R_{12tot})}{K^{'}_M}D^R_2,$$

$$\bar{C}_{R12}=\frac{\bar{a}_2D^R_1\bar{M}}{\bar{d}_2+\bar{\kappa}_M}=\frac{W^R(C^0_1+C^0_{12})}{K^R_2K^R_{MM}}D^R_1=\frac{W^R(C^0_1+C^0_{12})}{\bar{K}_M}D^R_1,$$

$$\bar{C}^0_{R12} = \frac{\bar{a}_2 C^0_1 \bar{M}}{\bar{d}_2 + \bar{\kappa}_M} = \frac{W^R(C^0_1 + C^0_{12})}{K^R_2 K^R_{MM}} C^0_1 = \frac{W^R(C^0_1 + C^0_{12})}{\bar{K}_M} C^0_1,$$

$$\bar{C}_{R12h} = \frac{\bar{a}_2 D^R_{1h} \bar{M}}{\bar{d}_2 + \bar{\kappa}_M} = \frac{W^R(C^0_1 + C^0_{12})}{K^R_2 K^R_{MM}} D^R_{1h} = \frac{W^R(C^0_1 + C^0_{12})}{\bar{K}_M} D^R_{1h},$$

$$C^A_W = \frac{(a^A_{W0} + a^A_W) W^A \bar{C}_A}{d^A_W + \kappa^A_W} = (\frac{1}{K^A_{W0}} + \frac{1}{K^A_W}) W^A \bar{C}_A = (\frac{1}{K^A_{W0}} + \frac{1}{K^A_W}) \frac{W^A A^n}{K_A \bar{K}_A} D,$$

$$C^A_{W0} = \frac{a^A_{W0} W^A D}{d^A_W + \kappa^A_W} = \frac{W^A}{K^A_{W0}} D, \;\; C^A_{W2} = \frac{a^A_{W0} W^A \bar{C}^0_{R1}}{d^A_W + \kappa^A_W} = \frac{W^A \bar{C}^0_{R1}}{K^A_{W0}} = \frac{W^A R^n}{K^A_{W0} K_R \bar{K}_R} D,$$

$$M^A = \frac{a^A_M W^A D^A_{tot}}{d^A_M} = \frac{W^A D^A_{tot}}{K^A_{MM}}, \;\; C^A_M = \frac{\bar{a}^A M^A D}{\bar{d}^A + \kappa^A_M} = \frac{W^A D^A_{tot}}{\bar{K}^A_M K^A_{MM}} D = \frac{W^A D^A_{tot}}{K^A_M} D,$$

$$C^A_{M1} = \frac{\bar{a}^A M^A \bar{C}_A}{\bar{d}^A + \kappa^A_M} = \frac{W^A D^A_{tot}}{\bar{K}^A_M K^A_{MM}} \bar{C}_A = \frac{A^n W^A D^A_{tot}}{K_A \bar{K}_A K^A_M} D,$$

$$C^A_{M2} = \frac{\bar{a}^A M^A \bar{C}^0_{R1}}{\bar{d}^A + \kappa^A_M} = \frac{W^A D^A_{tot}}{\bar{K}^A_M K^A_{MM}} \bar{C}^0_{R1} = \frac{R^n W^A D^A_{tot}}{K_R \bar{K}_R K^A_M} D,$$

$$E^A_{act} = \frac{a^A_e (D^R_2 + D^R_{12h} + D^R_{12tot}) E^A}{d^A_e} = \frac{(D^R_2 + D^R_{12h} + D^R_{12tot}) E^A}{K^A_{EE}},$$

$$E^{Ad}_{act} = \frac{a^A_e (D^R_{1tot} + D^R_{12tot}) E^A}{d^A_e} = \frac{(D^R_{1tot} + D^R_{12tot}) E^A}{K^A_{EE}}, \;\; C^A_{E_{tot}} = \frac{a^A_E E^A D^A_{tot}}{d^A_E + \kappa^A_E} = \frac{E^A}{K^A_E} D^A_{tot},$$

$$C^A_{E_{act}tot} = \frac{E^A_{act}}{K^A_E} D^A_{tot} = \frac{E^A(D^R_2 + D^R_{12h} + D^R_{12tot})}{K^A_{EE} K^A_E} D^A_{tot} = \frac{E^A(D^R_2 + D^R_{12h} + D^R_{12tot})}{K^{A*}_E} D^A_{tot},$$

$$C^{Ad}_{E_{tot}} = \frac{E^{Ad}_{act}}{K^A_E} D^A_{tot} = \frac{E^A(D^R_{1tot} + D^R_{12tot})}{K^A_{EE} K^A_E} D^A_{tot} = \frac{E^A(D^R_{1tot} + D^R_{12tot})}{K^{A*}_E} D^A_{tot},$$

Substituting the QSS values in the ODEs and defining $C^0_{tot} = C^0_1 + C^0_{12}$ and $D^R_{htot} = D^R_{1h} + D^R_{12h}$, model (70) becomes

$$\begin{aligned}
\dot{D}^R_{1tot} &= (\kappa^d_W \frac{W^d}{K^d_{W0}} \left(1 + \frac{A^n}{K_A \bar{K}_A}\right) + \kappa^d_W (\frac{1}{K^d_{W0}} + \frac{1}{K^d_W}) \frac{W^d R^n}{K_R \bar{K}_R} \\
&+ \kappa^{'}_M \frac{W^d}{K^{'}_M} \left(1 + \frac{A^n}{K_A \bar{K}_A} + \frac{R^n}{K_R \bar{K}_R}\right) (D^R_2 + D^R_{12h} + D^R_{12tot}))D - \delta^{'} D^R_{1tot} - (\kappa^d_T \frac{T}{K^d_T} + \kappa^d_T \frac{T D^A_{tot}}{K^*_T}) D^R_1 \\
&- (\kappa^2_W \frac{W^R}{K^R_{W0}} + \kappa_M \frac{W^R}{K^R_M} (D^R_2 + D^R_{12h} + D^R_{12tot}) + \bar{\kappa}_M \frac{W^R}{\bar{K}_M} (C^0_1 + C^0_{12})) D^R_{1tot} \\
&+ (\delta + \kappa^R_E \frac{E^R}{K^R_E} + \kappa^R_E \frac{E^R D^A_{tot}}{K^{R*}_E}) D^R_{12tot} \\
\dot{D}^R_{1h} &= (\kappa^d_T \frac{T}{K^d_T} + \kappa^d_T \frac{T D^A_{tot}}{K^*_T}) D^R_1 - \delta D^R_{1h} \\
&- (\kappa^2_W \frac{W^R}{K^R_{W0}} + \kappa_M \frac{W^R}{K^R_M} (D^R_2 + D^R_{12h} + D^R_{12tot}) + \bar{\kappa}_M \frac{W^R}{\bar{K}_M} (C^0_1 + C^0_{12})) D^R_{1h} \\
&+ (\delta + \kappa^R_E \frac{E^R}{K^R_E} + \kappa^R_E \frac{E^R D^A_{tot}}{K^{R*}_E}) D^R_{12h}
\end{aligned}$$

$$
\begin{aligned}
\dot{D}_2^R &= (\kappa_W^2 \frac{W^R}{K_{W0}^R}\left(1+\frac{A^n}{K_A\bar{K}_A}\right) + \kappa_W^2(\frac{1}{K_{W0}^R}+\frac{1}{K_W^R})\frac{W^R R^n}{K_R\bar{K}_R} \\
&+ \kappa_M \frac{W^R}{K_M^R}\left(1+\frac{A^n}{K_A\bar{K}_A}+\frac{R^n}{K_R\bar{K}_R}\right)(D_2^R + D_{12h}^R + D_{12tot}^R) \\
&+ \bar{\kappa}_M \frac{W^R}{\bar{K}_M}\left(1+\frac{A^n}{K_A\bar{K}_A}+\frac{R^n}{K_R\bar{K}_R}\right)(C_1^0 + C_{12}^0))D - (\delta + \kappa_E^R \frac{E^R}{K_E^R} + \kappa_E^R \frac{E^R D_{tot}^A}{K_E^{R*}})D_2^R \\
&- [(\kappa_W^d \frac{W^d}{K_{W0}^d} + \kappa_M^{'} \frac{W^d}{K_M^{'}}(D_2^R + D_{12h}^R + D_{12tot}^R))D_2^R - \delta^{'} D_{12tot}^R - \delta D_{12h}^R] \\
\dot{D}_{12tot}^R &= (\kappa_W^2 \frac{W^R}{K_{W0}^R} + \kappa_M \frac{W^R}{K_M^R}(D_2^R + D_{12h}^R + D_{12tot}^R) + \bar{\kappa}_M \frac{W^R}{\bar{K}_M}(C_1^0 + C_{12}^0))D_{1tot}^R \\
&- (\delta + \kappa_E^R \frac{E^R}{K_E^R} + \kappa_E^R \frac{E^R D_{tot}^A}{K_E^{R*}})D_{12tot}^R + (\kappa_W^d \frac{W^d}{K_{W0}^d} + \kappa_M^{'} \frac{W^d}{K_M^{'}}(D_2^R + D_{12h}^R + D_{12}^R))D_2^R \\
&- \delta^{'} D_{12tot}^R - (\kappa_T^d \frac{T}{K_T^d} + \kappa_T^d \frac{T D_{tot}^A}{K_T^*})D_{12}^R \\
\dot{D}_{12h}^R &= (\kappa_T^d \frac{T}{K_T^d} + \kappa_T^d \frac{T D_{tot}^A}{K_T^*})D_{12}^R - \delta D_{12h}^R \\
&+ [(\kappa_W^2 \frac{W^R}{K_{W0}^R} + \kappa_M \frac{W^R}{K_M^R}(D_2^R + D_{12h}^R + D_{12tot}^R) + \bar{\kappa}_M \frac{W^R}{\bar{K}_M}(C_1^0 + C_{12}^0))D_{1h}^R \\
&- (\delta + \kappa_E^R \frac{E^R}{K_E^R} + \kappa_E^R \frac{E^R D_{tot}^A}{K_E^{R*}})D_{12h}^R] \\
\dot{C}_{tot}^0 &= \kappa_B^d \frac{B}{K_B^d}(D_1^R + D_{12}^R) - dC_{tot}^0 - \delta^{'} C_{tot}^0 \\
\dot{D}_{htot}^R &= (\kappa_T^d \frac{T}{K_T^d} + \kappa_T^d \frac{T D_{tot}^A}{K_T^*})(D_1^R + D_{12}^R) - \delta D_{htot}^R \\
\dot{D}_{tot}^A &= (\kappa_W^A \frac{W^A}{K_{W0}^A}\left(1+\frac{R^n}{K_R\bar{K}_R}\right) + \kappa_W^A(\frac{1}{K_{W0}^A}+\frac{1}{K_W^A})\frac{W^A A^n}{K_A\bar{K}_A} + \kappa_M^A \frac{W^A}{K_M^A}\left(1+\frac{A^n}{K_A\bar{K}_A}+\frac{R^n}{K_R\bar{K}_R}\right)D_{tot}^A)D \\
&- (\delta + \kappa_E^A \frac{E^A}{K_E^A} + \kappa_E^A \frac{E^A(D_2^R + D_{12h}^R + D_{12tot}^R)}{K_E^{A*}} + \kappa_E^A \frac{E^A(D_{1tot}^R + D_{12tot}^R)}{K_E^{A*}})D_{tot}^A,
\end{aligned}
\tag{71}
$$

in which $D_{1tot}^R = D_1^R + C_1^0$ and $D_{12tot}^R = D_{12}^R + C_{12}^0$, as defined at the beginning of the section. Furthermore, we make a variable substitution, by introducing $Y_1 = D_{1tot}^R + D_{12tot}^R$ = "total concentration of nucleosomes characterized by methylated DNA", $Y_2 = D_2^R + D_{12h}^R + D_{12tot}^R$ = "total concentration of nucleosomes characterized by H3K9me3" and $Y_{tot} = D_{1tot}^R + D_2^R + D_{12h}^R + D_{12tot}^R$ = "total concentration of nucleosomes characterized by at least one between methyalated DNA and H3K9me3". The model can be rewritten as following:

$$
\begin{aligned}
\dot{Y}_1 &= (\kappa_W^d \frac{W^d}{K_W^d} \frac{\frac{R^n}{K_R\bar{K}_R}}{1+\frac{A^n}{K_A\bar{K}_A}+\frac{R^n}{K_R\bar{K}_R}}) \left(\left(1+\frac{A^n}{K_A\bar{K}_A}+\frac{R^n}{K_R\bar{K}_R} \right) D \right) \\
&+ (\kappa_W^d \frac{W^d}{K_{W0}^d} + \kappa_M^{'} \frac{W^d}{K_M^{'}}(Y_2)) \left(\left(1+\frac{A^n}{K_A\bar{K}_A}+\frac{R^n}{K_R\bar{K}_R} \right) D + D_2^R \right) \\
&- \delta^{'} Y_1 - \kappa_T^d (\frac{T}{K_T^d} + \frac{TD_{tot}^A}{K_T^*})(D_1^R + D_{12}^R) \\
\dot{Y}_2 &= (\kappa_W^2 \frac{W^R}{K_W^R} \frac{\frac{R^n}{K_R\bar{K}_R}}{1+\frac{A^n}{K_A\bar{K}_A}+\frac{R^n}{K_R\bar{K}_R}}) \left(\left(1+\frac{A^n}{K_A\bar{K}_A}+\frac{R^n}{K_R\bar{K}_R} \right) D \right) \\
&+ (\kappa_W^2 \frac{W^R}{K_{W0}^R} + \kappa_M \frac{W^R}{K_M^R}(Y_2) + \bar{\kappa}_M \frac{W^R}{\bar{K}_M}(C_{tot}^0)) \left(\left(1+\frac{A^n}{K_A\bar{K}_A}+\frac{R^n}{K_R\bar{K}_R} \right) D + D_{1tot}^R + D_{1h}^R \right) \\
&- (\delta + \kappa_E^R (\frac{E^R}{K_E^R} + \frac{E^R D_{tot}^A}{K_E^{R*}}))Y_2 \\
\dot{Y}_{tot} &= (\kappa_W^d \frac{W^d}{K_{W0}^d} + \kappa_W^d \frac{W^d}{K_W^d} \frac{\frac{R^n}{K_R\bar{K}_R}}{1+\frac{A^n}{K_A\bar{K}_A}+\frac{R^n}{K_R\bar{K}_R}} + \kappa_M^{'} \frac{W^d}{K_M^{'}}(Y_2) + \kappa_W^2 \frac{W^R}{K_{W0}^R} \\
&+ \kappa_W^2 \frac{W^R}{K_W^R} \frac{\frac{R^n}{K_R\bar{K}_R}}{1+\frac{A^n}{K_A\bar{K}_A}+\frac{R^n}{K_R\bar{K}_R}} + \kappa_M \frac{W^R}{K_M^R}(Y_2) + \bar{\kappa}_M \frac{W^R}{\bar{K}_M}(C_{tot}^0)) \left(\left(1+\frac{A^n}{K_A\bar{K}_A}+\frac{R^n}{K_R\bar{K}_R} \right) D \right) \\
&- \delta^{'}(Y_{tot} - Y_2) - \kappa_T^d (\frac{T}{K_T^d} + \frac{TD_{tot}^A}{K_T^*})(D_1^R + D_{12}^R) - (\delta + \kappa_E^R (\frac{E^R}{K_E^R} + \frac{E^R D_{tot}^A}{K_E^{R*}}))(Y_{tot} - Y_1) \\
\dot{C}_{tot}^0 &= \kappa_B^d \frac{B}{K_B^d}(D_1^R + D_{12}^R) - dC_{tot}^0 - \delta^{'} C_{tot}^0 \\
\dot{D}_{1h}^R &= \kappa_T^d (\frac{T}{K_T^d} + \frac{TD_{tot}^A}{K_T^*})D_1^R - \delta D_{1h}^R \\
&- [(\kappa_W^2 \frac{W^R}{K_{W0}^R} + \kappa_M \frac{W^R}{K_M^R}(Y_2) + \bar{\kappa}_M \frac{W^R}{\bar{K}_M}(C_{tot}^0))D_{1h}^R - (\delta + \kappa_E^R (\frac{E^R}{K_E^R} + \frac{E^R D_{tot}^A}{K_E^{R*}}))D_{12h}^R] \\
\dot{D}_{12h}^R &= \kappa_T^d (\frac{T}{K_T^d} + \frac{TD_{tot}^A}{K_T^*})D_{12}^R - \delta D_{12h}^R \\
&+ [(\kappa_W^2 \frac{W^R}{K_{W0}^R} + \kappa_M \frac{W^R}{K_M^R}(Y_2) + \bar{\kappa}_M \frac{W^R}{\bar{K}_M}(C_{tot}^0))D_{1h}^R - (\delta + \kappa_E^R (\frac{E^R}{K_E^R} + \frac{E^R D_{tot}^A}{K_E^{R*}}))D_{12h}^R] \\
\dot{D}_{htot}^R &= \kappa_T^d (\frac{T}{K_T^d} + \frac{TD_{tot}^A}{K_T^*})(D_1^R + D_{12}^R) - \delta D_{htot}^R \\
\dot{D}_{tot}^A &= (\kappa_W^A \frac{W^A}{K_{W0}^A} + \kappa_W^A \frac{W^A}{K_W^A} \frac{\frac{A^n}{K_A\bar{K}_A}}{1+\frac{A^n}{K_A\bar{K}_A}+\frac{R^n}{K_R\bar{K}_R}} + \kappa_M^A \frac{W^A}{K_M^A} D_{tot}^A) \left((1+\frac{A^n}{K_A\bar{K}_A}+\frac{R^n}{K_R\bar{K}_R})D \right) \\
&- (\delta + \kappa_E^A (\frac{E^A}{K_E^A} + \frac{E^A Y_2}{K_E^{A*}} + \frac{E^A Y_1}{K_E^{A*}}))D_{tot}^A.
\end{aligned}
\tag{72}
$$

Assuming $d \gg \delta$, we can set C_{tot}^0 at its QSS (that is $C_1^0 = \frac{\kappa_B^d}{d+\delta'} \frac{B}{K_B^d}(D_1^R + D_{12}^R)$) and so, since $Y_1 = D_{1tot}^R + D_{12tot}^R = D_1^R + D_{12}^R + C_{tot}^0$, we can express express $(D_1^R + D_{12}^R)$ as function of Y_1 and

C^0_{tot}. Then, defining $k_B = \kappa^d_B \frac{B}{K^d_B}$, $\bar{\delta} = d + \delta'$ and $\bar{\kappa}_{M1} = \bar{\kappa}_M \frac{k_B}{k_B + \bar{\delta}}$, equations (72) become

$$\begin{aligned}
\dot{Y}_1 &= (\kappa^d_W \frac{W^d}{K^d_W} \frac{\frac{R^n}{K_R \bar{K}_R}}{1 + \frac{A^n}{K_A \bar{K}_A} + \frac{R^n}{K_R \bar{K}_R}}) \left(\left(1 + \frac{A^n}{K_A \bar{K}_A} + \frac{R^n}{K_R \bar{K}_R} \right) D \right) \\
&+ (\kappa^d_W \frac{W^d}{K^d_{W0}} + \kappa^{'}_M \frac{W^d}{K^{'}_M}(Y_2)) \left(\left(1 + \frac{A^n}{K_A \bar{K}_A} + \frac{R^n}{K_R \bar{K}_R} \right) D + D^R_2 \right) \\
&- (\delta^{'} + \kappa^d_T \frac{\bar{\delta}}{k_B + \bar{\delta}} (\frac{T}{K^d_T} + \frac{TD^A_{tot}}{K^*_T})) Y_1 \\
&= k^1_W \left(\left(1 + \frac{A^n}{K_A \bar{K}_A} + \frac{R^n}{K_R \bar{K}_R} \right) D \right) + (k^1_{W0} + k^{'}_M)(Y_2) \left(\left(1 + \frac{A^n}{K_A \bar{K}_A} + \frac{R^n}{K_R \bar{K}_R} \right) D + D^R_2 \right) \\
&- (\delta^{'} + \kappa^d_T \frac{\bar{\delta}}{k_B + \bar{\delta}} (\frac{T}{K^d_T} + \frac{TD^A_{tot}}{K^*_T})) Y_1 \\
\dot{Y}_2 &= (\kappa^2_W \frac{W^R}{K^R_W} \frac{\frac{R^n}{K_R \bar{K}_R}}{1 + \frac{A^n}{K_A \bar{K}_A} + \frac{R^n}{K_R \bar{K}_R}}) \left(\left(1 + \frac{A^n}{K_A \bar{K}_A} + \frac{R^n}{K_R \bar{K}_R} \right) D \right) \\
&+ (\kappa^2_W \frac{W^R}{K^R_{W0}} + \kappa_M \frac{W^R}{K^R_M}(Y_2) + \bar{\kappa}_{M1} \frac{W^R}{\bar{K}_M}(Y_1)) \left(\left(1 + \frac{A^n}{K_A \bar{K}_A} + \frac{R^n}{K_R \bar{K}_R} \right) D + D^R_{1tot} + D^R_{1h} \right) \\
&- (\delta + \kappa^R_E (\frac{E^R}{K^R_E} + \frac{E^R D^A_{tot}}{K^{R*}_E})) Y_2 \\
&= k^2_W \left(\left(1 + \frac{A^n}{K_A \bar{K}_A} + \frac{R^n}{K_R \bar{K}_R} \right) D \right) \\
&+ (k^2_{W0} + k_M(Y_2) + \bar{k}_M(Y_1)) \left(\left(1 + \frac{A^n}{K_A \bar{K}_A} + \frac{R^n}{K_R \bar{K}_R} \right) D + D^R_{1tot} + D^R_{1h} \right) \\
&- (\delta + \kappa^R_E (\frac{E^R}{K^R_E} + \frac{E^R D^A_{tot}}{K^{R*}_E})) Y_2 \\
\dot{Y}_{tot} &= (\kappa^d_W \frac{W^d}{K^d_{W0}} + \kappa^d_W \frac{W^d}{K^d_W} \frac{\frac{R^n}{K_R \bar{K}_R}}{1 + \frac{A^n}{K_A \bar{K}_A} + \frac{R^n}{K_R \bar{K}_R}} + \kappa^{'}_M \frac{W^d}{K^{'}_M}(Y_2) + \kappa^2_W \frac{W^R}{K^R_{W0}} \\
&+ \kappa^2_W \frac{W^R}{K^R_W} \frac{\frac{R^n}{K_R \bar{K}_R}}{1 + \frac{A^n}{K_A \bar{K}_A} + \frac{R^n}{K_R \bar{K}_R}} + \kappa_M \frac{W^R}{K^R_M}(Y_2) + \bar{\kappa}_{M1} \frac{W^R}{\bar{K}_M}(Y_1)) \left(\left(1 + \frac{A^n}{K_A \bar{K}_A} + \frac{R^n}{K_R \bar{K}_R} \right) D \right) \\
&- (\delta^{'} + \kappa^d_T \frac{\bar{\delta}}{k_B + \bar{\delta}} (\frac{T}{K^d_T} + \frac{TD^A_{tot}}{K^*_T}))(Y_{tot} - Y_2) - (\delta + \kappa^R_E (\frac{E^R}{K^R_E} + \frac{E^R D^A_{tot}}{K^{R*}_E}))(Y_{tot} - Y_1) \\
&= (k^1_{W0} + k^1_W + k^{'}_M(Y_2) + k^2_{W0} + k^2_W + k_M(Y_2) + \bar{k}_M(Y_1)) \left(\left(1 + \frac{A^n}{K_A \bar{K}_A} + \frac{R^n}{K_R \bar{K}_R} \right) D \right) \\
&- (\delta^{'} + \kappa^d_T \frac{\bar{\delta}}{k_B + \bar{\delta}} (\frac{T}{K^d_T} + \frac{TD^A_{tot}}{K^*_T}))(Y_{tot} - Y_2) - (\delta + \kappa^R_E (\frac{E^R}{K^R_E} + \frac{E^R D^A_{tot}}{K^{R*}_E}))(Y_{tot} - Y_1) \\
\dot{D}^R_{1h} &= \kappa^d_T (\frac{T}{K^d_T} + \frac{TD^A_{tot}}{K^*_T}) D^R_1 - \delta D^R_{1h} \\
&- [(\kappa^2_W \frac{W^R}{K^R_{W0}} + \kappa_M \frac{W^R}{K^R_M}(Y_2) + \bar{\kappa}_{M1} \frac{W^R}{\bar{K}_M}(Y_1)) D^R_{1h} - (\delta + \kappa^R_E (\frac{E^R}{K^R_E} + \frac{E^R D^A_{tot}}{K^{R*}_E})) D^R_{12h}] \\
&= \kappa^d_T (\frac{T}{K^d_T} + \frac{TD^A_{tot}}{K^*_T}) D^R_1 - \delta D^R_{1h} - [(k^2_{W0} + k_M(Y_2) + \bar{k}_M(Y_1)) D^R_{1h} - (\delta + \kappa^R_E (\frac{E^R}{K^R_E} + \frac{E^R D^A_{tot}}{K^{R*}_E})) D^R_{12h}]
\end{aligned} \quad (73)$$

$$
\begin{aligned}
\dot{D}^R_{12h} &= \kappa^d_T(\frac{T}{K^d_T} + \frac{TD^A_{tot}}{K^*_T})D^R_{12} - \delta D^R_{12h} \\
&+ [(\kappa^2_W \frac{W^R}{K^R_{W0}} + \kappa_M \frac{W^R}{K^R_M}(Y_2) + \bar{\kappa}_{M1}\frac{W^R}{\bar{K}_M}(Y_1))D^R_{1h} - (\delta + \kappa^R_E(\frac{E^R}{K^R_E} + \frac{E^R D^A_{tot}}{K^{R*}_E}))D^R_{12h}] \\
&= \kappa^d_T(\frac{T}{K^d_T} + \frac{TD^A_{tot}}{K^*_T})D^R_{12} - \delta D^R_{12h} + [(k^2_{W0} + k_M(Y_2) + \bar{k}_M(Y_1))D^R_{1h} - (\delta + \kappa^R_E(\frac{E^R}{K^R_E} + \frac{E^R D^A_{tot}}{K^{R*}_E}))D^R_{12h}] \\
\dot{D}^R_{htot} &= \kappa^d_T \frac{\bar{\delta}}{k_B + \bar{\delta}}(\frac{T}{K^d_T} + \frac{TD^A_{tot}}{K^*_T})Y_1 - \delta D^R_{htot} \\
\dot{D}^A_{tot} &= (\kappa^A_W \frac{W^A}{K^A_{W0}} + \kappa^A_W \frac{W^A}{K^A_W}\frac{\frac{A^n}{K_A\bar{K}_A}}{1 + \frac{A^n}{K_A\bar{K}_A} + \frac{R^n}{K_R\bar{K}_R}} + \kappa^A_M \frac{W^A}{K^A_M}D^A_{tot})\left((1 + \frac{A^n}{K_A\bar{K}_A} + \frac{R^n}{K_R\bar{K}_R})D\right) \\
&- (\delta + \kappa^A_E(\frac{E^A}{K^A_E} + \frac{E^A Y_2}{K^{A*}_E} + \frac{E^A Y_1}{K^{A*}_E}))D^A_{tot} \\
&= (k^A_{W0} + k^A_W + k^A_M D^A_{tot})\left((1 + \frac{A^n}{K_A\bar{K}_A} + \frac{R^n}{K_R\bar{K}_R})D\right)(\delta + \kappa^A_E(\frac{E^A}{K^A_E} + \frac{E^A Y_2}{K^{A*}_E} + \frac{E^A Y_1}{K^{A*}_E}))D^A_{tot}.
\end{aligned}
$$

D can be obtained by the DNA conservation law

$$
\begin{aligned}
D_{tot} &= D + \bar{C}^0_{R1} + C^{d,2}_{W1} + C^d_{W10} + C^d_{W1} + C^d_{T1} + T_{act} + C^d_{T1_{act}} + C^d_{B1} + C^R_{W20} + C^{R,2}_W + C^R_{W2} + M^R_{tot} + C^R_{M2} \\
&+ C^R_{M2_c} + C^R_{M2_{ac}} + C^R_{E2} + E^R_{act} + C^R_{E2_{act}} + D^R_2 + C^d_{W120} + C^d_{T12} + C^d_{T12_{act}} + C^d_{B12} + D^R_{1tot} + C^R_{W120} \\
&+ C^R_{M12} + C^R_{E12} + C^R_{E12_{act}} + D^R_{1h} + C^R_{W12h0} + C^R_{M12h} + C^R_{E12h} + C^R_{E12h_{act}} + C^{R0}_{W120} + C^{R0}_{M12} + C^{R0}_{E12} \\
&+ C^{R0}_{E12_{act}} + M' + \bar{M} + C'_{R1} + C'_{R1_c} + C'_{R1_{ac}} + \bar{C}_{R2} + \bar{C}_{R2_c} + \bar{C}_{R2_{ac}} + C'_{R12} + \bar{C}_{R12} + \bar{C}^0_{R12} \\
&+ \bar{C}_{R12h} + D^R_{12tot} + D^R_{12h} + \bar{C}_A + C^A_W + C^A_{W0} + C^{A,2}_W + M^A + C^A_M + C^A_{M1} + C^A_{M2} + E^{Ad}_{act} + E^A_{act} \\
&+ C^A_{E_{tot}} + C^A_{E_{act}tot} + C^{Ad}_{E_{tot}} + D^A_{tot}.
\end{aligned} \tag{74}
$$

If we assume that the sum of the complexes C^d_{W10}, $C^{d,2}_{W1}$, C^d_{W1}, C^d_{T1}, T_{act}, $C^d_{T1_{act}}$, C^d_{B1}, C^R_{W20} $C^{R,2}_W$, C^R_{W2}, M^R_{tot}, C^R_{M2}, $C^R_{M2_c}$, $C^R_{M2_{ac}}$, C^R_{E2}, E^R_{act}, $C^R_{E2_{act}}$, C^d_{W120}, C^d_{T12}, $C^d_{T12_{act}}$, C^d_{B12}, C^R_{W120}, C^R_{M12}, C^R_{E12}, $C^R_{E12_{act}}$, C^R_{W12h0}, C^R_{M12h}, C^R_{E12h}, $C^R_{E12h_{act}}$, C^{R0}_{M12}, C^{R0}_{W120}, C^{R0}_{E12}, $C^{R0}_{E12_{act}}$, M', $\bar{M}$, C'_{R1}, C'_{R1_c}, $C'_{R1_{ac}}$, $\bar{C}_{R2}$, $\bar{C}_{R2_c}$, $\bar{C}_{R2_{ac}}$, C'_{R12}, $\bar{C}_{R12}$, $\bar{C}^0_{R12}$, $\bar{C}_{R12h}$, C^A_{W0}, $C^{A,2}_W$, C^A_W, M^A, C^A_M, C^A_{M1}, C^A_{M2}, E^{Ad}_{act}, E^A_{act}, $C^A_{E_{tot}}$, $C^A_{E_{act}tot}$ and $C^{Ad}_{E_{tot}}$ is negligible with respect to $D + \bar{C}^0_{R1} + \bar{C}_A + D^R_2 + D^R_{1tot} + D^R_{1h} + D^R_{12tot} + D^R_{12h} + D^A_{tot}$, D_{tot} can be approximated as

$$
\begin{aligned}
D_{tot} &\approx D + \bar{C}^0_{R1} + \bar{C}^A + D^R_2 + D^R_{1tot} + D^R_{1h} + D^R_{12tot} + D^R_{12h} \\
&= \left(1 + \frac{A^n}{K_A\bar{K}_A} + \frac{R^n}{K_R\bar{K}_R}\right)D + D^R_2 + D^R_{1tot} + D^R_{1h} + D^R_{12tot} + D^R_{12h}
\end{aligned} \tag{75}
$$

in which the last equality is obtained by considering the complexes dynamics to the QSS. Then, defining k'^*_T as

$$
k'^*_T = \kappa^d_T \frac{\bar{\delta}}{k_B + \bar{\delta}}\frac{T}{K^*_T}, \tag{76}
$$

equations (73) become

$$
\begin{aligned}
\dot{Y}_1 &= k^1_W(D_{tot} - (Y_{tot} + D^R_{1h}) - D^A_{tot}) + (k^1_{W0} + k'_M Y_2)(D_{tot} - (Y_1 + D^R_{htot}) - D^A_{tot}) \\
&- (\delta' + \kappa^d_T \frac{\bar{\delta}}{k_B + \bar{\delta}}(\frac{T}{K^d_T} + \frac{TD^A_{tot}}{K^*_T}))Y_1
\end{aligned}
$$

$$
\begin{aligned}
&= k_W^1(D_{tot} - Y_{tot} - D_{tot}^A) + (k_{W0}^1 + k_M^{'} Y_2)(D_{tot} - Y_1 - D_{tot}^A) - (\delta^{'} + k_T^{'} + k_T^{'*} D_{tot}^A) Y_1 \\
\dot{Y}_2 &= k_W^2(D_{tot} - (Y_{tot} + D_{1h}^R) - D_{tot}^A) + (k_{W0}^2 + k_M Y_2 + \bar{k}_M Y_1)(D_{tot} - Y_2 - D_{tot}^A) \\
&\quad - (\delta + \kappa_E^R(\frac{E^R}{K_E^R} + \frac{E^R D_{tot}^A}{K_E^{R*}})) Y_2 \\
&= k_W^2(D_{tot} - Y_{tot} - D_{tot}^A) + (k_{W0}^2 + k_M(Y_2) + \bar{k}_M(Y_1))(D_{tot} - Y_2 - D_{tot}^A) - (\delta + \bar{k}_E^R + k_E^R D_{tot}^A) Y_2 \\
\dot{Y}_{tot} &= (k_{W0}^1 + k_W^1 + k_M^{'} Y_2 + k_{W0}^2 + k_W^2 + k_M Y_2 + \bar{k}_M Y_1)(D_{tot} - (Y_{tot} + D_{1h}^R) - D_{tot}^A) \qquad (77) \\
&\quad - (\delta^{'} + \kappa_T^d \frac{\bar{\delta}}{k_B + \bar{\delta}}(\frac{T}{K_T^d} + \frac{T D_{tot}^A}{K_T^*}))(Y_{tot} - Y_2) - (\delta + \kappa_E^R(\frac{E^R}{K_E^R} + \frac{E^R D_{tot}^A}{K_E^{R*}}))(Y_{tot} - Y_1) \\
&= (k_{W0}^1 + k_W^1 + k_M^{'} Y_2 + k_{W0}^2 + k_W^2 + k_M Y_2 + \bar{k}_M(Y_1))(D_{tot} - Y_{tot} - D_{tot}^A) \\
&\quad - (\delta^{'} + k_T^{'} + k_T^{'*} D_{tot}^A)(Y_{tot} - Y_2) - (\delta + \bar{k}_E^R + k_E^R D_{tot}^A)(Y_{tot} - Y_1) \\
\dot{D}_{tot}^A &= (k_{W0}^A + k_W^A + k_M^A D_{tot}^A)(D_{tot} - (Y_{tot} + D_{1h}^R) - D_{tot}^A) - (\delta + \kappa_E^A(\frac{E^A}{K_E^A} + \frac{E^A Y_2}{K_E^{A*}} + \frac{E^A Y_1}{K_E^{A*}})) D_{tot}^A \\
&= (k_{W0}^A + k_W^A + k_M^A D_{tot}^A)(D_{tot} - Y_{tot} - D_{tot}^A) - (\delta + \bar{k}_E^A + k_E^A Y_2 + k_E^A Y_1) D_{tot}^A
\end{aligned}
$$

in which, in the first equation, we safely neglect D_{htot}^R because we assume that $k_T^{'} \ll \delta$, which ensures that D_{htot}^R is well approximated by its quasi-steady state $D_{htot}^R = (k_T^{'}/\delta) Y_1$ and hence it is also negligible compared to Y_1. Furthermore, since $D_{1h}^R < D_{htot}^R$ and $Y_1 < Y_{tot}$, we can also neglect D_{1h}^R with respect to Y_{tot} in all the ODEs. In conclusion, the ODE model is given by

$$
\begin{aligned}
\dot{Y}_1 &= k_W^1(D_{tot} - Y_{tot} - D_{tot}^A) + (k_{W0}^1 + k_M^{'}) Y_2 (D_{tot} - Y_1 - D_{tot}^A) - (\delta^{'} + k_T^{'} + k_T^{'*} D_{tot}^A) Y_1 \\
\dot{Y}_2 &= k_W^2(D_{tot} - Y_{tot} - D_{tot}^A) + (k_{W0}^2 + k_M Y_2 + \bar{k}_M Y_1)(D_{tot} - Y_2 - D_{tot}^A) - (\delta + \bar{k}_E^R + k_E^R D_{tot}^A) Y_2 \\
\dot{Y}_{tot} &= (k_{W0}^1 + k_W^1 + k_M^{'} Y_2 + k_{W0}^2 + k_W^2 + k_M Y_2 + \bar{k}_M Y_1)(D_{tot} - Y_{tot} - D_{tot}^A) \\
&\quad - (\delta^{'} + k_T^{'} + k_T^{'*} D_{tot}^A)(Y_{tot} - Y_2) - (\delta + \bar{k}_E^R + k_E^R D_{tot}^A)(Y_{tot} - Y_1) \\
\dot{D}_{tot}^A &= (k_{W0}^A + k_W^A + k_M^A D_{tot}^A)(D_{tot} - Y_{tot} - D_{tot}^A) - (\delta + \bar{k}_E^A + k_E^A Y_2 + k_E^A Y_1) D_{tot}^A
\end{aligned}
\qquad (78)
$$

or, expressed in D_{1tot}^R, D_{12tot}^R, $D_{2tot}^R = D_2^R + D_{12h}^R$ and D_{tot}^A variables,

$$
\begin{aligned}
\dot{D}_1^R &= (k_{W0}^1 + k_W^1 + k_M^{'}(D_2^R + D_{12}^R)) D + (\delta + \bar{k}_E^R + k_E^R D^A) D_{12}^R \\
&\quad - (k_{W0}^2 + k_M(D_2^R + D_{12}^R) + \bar{k}_M(D_1^R + D_{12}^R) + \delta^{'} + k_T^{'} + k_T^{'*} D^A) D_1^R \\
\dot{D}_2^R &= (k_{W0}^2 + k_W^2 + k_M(D_2^R + D_{12}^R) + \bar{k}_M(D_1^R + D_{12}^R)) D + (\delta^{'} + k_T^{'} + k_T^{'*} D^A) D_{12}^R \\
&\quad - (k_{W0}^1 + k_M^{'}(D_2^R + D_{12}^R) + \delta + \bar{k}_E^R + k_E^R D^A) D_2^R \\
\dot{D}_{12}^R &= (k_{W0}^1 + k_M^{'}(D_2^R + D_{12}^R)) D_2^R + (k_{W0}^2 + k_M(D_2^R + D_{12}^R) + \bar{k}_M(D_1^R + D_{12}^R)) D_1^R \\
&\quad - (\delta^{'} + k_T^{'} + k_T^{'*} D^A + \delta + \bar{k}_E^R + k_E^R D^A) D_{12}^R \\
\dot{D}^A &= (k_{W0}^A + k_W^A + k_M^A D^A) D - (\delta + \bar{k}_E^A + k_E^A(D_2^R + D_{12}^R) + k_E^A(D_1^R + D_{12}^R)) D^A
\end{aligned}
\qquad (79)
$$

in which, with abuse of notation, we indicate D_{1tot}^R, D_{2tot}^R, D_{12tot}^R and D_{tot}^A with D_1^R, D_2^R, D_{12}^R and D^A, respectively, and $D = D_{tot} - D_1^R - D_2^R - D_{12}^R - D^A$.

Now, let us define $\bar{D}^A = D^A/D_{tot} = n^A/\mathrm{D_{tot}}$, $\bar{D}_1^R = D_1^R/D_{tot} = n_1^R/\mathrm{D_{tot}}$, $\bar{D}_2^R = D_2^R/D_{tot} = n_2^R/\mathrm{D_{tot}}$, $\bar{D}_{12}^R = D_{12}^R/D_{tot} = n_{12}^R/\mathrm{D_{tot}}$, $\bar{D} = D/D_{tot} = n^D/\mathrm{D_{tot}}$, the normalized time $\tau = t k_M^A D_{tot}$, the normalized inputs $\bar{u}^A = u_0^A + u^A$ with $u_0^A = k_{W0}^A/(k_M^A D_{tot})$, $u^A = k_W^A/(k_M^A D_{tot})$, $\bar{u}_1^R = u_{10}^R + u_1^R$ with $u_{10}^R = k_{W0}^1/k_M^A D_{tot}$ and $u_1^R = k_W^1/(k_M^A D_{tot})$, $\bar{u}_2^R = u_{20}^R + u_2^R$ with $u_{20}^R = k_{W0}^2/(k_M^A D_{tot})$ and $u_2^R = k_W^2/(k_M^A D_{tot})$, and the non-dimensional parameters $\epsilon = (\delta + \bar{k}_E^A)/(k_M^A D_{tot})$, $\mu = k_E^R/k_E^A$,

with a constant b such that $(\delta + \bar{k}_E^R)/(\delta + \bar{k}_E^A) = b\mu$, $\mu^{'} = (k_T^{'*})/(k_E^A)$, with a constant β such that $(\delta^{'} + k_T^{'})/(\delta + \bar{k}_E^A) = \beta\mu^{'}$, $\alpha = k_M/k_M^A$, $\bar{\alpha} = \bar{k}_M/k_M^A$, $\alpha^{'} = k_M^{'}/k_M^A$, and $\epsilon^{'} = k_E^A/k_M^A$. With these definitions and letting $\dot{x} := dx/d\tau$, we can rewrite the system model in terms of non-dimensional variables and non-dimensional parameters as follows:

$$\begin{aligned}
\dot{\bar{D}}_1^R &= (\bar{u}_1^R + \alpha^{'}(\bar{D}_2^R + \bar{D}_{12}^R))(1 - \bar{D}_1^R - \bar{D}_2^R - \bar{D}_{12}^R - \bar{D}^A) + \mu(b\epsilon + \epsilon^{'}\bar{D}^A)\bar{D}_{12}^R \\
&\quad - (u_{20}^R + \alpha(\bar{D}_2^R + \bar{D}_{12}^R) + \bar{\alpha}(\bar{D}_1^R + \bar{D}_{12}^R) + \mu^{'}(\beta\epsilon + \epsilon^{'}\bar{D}^A))\bar{D}_1^R \\
\dot{\bar{D}}_2^R &= (\bar{u}_2^R + \alpha(\bar{D}_2^R + \bar{D}_{12}^R) + \bar{\alpha}(\bar{D}_1^R + \bar{D}_{12}^R))(1 - \bar{D}_1^R - \bar{D}_2^R - \bar{D}_{12}^R - \bar{D}^A) + \mu^{'}(\beta\epsilon + \epsilon^{'}\bar{D}^A)\bar{D}_{12}^R \\
&\quad - (u_{10}^R + \alpha^{'}(\bar{D}_2^R + \bar{D}_{12}^R) + \mu(b\epsilon + \epsilon^{'}\bar{D}^A))\bar{D}_2^R \\
\dot{\bar{D}}_{12}^R &= (u_{10}^R + \alpha^{'}(\bar{D}_2^R + \bar{D}_{12}^R))\bar{D}_2^R + (u_{20}^R + \alpha(\bar{D}_2^R + \bar{D}_{12}^R) + \bar{\alpha}(\bar{D}_1^R + \bar{D}_{12}^R))\bar{D}_1^R \\
&\quad - (\mu'(\beta\epsilon + \epsilon^{'}\bar{D}^A) + \mu(b\epsilon + \epsilon^{'}\bar{D}^A))\bar{D}_{12}^R \\
\dot{\bar{D}}^A &= (u_0^A + u^A + \bar{D}^A)(1 - \bar{D}_1^R - \bar{D}_2^R - \bar{D}_{12}^R - \bar{D}^A) - (\epsilon + \epsilon^{'}(\bar{D}_2^R + \bar{D}_{12}^R) + \epsilon^{'}(\bar{D}_1^R + \bar{D}_{12}^R))\bar{D}^A.
\end{aligned} \tag{80}$$

1.7 Expressions of the k_W^A, k_W^1 and k_W^2

In the derivation of the model (79), we obtain the following expressions of k_W^A, k_W^1 and k_W^2:

$$\begin{aligned}
k_W^A &= \kappa_W^A \frac{W^A}{K_W^A} \frac{\frac{A^n}{K_A\bar{K}_A}}{1 + \frac{A^n}{K_A\bar{K}_A} + \frac{R^n}{K_R\bar{K}_R}} = \kappa_W^A \frac{W^A}{K_W^A} \frac{\frac{A^n}{K_{AA}}}{1 + \frac{A^n}{K_{AA}} + \frac{R^n}{K_{RR}}}, \\
k_W^1 &= \kappa_W^d \frac{W^d}{K_W^d} \frac{\frac{R^n}{K_R\bar{K}_R}}{1 + \frac{A^n}{K_A\bar{K}_A} + \frac{R^n}{K_R\bar{K}_R}} = \kappa_W^d \frac{W^d}{K_W^d} \frac{\frac{R^n}{K_{RR}}}{1 + \frac{A^n}{K_{AA}} + \frac{R^n}{K_{RR}}}, \\
k_W^2 &= \kappa_W^2 \frac{W^R}{K_W^R} \frac{\frac{R^n}{K_R\bar{K}_R}}{1 + \frac{A^n}{K_A\bar{K}_A} + \frac{R^n}{K_R\bar{K}_R}} = \kappa_W^2 \frac{W^R}{K_W^R} \frac{\frac{R^n}{K_{RR}}}{1 + \frac{A^n}{K_{AA}} + \frac{R^n}{K_{RR}}},
\end{aligned} \tag{81}$$

in which $A = \frac{n^{Act}}{\Omega}$, $R = \frac{n^{Rep}}{\Omega}$, with Ω the reaction volume and n^{Act} (n^{Rep}) the number of molecules of the activator A (the repressor R), and with $K_{AA} = K_A\bar{K}_A$ and $K_{RR} = K_R\bar{K}_R$. Then, assuming that A and R bind DNA with cooperativity 1, expressions (81) can be re-written as follows:

$$k_W^A = \frac{\tilde{k}_W^A}{\Omega} \frac{n^{Act}}{1 + \frac{n^{Act}}{K_{AA}\Omega} + \frac{n^{Rep}}{K_{RR}\Omega}}, \tag{82}$$

$$\begin{aligned}
k_W^1 &= \frac{\tilde{k}_W^1}{\Omega} \frac{n^{Rep}}{1 + \frac{n^{Act}}{K_{AA}\Omega} + \frac{n^{Rep}}{K_{RR}\Omega}}, \\
k_W^2 &= \frac{\tilde{k}_W^2}{\Omega} \frac{n^{Rep}}{1 + \frac{n^{Act}}{K_{AA}\Omega} + \frac{n^{Rep}}{K_{RR}\Omega}}.
\end{aligned} \tag{83}$$

with $\tilde{k}_W^A = \kappa_W^A \frac{W^A}{K_W^A K_{AA}}$, $\tilde{k}_W^1 = \kappa_W^d \frac{W^d}{K_W^d K_{RR}}$ and $\tilde{k}_W^2 = \kappa_W^2 \frac{W^R}{K_W^R K_{RR}}$.

Now let us define u^A, u_1^R and u_2^R as $u^A = k_W^A/(k_M^A D_{tot})$, $u_1^R = k_W^1/(k_M^A D_{tot})$ and $u_2^R = k_R^2/(k_M^A D_{tot})$, respectively. Then, if we assume $n^{Act}/\Omega \ll K_{AA}$ and $n^{Rep}/\Omega \ll K_{RR}$, u^A, u_1^R and u_2^R can be written as

$$u^A = \frac{\tilde{k}_W^A n^{Act}}{\Omega k_M^A D_{tot}} = \frac{\tilde{k}_W^A n^{Act}}{k_M^A \mathrm{D_{tot}}} = \frac{\tilde{k}_W^A}{k_M^A}\bar{A} = \tilde{u}^A\bar{A}, \tag{84}$$

$$u_1^R = \frac{\tilde{k}_W^1 n^{Rep}}{\Omega k_M^A D_{tot}} = \frac{\tilde{k}_W^1 n^{Rep}}{k_M^A \mathrm{D_{tot}}} = \frac{\tilde{k}_W^1}{k_M^A}\bar{R} = \tilde{u}_1^R \bar{R},$$
$$u_2^R = \frac{\tilde{k}_W^2 n^{Rep}}{\Omega k_M^A D_{tot}} = \frac{\tilde{k}_W^2 n^{Rep}}{k_M^A \mathrm{D_{tot}}} = \frac{\tilde{k}_W^2}{k_M^A}\bar{R} = \tilde{u}_2^R \bar{R}, \quad (85)$$

in which we define $\bar{A} := n^{Act}/\mathrm{D_{tot}}$, $\bar{R} := n^{Rep}/\mathrm{D_{tot}}$, $\tilde{u}^A = \tilde{k}_W^A/k_M^A$, $\tilde{u}_1^R = \tilde{k}_W^1/k_M^A$ and $\tilde{u}_2^R = \tilde{k}_W^2/k_M^A$.

1.8 Summary of the assumptions considered in the models derivation

- We lump together the two methylation states (me2 and me3) for simplicity because both of them are associated with gene repression. This will not affect the type of qualitative predictions that we seek to make in this paper.
- A nucleosome cannot be characterized by more than one modified histone simultaneously.
- We assume for simplicity that the DNA wrapped around each nucleosome can have only one modifiable CpG.
- We assume that the sequence-specific TF does not sequester D from the writer enzyme recruited during the *de novo* establishment phase [1](Chapter 6), [3, 4]. Similarly, we assumed that the sequence-specific TF does not sequester D from the writer enzyme recruited *via* the read-write mechanism.
- Activating histone modifications are anti-correlated with DNA methylation (see Section 1.5) and then we assume that a nucleosome characterized by an activating histone modification cannot acquire CpG methylation.
- Repressive histone modifications can co-exist correlated with DNA methylation (see Section 1.4) and then we assume that a nucleosome characterized by a repressive histone modification can acquire CpG methylation, and *viceversa.*
- The binding reactions are much faster than the other reactions and thus we set the intermediate complexes dynamics to the QSS [49].
- The rate constant k_E^A of the enzymatic reactions erasing H3K4me3/ac, is independent of which repressive chromatin modification is recruiting the erasers. This assumption is not affecting the qualitatively results related to the effect of the repressive marks on the erasure and then on the memory of the active state.
- The rate constant k_M of the enzymatic reactions writing H3K9me3 when recruited by H3K9me3 itself is independent of whether the recruiting nucleosome has both H3K9me3 and CpGme or only H3K9me3. This assumption is not affecting the qualitatively results related to the effect of the auto-catalysis of H3K9me3 on the memory of the repressive state.
- The rate constant $\bar{k}_M$ of the enzymatic reactions writing H3K9me3 when recruited by CpGme is independent of whether the recruiting nucleosome has both H3K9me3 and CpGme or only CpGme. This assumption is not affecting the qualitatively results related to the effect of the cooperative interactions between repressive marks on the epigenetic cell memory.
- The rate constant of the enzymatic reactions writing H3K9me3 on either D or $\mathrm{D_1^R}$ are equal and equal to k_M when recruited by H3K9me3 itself or equal to $\bar{k}_M$ when recruited by CpGme. If this assumption were not true, there would be some differences in the relative amount of $\mathrm{D_2^R}$ and $\mathrm{D_{12}^R}$, but the qualitatively results would not change.

- The rate constant $k_M^{'}$ of the enzymatic reactions writing CpGme when recruited by H3K9me3 is independent of whether the recruiting nucleosome has both H3K9me3 and CpGme or only H3K9me3. This assumption is not affecting the qualitatively results related to the effect of the cooperative interactions between repressive marks on the epigenetic cell memory.

- The rate constant of the enzymatic reactions writing CpGme on either D or on D_2^R are equal and equal to $k_M^{'}$. If this assumption were not true, there would be some differences in the relative amount of D_1^R and D_{12}^R, but the qualitatively results would not change.

- The rate constant of the enzymatic reactions erasing H3K9me3 on either D_2^R or D_{12}^R are equal and equal to k_E^R. If this assumption wer not true, there would be some differences in the relative amount of D_2^R and D_{12}^R, but the qualitatively results would not be different.

- The rate constant of the enzymatic reactions erasing CpGme on either D_1^R or D_{12}^R are equal and equal to $k_T^{'*}$. If this assumption wer not true, there would be some differences in the relative amount of D_1^R and D_{12}^R, but the qualitatively results would not be different.

- If a repressive modification is present on the nucleosome, TFs cannot bind and then the only *de novo* establishment that can occur is due to non-specific enzyme recruitment (Section 4).

- For the model of k_W^A, k_W^1 and k_W^2 we assume that activator A and repressor R bind DNA with cooperativity 1 and that activators and repressors interfere with each other by competing for promoter binding. The formulas obtained can be written as (82),(83). Different forms of k_W^A, k_W^1 and k_W^2 can be obtained without without major changes in the model as long as they are increasing with A and decreasing with R.

2 Detailed analysis of the histone modification circuit model (Fig 1C)

2.1 Deterministic analysis

For the deterministic analysis of (29) we consider no external inputs ($\bar{u}^A = u_0^A$ and $\bar{u}^R = u_0^R$ small) and we determine the number of stable non-zero steady states that the system admits as functions of the key parameters ϵ, $\epsilon^{'}$ and μ. This is one of the key features to analyze an ODE model witnessing epigenetic cell memory because a non-zero stable steady state for the unstimulated system indicates that the system is able to keep in memory this state theoretically for an indefinite time [49].

For this analysis, we can rewrite system (29) as follows:

$$\begin{aligned}\dot{\bar{D}}^A &= (u_0^A + \bar{D}^A)(1 - \bar{D}^A - \bar{D}^R) - (\epsilon + \epsilon' \bar{D}^R)\bar{D}^A \\ \dot{\bar{D}}^R &= (u_0^R + \alpha \bar{D}^R)(1 - \bar{D}^A - \bar{D}^R) - \mu(b\epsilon + \epsilon' \bar{D}^A)\bar{D}^R.\end{aligned} \tag{86}$$

in which $u_0^A, u_0^R \ll 1$. Since u_0^A and u_0^R are much smaller than 1, this can be viewed as a regular perturbation problem [50]. In particular, let $u_0^A = u_0^R = u_0$, $\nu = u_0$ and $x = (\bar{D}^A, \bar{D}^R)$, then the above system can be described as

$$\dot{x} = f(x, \nu). \tag{87}$$

On this system, we are interested in characterizing the steady states (the locally unique solutions to $f(x,\nu) = 0$) with $x \geq 0$. Since $\nu \ll 1$, we can determine the steady states of our system by studying the locally unique solutions to $f(x, 0) = 0$. In particular, let us define $\bar{x}_i \geq 0$ a value of x such that $f(\bar{x}_i, 0) = 0$. Then we can exploit the Implicit Function Theorem (IFT) [51] that allows

us to claim that if $\frac{\partial f(x,\nu)}{\partial x}|_{x=\bar{x}_i,\nu=0}$ is non-singular, then there is a local continuous function $\gamma_i(\nu)$ such that $\gamma_i(0) = \bar{x}_i$ and $f(\gamma_i(\nu),\nu) = 0$ in a neighborhood around $\nu = 0$. For our system (86), when we set $u_0 = 0$, we have four steady states:

$$\begin{aligned}
&\bar{x}_1 = (0,0),\\
&\bar{x}_2 = (0, 1 - \frac{\mu b\epsilon}{\alpha}),\\
&\bar{x}_3 = (1-\epsilon, 0),\\
&\bar{x}_4 = (\frac{\epsilon'(\alpha - \mu b\epsilon) + \epsilon(\alpha - \mu b)}{\alpha\epsilon' + \mu\epsilon'(1+\epsilon')}, \frac{\mu\epsilon'(1-\epsilon) + \epsilon(\mu b - \alpha)}{\alpha\epsilon' + \mu\epsilon'(1+\epsilon')}).
\end{aligned} \tag{88}$$

Now, in order to verify for which steady states the conditions of the IFT hold, we evaluate the Jacobian $\frac{\partial f(x,\nu)}{\partial x}$, with $\nu = u_0$ and $x = (\bar{D}^A, \bar{D}^R)$:

$$J = \begin{pmatrix} (1 - \bar{D}^A - \bar{D}^R) - (u_0 + \bar{D}^A) - (\epsilon + \epsilon'\bar{D}^R) & -(1+\epsilon')\bar{D}^A - u_0 \\ -(\alpha + \mu\epsilon')\bar{D}^R - u_0 & \alpha(1 - \bar{D}^A - \bar{D}^R) - (u_0 + \alpha\bar{D}^R) - \mu(b\epsilon + \epsilon'\bar{D}^A) \end{pmatrix}. \tag{89}$$

In particular for $\bar{x}_1$ the conditions of the IFT hold if $\epsilon \neq 1$ and $\epsilon \neq \frac{\alpha}{\mu b}$, for $\bar{x}_2$ the conditions of the IFT hold if $\epsilon \neq \frac{\alpha}{\mu b}$ and $\mu \neq \frac{\alpha(\epsilon' + \epsilon)}{b\epsilon(\epsilon'+1)} = \mu_2$, for $\bar{x}_3$ the conditions of the IFT hold if $\epsilon \neq 1$ and $\mu \neq \frac{\epsilon\alpha}{\epsilon'(1-\epsilon)+\epsilon b} = \mu_1$ and $\bar{x}_4$ the conditions of the IFT hold if $\mu \neq \mu_1$ and $\mu \neq \mu_2$. If the conditions hold for all the steady states, this means that system (86) with u_0 small also has four equilibria which are close to those in (88). In particular they can be approximated as [51]

$$\gamma_i = \bar{x}_i - \nu[\frac{\partial f(x,\nu)}{\partial x}|_{x=\bar{x}_i,\nu=0}]^{-1}\frac{\partial f(x,\nu)}{\partial \nu}|_{x=\bar{x}_i,\nu=0} = \bar{x}_i + \nu\bar{x}_i^1, \tag{90}$$

in which we define $\bar{x}_i^1 = -[\frac{\partial f(x,\nu)}{\partial x}|_{x=\bar{x}_i,\nu=0}]^{-1}\frac{\partial f(x,\nu)}{\partial \nu}|_{x=\bar{x}_i,\nu=0}$. Now, for the first three steady states γ_i with $i = 1,2,3$, we determine the sign of $\bar{x}_i$ and $\bar{x}_i^1$ to check if they are in the positive quadrant. In particular, we obtain that

$$\begin{aligned}
&\gamma_1 \geq 0 \; if \; \epsilon > max\{1, \frac{\alpha}{\mu b}\}\\
&\gamma_2 \geq 0 \; if \; \epsilon < \frac{\alpha}{\mu b}, \; \mu < \frac{\alpha(\epsilon' + \epsilon)}{b\epsilon(\epsilon' + 1)} = \mu_2\\
&\gamma_3 \geq 0 \; if \; \epsilon < 1, \; \mu > \frac{\epsilon\alpha}{\epsilon'(1-\epsilon) + \epsilon b} = \mu_1.
\end{aligned} \tag{91}$$

Concerning the fourth steady state, we have to check when $\bar{x}_4$ is in the positive and does not collide with one of the two steady states $\bar{x}_1$ and $\bar{x}_2$ (find the conditions such that $\bar{x}_4 \geq 0$, $\bar{x}_4 \neq \bar{x}_2$ and $\bar{x}_4 \neq \bar{x}_3$). In particular, this is verified when $\mu > \mu_1$, $\mu < \mu_2$.

Concerning the study of the stability of these equilibria, we exploit the fact that the eigenvalues of a square real or complex matrix depend continuously on its entries [52, 53]. Thanks to this property, it is possible to claim that, introducing the parameter ν defined over an open set $\Omega \subset \mathbb{R}$ and a matrix $A(\nu)$, if the entries of $A(\nu)$ are continuous, then the spectrum of the matrix, $\mathrm{sp}(A(\nu))$, is continuous. This means that, given $\nu_0 \in \Omega$ and given $\bar{\lambda} \in \mathrm{sp}(A(\nu_0))$ with multiplicity m as a root of the characteristic polynomial of $A(\nu_0)$, for any sufficiently small $r > 0$, there exists a neighborhood U of ν_0 in Ω such that, for all $\nu \in U$, the matrix $A(\nu)$ has m eigenvalues (counting multiplicities) in $B(\bar{\lambda}, r)$ [53]. Then, since the Jacobian (89) is continuous in x and ν and since we showed with the IFT that the steady states are continuous function of ν, we can claim that $\mathrm{sp}(J(\nu))$ is continuous.

This allows us to determine the stability conditions of $\bar{x}_i$ (conditions under which the eigenvalues of the Jacobian J with $u_0 = 0$ and $x = \bar{x}_i$ have negative real part) and then to extend, for a sufficiently small u_0, these conditions to the steady states of our original system.

In particular, if we set $x = \bar{x}_1$ in the Jacobian (89) with $u_0 = 0$, the matrix eigenvalues are $\bar{\lambda}_1 = 1 - \epsilon$ and $\bar{\lambda}_2 = \alpha - \mu b \epsilon$ and then this steady state is stable only if $\epsilon >\max\{1, \frac{\alpha}{\mu b}\}$.

If we set $x = \bar{x}_2$ in (89) with $u_0 = 0$, the Jacobian's eigenvalues are $\bar{\lambda}_1 = (1-\bar{D}^R_{ss})-(\epsilon+\epsilon'\bar{D}^R_{ss})$ and $\bar{\lambda}_2 = \alpha(1-\bar{D}^R_{ss})-\alpha\bar{D}^R_{ss}-\mu b\epsilon$, with $\bar{D}^R_{ss} = 1-(\mu b/\alpha)\epsilon$. Studying the sign of $\bar{\lambda}_2$, it is possible to notice that, if $(\bar{D}^R_{ss}, \bar{D}^A_{ss})_2$ exists (that is, if $\frac{\mu b}{\alpha}\epsilon < 1$), $\bar{\lambda}_2 = \mu b\epsilon - \alpha(1 - (\mu b/\alpha)\epsilon) + \mu b\epsilon = -\alpha(1 - (\mu b/\alpha)\epsilon)$ is always negative. Concerning $\bar{\lambda}_1$, it is negative if

$$\mu < \mu_2. \tag{92}$$

If we set $x = \bar{x}_3$ in the Jacobian (89) with $u_0 = 0$, the Jacobian's eigenvalues are $\bar{\lambda}_1 = (1 - \bar{D}^A_{ss}) - \bar{D}^A_{ss} - \epsilon$ and $\bar{\lambda}_2 = \alpha(1-\bar{D}^A_{ss}) - (\mu b\epsilon + \mu\epsilon'\bar{D}^A_{ss})$ with $\bar{D}^A_{ss} = 1-\epsilon$. Studying the sign of the eigenvalues, it is possible to notice that, if $(D^R_{ss}, D^A_{ss})_3$ exists (if $\epsilon < 1$), $\bar{\lambda}_1 = -(1-\epsilon)$ is always negative and $\bar{\lambda}_2$ is negative if

$$\mu > \mu_1, \tag{93}$$

with μ_1 defined as done in (91). Furthermore, by comparing the formulas of μ_1 and μ_2 defined as done in (91), it is possible to calculate that, if $\epsilon < 1$, μ_1 is always lower than μ_2. If we set $x = \bar{x}_4$ in (89) with $u_0 = 0$, it is possible to show that, when it is in the positive quadrant, it has never both eigenvalues with negative real part. To summarize, this analysis shows that, for a sufficiently small u_0,

- System (86) is characterized by a unique stable steady state $\gamma_1 = (\bar{D}^A_{ss}, \bar{D}^R_{ss})_1 \approx (0, 0)$ if $\epsilon > max\{1, \frac{\alpha}{\mu b}\}$;
- System (86) is characterized by a unique stable steady state $\gamma_2 = (\bar{D}^A_{ss}, \bar{D}^R_{ss})_2 \approx (0, 1 - \frac{\mu b\epsilon}{\alpha})$ if $1 < \epsilon < \frac{\alpha}{\mu b}$ and $\mu < \mu_2$ or $\epsilon < min\{1, \frac{\alpha}{\mu b}\}$ and $\mu < \mu_1$;
- System (86) is characterized by a unique stable steady state $\gamma_3 = (\bar{D}^A_{ss}, \bar{D}^R_{ss})_3 \approx (1 - \epsilon, 0)$ if $\frac{\alpha}{\mu b} < \epsilon < 1$ and $\mu > \mu_1$ or $\epsilon < min\{1, \frac{\alpha}{\mu b}\}$ and $\mu > \mu_2$;
- System (86) is characterized by two stable steady states $\gamma_2 \approx (0, 1 - \frac{\mu b\epsilon}{\alpha})$ and $\gamma_3 \approx (1 - \epsilon, 0)$ if $\epsilon < min\{1, \frac{\alpha}{\mu b}\}$ and $\mu_1 < \mu < \mu_2$.

This implies that, for having non-zero stable steady states, ϵ has to be sufficiently small and then, depending on the value of μ, we can have either one or two non-zero stable steady states. Furthermore, if $\epsilon \ll min\{1, \frac{\alpha}{\mu b}\}$, $(D^A_{ss}, D^R_{ss})_2 \approx (0, 1)$ and $(D^A_{ss}, D^R_{ss})_3 \approx (1, 0)$, that is, at these states, the gene is either almost fully modified with repressive marks or activating marks. The results of this deterministic analysis are shown in Fig D, in which we plot the system nullclines ($\dot{\bar{D}}^A = 0$ and $\dot{\bar{D}}^R = 0$) for all the qualitatively different parameter regimes.

2.2 Model reduction of the 2D model

In order to reduce the system, let us re-write model (28), writing explicitly the ODE for D:

$$\begin{aligned}
\dot{D}^A &= (k^A_{W0} + k^A_W + k^A_M D^A)D - (\delta + \bar{k}^A_E + k^A_E D^R)D^A \\
\dot{D}^R &= (k^R_{W0} + k^R_W + k^R_M D^R)D - (\delta + \bar{k}^R_E + k^R_E D^A)D^R \\
\dot{D} &= (\delta + \bar{k}^R_E + k^R_E D^A)D^R + (\delta + \bar{k}^A_E + k^A_E D^R)D^A - (k^A_{W0} + k^A_W + k^A_M D^A + k^R_{W0} + k^R_W + k^R_M D^R)D
\end{aligned} \tag{94}$$

with initial condition such that $D_{tot} = D + D^A + D^R$. Now, let us introduce the new time variable $\bar{\tau} = tD_{tot}k_E^A$ in the system written above, obtaining

$$\begin{aligned}
\epsilon' \frac{d\bar{D}^A}{d\bar{\tau}} &= (u_0^A + u^A + \bar{D}^A)\bar{D} - \epsilon'(c + \bar{D}^R)\bar{D}^A \\
\epsilon' \frac{d\bar{D}^R}{d\bar{\tau}} &= (u_0^R + u^R + \alpha\bar{D}^R)\bar{D} - \mu\epsilon'(cb + \bar{D}^A)\bar{D}^R \\
\epsilon' \frac{d\bar{D}}{d\bar{\tau}} &= \epsilon'[\mu(cb + \bar{D}^A)\bar{D}^R + (c + \bar{D}^R)\bar{D}^A] - (u_0^A + u^A + \bar{D}^A + u_0^R + u^R + \alpha\bar{D}^R)\bar{D}.
\end{aligned} \tag{95}$$

in which each species is normalized with respect to D_{tot} ($\bar{X} := X/D_{tot}$), in which we introduce $c = \frac{\epsilon}{\epsilon'}$ and all the other parameters are defined as done in (29). Based on the results of [54], it is possible to obtain a reduced version of this type of system if certain conditions are satisfied. In particular, given a general dynamical system $\frac{dx}{dt} = f(x,t)$ with $x \in \mathbb{R}^n$, let us define a smooth surface S in $\mathbb{R}^n \times \mathbb{R}$ as integral manifold of the system if any trajectory of the system that has at least one point in common with S lies entirely on S [55, 56]. Now, let us consider the system:

$$\begin{aligned}
\epsilon' \dot{x} &= f_1(x, y_2, t, \epsilon') \\
\epsilon' \dot{y}_2 &= f_2(x, y_2, t, \epsilon')
\end{aligned} \tag{96}$$

with $x \in \mathbb{R}^m$ and $y_2 \in \mathbb{R}^n$. If the matrix $A(x, y_2, t)$ given by

$$A(x, y_2, t) = \begin{pmatrix} \frac{\partial f_1}{\partial x} & \frac{\partial f_1}{\partial y_2} \\ \frac{\partial f_2}{\partial x} & \frac{\partial f_2}{\partial y_2} \end{pmatrix} = \begin{pmatrix} f_{1x} & f_{1y_2} \\ f_{2x} & f_{2y_2} \end{pmatrix} \tag{97}$$

with $\epsilon' = 0$ is singular on some subspace of $\mathbb{R}^m \times \mathbb{R}^n \times \mathbb{R}$, system (96) is referred to as *singular singularly perturbed system* [54]. Let us introduce the following conditions [54]:

- **C1:** the equation $f_2(x, y_2, t, 0) = 0$ has a smooth isolated root $y_2 = \phi(x,t)$ with $x \in \mathbb{R}^m$, $t \in \mathbb{R}$ and $f_2(x, \phi(x,t), t, 0) = 0$;
- **C2:** the matrix A, defined in (97), with $y_2 = \phi(x,t)$ and $\epsilon' = 0$ has a m-dimensional kernel and m corresponding linearly independent eigenvectors, and the matrix

$$B(x, y_2 = \phi(x,t), t, \epsilon' = 0) = \frac{\partial f_2(x, \phi(x,t), t, 0)}{\partial y_2} \tag{98}$$

  has n eigenvalues $\lambda_i(x,t)$ such that $Re\lambda_i(x,t) \leq -2\alpha$, with $\alpha > 0$;
- **C3:** in the domain

$$\Omega = \{(x, y_2, t, \epsilon') | x \in \mathbb{R}^m, ||y_2 - \phi(x,t)|| \leq \rho, t \in \mathbb{R}, 0 \leq \epsilon' \leq \epsilon_0'\}$$

  the function f_1 and f_2 and the matrix A are continuously differentiable $(k+2)$ times, with $k \geq 0$ for some positive ϵ_0' and ρ.

Then, by introducing the change of variable $y_2 = y_1 + \phi(x,t)$ in (96), we obtain

$$\begin{aligned}
\epsilon' \dot{x} &= C(x,t)y_1 + F_1(x, y_1, t) + \epsilon' X(x, y_1, t, \epsilon') \\
\epsilon' \dot{y}_1 &= B(x,t)y_1 + F_2(x, y_1, t) + \epsilon' Y(x, y_1, t, \epsilon'),
\end{aligned} \tag{99}$$

in which

$$
\begin{aligned}
&C(x,t) = f_{1y_2}(x,\phi(x,t),t,0), \quad B(x,t) = f_{2y_2}(x,\phi(x,t),t,0),\\
&F_1(x,y_1,t) = f_1(x,y_1+\phi(x,t),t,0) - C(x,t)y_1,\\
&F_2(x,y_2,t) = f_2(x,y_1+\phi(x,t),t,0) - B(x,t)y_1,\\
&\epsilon^{'} X(x,y_1,t,\epsilon^{'}) = f_1(x,y_1+\phi(x,t),t,\epsilon^{'}) - f_1(x,y_1+\phi(x,t),t,0)\\
&\epsilon^{'} Y(x,y_1,t,\epsilon^{'}) = f_2(x,y_1+\phi(x,t),t,\epsilon^{'}) - f_2(x,y_1+\phi(x,t),t,0).
\end{aligned}
\tag{100}
$$

with F_i (i=1,2) satisfying $||F_i(x,y_1,t)|| = O(||y_1||^2)$ and $\epsilon^{'-1}F_i(x,\epsilon^{'}y,t)$ continuous in Ω, with Ω defined in condition **C3** [54]. At this point we can apply Theorem 7.1 in [54], which allows us to claim that if conditions **C1** - **C3** are satisfied, then there exists $\epsilon_1^{'}$, $0 < \epsilon_1^{'} < \epsilon_0^{'}$, such that, for any $\epsilon^{'} \in (0,\epsilon_1^{'})$, system (99) has a unique slow integral manifold $y_1 = \epsilon^{'} h(x,t,\epsilon^{'})$ exponentially attractive and the motion along this manifold is described by the equation:

$$
\dot{\bar{x}} = X_1(\bar{x},t,\epsilon^{'}) \tag{101}
$$

in which $X_1(\bar{x},t,\epsilon^{'}) = C(\bar{x},t)h(\bar{x},t,\epsilon^{'}) + X(\bar{x},\epsilon^{'}h,t,\epsilon^{'}) + \epsilon^{'-1}F_1(\bar{x},\epsilon^{'}h,t)$ and the function $h(x,t,\epsilon^{'})$ is k times continuously differentiable with respect to x and t [54, 57]. Since the slow integral manifold is exponentially attractive for a sufficiently small $\epsilon^{'}$, then, for any solution $x(t), y_1(t), x(t_0) = x_0, y_1(t_0) = y_{10}$ of (99) with $|y_{10} - \epsilon^{'}h(x_0,t_0,\epsilon^{'})|$ sufficiently small, we have a solution of (101) such that

$$
x(t) = \bar{x}(t) + \zeta_1(t), \qquad y_1(t) = \epsilon^{'}h(\bar{x}(t),t,\epsilon^{'}) + \zeta_2(t), \tag{102}
$$

with $\zeta_i(t) = O(e^{-(\alpha/\epsilon^{'})(t-t_0)})$, $i = 1,2$, and $t \geq t_0$ ([54], [58], [59](Chapter 6). This allows us to determine the behavior of the trajectories of the original system near the integral manifold by analyzing the behavior of the trajectories of the reduced system (101).

In order to find $h(x,t,\epsilon^{'})$, it is important to note that the change of variable $y = y_1/\epsilon^{'}$ allows us to re-write (99) in the standard singular perturbation form:

$$
\begin{aligned}
\dot{x} &= \tilde{X}(x,y,t,\epsilon^{'}), \quad x \in \mathbb{R}^m, \ t \in \mathbb{R},\\
\epsilon^{'}\dot{y} &= \tilde{Y}(x,y,t,\epsilon^{'}), \quad x \in \mathbb{R}^n,
\end{aligned}
\tag{103}
$$

in which $\tilde{X}(x,y,t,\epsilon^{'}) = C(x,t)y + \epsilon^{'-1}F_1(x,\epsilon^{'}y,t) + X(x,\epsilon^{'}y,t,\epsilon^{'})$, $\tilde{Y}(x,y,t,\epsilon^{'}) = B(x,t)y + \epsilon^{'-1}F_2(x,\epsilon^{'}y,t) + Y(x,\epsilon^{'}y,t,\epsilon^{'})$. Since F_i (i=1,2) satisfy $||F_i(x,y_1,t)|| = O(||y_1||^2)$ in Ω, then $\epsilon^{'-1}F_i(x,\epsilon^{'}y,t)$ are well defined as $\epsilon^{'}$ approaches zero [54]. Then, being conditions C1 - C3 satisfied, it is possible to show that, defining $y = h_0(x,t)$ the smooth isolated root of $\tilde{Y}(x,y,t,0) = 0$, the eigenvalues λ_i of the matrix $(\partial\tilde{Y}/\partial y)(x,h_0(x,t),t,0)$ satisfy the inequality $Re(\lambda_i) \leq -2\alpha$, with $\alpha > 0$. Then the integral manifold $y = y_1/\epsilon^{'} = h(x,t,\epsilon^{'})$ can be calculated as asymptotic expansion in integer powers of $\epsilon^{'}$, $h(x,t,\epsilon^{'}) = h_0(x,t) + \epsilon^{'}h_1(x,t) + ... + \epsilon^{'k}h_k(x,t) + ...$, whose coefficients are smooth function with bounded norm [56] and they can be found substituting the expansion in the second equation of (103), obtaining [54]

$$
\epsilon^{'}\frac{\partial h}{\partial t} + \epsilon^{'}\frac{\partial h}{\partial x}\tilde{X}(x,h,t,\epsilon^{'}) = \tilde{Y}(x,h,t,\epsilon^{'}). \tag{104}
$$

Now, it is possible to show that system (95) is a singular singularly perturbed system. Defining

$$
\begin{aligned}
&x = \begin{pmatrix}\bar{D}^A\\ \bar{D}^R\end{pmatrix}, \quad y_2 = \bar{D},\\
&f_1 = \begin{pmatrix}(u_0^A + u^A + \bar{D}^A)\bar{D} - \epsilon'(c+\bar{D}^R)\bar{D}^A\\ (u_0^R + u^R + \alpha\bar{D}^R)\bar{D} - \mu\epsilon'(cb+\bar{D}^A)\bar{D}^R\end{pmatrix},\\
&f_2 = \epsilon^{'}[\mu(cb+\bar{D}^A)\bar{D}^R + (c+\bar{D}^R)\bar{D}^A] - (u_0^A + u^A + \bar{D}^A + u_0^R + u^R + \alpha\bar{D}^R)\bar{D},
\end{aligned}
\tag{105}
$$

it is possible to show that $\phi(x) = 0$ and that the matrix A as defined in (97) with $\bar{D} = 0$ and $\epsilon^{'} = 0$ can be written as follows:

$$A(x) = \begin{pmatrix} 0 & 0 & (u^A + u_0^A + \bar{D}^A) \\ 0 & 0 & (u^R + u_0^R + \alpha\bar{D}^R) \\ 0 & 0 & -(u^A + u_0^A + \bar{D}^A + u^R + u_0^R + \alpha\bar{D}^R) \end{pmatrix}. \tag{106}$$

This matrix has two zero eigenvalues and two corresponding linearly independent eigenvectors. Furthermore, the matrix B defined in (98) can be written as $B = -(u^A + u_0^A + \bar{D}^A + u^R + u_0^R + \alpha\bar{D}^R)$ and, in the case where no inputs are applied ($u^A = u^R = 0$), it has always negative real part if $u_0^R + u_0^A \geq l$ with $l > 0$. To find the slow integral manifold and the reduced system, let us introduce in (95) the change of variable $\tilde{D} = \bar{D}/\epsilon^{'}$, obtaining

$$\begin{aligned} \frac{d\bar{D}^A}{d\bar{\tau}} &= (u_0^A + u^A + \bar{D}^A)\tilde{D} - (c + \bar{D}^R)\bar{D}^A \\ \frac{d\bar{D}^R}{d\bar{\tau}} &= (u_0^R + u^R + \alpha\bar{D}^R)\tilde{D} - \mu(cb + \bar{D}^A)\bar{D}^R \\ \epsilon^{'}\frac{d\tilde{D}}{d\bar{\tau}} &= [\mu(cb + \bar{D}^A)\bar{D}^R + (c + \bar{D}^R)\bar{D}^A] - (u_0^A + u^A + \bar{D}^A + u_0^R + u^R + \alpha\bar{D}^R)\tilde{D}. \end{aligned} \tag{107}$$

To calculate the slow integral manifold, let us construct the asymptotic expansion of $\tilde{D}$:

$$\tilde{D} = h(\bar{D}^A, \bar{D}^R, \epsilon^{'}) = h_0(\bar{D}^A, \bar{D}^R) + \epsilon^{'} h_1(\bar{D}^A, \bar{D}^R) + O(\epsilon^{'2}). \tag{108}$$

Substituting (108) in the last ODE of (107), we obtain

$$\epsilon^{'}\frac{dh}{d\bar{\tau}} = \epsilon^{'}(\frac{\partial h}{\partial \bar{D}^A}\frac{d\bar{D}^A}{d\bar{\tau}} + \frac{\partial h}{\partial \bar{D}^R}\frac{d\bar{D}^R}{d\bar{\tau}}) = [\mu(cb + \bar{D}^A)\bar{D}^R + (c + \bar{D}^R)\bar{D}^A] - (u_0^A + u^A + \bar{D}^A + u_0^R + u^R + \alpha\bar{D}^R)h. \tag{109}$$

To calculate h_0 and h_1 we equate the terms on the left and right hand side multiplied by the same power of $\epsilon^{'}$, obtaining

$$\begin{aligned} h_0 &= \frac{[\mu(cb + \bar{D}^A)\bar{D}^R + (c + \bar{D}^R)\bar{D}^A]}{(u_0^A + u^A + \bar{D}^A + u_0^R + u^R + \alpha\bar{D}^R)}, \\ h_1 &= -\frac{\frac{\partial h_0}{\partial \bar{D}^R}((u_0^R + u^R + \alpha\bar{D}^R)h_0 - \mu(cb + \bar{D}^A)\bar{D}^R) + \frac{\partial h_0}{\partial \bar{D}^A}((u_0^A + u^A + \bar{D}^A)h_0 - (c + \bar{D}^R)\bar{D}^A)}{(u_0^A + u^A + \bar{D}^A + u_0^R + u^R + \alpha\bar{D}^R)}. \end{aligned} \tag{110}$$

Since $\frac{\partial h_0}{\partial \bar{D}^R}$ and $\frac{\partial h_0}{\partial \bar{D}^A}$ are bounded (and then $\epsilon^{'}\frac{\partial h_0}{\partial \bar{D}^R}, \epsilon^{'}\frac{\partial h_0}{\partial \bar{D}^A} \ll 1$ for a sufficiently small $\epsilon^{'}$), substituting (110) into (108), we obtain

$$\tilde{D} = \frac{[\mu(cb + \bar{D}^A)\bar{D}^R + (c + \bar{D}^R)\bar{D}^A]}{(u_0^A + u^A + \bar{D}^A + u_0^R + u^R + \alpha\bar{D}^R)}. \tag{111}$$

Now, substituting (111) into (107), we obtain the reduced system as follows:

$$\begin{aligned} \frac{d\bar{D}^A}{d\bar{\tau}} &= \left(\frac{\mu(cb + \bar{D}^A)(u^A + \bar{D}^A)}{(u_0^A + u^A + \bar{D}^A) + (u_0^R + u^R + \alpha\bar{D}^R)}\right)\bar{D}^R \\ &\quad - \left(\frac{(c + \bar{D}^R)(u_0^R + u^R + \alpha\bar{D}^R)}{(u_0^A + u^A + \bar{D}^A) + (u_0^R + u^R + \alpha\bar{D}^R)}\right)\bar{D}^A \\ \frac{d\bar{D}^R}{d\bar{\tau}} &= \left(\frac{(c + \bar{D}^R)(u_0^R + u^R + \alpha\bar{D}^R)}{(u_0^A + u^A + \bar{D}^A) + (u_0^R + u^R + \alpha\bar{D}^R)}\right)\bar{D}^A \\ &\quad - \left(\frac{\mu(cb + \bar{D}^A)(u_0^A + u^A + \bar{D}^A)}{(u_0^A + u^A + \bar{D}^A) + (u_0^R + u^R + \alpha\bar{D}^R)}\right)\bar{D}^R. \end{aligned} \tag{112}$$

Summing the two ODEs written above, it is possible to notice that $\frac{d\bar{D}^A}{d\bar{\tau}} + \frac{d\bar{D}^R}{d\bar{\tau}} = 0$, that is $\bar{D}^A + \bar{D}^R = \text{constant}$. In particular, since $\bar{D}^A + \bar{D}^R + \bar{D} = 1$ and $\bar{D} = 0$ for $\epsilon^{'} = 0$, we have that $\bar{D}^A + \bar{D}^R = 1$ for $\epsilon^{'} = 0$.

We further validated *via* simulation that system (112) is a proper reduction of the full system (95) when $\epsilon^{'}$ is small. We perform simulations of both systems for different values of $\epsilon^{'}$ and initial conditions, showing that the trajectories of $\bar{D}^R$ and $\bar{D}^A$ of the full and reduced systems become closer as $\epsilon^{'}$ decreases (Fig E).

It is important to point out that in the reduction we let $\epsilon = c\epsilon^{'}$, with $c = O(1)$, implying that as $\epsilon^{'}$ decreases also ϵ decreases. Given the definition of ϵ and $\epsilon^{'}$, it is reasonable that, if the specifically recruited erasure reaction is slow compared to the auto and cross-catalysis reactions, then also the basal erasure reaction due to non-specific binding of enzymes and dilution is slow compared to the auto and cross-catalysis reactions. This is consistent with the fact that the rates of enzymatic reactions where enzyme-substrate binding is highly specific tend to be larger than the rates of enzymatic reactions occurring though non-specific enzyme substrate binding and to removal due to simple dilution from cell growth.

Now, multiplying both sides by $D_{tot}(k_E^A D_{tot})$, system (112) can be rewritten in a dimensional form:

$$\begin{aligned}
\dot{D}^A &= \left(\frac{(k_{W0}^A + k_W^A + k_M^A D^A)(\delta + \bar{k}_E^R + k_E^R D^A)}{(k_{W0}^A + k_W^A + k_M^A D^A) + (k_{W0}^R + k_W^R + k_M^R D^R)}\right) D^R \\
&\quad - \left(\frac{(k_{W0}^R + k_W^R + k_M^R D^R)(\delta + \bar{k}_E^A + k_E^A D^R)}{(k_{W0}^A + k_W^A + k_M^A D^A) + (k_{W0}^R + k_W^R + k_M^R D^R)}\right) D^A \\
\dot{D}^R &= \left(\frac{(k_{W0}^R + k_W^R + k_M^R D^R)(\delta + \bar{k}_E^A + k_E^A D^R)}{(k_{W0}^A + k_W^A + k_M^A D^A) + (k_{W0}^R + k_W^R + k_M^R D^R)}\right) D^A \\
&\quad - \left(\frac{(k_{W0}^A + k_W^A + k_M^A D^A)(\delta + \bar{k}_E^R + k_E^R D^A)}{(k_{W0}^A + k_W^A + k_M^A D^A) + (k_{W0}^R + k_W^R + k_M^R D^R)}\right) D^R,
\end{aligned} \tag{113}$$

or, since $D^R + D^A = D_{tot}$, it can be written as

$$\begin{aligned}
\dot{D}^R &= \left(\frac{(k_{W0}^R + k_W^R + k_M^R D^R)(\delta + \bar{k}_E^A + k_E^A D^R)}{(k_{W0}^A + k_W^A + k_M^A (D_{tot} - D^R)) + (k_{W0}^R + k_W^R + k_M^R D^R)}\right) (D_{tot} - D^R) \\
&\quad - \left(\frac{(k_{W0}^A + k_W^A + k_M^A (D_{tot} - D^R))(\delta + \bar{k}_E^R + k_E^R (D_{tot} - D^R))}{(k_{W0}^A + k_W^A + k_M^A (D_{tot} - D^R)) + (k_{W0}^R + k_W^R + k_M^R D^R)}\right) D^R.
\end{aligned} \tag{114}$$

Finally, the system can also be represented through the following simplified chemical reactions:

$$\begin{aligned}
\mathrm{D^A} &\xrightarrow{k_{AR}} \mathrm{D^R}, \quad k_{AR} = \left(\frac{(k_{W0}^R + k_W^R + k_M^R D^R)(\delta + \bar{k}_E^A + k_E^A D^R)}{(k_{W0}^A + k_W^A + k_M^A D^A) + (k_{W0}^R + k_W^R + k_M^R D^R)}\right), \\
\mathrm{D^R} &\xrightarrow{k_{RA}} \mathrm{D^A}, \quad k_{RA} = \left(\frac{(k_{W0}^A + k_W^A + k_M^A D^A)(\delta + \bar{k}_E^R + k_E^R D^A)}{(k_{W0}^A + k_W^A + k_M^A D^A) + (k_{W0}^R + k_W^R + k_M^R D^R)}\right).
\end{aligned} \tag{115}$$

2.3 Derivation of stationary distribution formula

Since $n^R + n^A = \mathrm{D_{tot}}$, the reduced chemical reaction system (115) can be represented by a one-dimensional Markov chain in which the state x represents the total number of repressive histone modifications, that is, $x = n^R$. In particular, the state x can vary between zero and $\mathrm{D_{tot}}$. Furthermore, let us define the transition rate from state $x = i$ to state $x = j$ as $q_{i,j}$ [60]. Specifically, $q_{i,j}$

for our Markov chain takes the following form:

$$
\begin{aligned}
q_{x,(x+1)} &= \left(\frac{(k_{W0}^R + k_W^R + \frac{k_M^R}{\Omega} x)(\delta + \bar{k}_E^A + \frac{k_E^A}{\Omega} x)}{(k_{W0}^A + k_W^A + \frac{k_M^A}{\Omega}(\mathrm{D_{tot}} - x)) + (k_{W0}^R + k_W^R + \frac{k_M^R}{\Omega} x)} \right) (\mathrm{D_{tot}} - x), \\
q_{x,(x-1)} &= \left(\frac{(k_{W0}^A + k_W^A + \frac{k_M^A}{\Omega}(\mathrm{D_{tot}} - x))(\delta + \bar{k}_E^R + \frac{k_E^R}{\Omega}(\mathrm{D_{tot}} - x))}{(k_{W0}^A + k_W^A + \frac{k_M^A}{\Omega}(\mathrm{D_{tot}} - x)) + (k_{W0}^R + k_W^R + \frac{k_M^R}{\Omega} x)} \right) x,
\end{aligned}
\tag{116}
$$

for $x \in [0, \mathrm{D_{tot}}]$ [49, 60]. Since this Markov chain is irreducible and reversible, we can apply detailed balance [60] to determine an analytical expression for the stationary probability distribution $\pi(x)$. According to detailed balance, for each state x, the product of $\pi(x)$ and the rate $q_{x,(x-1)}$ is equal to the product of $\pi(x-1)$ and the rate $q_{(x-1),x}$, that is $\pi(x)q_{x,(x-1)} = \pi(x-1)q_{(x-1),x}$ or

$$\pi(x) = \frac{q_{(x-1),x}}{q_{x,(x-1)}} \pi(x-1). \tag{117}$$

Applying this equality recursively we are able to express $\pi(x)$ for any state x as a function of $\pi(0)$. In order to derive this formula, let us consider a particular state $x = \bar{x}$. Formula (117) allows us to express $\pi(\bar{x})$ as a function of $\pi(\bar{x}-1)$, that is

$$\pi(\bar{x}) = \frac{q_{(\bar{x}-1),\bar{x}}}{q_{\bar{x},(\bar{x}-1)}} \pi(\bar{x}-1). \tag{118}$$

Since (117) holds for any state x, it can be rewritten also for state $x = \bar{x} - 1$, obtaining

$$\pi(\bar{x}-1) = \frac{q_{(\bar{x}-2),(\bar{x}-1)}}{q_{(\bar{x}-1),(\bar{x}-2)}} \pi(\bar{x}-2). \tag{119}$$

If we substitute in (118) $\pi(\bar{x}-1)$ obtained in (119), we obtain an expression for $\pi(\bar{x})$ as a function of $\pi(\bar{x}-2)$:

$$\pi(\bar{x}) = \frac{q_{(\bar{x}-1),\bar{x}}}{q_{\bar{x},(\bar{x}-1)}} \frac{q_{(\bar{x}-2),(\bar{x}-1)}}{q_{(\bar{x}-1),(\bar{x}-2)}} \pi(\bar{x}-2). \tag{120}$$

Applying (117) recursively for each state $x \in [1, \bar{x}]$ we then obtain an expression for $\pi(\bar{x})$ as a function of $\pi(0)$:

$$\pi(\bar{x}) = \frac{q_{(\bar{x}-1),\bar{x}}}{q_{\bar{x},(\bar{x}-1)}} \frac{q_{(\bar{x}-2),(\bar{x}-1)}}{q_{(\bar{x}-1),(\bar{x}-2)}} \frac{q_{(\bar{x}-3),(\bar{x}-2)}}{q_{(\bar{x}-2),(\bar{x}-3)}} \frac{q_{(\bar{x}-4),(\bar{x}-3)}}{q_{(\bar{x}-3),(\bar{x}-4)}} \ldots \frac{q_{1,2}}{q_{2,1}} \frac{q_{0,1}}{q_{1,0}} \pi(0). \tag{121}$$

Since this derivation does not depend on the specific initial state $\bar{x}$ we choose, formula (121) can be rewritten for a generic state x as follows:

$$\pi(x) = \prod_{i=1}^{x} \frac{q_{(i-1),i}}{q_{i,(i-1)}} \pi(0). \tag{122}$$

Now, in order to find an analytical expression for $\pi(0)$, we use $\sum_{j=0}^{\mathrm{D_{tot}}} \pi(j) = \sum_{j=1}^{\mathrm{D_{tot}}} \left(\prod_{i=1}^{j} \frac{q_{(i-1),i}}{q_{i,(i-1)}} \right) \pi(0) + \pi(0) = 1$ so that we can express $\pi(0)$ as follows:

$$\pi(0) = \frac{1}{\left(1 + \sum_{j=1}^{\mathrm{D_{tot}}} \left(\prod_{i=1}^{j} \frac{q_{(i-1),i}}{q_{i,(i-1)}} \right)\right)}. \tag{123}$$

Substituting in (122) the $\pi(0)$ expression obtained in (123), the stationary probability distribution $\pi(x)$ can finally be expressed as

$$\pi(x) = \prod_{i=1}^{x} \frac{q_{(i-1),i}}{q_{i,(i-1)}} \pi(0) = \frac{\prod_{i=1}^{x} \frac{q_{(i-1),i}}{q_{i,(i-1)}}}{\left(1 + \sum_{j=1}^{\mathrm{D_{tot}}} \left(\prod_{i=1}^{j} \frac{q_{(i-1),i}}{q_{i,(i-1)}}\right)\right)} \tag{124}$$

for any $x \in [1, \mathrm{D_{tot}}]$. Now, let us compute $\prod_{i=1}^{x} \frac{q_{(i-1),i}}{q_{i,(i-1)}}$ for our system by writing explicitly the expression for the rates and rearranging properly the terms. In particular, for any $x \in [1, \mathrm{D_{tot}} - 1]$, it can be written as

$$\begin{aligned}
\prod_{i=1}^{x} \frac{q_{(i-1),i}}{q_{i,(i-1)}} =& \binom{\mathrm{D_{tot}}}{x} \left(1 + x \frac{\frac{k_M^R}{\Omega} - \frac{k_M^A}{\Omega}}{\frac{k_M^A}{\Omega}\mathrm{D_{tot}} + k_{W0}^A + k_W^A + k_{W0}^R + k_W^R}\right) \cdot \\
&\cdot \prod_{i=1}^{x-1} \left(\frac{(k_{W0}^R + k_W^R + \frac{k_M^R}{\Omega} i)(\delta + \bar{k}_E^A + \frac{k_E^A}{\Omega} i)}{(k_{W0}^A + k_W^A + \frac{k_M^A}{\Omega}(\mathrm{D_{tot}} - i))(\delta + \bar{k}_E^R + \frac{k_E^R}{\Omega}(\mathrm{D_{tot}} - i))} \right) \cdot \\
&\cdot \frac{(k_{W0}^R + k_W^R)(\delta + \bar{k}_E^A)}{(k_{W0}^A + k_W^A + \frac{k_M^A}{\Omega}(\mathrm{D_{tot}} - x))(\delta + \bar{k}_E^R + \frac{k_E^R}{\Omega}(\mathrm{D_{tot}} - x))} \\
=& \binom{\mathrm{D_{tot}}}{x} \left(1 + \frac{x}{\mathrm{D_{tot}}} \frac{\alpha - 1}{1 + u_0^A + u^A + u_0^R + u^R}\right) \cdot \\
&\cdot \prod_{i=1}^{x-1} \left(\frac{(u_0^R + u^R + \alpha \frac{i}{\mathrm{D_{tot}}})(\epsilon + \epsilon' \frac{i}{\mathrm{D_{tot}}})}{(u_0^A + u^A + \frac{(\mathrm{D_{tot}} - i)}{\mathrm{D_{tot}}})\mu(b\epsilon + \epsilon' \frac{(\mathrm{D_{tot}} - i)}{\mathrm{D_{tot}}})} \right) \cdot \\
&\cdot \frac{(u_0^R + u^R)\epsilon}{(u_0^A + u^A + \frac{(\mathrm{D_{tot}} - x)}{\mathrm{D_{tot}}})\mu(b\epsilon + \epsilon' \frac{(\mathrm{D_{tot}} - x)}{\mathrm{D_{tot}}})},
\end{aligned} \tag{125}$$

in which the final formula has been obtained by dividing numerator and denominator by $(\frac{k_M^A \mathrm{D_{tot}}}{\Omega})$ in each of the factors. For $x = \mathrm{D_{tot}}$ it can be written as

$$\begin{aligned}
\prod_{i=1}^{\mathrm{D_{tot}}} \frac{q_{(i-1),i}}{q_{i,(i-1)}} =& \left(1 + \mathrm{D_{tot}} \frac{\frac{k_M^R}{\Omega} - \frac{k_M^A}{\Omega}}{\frac{k_M^A}{\Omega}\mathrm{D_{tot}} + k_{W0}^A + k_W^A + k_{W0}^R + k_W^R}\right) \cdot \\
&\cdot \prod_{i=1}^{\mathrm{D_{tot}}-1} \left(\frac{(k_{W0}^R + k_W^R + \frac{k_M^R}{\Omega} i)(\delta + \bar{k}_E^A + \frac{k_E^A}{\Omega} i)}{(k_{W0}^A + k_W^A + \frac{k_M^A}{\Omega}(\mathrm{D_{tot}} - i))(\delta + \bar{k}_E^R + \frac{k_E^R}{\Omega}(\mathrm{D_{tot}} - i))} \right) \frac{(k_{W0}^R + k_W^R)(\delta + \bar{k}_E^A)}{(k_{W0}^A + k_W^A)(\delta + \bar{k}_E^R)} \\
=& \left(1 + \frac{\alpha - 1}{1 + u_0^A + u^A + u_0^R + u^R}\right) \cdot \\
&\cdot \prod_{i=1}^{\mathrm{D_{tot}}-1} \left(\frac{(u_0^R + u^R + \alpha \frac{i}{\mathrm{D_{tot}}})(\epsilon + \epsilon' \frac{i}{\mathrm{D_{tot}}})}{(u_0^A + u^A + \frac{(\mathrm{D_{tot}} - i)}{\mathrm{D_{tot}}})\mu(b\epsilon + \epsilon' \frac{(\mathrm{D_{tot}} - i)}{\mathrm{D_{tot}}})} \right) \frac{u_0^R + u^R}{(u_0^A + u^A)\mu b},
\end{aligned} \tag{126}$$

in which the final formula has been obtained by dividing numerator and denominator by $(\frac{k_M^A \mathrm{D_{tot}}}{\Omega})$ in each of the factors. Then, assuming that $\epsilon' \neq 0$, equations (125) and (126) show that, in the limiting condition where $\epsilon \to 0$, $\prod_{i=1}^{x} \frac{q_{(i-1),i}}{q_{i,(i-1)}}$ tends to zero unless $x = \mathrm{D_{tot}}$. This implies that the

stationary probability is such that

$$\lim_{\epsilon\to 0}\pi(x)=\pi_{\epsilon 0}(x)=\begin{cases}\frac{1}{1+P} & \text{if } x=0\\ 0 & \text{if } x\neq 0,\mathrm{D_{tot}}\\ \frac{P}{1+P} & \text{if } x=\mathrm{D_{tot}}\end{cases}$$

with

$$P=\frac{(u_0^A+u^A+u_0^R+u^R+\alpha)(u_0^R+u^R)}{(u_0^A+u^A+u_0^R+u^R+1)(u_0^A+u^A)b}\prod_{i=1}^{\mathrm{D_{tot}}-1}\left(\frac{u_0^R+u^R+\alpha\frac{i}{\mathrm{D_{tot}}}}{u_0^A+u^A+\frac{\mathrm{D_{tot}}-i}{\mathrm{D_{tot}}}}\right)\left(\frac{1}{\mu}\right)^{\mathrm{D_{tot}}}. \tag{127}$$

This implies that, if ϵ tends to zero, $\pi(x)\to 0$ except for $x=\mathrm{D_{tot}}$ (fully repressed gene state) and $x=0$ (fully active gene state), that is, the probability of finding the system in one of the intermediate states is almost zero. Furthermore, given the expression for P in (127), it is possible to notice that if $\mu\ll 1$, $\pi_{\epsilon 0}(0)\to 0$ and $\pi_{\epsilon 0}(\mathrm{D_{tot}})\to 1$ and this implies that, under these parameter conditions, the probability of finding the gene in an active state is close to zero. However, this stationary probability distribution can be modified by varying the input stimuli (u^A and u^R). In particular, increasing u^R leads $\pi_{\epsilon 0}(\mathrm{D_{tot}})$ to increase and $\pi_{\epsilon 0}(0)$ to decrease. By contrast, increasing u^A is going to lead to $\pi_{\epsilon 0}(\mathrm{D_{tot}})\to 0$ and $\pi_{\epsilon 0}(0)\to 1$ and, looking at the expression for P (127), for smaller μ, it is required a larger u^A to decrease $\pi_{\epsilon 0}(\mathrm{D_{tot}})$ to the same level.

2.4 Derivation of time to memory loss formula for the 2D model

Let us define the time to memory loss of the fully repressed gene state, $\tau^0_{\mathrm{D_{tot}}}$, as the expected value of the first time at which the state x of the Markov chain hits 0, starting from $x=\mathrm{D_{tot}}$. In particular, we define the hitting time of $x=0$ starting from $x=i$ as $t_i^0:=[\ \inf\{t\geq 0: x(t)=0$ with $x(0)=i\}$ with $i\in[0,\mathrm{D_{tot}}]]$. Then, the time to memory loss of the fully repressed gene state can be defined as the expected value of $t^0_{\mathrm{D_{tot}}}$, that is, $\tau^0_{\mathrm{D_{tot}}}=\mathbb{E}(t^0_{\mathrm{D_{tot}}})$. In order to compute $\tau^0_{\mathrm{D_{tot}}}$ we use first step analysis [61], which allows us to evaluate the vector of expected hitting times $\tau^0=(\tau_i^0: i\in[0,\mathrm{D_{tot}}])$ as the solution of the following system of equations

$$\begin{cases}\tau_i^0=0 & \text{if } i=0\\ -\sum_{j=0}^{\mathrm{D_{tot}}}q_{i,j}\tau_j^0=1 & \text{if } i\neq 0,\end{cases} \tag{128}$$

with $q_{i,j}$ defined as the rate of going from state i to state j. In our one-dimensional Markov chain the state i can only go either to $i+1$ or to $i-1$. Then, defining $q_{i,i+1}=\alpha_i$, $\gamma_i=q_{i,i-1}$ and $q_{i,i}=-q_i=-\sum_{j=0,j\neq i}^{\mathrm{D_{tot}}}q_{i,j}$ with q_i defined as the rate of leaving state i [61] and then rewriting $q_{i,i}$ as $-(\alpha_i+\gamma_i)$, each equation of system (128) can be expressed as

$$\tau_i^0=\frac{1}{\alpha_i+\gamma_i}+\frac{\alpha_i}{\alpha_i+\gamma_i}\tau^0_{i+1}+\frac{\gamma_i}{\alpha_i+\gamma_i}\tau^0_{i-1},\qquad \text{if } i\in[1,\mathrm{D_{tot}}-1] \tag{129}$$

$$\tau^0_{\mathrm{D_{tot}}}=\frac{1}{\gamma_{\mathrm{D_{tot}}}}+\tau^0_{\mathrm{D_{tot}}-1}. \tag{130}$$

In particular, we can rearrange (129) as $\alpha_i(\tau_i^0-\tau^0_{i+1})=1+\gamma_i(\tau^0_{i-1}-\tau_i^0)$ and, defining $\Delta\tau_i^0=(\tau_i^0-\tau^0_{i+1})$, (129) can be rewritten as

$$\Delta\tau_i^0=\frac{1}{\alpha_i}+\frac{\gamma_i}{\alpha_i}\Delta\tau^0_{i-1}. \tag{131}$$

In equation (131), we can express $\Delta\tau_i^0$ as a function of $\Delta\tau_{i-1}^0$ and thus, applying (131) recursively, we can express $\Delta\tau_i^0$ as a function of $\Delta\tau_0^0$ as follows:

$$\Delta\tau_i^0 = \sum_{j=1}^{i} \frac{1}{\alpha_j}\frac{r_j}{r_i} + \frac{\Delta\tau_0^0}{r_i}, \tag{132}$$

in which $r_j = \frac{\alpha_1\alpha_2...\alpha_j}{\gamma_1\gamma_2...\gamma_j}$. In order to evaluate the time to memory loss of the fully repressed gene state $\tau_{\mathrm{D_{tot}}}^0$, we apply $\sum_{i=1}^{\mathrm{D_{tot}}-1}$ to the left and right-hand side of (132), that is $\sum_{i=1}^{\mathrm{D_{tot}}-1}\left(\Delta\tau_i^0\right) = \sum_{i=1}^{\mathrm{D_{tot}}-1}\left(\sum_{j=1}^{i}\frac{1}{\alpha_j}\frac{r_j}{r_i} + \frac{\Delta\tau_0^0}{r_i}\right)$, in which the left-hand side can be explicitly expressed as

$$\sum_{i=1}^{\mathrm{D_{tot}}-1}\left(\Delta\tau_i^0\right) = \Delta\tau_1^0 + \Delta\tau_2^0 + ... + \Delta\tau_{\mathrm{D_{tot}}-1}^0 = \tau_1^0 - \tau_2^0 + \tau_2^0 - \tau_3^0 + ... + \tau_{\mathrm{D_{tot}}-1}^0 - \tau_{\mathrm{D_{tot}}}^0 = \tau_1^0 - \tau_{\mathrm{D_{tot}}}^0$$

from which, solving for $\tau_{\mathrm{D_{tot}}}^0$, we obtain

$$\tau_{\mathrm{D_{tot}}}^0 = \tau_1^0 - \sum_{i=1}^{\mathrm{D_{tot}}-1}\left(\sum_{j=1}^{i}\frac{1}{\alpha_j}\frac{r_j}{r_i} - \frac{\tau_1^0}{r_i}\right). \tag{133}$$

At this point, we need to evaluate τ_1^0. To this end, we rewrite equation (132) for $i = \mathrm{D_{tot}}$:

$$\tau_{\mathrm{D_{tot}}-1}^0 - \tau_{\mathrm{D_{tot}}}^0 = \sum_{j=1}^{\mathrm{D_{tot}}-1}\frac{1}{\alpha_j}\frac{r_j}{r_{\mathrm{D_{tot}}-1}} - \frac{\tau_1^0}{r_{\mathrm{D_{tot}}-1}}, \tag{134}$$

in which we have substituted $\Delta\tau_{\mathrm{D_{tot}}-1}^0$ with $\tau_{\mathrm{D_{tot}}-1}^0 - \tau_{\mathrm{D_{tot}}}^0$ and $\Delta\tau_0^0$ with $\tau_0^0 - \tau_1^0 = -\tau_1^0$ ($\tau_0^0 = 0$ as given in (128)). Now, $\tau_{\mathrm{D_{tot}}-1}^0 - \tau_{\mathrm{D_{tot}}}^0$ can be expressed as $-\frac{1}{\gamma_{\mathrm{D_{tot}}}}$ (it can be obtained by rearranging (130)) and then τ_1^0 can be written as

$$\tau_1^0 = \sum_{j=1}^{\mathrm{D_{tot}}-1}\frac{r_j}{\alpha_j} + \frac{r_{\mathrm{D_{tot}}-1}}{\gamma_{\mathrm{D_{tot}}}}. \tag{135}$$

Finally, plugging this expression for τ_1^0 into (133), we obtain the expression for the time to memory loss of the fully repressed gene state $\tau_{\mathrm{D_{tot}}}^0$ as a function of the system parameters:

$$\begin{aligned}
\tau_{\mathrm{D_{tot}}}^0 &= \sum_{i=1}^{\mathrm{D_{tot}}-1}\frac{r_i}{\alpha_i} + \frac{r_{\mathrm{D_{tot}}-1}}{\gamma_{\mathrm{D_{tot}}}} - \sum_{i=1}^{\mathrm{D_{tot}}-1}\left(\sum_{j=1}^{i}\frac{1}{\alpha_j}\frac{r_j}{r_i} - \sum_{j=1}^{\mathrm{D_{tot}}-1}\frac{1}{\alpha_j}\frac{r_j}{r_i} - \frac{r_{\mathrm{D_{tot}}-1}}{\gamma_{\mathrm{D_{tot}}} r_i}\right) \\
&= \sum_{i=1}^{\mathrm{D_{tot}}-1}\frac{r_i}{\alpha_i} + \frac{r_{\mathrm{D_{tot}}-1}}{\gamma_{\mathrm{D_{tot}}}} + \sum_{i=1}^{\mathrm{D_{tot}}-2}\sum_{j=i+1}^{\mathrm{D_{tot}}-1}\frac{1}{\alpha_j}\frac{r_j}{r_i} + \sum_{i=1}^{\mathrm{D_{tot}}-1}\frac{r_{\mathrm{D_{tot}}-1}}{\gamma_{\mathrm{D_{tot}}} r_i} \\
&= \frac{r_{\mathrm{D_{tot}}-1}}{\gamma_{\mathrm{D_{tot}}}}\left(1 + \sum_{i=1}^{\mathrm{D_{tot}}-1}\frac{1}{r_i}\right) + \sum_{i=1}^{\mathrm{D_{tot}}-1}\frac{r_i}{\alpha_i} + \sum_{i=1}^{\mathrm{D_{tot}}-2}\sum_{j=i+1}^{\mathrm{D_{tot}}-1}\frac{1}{\alpha_j}\frac{r_j}{r_i} \\
&= \frac{r_{\mathrm{D_{tot}}-1}}{\gamma_{\mathrm{D_{tot}}}}\left(1 + \sum_{i=1}^{\mathrm{D_{tot}}-1}\frac{1}{r_i}\right) + \frac{1}{\gamma_1} + \sum_{i=2}^{\mathrm{D_{tot}}-1}\left[\frac{r_{i-1}}{\gamma_i}\left(1 + \sum_{j=1}^{i-1}\frac{1}{r_j}\right)\right].
\end{aligned} \tag{136}$$

In particular, for our Markov chain, α_i and γ_i are given by

$$\begin{aligned}
\alpha_i &= \left(\frac{(k^R_{W0} + k^R_W + \frac{k^R_M}{\Omega} i)(\delta + \bar{k}^A_E + \frac{k^A_E}{\Omega} i)}{(k^A_{W0} + k^A_W + \frac{k^A_M}{\Omega}(\mathrm{D_{tot}} - i)) + (k^R_{W0} + k^R_W + \frac{k^R_M}{\Omega} i)} \right) (\mathrm{D_{tot}} - i) \\
&= \left(\frac{(k^R_{W0} + k^R_W + \frac{k^R_M}{\Omega} i)(\epsilon + \epsilon' \frac{i}{\mathrm{D_{tot}}})}{(u^A_0 + u^A + \frac{(\mathrm{D_{tot}} - i)}{\mathrm{D_{tot}}}) + (u^R_0 + u^R + \alpha \frac{i}{\mathrm{D_{tot}}})} \right) (\mathrm{D_{tot}} - i) \\
\gamma_i &= \left(\frac{(k^A_{W0} + k^A_W + \frac{k^A_M}{\Omega}(\mathrm{D_{tot}} - i))(\delta + \bar{k}^R_E + \frac{k^R_E}{\Omega}(\mathrm{D_{tot}} - i))}{(k^A_{W0} + k^A_W + \frac{k^A_M}{\Omega}(\mathrm{D_{tot}} - i)) + (k^R_{W0} + k^R_W + \frac{k^R_M}{\Omega} i)} \right) i \\
&= \left(\frac{(k^A_{W0} + k^A_W + \frac{k^A_M}{\Omega}(\mathrm{D_{tot}} - i))\mu(b\epsilon + \epsilon' \frac{(\mathrm{D_{tot}} - i)}{\mathrm{D_{tot}}})}{(u^A_0 + u^A + \frac{(\mathrm{D_{tot}} - i)}{\mathrm{D_{tot}}}) + (u^R_0 + u^R + \alpha \frac{i}{\mathrm{D_{tot}}})} \right) i,
\end{aligned} \tag{137}$$

in which the final expressions are obtained by multiplying and dividing the intermediate expression by $\frac{k^A_M \mathrm{D_{tot}}}{\Omega}$. In the absence of external input stimuli and assuming that $\epsilon' \neq 0$, it is possible to notice that, for $\epsilon \ll 1$, the dominant term of $\tau^0_{\mathrm{D_{tot}}}$ is the first addend in (136). Then, by normalizing the time to memory loss with respect $\frac{k^A_M \mathrm{D_{tot}}}{\Omega}$ ($\bar{\tau}^0_{\mathrm{D_{tot}}} = \tau^0_{\mathrm{D_{tot}}} \frac{k^A_M \mathrm{D_{tot}}}{\Omega}$), $\tau^0_{\mathrm{D_{tot}}}$ in the regime $\epsilon \ll 1$ can be re-written as follows:

$$\bar{\tau}^0_{\mathrm{D_{tot}}} = \bar{\tau}_R \approx \frac{K_R}{\mu\epsilon} \left(1 + \sum_{i=1}^{\mathrm{D_{tot}}-1} \frac{K^i_R}{h^i_1(\mu)} \right), \tag{138}$$

with h^i_1 an increasing function, $h^i_1(0) = 0$, K_R and K^i_R functions independent of ϵ and μ, and in which we redefine $\bar{\tau}^0_{\mathrm{D_{tot}}}$ as $\bar{\tau}_R$ to simplify the notation.

In a similar way we can determine the time to memory loss of the fully active gene state, $\tau^{\mathrm{D_{tot}}}_0$, that is the expected value of the first time at which the state x of the Markov chain hits $\mathrm{D_{tot}}$, starting from $x = 0$. In particular, defining $\tilde{r}_j = \frac{\gamma_{\mathrm{D_{tot}}-1}\gamma_{\mathrm{D_{tot}}-2}\cdots\gamma_{\mathrm{D_{tot}}-j}}{\alpha_{\mathrm{D_{tot}}-1}\alpha_{\mathrm{D_{tot}}-2}\cdots\alpha_{\mathrm{D_{tot}}-j}}$, $\tau^{\mathrm{D_{tot}}}_0$ can be written as follows:

$$\tau^{\mathrm{D_{tot}}}_0 = \frac{\tilde{r}_{\mathrm{D_{tot}}-1}}{\alpha_0} \left(1 + \sum_{j=1}^{\mathrm{D_{tot}}-1} \frac{1}{\tilde{r}_i} \right) + \frac{1}{\alpha_{\mathrm{D_{tot}}-1}} + \sum_{i=2}^{\mathrm{D_{tot}}-1} \left[\frac{\tilde{r}_{i-1}}{\alpha_{\mathrm{D_{tot}}-i}} \left(1 + \sum_{j=1}^{i-1} \frac{1}{\tilde{r}_j} \right) \right]. \tag{139}$$

Also in this case, in the absence of external input stimuli and assuming that $\epsilon' \neq 0$, it is possible to notice that, for $\epsilon \ll 1$, the dominant term of $\tau^{\mathrm{D_{tot}}}_0$ is the first addend in (139). Then, by normalizing the time to memory loss with respect $\frac{k^A_M \mathrm{D_{tot}}}{\Omega}$ ($\bar{\tau}^{\mathrm{D_{tot}}}_0 = \tau^{\mathrm{D_{tot}}}_0 \frac{k^A_M \mathrm{D_{tot}}}{\Omega}$), $\tau^{\mathrm{D_{tot}}}_0$ in the regime $\epsilon \ll 1$ can be re-written as follows:

$$\bar{\tau}^{\mathrm{D_{tot}}}_0 = \bar{\tau}_A \approx \frac{K_A}{\epsilon} \left(1 + \sum_{i=1}^{\mathrm{D_{tot}}-1} \frac{h^i_2(\mu)}{K^i_A} \right), \tag{140}$$

with h^i_2 an increasing function, $h^i_2(0) = 0$, K_A and K^i_A functions independent of ϵ and μ, and in which we redefine $\bar{\tau}^{\mathrm{D_{tot}}}_0$ as $\bar{\tau}_A$ to simplify the notation.

3 Detailed analysis of the chromatin modification circuit model (Fig 1D)

3.1 Estimation of $\mu^{'}$

By analyzing experimental data available in the literature, it was possible to show that the parameter $\mu^{'}$ is small. In fact, in ES cells for example, *in vivo* experimental studies have shown that in the absence of DNMT3 ($k^1_{W0} = k^1_W = 0$) DNA methylation goes from 22% to 0.6% in 216 cell divisions [39], which corresponds to $\delta' + k^{'}_T = 0.024\delta$. This means that, in our model, $\beta\mu^{'} = \frac{\delta'+k^{'}_T}{\delta+\bar{k}^A_E} \leq \frac{\delta'+k^{'}_T}{\delta} = 0.024$. Now, let us rewrite explicitly β:

$$\beta = \frac{k^A_E D_{tot}}{\delta + \bar{k}^A_E}\frac{\delta' + k^{'}_T}{k^{'*}_T D_{tot}} = \frac{\frac{k^A_E D_{tot}}{\delta+\bar{k}^A_E}}{\frac{k^{'*}_T D_{tot}}{\delta'+k^{'}_T}}. \tag{141}$$

Then, assuming that the ratio between specifically recruited erasure rate constant and basal erasure rate constant has the same order of magnitude for any chromatin modification (that is the numerator and denominator of β have the same order of magnitude), we can conclude that, in this case, $\mu^{'} \leq 0.024$.

3.2 Model reduction of the 4D model

Before reducing the system (79), we define $c = \frac{\delta+\bar{k}^A_E}{k^A_E D_{tot}}$, $\mu = \frac{k^R_E}{k^A_E}$ with the constant b such that $bc\mu = \frac{\delta+\bar{k}^R_E}{k^A_E D_{tot}}$, $\mu^{'} = \frac{k^{'*}_T}{k^A_E}$ with the constant β such that $\beta c\mu^{'} = \frac{\delta'+k^{'}_T}{k^A_E D_{tot}}$. System (79) can thus be rewritten as follows:

$$\begin{aligned}
\dot{D}^R_1 &= (k^1_W + k^1_{W0} + k^{'}_M(D^R_2 + D^R_{12}))D + k^A_E D_{tot}\mu\left(bc + \frac{D^A}{D_{tot}}\right)D^R_{12} \\
&\quad - (k^2_{W0} + k_M(D^R_2 + D^R_{12}) + \bar{k}_M(D^R_1 + D^R_{12}) + k^A_E D_{tot}\mu^{'}\left(\beta c + \frac{D^A}{D_{tot}}\right))D^R_1 \\
\dot{D}^R_2 &= (k^2_W + k^2_{W0} + k_M(D^R_2 + D^R_{12}) + \bar{k}_M(D^R_1 + D^R_{12}))D + k^A_E D_{tot}\mu^{'}\left(\beta c + \frac{D^A}{D_{tot}}\right)D^R_{12} \\
&\quad - (k^1_{W0} + k^{'}_M(D^R_2 + D^R_{12}) + k^A_E D_{tot}\mu\left(bc + \frac{D^A}{D_{tot}}\right))D^R_2 \\
\dot{D}^R_{12} &= (k^1_{W0} + k^{'}_M(D^R_2 + D^R_{12}))D^R_2 + (k^2_{W0} + k_M(D^R_2 + D^R_{12}) + \bar{k}_M(D^R_1 + D^R_{12}))D^R_1 \\
&\quad - k^A_E D_{tot}(\mu\left(bc + \frac{D^A}{D_{tot}}\right) + \mu^{'}\left(\beta c + \frac{D^A}{D_{tot}}\right))D^R_{12} \\
\dot{D} &= k^A_E D_{tot}\left(\mu^{'}\left(\beta c + \frac{D^A}{D_{tot}}\right)D^R_1 + \mu\left(bc + \frac{D^A}{D_{tot}}\right)D^R_2 + \left(b^A + \frac{Y_1}{D_{tot}} + \frac{D^R_2 + D^R_{12}}{D_{tot}}\right)D^A\right) \\
&\quad - (k^2_W + k^2_{W0} + k_M(D^R_2 + D^R_{12}) + \bar{k}_M(D^R_1 + D^R_{12}) + k^1_W + k^1_{W0} + k^{'}_M(D^R_2 + D^R_{12}) + k^A_W + k^A_{W0} + k^A_M D^A)D \\
\dot{D}^A &= (k^A_W + k^A_{W0} + k^A_M D^A)D \\
&\quad - k^A_E D_{tot}\left(b^A + \frac{D^R_1 + D^R_{12}}{D_{tot}} + \frac{D^R_2 + D^R_{12}}{D_{tot}}\right)D^A,
\end{aligned} \tag{142}$$

with initial condition such that $D_{tot} = D + D^A + D^R_2 + D^R_1 + D^R_{12}$. In order to reduce the system, we consider the system in the parameter regime where $\epsilon^{'} \ll 1$, that is the specifically recruited erasure

reaction is slow compared to the auto and cross-catalysis reactions. Biophysically, in this regime, the reactions represented by the label $\mathrm{RE_i}$ in Fig 3A become slower compared to those represented by the label $\mathrm{C_i}$. In this regime, by reducing ϵ, also the reactions represented by the label $\mathrm{BE_i}$ in the diagram in Fig 3A become slower compared to the ones represented by the label $\mathrm{C_i}$. Specifically, this means that, whenever a nucleosome is characterized by either DNA methylation or H3K9me3 histone modification, it tends to acquire the other repressive modification much faster than loosing the modification that already characterizes it. Then we introduce the time variable $\bar{\tau} = tD_{tot}k_E^A$ and, in order to rewrite the model in terms of non-dimensional variables, we divide both sides of the ODEs (142) by D_{tot}, obtaining

$$\begin{aligned}
\frac{d\bar{D}_1^R}{d\bar{\tau}} &= \frac{k_W^1 + k_{W0}^1 + k_M^{'}(D_2^R + D_{12}^R)}{k_E^A D_{tot}}\bar{D} + \mu\left(bc + \bar{D}^A\right)\bar{D}_{12}^R \\
&- (\frac{k_{W0}^2 + k_M(D_2^R + D_{12}^R) + \bar{k}_M(D_1^R + D_{12}^R)}{k_E^A D_{tot}} + \mu^{'}\left(\beta c + \bar{D^A}\right))\bar{D}_1^R \\
\frac{d\bar{D}_2^R}{d\bar{\tau}} &= \frac{k_W^2 + k_{W0}^2 + k_M(D_2^R + D_{12}^R) + \bar{k}_M(D_1^R + D_{12}^R)}{k_E^A D_{tot}}\bar{D} + \mu^{'}\left(\beta c + \bar{D^A}\right)\bar{D}_{12}^R \\
&- (\frac{k_{W0}^1 + k_M^{'}(D_2^R + D_{12}^R)}{k_E^A D_{tot}} + \mu\left(bc + \bar{D^A}\right))\bar{D}_2^R \\
\frac{d\bar{D}_{12}^R}{d\bar{\tau}} &= \frac{k_{W0}^1 + k_M^{'}(D_2^R + D_{12}^R)}{k_E^A D_{tot}}\bar{D}_2^R + \frac{k_{W0}^2 + k_M(D_2^R + D_{12}^R) + \bar{k}_M(D_1^R + D_{12}^R)}{k_E^A D_{tot}}\bar{D}_1^R \\
&- (\mu\left(bc + \bar{D^A}\right) + \mu^{'}\left(\beta c + \bar{D^A}\right))\bar{D}_{12}^R \\
\frac{d\bar{D}}{d\bar{\tau}} &= \left(\mu^{'}\left(\beta c + \bar{D^A}\right)\bar{D}_1^R + \mu\left(bc + \bar{D^A}\right)\bar{D}_2^R + \left(c + (\bar{D}_1^R + \bar{D}_{12}^R) + (\bar{D}_2^R + \bar{D}_{12}^R)\right)\bar{D}^A\right) \\
&- \frac{(k_W^2 + k_{W0}^2 + k_M(D_2^R + D_{12}^R) + \bar{k}_M(D_1^R + D_{12}^R) + k_W^1 + k_{W0}^1 + k_M^{'}(D_2^R + D_{12}^R) + k_W^A + k_{W0}^A + k_M^A D^A)}{k_E^A D_{tot}}\bar{D} \\
\frac{d\bar{D}^A}{d\bar{\tau}} &= \frac{k_W^A + k_{W0}^A + k_M^A D^A}{k_E^A D_{tot}}\bar{D} - \left(c + (\bar{D}_1^R + \bar{D}_{12}^R) + (\bar{D}_2^R + \bar{D}_{12}^R)\right)\bar{D}^A,
\end{aligned} \tag{143}$$

in which each species is normalized with respect to D_{tot} ($\bar{X} := X/D_{tot}$). Furthermore, we rewrite the following terms as a function of $\epsilon^{'}$:

$$\begin{aligned}
&\frac{k_W^1 + k_{W0}^1 + k_M^{'}(D_2^R + D_{12}^R)}{k_E^A D_{tot}} = \frac{u_1^R + u_{10}^R + \alpha^{'}(\bar{D}_2^R + \bar{D}_{12}^R)}{\epsilon^{'}}, \\
&\frac{k_W^2 + k_{W0}^2 + k_M(D_2^R + D_{12}^R) + \bar{k}_M(D_1^R + D_{12}^R)}{k_E^A D_{tot}} = \frac{u_2^R + u_{20}^R + \alpha(\bar{D}_2^R + \bar{D}_{12}^R) + \bar{\alpha}(\bar{D}_1^R + \bar{D}_{12}^R)}{\epsilon^{'}}, \\
&\frac{k_W^A + k_{W0}^A + k_M^A D^A}{k_E^A D_{tot}} = \frac{u^A + u_0^A + \bar{D}^A}{\epsilon^{'}},
\end{aligned} \tag{144}$$

in which the final expressions are obtained by multiplying and dividing the first expressions by $\frac{k_M^A \mathrm{D_{tot}}}{\Omega}$ and all the parameters in the final formulas are defined as in system Main Text: Eqs (3). By substituting (144) in the equations (143), the ODE system (143) becomes

$$\begin{aligned}
\epsilon^{'}\frac{d\bar{D}^A}{d\bar{\tau}} &= (u^A + u_0^A + \bar{D}^A)\bar{D} - \epsilon^{'}\left(c + (\bar{D}_1^R + \bar{D}_{12}^R) + (\bar{D}_2^R + \bar{D}_{12}^R)\right)\bar{D}^A \\
\epsilon^{'}\frac{d\bar{D}_{12}^R}{d\bar{\tau}} &= (u_{10}^R + \alpha^{'}(\bar{D}_2^R + \bar{D}_{12}^R))\bar{D}_2^R + (u_{20}^R + \alpha(\bar{D}_2^R + \bar{D}_{12}^R) + \bar{\alpha}(\bar{D}_1^R + \bar{D}_{12}^R))\bar{D}_1^R
\end{aligned}$$

$$
\begin{aligned}
&- \epsilon^{'}(\mu \left(bc + \bar{D^A}\right) + \mu^{'} \left(\beta c + \bar{D^A}\right))\bar{D}^R_{12} \\
\epsilon^{'} \frac{d\bar{D}^R_1}{d\bar{\tau}} &= (u^R_1 + u^R_{10} + \alpha^{'}(\bar{D}^R_2 + \bar{D}^R_{12}))\bar{D} + \epsilon^{'}\mu \left(bc + \bar{D}^A\right) \bar{D}^R_{12} \\
&- ((u^R_{20} + \alpha(\bar{D}^R_2 + \bar{D}^R_{12}) + \bar{\alpha}(\bar{D}^R_1 + \bar{D}^R_{12})) + \epsilon^{'}\mu^{'} \left(\beta c + \bar{D^A}\right))\bar{D}^R_1 \qquad (145) \\
\epsilon^{'} \frac{d\bar{D}^R_2}{d\bar{\tau}} &= (u^R_2 + u^R_{20} + \alpha(\bar{D}^R_2 + \bar{D}^R_{12}) + \bar{\alpha}(\bar{D}^R_1 + \bar{D}^R_{12}))\bar{D} + \epsilon^{'}\mu^{'} \left(\beta c + \bar{D}^A\right) \bar{D}^R_{12} \\
&- ((u^R_{10} + \alpha^{'}(\bar{D}^R_2 + \bar{D}^R_{12})) + \epsilon^{'}\mu \left(bc + \bar{D^A}\right))\bar{D}^R_2 \\
\epsilon^{'} \frac{d\bar{D}}{d\bar{\tau}} &= \epsilon^{'} \left(\mu^{'} \left(\beta c + \bar{D^A}\right) \bar{D}^R_1 + \mu \left(bc + \bar{D^A}\right) \bar{D}^R_2 + \left(c + (\bar{D}^R_1 + \bar{D}^R_{12}) + (\bar{D}^R_2 + \bar{D}^R_{12})\right) \bar{D}^A\right) \\
&- (u^R_2 + u^R_{20} + \alpha(\bar{D}^R_2 + \bar{D}^R_{12}) + \bar{\alpha}(\bar{D}^R_1 + \bar{D}^R_{12}) + u^R_1 + u^R_{10} + \alpha^{'}(\bar{D}^R_2 + \bar{D}^R_{12}) + u^A + u^A_0 + \bar{D}^A)\bar{D}.
\end{aligned}
$$

It is possible to show that this system satisfies the conditions **C1**, **C2**, **C3** listed in Section 2.2 and hence that (145) is a *singular singularly perturbed system* [56]. In particular, defining

$$
x = \begin{pmatrix} \bar{D}^A \\ \bar{D}^R_{12} \end{pmatrix}, \quad y_2 = \begin{pmatrix} \bar{D}^R_1 \\ \bar{D}^R_2 \\ \bar{D} \end{pmatrix},
$$

$$
f_1 = \begin{pmatrix} (u^A + u^A_0 + \bar{D}^A)\bar{D} - \epsilon^{'} \left(c + (\bar{D}^R_1 + \bar{D}^R_{12}) + (\bar{D}^R_2 + \bar{D}^R_{12})\right) \bar{D}^A \\ \\ (u^R_{10} + \alpha^{'}(\bar{D}^R_2 + \bar{D}^R_{12}))\bar{D}^R_2 + (u^R_{20} + \alpha(\bar{D}^R_2 + \bar{D}^R_{12}) + \bar{\alpha}(\bar{D}^R_1 + \bar{D}^R_{12}))\bar{D}^R_1 \\ -\epsilon^{'}(\mu \left(bc + \bar{D^A}\right) + \mu^{'} \left(\beta c + \bar{D^A}\right))\bar{D}^R_{12} \end{pmatrix},
$$

$$
f_2 = \begin{pmatrix} (u^R_1 + u^R_{10} + \alpha^{'}(\bar{D}^R_2 + \bar{D}^R_{12}))\bar{D} + \epsilon^{'}\mu \left(bc + \bar{D}^A\right) \bar{D}^R_{12} \\ -((u^R_{20} + \alpha(\bar{D}^R_2 + \bar{D}^R_{12}) + \bar{\alpha}(\bar{D}^R_1 + \bar{D}^R_{12})) + \epsilon^{'}\mu^{'} \left(\beta c + \bar{D^A}\right))\bar{D}^R_1 \\ \\ (u^R_2 + u^R_{20} + \alpha(\bar{D}^R_2 + \bar{D}^R_{12}) + \bar{\alpha}(\bar{D}^R_1 + \bar{D}^R_{12}))\bar{D} + \epsilon^{'}\mu^{'} \left(\beta c + \bar{D}^A\right) \bar{D}^R_{12} \\ -((u^R_{10} + \alpha^{'}(\bar{D}^R_2 + \bar{D}^R_{12})) + \epsilon^{'}\mu \left(bc + \bar{D^A}\right))\bar{D}^R_2 \\ \\ \epsilon^{'} \left(\mu^{'} \left(\beta c + \bar{D^A}\right) \bar{D}^R_1 + \mu \left(bc + \bar{D^A}\right) \bar{D}^R_2 + \left(c + (\bar{D}^R_1 + \bar{D}^R_{12}) + (\bar{D}^R_2 + \bar{D}^R_{12})\right) \bar{D}^A\right) \\ -(u^R_2 + u^R_{20} + \alpha(\bar{D}^R_2 + \bar{D}^R_{12}) + \bar{\alpha}(\bar{D}^R_1 + \bar{D}^R_{12}) + u^R_1 + u^R_{10} + \alpha^{'}(\bar{D}^R_2 + \bar{D}^R_{12}) + u^A + u^A_0 + \bar{D}^A)\bar{D} \end{pmatrix}, \qquad (146)
$$

it is possible to show that $\phi(x) = (0, 0, 0)$ and that the matrix A as defined in (97) with $\bar{D} = \bar{D}^R_1 = \bar{D}^R_2 = 0$ and $\epsilon^{'} = 0$ can be written as follows:

$$
A(x) = \begin{pmatrix} 0_{2,2} & \bar{A}_{2,3} \\ 0_{3,2} & \bar{A}_{3,3} \end{pmatrix} \qquad (147)
$$

with

$$
\bar{A}_{2,3} = \begin{pmatrix} 0 & 0 & (u^A + u^A_0 + \bar{D}^A) \\ (u^R_{20} + (\alpha + \bar{\alpha})\bar{D}^R_{12}) & (u^R_{10} + \alpha^{'}\bar{D}^R_{12}) & \end{pmatrix} \qquad (148)
$$

$$\bar{A}_{3,3} = \begin{pmatrix} -(u_{20}^R + (\alpha+\bar{\alpha})\bar{D}_{12}^R) & 0 & (u_1^R + u_{10}^R + \alpha^{'}\bar{D}_{12}^R) \\ 0 & -(u_{10}^R + \alpha^{'}\bar{D}_{12}^R) & (u_2^R + u_{20}^R + (\alpha+\bar{\alpha})\bar{D}_{12}^R) \\ 0 & 0 & -(u^A + u_0^A + \bar{D}^A) \\ & & -(u_1^R + u_{10}^R + u_2^R + u_{20}^R + (\alpha+\bar{\alpha}+\alpha^{'})\bar{D}_{12}^R) \end{pmatrix}. \tag{149}$$

Matrix A has two zero eigenvalues and two corresponding linearly independent eigenvectors. Furthermore, the matrix B defined in (98) can be written as $\bar{A}_{3,3}$ and it has three eigenvalues always characterized by negative real part if $u_{10}^R, u_{20}^R, u_0^A \geq l$ with $l > 0$. To find the slow integral manifold and the reduced system defined in (96), we follow the procedure explained in Section 2.2: we first introduce in (145) the change of variable $\tilde{D} = \bar{D}/\epsilon^{'}$, $\tilde{D}_1^R = \bar{D}_1^R/\epsilon^{'}$ and $\tilde{D}_2^R = \bar{D}_2^R/\epsilon^{'}$, obtaining

$$\begin{aligned}
\epsilon^{'}\frac{d\tilde{D}_1^R}{d\bar{\tau}} &= (u_1^R + u_{10}^R + \alpha^{'}(\epsilon^{'}\tilde{D}_2^R + \bar{D}_{12}^R))\tilde{D} + \mu\left(bc + \bar{D}^A\right)\bar{D}_{12}^R \\
&\quad - ((u_{20}^R + \alpha(\epsilon^{'}\tilde{D}_2^R + \bar{D}_{12}^R) + \bar{\alpha}(\epsilon^{'}\tilde{D}_1^R + \bar{D}_{12}^R)) + \epsilon^{'}\mu^{'}\left(\beta c + \bar{D^A}\right))\tilde{D}_1^R \\
\epsilon^{'}\frac{d\tilde{D}_2^R}{d\bar{\tau}} &= (u_2^R + u_{20}^R + \alpha(\epsilon^{'}\tilde{D}_2^R + \bar{D}_{12}^R) + \bar{\alpha}(\epsilon^{'}\tilde{D}_1^R + \bar{D}_{12}^R))\tilde{D} + \mu^{'}\left(\beta c + \bar{D^A}\right)\bar{D}_{12}^R \\
&\quad - ((u_{10}^R + \alpha^{'}(\epsilon^{'}\tilde{D}_2^R + \bar{D}_{12}^R)) + \epsilon^{'}\mu\left(bc + \bar{D^A}\right))\tilde{D}_2^R \\
\epsilon^{'}\frac{d\tilde{D}}{d\bar{\tau}} &= \left(\mu^{'}\left(\beta c + \bar{D^A}\right)\epsilon^{'}\tilde{D}_1^R + \mu\left(bc + \bar{D^A}\right)\epsilon^{'}\tilde{D}_2^R + \left(c + (\epsilon^{'}\tilde{D}_1^R + \bar{D}_{12}^R) + (\epsilon^{'}\tilde{D}_2^R + \bar{D}_{12}^R)\right)\bar{D}^A\right) \\
&\quad - (u_2^R + u_{20}^R + \alpha(\epsilon^{'}\tilde{D}_2^R + \bar{D}_{12}^R) + \bar{\alpha}(\epsilon^{'}\tilde{D}_1^R + \bar{D}_{12}^R) + u_1^R + u_{10}^R + \alpha^{'}(\epsilon^{'}\tilde{D}_2^R + \bar{D}_{12}^R) + u^A + u_0^A + \bar{D}^A)\tilde{D} \\
\frac{d\bar{D}_{12}^R}{d\bar{\tau}} &= (u_{10}^R + \alpha^{'}(\epsilon^{'}\tilde{D}_2^R + \bar{D}_{12}^R))\tilde{D}_2^R + (u_{20}^R + \alpha(\epsilon^{'}\tilde{D}_2^R + \bar{D}_{12}^R) + \bar{\alpha}(\epsilon^{'}\tilde{D}_1^R + \bar{D}_{12}^R))\tilde{D}_1^R \\
&\quad - (\mu\left(bc + \bar{D^A}\right) + \mu^{'}\left(\beta c + \bar{D^A}\right))\bar{D}_{12}^R \\
\frac{d\bar{D}^A}{d\bar{\tau}} &= (u^A + u_0^A + \bar{D}^A)\tilde{D} - \left(c + (\epsilon^{'}\tilde{D}_1^R + \bar{D}_{12}^R) + (\epsilon^{'}\tilde{D}_2^R + \bar{D}_{12}^R)\right)\bar{D}^A.
\end{aligned} \tag{150}$$

It is important to point out that in the reduction we let $\epsilon = c\epsilon^{'}$, with $c = O(1)$, implying that as $\epsilon^{'}$ decreases also ϵ decreases. Given the definition of ϵ and $\epsilon^{'}$, it is reasonable that, if the specifically recruited erasure reaction is slow compared to the auto and cross-catalysis reactions, then also the basal erasure reaction due to non-specific binding of enzymes and dilution is slow compared to the auto and cross-catalysis reactions. This is consistent with the fact that the rates of enzymatic reactions where enzyme-substrate binding is highly specific tend to be larger than the rates of enzymatic reactions occurring though non-specific enzyme substrate binding and to removal due to simple dilution from cell growth.

Then, to calculate the slow integral manifold, let us construct the asymptotic expansion of $\tilde{D}$, $\tilde{D}_1^R$ and $\tilde{D}_2^R$:

$$\begin{aligned}
\tilde{D} &= h_0(\bar{D}^A, \bar{D}_{12}^R, \epsilon^{'}) = h_{00}(\bar{D}^A, \bar{D}_{12}^R) + \epsilon^{'}h_{01}(\bar{D}^A, \bar{D}_{12}^R) + O(\epsilon^{'2}), \\
\tilde{D}_1^R &= h_1(\bar{D}^A, \bar{D}_{12}^R, \epsilon^{'}) = h_{10}(\bar{D}^A, \bar{D}_{12}^R) + \epsilon^{'}h_{11}(\bar{D}^A, \bar{D}_{12}^R) + O(\epsilon^{'2}), \\
\tilde{D}_2^R &= h_2(\bar{D}^A, \bar{D}_{12}^R, \epsilon^{'}) = h_{20}(\bar{D}^A, \bar{D}_{12}^R) + \epsilon^{'}h_{21}(\bar{D}^A, \bar{D}_{12}^R) + O(\epsilon^{'2}).
\end{aligned} \tag{151}$$

Substituting (151) in the first three ODEs of (150), we obtain

$$
\begin{aligned}
\epsilon^{'}\frac{dh_1}{d\bar{\tau}} &= \epsilon^{'}(\frac{\partial h_1}{\partial \bar{D}^A}\frac{d\bar{D}^A}{d\bar{\tau}} + \frac{\partial h_1}{\partial \bar{D}^R_{12}}\frac{d\bar{D}^R_{12}}{d\bar{\tau}}) \\
&= (u^R_1 + u^R_{10} + \alpha^{'}(\epsilon^{'}h_2 + \bar{D}^R_{12}))h_0 + \mu\left(bc + \bar{D}^A\right)\bar{D}^R_{12} \\
&- ((u^R_{20} + \alpha(\epsilon^{'}h_2 + \bar{D}^R_{12}) + \bar{\alpha}(\epsilon^{'}h_1 + \bar{D}^R_{12})) + \epsilon^{'}\mu^{'}\left(\beta c + \bar{D^A}\right))h_1 \\
\epsilon^{'}\frac{dh_2}{d\bar{\tau}} &= \epsilon^{'}(\frac{\partial h_2}{\partial \bar{D}^A}\frac{d\bar{D}^A}{d\bar{\tau}} + \frac{\partial h_2}{\partial \bar{D}^R_{12}}\frac{d\bar{D}^R_{12}}{d\bar{\tau}}) \\
&= (u^R_2 + u^R_{20} + \alpha(\epsilon^{'}h_2 + \bar{D}^R_{12}) + \bar{\alpha}(\epsilon^{'}h_1 + \bar{D}^R_{12}))h_0 + \mu^{'}\left(\beta c + \bar{D^A}\right)\bar{D}^R_{12} \qquad (152) \\
&- ((u^R_{10} + \alpha^{'}(\epsilon^{'}h_2 + \bar{D}^R_{12})) + \epsilon^{'}\mu\left(bc + \bar{D^A}\right))h_2 \\
\epsilon^{'}\frac{dh_0}{d\bar{\tau}} &= \epsilon^{'}(\frac{\partial h_0}{\partial \bar{D}^A}\frac{d\bar{D}^A}{d\bar{\tau}} + \frac{\partial h_0}{\partial \bar{D}^R_{12}}\frac{d\bar{D}^R_{12}}{d\bar{\tau}}) \\
&= \left(\mu^{'}\left(\beta c + \bar{D^A}\right)\epsilon^{'}h_1 + \mu\left(bc + \bar{D^A}\right)\epsilon^{'}h_2 + \left(c + (\epsilon^{'}h_1 + \bar{D}^R_{12}) + (\epsilon^{'}h_2 + \bar{D}^R_{12})\right)\bar{D}^A\right) \\
&- (u^R_2 + u^R_{20} + \alpha(\epsilon^{'}h_2 + \bar{D}^R_{12}) + \bar{\alpha}(\epsilon^{'}h_1 + \bar{D}^R_{12}) + u^R_1 + u^R_{10} + \alpha^{'}(\epsilon^{'}h_2 + \bar{D}^R_{12}) + u^A + u^A_0 + \bar{D}^A)h_0.
\end{aligned}
$$

To calculate h_{i0} and h_{i1}, with $i = 0, 1, 2$, we equate the terms on the left and right hand side of the

equations multiplied by the same power of $\epsilon^{'}$, obtaining

$$h_{00} = \frac{(c+2\bar{D}^R_{12})\bar{D}^A}{u^R_2 + u^R_{20} + \alpha\bar{D}^R_{12} + \bar{\alpha}\bar{D}^R_{12} + u^R_1 + u^R_{10} + \alpha^{'}\bar{D}^R_{12} + u^A + u^A_0 + \bar{D}^A},$$

$$h_{10} = \frac{(u^R_1 + u^R_{10} + \alpha^{'}\bar{D}^R_{12})h_{00} + \mu\left(bc + \bar{D}^A\right)\bar{D}^R_{12}}{u^R_{20} + (\alpha+\bar{\alpha})\bar{D}^R_{12}},$$

$$h_{20} = \frac{(u^R_2 + u^R_{20} + \alpha\bar{D}^R_{12} + \bar{\alpha}\bar{D}^R_{12})h_{00} + \mu^{'}\left(\beta c + \bar{D^A}\right)\bar{D}^R_{12}}{u^R_{10} + \alpha^{'}\bar{D}^R_{12}},$$

$$\begin{aligned}
&(\frac{\partial h_{00}}{\partial \bar{D}^A}((u^A + u^A_0 + \bar{D}^A)h_{00} - (c+2\bar{D}^R_{12})\bar{D}^A) \\
&+ \frac{\partial h_{00}}{\partial \bar{D}^R_{12}}((u^R_{10} + \alpha^{'}\bar{D}^R_{12})h_{20} + (u^R_{20} + (\alpha+\bar{\alpha})\bar{D}^R_{12})h_{10} - (\mu\left(bc + \bar{D^A}\right) + \mu^{'}\left(\beta c + \bar{D^A}\right))\bar{D}^R_{12})) \\
&= \mu^{'}\left(\beta c + \bar{D^A}\right)h_{10} + \mu\left(bc + \bar{D^A}\right)h_{20} + (h_{10}+h_{20})\bar{D}^A - (\alpha^{'}h_{20} + (\alpha+\bar{\alpha})h_{10})h_{00} \\
&- (u^R_2 + u^R_{20} + \alpha\bar{D}^R_{12} + \bar{\alpha}\bar{D}^R_{12} + u^R_1 + u^R_{10} + \alpha^{'}\bar{D}^R_{12} + u^A + u^A_0 + \bar{D}^A)h_{01},
\end{aligned} \tag{153}$$

$$\begin{aligned}
&(\frac{\partial h_{10}}{\partial \bar{D}^A}((u^A + u^A_0 + \bar{D}^A)h_{00} - (c+2\bar{D}^R_{12})\bar{D}^A) \\
&+ \frac{\partial h_{10}}{\partial \bar{D}^R_{12}}((u^R_{10} + \alpha^{'}\bar{D}^R_{12})h_{20} + (u^R_{20} + (\alpha+\bar{\alpha})\bar{D}^R_{12})h_{10} - (\mu\left(bc + \bar{D^A}\right) + \mu^{'}\left(\beta c + \bar{D^A}\right))\bar{D}^R_{12})) \\
&= \alpha^{'}h_{20}h_{00} + (u^R_1 + u^R_{10} + \alpha^{'}\bar{D}^R_{12})h_{01} - (\alpha h_{20} + \bar{\alpha}h_{10})h_{10} - (u^R_{20} + (\alpha+\bar{\alpha})\bar{D}^R_{12})h_{11},
\end{aligned}$$

$$\begin{aligned}
&(\frac{\partial h_{20}}{\partial \bar{D}^A}((u^A + u^A_0 + \bar{D}^A)h_{00} - (c+2\bar{D}^R_{12})\bar{D}^A) \\
&+ \frac{\partial h_{20}}{\partial \bar{D}^R_{12}}((u^R_{10} + \alpha^{'}\bar{D}^R_{12})h_{20} + (u^R_{20} + (\alpha+\bar{\alpha})\bar{D}^R_{12})h_{10} - (\mu\left(bc + \bar{D^A}\right) + \mu^{'}\left(\beta c + \bar{D^A}\right))\bar{D}^R_{12})) \\
&= (\alpha h_{20} + \bar{\alpha}h_{10})h_{00} + (u^R_2 + u^R_{20} + (\alpha+\bar{\alpha})\bar{D}^R_{12})h_{01} - \alpha^{'}h^2_{20} - (u^R_{10} + \alpha^{'}\bar{D}^R_{12})h_{21}.
\end{aligned}$$

Since $\frac{\partial h_{i0}}{\partial \bar{D}^R_{12}}$ and $\frac{\partial h_{i0}}{\partial \bar{D}^A}$ are bounded for any $i = 0, 1, 2$ (that is, $\epsilon^{'}\frac{\partial h_{i0}}{\partial \bar{D}^R_{12}}, \epsilon^{'}\frac{\partial h_{i0}}{\partial \bar{D}^A} \ll 1$ for a sufficiently

small $\epsilon^{'}$), by solving (153) for $h_{00}, h_{10}, h_{20}, h_{01}, h_{11}, h_{21}$ we obtain

$$\begin{aligned}
h_{00} &= \frac{(c+2\bar{D}_{12}^R)\bar{D}^A}{u_2^R+u_{20}^R+\alpha\bar{D}_{12}^R+\bar{\alpha}\bar{D}_{12}^R+u_1^R+u_{10}^R+\alpha^{'}\bar{D}_{12}^R+u^A+u_0^A+\bar{D}^A}\\
h_{10} &= \frac{(u_1^R+u_{10}^R+\alpha^{'}\bar{D}_{12}^R)h_{00}+\mu\left(bc+\bar{D}^A\right)\bar{D}_{12}^R}{u_{20}^R+(\alpha+\bar{\alpha})\bar{D}_{12}^R}\\
h_{20} &= \frac{(u_2^R+u_{20}^R+\alpha\bar{D}_{12}^R+\bar{\alpha}\bar{D}_{12}^R)h_{00}+\mu^{'}\left(\beta c+\bar{D^A}\right)\bar{D}_{12}^R}{u_{10}^R+\alpha^{'}\bar{D}_{12}^R}\\
h_{01} &= \frac{(\mu(cb+\bar{D}^A)h_{20}+\mu^{'}(\beta c+\bar{D}^A)h_{10})}{u_2^R+u_{20}^R+\alpha\bar{D}_{12}^R+\bar{\alpha}\bar{D}_{12}^R+u_1^R+u_{10}^R+\alpha^{'}\bar{D}_{12}^R+u^A+u_0^A+\bar{D}^A}\\
h_{11} &= \frac{(u_1^R+u_{10}^R+\alpha^{'}\bar{D}_{12}^R)h_{01}-(\alpha h_{20}+\bar{\alpha}h_{10}+\mu^{'}(\beta c+\bar{D}^A))h_{10}}{u_{20}^R+(\alpha+\bar{\alpha})\bar{D}_{12}^R}\\
h_{21} &= \frac{(u_2^R+u_{20}^R+(\alpha+\bar{\alpha})\bar{D}_{12}^R)h_{01}-(\alpha^{'}h_{20}^2+\mu(bc+\bar{D}^A))h_{20}}{u_{10}^R+\alpha^{'}\bar{D}_{12}^R}.
\end{aligned} \tag{154}$$

Substituting $h_{00}, h_{01}, h_{10}, h_{11}, h_{20}$ and h_{21} into (151) and (151) into (150), we obtain the reduced system as follows:

$$\begin{aligned}
\frac{d\bar{D}^A}{d\bar{\tau}} &= \left(\frac{\epsilon^{'}(\mu(bc+\bar{D}^A)\mu^{'}(\beta c+\bar{D^A}))\bar{K}(u^A+u_0^A+\bar{D}^A)}{u^A+u_0^A+\bar{D}^A+u_R^2+u_{20}^R+u_1^R+u_{10}^R+(\alpha+\bar{\alpha}+\alpha^{'})\bar{D}_{12}^R}\right)\bar{D}_{12}^R\\
&\quad-\left(\frac{(c+2\bar{D}_{12}^R)(u_R^2+u_{20}^R+u_1^R+u_{10}^R+(\alpha+\bar{\alpha}+\alpha^{'})\bar{D}_{12}^R)}{u^A+u_0^A+\bar{D}^A+u_R^2+u_{20}^R+u_1^R+u_{10}^R+(\alpha+\bar{\alpha}+\alpha^{'})\bar{D}_{12}^R}\right)\bar{D}^A\\
\frac{d\bar{D}_{12}^R}{d\bar{\tau}} &= \left(\frac{(c+2\bar{D}_{12}^R)(u_R^2+u_{20}^R+u_1^R+u_{10}^R+(\alpha+\bar{\alpha}+\alpha^{'})\bar{D}_{12}^R)}{u^A+u_0^A+\bar{D}^A+u_R^2+u_{20}^R+u_1^R+u_{10}^R+(\alpha+\bar{\alpha}+\alpha^{'})\bar{D}_{12}^R}\right)\bar{D}^A\\
&\quad-\left(\frac{\epsilon^{'}(\mu(bc+\bar{D}^A)\mu^{'}(\beta c+\bar{D^A}))\bar{K}(u^A+u_0^A+\bar{D}^A)}{u^A+u_0^A+\bar{D}^A+u_R^2+u_{20}^R+u_1^R+u_{10}^R+(\alpha+\bar{\alpha}+\alpha^{'})\bar{D}_{12}^R}\right)\bar{D}_{12}^R,
\end{aligned} \tag{155}$$

with $\bar{K} = \frac{1}{u_{10}^R+\alpha^{'}\bar{D}_{12}^R} + \frac{1}{u_{20}^R+(\alpha+\bar{\alpha})\bar{D}_{12}^R}$. Summing the two ODEs written above, it is possible to notice that $\frac{d\bar{D}^A}{d\bar{\tau}} + \frac{d\bar{D}_{12}^R}{d\bar{\tau}} = 0$, that is $\bar{D}^A + \bar{D}_{12}^R =$ constant. In particular, since $\bar{D}^A + \bar{D}_{12}^R + \bar{D} + \bar{D}_1^R + \bar{D}_2^R = 1$ and $\bar{D} = \bar{D}_1^R = \bar{D}_2^R = 0$ for $\epsilon' = 0$, we have that $\bar{D}^A + \bar{D}_{12}^R = 1$, for $\epsilon' = 0$.

We further validate *via* simulation that system (155) is a proper reduction of the full system (145) when $\epsilon^{'}$ is small. We perform simulations of both system for different values of $\epsilon^{'}$ and initial conditions, showing that the trajectories of $\bar{D}_{12}^R$ and $\bar{D}^A$ of the full and reduced systems become closer as $\epsilon^{'}$ decreases (Fig J). Finally, multiplying both sides of the ODEs in (155) by $D_{tot}(k_E^A D_{tot})$,

system (155) can be rewritten in a dimensional way:

$$\begin{aligned}
\dot{D}^A &= \left(\frac{(\delta + \bar{k}_E^R + k_E^R D^A)(\delta' + k_T' + k_T'^* D^A)\bar{K}_{dim}(k_{W0}^A + k_W^A + k_M^A D^A)}{k_{W0}^A + k_W^A + k_M^A D^A + k_{W0}^2 + k_W^2 + k_{W0}^1 + k_W^1 + (k_M + \bar{k}_M + k_M')D_{12}^R}\right) D_{12}^R \\
&\quad - \left(\frac{(\delta + \bar{k}_E^A + 2k_E^A D_{12}^R)(k_{W0}^2 + k_W^2 + k_{W0}^1 + k_W^1 + (k_M + \bar{k}_M + k_M')D_{12}^R)}{k_{W0}^A + k_W^A + k_M^A D^A + k_{W0}^2 + k_W^2 + k_{W0}^1 + k_W^1 + (k_M + \bar{k}_M + k_M')D_{12}^R}\right) D^A \\
\dot{D}_{12}^R &= \left(\frac{(\delta + \bar{k}_E^A + 2k_E^A D_{12}^R)(k_{W0}^2 + k_W^2 + k_{W0}^1 + k_W^1 + (k_M + \bar{k}_M + k_M')D_{12}^R)}{k_{W0}^A + k_W^A + k_M^A D^A + k_{W0}^2 + k_W^2 + k_{W0}^1 + k_W^1 + (k_M + \bar{k}_M + k_M')D_{12}^R}\right) D^A \\
&\quad - \left(\frac{(\delta + \bar{k}_E^R + k_E^R D^A)(\delta' + k_T' + k_T'^* D^A)\bar{K}_{dim}(k_{W0}^A + k_W^A + k_M^A D^A)}{k_{W0}^A + k_W^A + k_M^A D^A + k_{W0}^2 + k_W^2 + k_{W0}^1 + k_W^1 + (k_M + \bar{k}_M + k_M')D_{12}^R}\right) D_{12}^R,
\end{aligned} \tag{156}$$

with $\bar{K}_{dim} = \frac{1}{k_{W0}^1 + k_M' D_{12}^R} + \frac{1}{k_{W0}^2 + (k_M + \bar{k}_M)D_{12}^R}$, or, since $D^R + D^A = D_{tot}$, it can be written as

$$\begin{aligned}
\dot{D}_{12}^R &= \left(\frac{(\delta + \bar{k}_E^A + 2k_E^A D_{12}^R)(k_{W0}^2 + k_W^2 + k_{W0}^1 + k_W^1 + (k_M + \bar{k}_M + k_M')D_{12}^R)}{k_{W0}^A + k_W^A + k_M^A(D_{tot} - D_{12}^R) + k_{W0}^2 + k_W^2 + k_{W0}^1 + k_W^1 + (k_M + \bar{k}_M + k_M')D_{12}^R}\right)(D_{tot} - D_{12}^R) \\
&\quad - \left(\frac{(\delta + \bar{k}_E^R + k_E^R(D_{tot} - D_{12}^R))(\delta' + k_T' + k_T'^*(D_{tot} - D^R))\bar{K}_{dim}(k_{W0}^A + k_W^A + k_M^A(D_{tot} - D_{12}^R))}{k_{W0}^A + k_W^A + k_M^A(D_{tot} - D_{12}^R) + k_{W0}^2 + k_W^2 + k_{W0}^1 + k_W^1 + (k_M + \bar{k}_M + k_M')D_{12}^R}\right) D_{12}^R.
\end{aligned} \tag{157}$$

The system is one-dimensional and it can be represented through the following simplified chemical reactions:

$$\begin{aligned}
&\mathrm{D^A} \xrightarrow{k_{AR}} \mathrm{D_{12}^R}, \quad k_{AR} = \left(\frac{(\delta + \bar{k}_E^A + 2k_E^A D_{12}^R)(k_{W0}^2 + k_W^2 + k_{W0}^1 + k_W^1 + (k_M + \bar{k}_M + k_M')D_{12}^R)}{k_{W0}^A + k_W^A + k_M^A D^A + k_{W0}^2 + k_W^2 + k_{W0}^1 + k_W^1 + (k_M + \bar{k}_M + k_M')D_{12}^R}\right), \\
&\mathrm{D_{12}^R} \xrightarrow{k_{RA}} \mathrm{D^A}, \quad k_{RA} = \left(\frac{(\delta + \bar{k}_E^R + k_E^R D^A)(\delta' + k_T' + k_T'^* D^A)\bar{K}_{dim}(k_{W0}^A + k_W^A + k_M^A D^A)}{k_{W0}^A + k_W^A + k_M^A D^A + k_{W0}^2 + k_W^2 + k_{W0}^1 + k_W^1 + (k_M + \bar{k}_M + k_M')D_{12}^R}\right).
\end{aligned} \tag{158}$$

3.3 Derivation of stationary probability distribution formula

Following the same procedure we used in Section 2.3 for system (115), the reduced chemical reaction system (158) can be represented by a one-dimensional Markov chain in which the state x represents the total number of $\mathrm{D_{12}^R}$, that is, $x = n_{12}^R$. In particular, the state x can vary between zero and $\mathrm{D_{tot}}$. Furthermore, the transition rate from state i to state j, $q_{i,j}$, for our Markov chain can be defined as follows:

$$\begin{aligned}
q_{x,(x+1)} &= \left(\frac{(\delta + \bar{k}_E^A + 2\frac{k_E^A}{\Omega}x)(k_{W0}^2 + k_W^2 + k_{W0}^1 + k_W^1 + \frac{(k_M + \bar{k}_M + k_M')}{\Omega}x)}{k_{W0}^A + k_W^A + \frac{k_M^A}{\Omega}(\mathrm{D_{tot}} - x) + k_{W0}^2 + k_W^2 + k_{W0}^1 + k_W^1 + \frac{(k_M + \bar{k}_M + k_M')}{\Omega}x}\right)(\mathrm{D_{tot}} - x), \\
q_{x,(x-1)} &= \left(\frac{(\delta + \bar{k}_E^R + \frac{k_E^R}{\Omega}(\mathrm{D_{tot}} - x))(\delta' + k_T' + \frac{k_T'^*}{\Omega}(\mathrm{D_{tot}} - x))\bar{K}_{dim}(k_{W0}^A + k_W^A + \frac{k_M^A}{\Omega}(\mathrm{D_{tot}} - x))}{k_{W0}^A + k_W^A + \frac{k_M^A}{\Omega}(\mathrm{D_{tot}} - x) + k_{W0}^2 + k_W^2 + k_{W0}^1 + k_W^1 + \frac{(k_M + \bar{k}_M + k_M')}{\Omega}x}\right)x,
\end{aligned} \tag{159}$$

in which we have used $D^A = n^A/\Omega$ and $D_{12}^R = n_{12}^R/\Omega$, with Ω the volume of the reactions and $\bar{K}_{dim} = \frac{1}{k_{W0}^1 + \frac{k_M^{'}}{\Omega}x} + \frac{1}{k_{W0}^2 + \frac{(k_M + \bar{k}_M)}{\Omega}x}$, for $x \in [0, \mathrm{D_{tot}}]$ [49]. Since this Markov chain is irreducible and reversible, we can apply detailed balance [60] to determine an analytical expression for the stationary probability distribution $\pi(x)$ as a function of $\pi(0)$:

$$\pi(x) = \prod_{i=1}^{x} \frac{q_{(i-1),i}}{q_{i,(i-1)}} \pi(0). \tag{160}$$

Now, introducing

$$\bar{K}_j = \frac{1}{u_{10}^R + \alpha' \frac{j}{\mathrm{D_{tot}}}} + \frac{1}{u_{20}^R + (\alpha + \bar{\alpha}) \frac{j}{\mathrm{D_{tot}}}}, \tag{161}$$

let us compute $\prod_{i=1}^{x} \frac{q_{(i-1),i}}{q_{i,(i-1)}}$ for our system by writing explicitly the expression for the transition rates and rearranging properly the terms. In particular, for any $x \in [1, \mathrm{D_{tot}} - 1]$, $\prod_{i=1}^{x} \frac{q_{(i-1),i}}{q_{i,(i-1)}}$ in (160) can be written as

$$\begin{aligned}
\prod_{i=1}^{x} \frac{q_{(i-1),i}}{q_{i,(i-1)}} =& \binom{\mathrm{D_{tot}}}{x} \left(1 + x \frac{\frac{k_M}{\Omega} + \frac{\bar{k}_M}{\Omega} + \frac{k_M^{'}}{\Omega} - \frac{k_M^A}{\Omega}}{\frac{k_M^A}{\Omega}\mathrm{D_{tot}} + k_{W0}^A + k_W^A + k_{W0}^1 + k_W^1 + k_{W0}^2 + k_W^2} \right) \cdot \\
& \cdot \prod_{i=1}^{x-1} \left(\frac{(\delta + \bar{k}_E^A + 2\frac{k_E^A}{\Omega} i)(k_{W0}^2 + k_W^2 + k_{W0}^1 + k_W^1 + \frac{(k_M + \bar{k}_M + k_M^{'})}{\Omega} i)}{(\delta + \bar{k}_E^R + \frac{k_E^R}{\Omega}(\mathrm{D_{tot}} - i))(\delta' + k_T^{'} + \frac{k_T^{'*}}{\Omega}(\mathrm{D_{tot}} - i))\bar{K}_{dim_i}(k_{W0}^A + k_W^A + \frac{k_M^A}{\Omega}(\mathrm{D_{tot}} - i))} \right) \cdot \\
& \cdot \frac{(\delta + \bar{k}_E^A)(k_{W0}^2 + k_W^2 + k_{W0}^1 + k_W^1)}{(\delta + \bar{k}_E^R + \frac{k_E^R}{\Omega}(\mathrm{D_{tot}} - x))(\delta' + k_T^{'} + \frac{k_T^{'*}}{\Omega}(\mathrm{D_{tot}} - x))\bar{K}_{dim_x}(k_{W0}^A + k_W^A + \frac{k_M^A}{\Omega}(\mathrm{D_{tot}} - x))} \\
=& \binom{\mathrm{D_{tot}}}{x} \left(1 + \frac{x}{\mathrm{D_{tot}}} \frac{(\alpha + \bar{\alpha} + \alpha') - 1}{1 + u_0^A + u^A + u_{10}^R + u_1^R + u_{20}^R + u_R^2} \right) \cdot \\
& \cdot \prod_{i=1}^{x-1} \left(\frac{(\epsilon + 2\epsilon' \frac{i}{\mathrm{D_{tot}}})(u_{10}^R + u_1^R + u_{20}^R + u_R^2 + (\alpha + \bar{\alpha} + \alpha') \frac{i}{\mathrm{D_{tot}}})}{\mu(b\epsilon + \epsilon' \frac{(\mathrm{D_{tot}} - i)}{\mathrm{D_{tot}}})\mu'(\beta\epsilon + \epsilon' \frac{(\mathrm{D_{tot}} - i)}{\mathrm{D_{tot}}})\bar{K}_i(u_0^A + u^A + \frac{(\mathrm{D_{tot}} - i)}{\mathrm{D_{tot}}})} \right) \cdot \\
& \cdot \frac{\epsilon(u_{10}^R + u_1^R + u_{20}^R + u_R^2)}{\mu(b\epsilon + \epsilon' \frac{(\mathrm{D_{tot}} - x)}{\mathrm{D_{tot}}})\mu'(\beta\epsilon + \epsilon' \frac{(\mathrm{D_{tot}} - x)}{\mathrm{D_{tot}}})\bar{K}_x(u_0^A + u^A + \frac{(\mathrm{D_{tot}} - x)}{\mathrm{D_{tot}}})},
\end{aligned} \tag{162}$$

in which the final expression has been obtained by dividing numerator and denominator by $(\frac{k_M^A \mathrm{D_{tot}}}{\Omega})$ in each of the factors. For $x = \mathrm{D_{tot}}$ expression (162) can be written as

$$\prod_{i=1}^{\mathrm{D_{tot}}} \frac{q_{(i-1),i}}{q_{i,(i-1)}} = \left(1 + x\frac{\frac{k_M}{\Omega} + \frac{\bar{k}_M}{\Omega} + \frac{k_M^{'}}{\Omega} - \frac{k_M^A}{\Omega}}{\frac{k_M^A}{\Omega}\mathrm{D_{tot}} + k_{W0}^A + k_W^A + k_{W0}^1 + k_W^1 + k_{W0}^2 + k_W^2}\right)\cdot$$
$$\cdot \prod_{i=1}^{\mathrm{D_{tot}}-1} \left(\frac{(\delta + \bar{k}_E^A + 2\frac{k_E^A}{\Omega}i)(k_{W0}^2 + k_W^2 + k_{W0}^1 + k_W^1 + \frac{(k_M + \bar{k}_M + k_M^{'})}{\Omega}i)}{(\delta + \bar{k}_E^R + \frac{k_E^R}{\Omega}(\mathrm{D_{tot}} - i))(\delta' + k_T^{'} + \frac{k_T^{'*}}{\Omega}(\mathrm{D_{tot}} - i))\bar{K}_{dim_{\mathrm{D_{tot}}}}(k_{W0}^A + k_W^A + \frac{k_M^A}{\Omega}(\mathrm{D_{tot}} - i))}\right)\cdot$$
$$\cdot \frac{(\delta + \bar{k}_E^A)(k_{W0}^2 + k_W^2 + k_{W0}^1 + k_W^1)}{(\delta + \bar{k}_E^R)(\delta' + k_T^{'})\bar{K}_{dim_i}(k_{W0}^A + k_W^A)}$$
$$= \left(1 + \frac{(\alpha + \bar{\alpha} + \alpha^{'}) - 1}{1 + u_0^A + u^A + u_{10}^R + u_1^R + u_{20}^R + u_R^2}\right)\cdot$$
$$\cdot \prod_{i=1}^{\mathrm{D_{tot}}-1} \left(\frac{(\epsilon + 2\epsilon^{'}\frac{i}{\mathrm{D_{tot}}})(u_{10}^R + u_1^R + u_{20}^R + u_R^2 + (\alpha + \bar{\alpha} + \alpha^{'})\frac{i}{\mathrm{D_{tot}}})}{\mu(b\epsilon + \epsilon^{'}\frac{(\mathrm{D_{tot}}-i)}{\mathrm{D_{tot}}})\mu'(\beta\epsilon + \epsilon^{'}\frac{(\mathrm{D_{tot}}-i)}{\mathrm{D_{tot}}})\bar{K}_i(u_0^A + u^A + \frac{(\mathrm{D_{tot}}-i)}{\mathrm{D_{tot}}})}\right)\cdot$$
$$\cdot \frac{\epsilon(u_{10}^R + u_1^R + u_{20}^R + u_R^2)}{\mu(b\epsilon)\mu'(\beta\epsilon)\bar{K}_{\mathrm{D_{tot}}}(u_0^A + u^A)}, \tag{163}$$

in which the final expression has been obtained by dividing numerator and denominator by $(\frac{k_M^A \mathrm{D_{tot}}}{\Omega})$ in each of the factors. Now, in order to find an expression for $\pi(0)$, we use $\sum_{j=0}^{\mathrm{D_{tot}}} \pi(j) = \sum_{j=1}^{\mathrm{D_{tot}}} \left(\prod_{i=1}^{j} \frac{q_{(i-1),i}}{q_{i,(i-1)}}\right) \pi(0)$ $+\pi(0) = 1$ and then we can express $\pi(0)$ as follows:

$$\pi(0) = \frac{1}{\left(1 + \sum_{j=1}^{\mathrm{D_{tot}}} \left(\prod_{i=1}^{j} \frac{q_{(i-1),i}}{q_{i,(i-1)}}\right)\right)}. \tag{164}$$

Substituting in (160) the $\pi(0)$ expression obtained in (164), the stationary probability $\pi(x)$ can finally be expressed as

$$\pi(x) = \prod_{i=1}^{x} \frac{q_{(i-1),i}}{q_{i,(i-1)}}\pi(0) = \frac{\prod_{i=1}^{x} \frac{q_{(i-1),i}}{q_{i,(i-1)}}}{\left(1 + \sum_{j=1}^{\mathrm{D_{tot}}} \left(\prod_{i=1}^{j} \frac{q_{(i-1),i}}{q_{i,(i-1)}}\right)\right)}, \tag{165}$$

for $x \in [1, \mathrm{D_{tot}}]$. Then, assuming that $\epsilon^{'} \neq 0$, comparing (162) and (163) it is possible to notice that in the condition $\epsilon \ll 1$, $\prod_{i=1}^{j} \frac{q_{(i-1),i}}{q_{i,(i-1)}} \ll \prod_{i=1}^{\mathrm{D_{tot}}} \frac{q_{(i-1),i}}{q_{i,(i-1)}}$ for any $j \in [1, \mathrm{D_{tot}} - 1]$. This implies that, when $\epsilon \ll 1$, $\sum_{j=0}^{\mathrm{D_{tot}}} \pi(j) = 1$ can be approximated as follows:

$$1 = \sum_{j=0}^{\mathrm{D_{tot}}} \pi(j) = \left[\sum_{j=1}^{\mathrm{D_{tot}}} \left(\prod_{i=1}^{j} \frac{q_{(i-1),i}}{q_{i,(i-1)}}\right)\right] \pi(0) + \pi(0) \approx \prod_{i=1}^{\mathrm{D_{tot}}} \frac{q_{(i-1),i}}{q_{i,(i-1)}}\pi(0) + \pi(0) \tag{166}$$

from which

$$\pi(0) \approx \frac{1}{1 + \prod_{i=1}^{\mathrm{D_{tot}}} \frac{q_{(i-1),i}}{q_{i,(i-1)}}}, \tag{167}$$

and, from equation (165),

$$\pi(\mathrm{D_{tot}}) \approx \frac{\prod_{i=1}^{\mathrm{D_{tot}}} \frac{q_{(i-1),i}}{q_{i,(i-1)}}}{1 + \prod_{i=1}^{\mathrm{D_{tot}}} \frac{q_{(i-1),i}}{q_{i,(i-1)}}}. \tag{168}$$

Then, the stationary probability is such that, under the condition $\epsilon \ll 1$, it can be expressed as follows:

$$\pi_{\epsilon \ll 1}(x) \approx \begin{cases} \frac{1}{1+P} & \text{if } x = 0 \\ 0 & \text{if } x \neq 0, \mathrm{D_{tot}} \\ \frac{P}{1+P} & \text{if } x = \mathrm{D_{tot}} \end{cases} \tag{169}$$

with

$$\begin{aligned} P = &\frac{(u_0^A + u^A + u_{10}^R + u_1^R + u_{20}^R + u_R^2 + \alpha + \bar{\alpha} + \alpha')}{(u_0^A + u^A + u_{10}^R + u_1^R + u_{20}^R + u_R^2 + 1)} \cdot \\ &\cdot \prod_{i=1}^{\mathrm{D_{tot}}-1} \left(\frac{2(u_{10}^R + u_1^R + u_{20}^R + u_R^2 + (\alpha + \bar{\alpha} + \alpha')\frac{i}{\mathrm{D_{tot}}})}{\mu\mu'\epsilon'\frac{(\mathrm{D_{tot}}-i)}{\mathrm{D_{tot}}}\bar{K}_i(u_0^A + u^A + \frac{(\mathrm{D_{tot}}-i)}{\mathrm{D_{tot}}})} \right) \cdot \frac{(u_{10}^R + u_1^R + u_{20}^R + u_R^2)}{\mu\mu' b\beta\epsilon\bar{K}_{\mathrm{D_{tot}}}(u_0^A + u^A)}. \end{aligned} \tag{170}$$

with

$$\bar{K}_{\mathrm{D_{tot}}} = \frac{1}{u_{10}^R + \alpha'} + \frac{1}{u_{20}^R + (\alpha + \bar{\alpha})}. \tag{171}$$

3.4 Derivation of time to memory loss formula for the 4D model

Now, in order to determine the time to memory loss of the fully repressed gene state, $\tau^0_{\mathrm{D_{tot}}}$, that is, the expected value of the first time at which the state x hits 0, starting from $x = \mathrm{D_{tot}}$ we can use the formula (136) derived in Section 2.4, that is

$$\tau^0_{\mathrm{D_{tot}}} = \frac{r_{\mathrm{D_{tot}}-1}}{\gamma_{\mathrm{D_{tot}}}} \left(1 + \sum_{i=1}^{\mathrm{D_{tot}}-1} \frac{1}{r_i}\right) + \frac{1}{\gamma_1} + \sum_{i=2}^{\mathrm{D_{tot}}-1} \left[\frac{r_{i-1}}{\gamma_i} \left(1 + \sum_{j=1}^{i-1} \frac{1}{r_j}\right) \right], \tag{172}$$

with $r_j = \frac{\alpha_1 \alpha_2 \ldots \alpha_j}{\gamma_1 \gamma_2 \ldots \gamma_j}$. In particular, for the Markov chain associated with the one-dimensional reduced model of the chromatin modification system, introducing $\bar{K}_i = \frac{1}{u_{10}^R + \alpha' \frac{i}{\mathrm{D_{tot}}}} + \frac{1}{u_{20}^R + (\alpha + \bar{\alpha})\frac{i}{\mathrm{D_{tot}}}}$, α_i and γ_i are given by

$$\begin{aligned} \alpha_i &= \left(\frac{(\delta + \bar{k}_E^A + 2\frac{k_E^A}{\Omega} i)(k_{W0}^2 + k_W^2 + k_{W0}^1 + k_W^1 + \frac{(k_M + \bar{k}_M + k_M')}{\Omega} i)}{k_{W0}^A + k_W^A + \frac{k_M^A}{\Omega}(\mathrm{D_{tot}} - i) + k_{W0}^2 + k_W^2 + k_{W0}^1 + k_W^1 + \frac{(k_M + \bar{k}_M + k_M')}{\Omega} i} \right) (\mathrm{D_{tot}} - i) \\ &= \left(\frac{(\epsilon + 2\epsilon' \frac{i}{\mathrm{D_{tot}}})(k_{W0}^2 + k_W^2 + k_{W0}^1 + k_W^1 + \frac{(k_M + \bar{k}_M + k_M')}{\Omega} i)}{u_0^A + u^A + \frac{(\mathrm{D_{tot}}-i)}{\mathrm{D_{tot}}} + u_{20}^R + u_2^R + u_{10}^R + u_1^R + (\alpha + \bar{\alpha} + \alpha')\frac{i}{\mathrm{D_{tot}}}} \right) (\mathrm{D_{tot}} - i) \\ \gamma_i &= \left(\frac{(\delta + \bar{k}_E^R + \frac{k_E^R}{\Omega}(\mathrm{D_{tot}} - i))(\delta' + k_T' + \frac{k_T'^*}{\Omega}(\mathrm{D_{tot}} - i))\bar{K}_i(k_{W0}^A + k_W^A + \frac{k_M^A}{\Omega}(\mathrm{D_{tot}} - i))}{k_{W0}^A + k_W^A + \frac{k_M^A}{\Omega}(\mathrm{D_{tot}} - i) + k_{W0}^2 + k_W^2 + k_{W0}^1 + k_W^1 + \frac{(k_M + \bar{k}_M + k_M')}{\Omega} i} \right) i \\ &= \left(\frac{\mu(b\epsilon + \epsilon' \frac{(\mathrm{D_{tot}}-i)}{\mathrm{D_{tot}}})\mu'(\beta\epsilon + \epsilon' \frac{(\mathrm{D_{tot}}-i)}{\mathrm{D_{tot}}})\bar{K}_i(k_{W0}^A + k_W^A + \frac{k_M^A}{\Omega}(\mathrm{D_{tot}} - i))}{u_0^A + u^A + \frac{(\mathrm{D_{tot}}-i)}{\mathrm{D_{tot}}} + u_{20}^R + u_2^R + u_{10}^R + u_1^R + (\alpha + \bar{\alpha} + \alpha')\frac{i}{\mathrm{D_{tot}}}} \right) i, \end{aligned} \tag{173}$$

in which the final expressions are obtained by multiplying and dividing the intermediate formula by $\frac{k_M^A \mathrm{D_{tot}}}{\Omega}$. Also in this case, in the absence of external input stimuli and assuming that $\epsilon' \neq 0$, it is possible to notice that, for $\epsilon \ll 1$, the dominant term of $\tau^0_{\mathrm{D_{tot}}}$ is the first addend in (172). Then, by

normalizing the time to memory loss with respect $\frac{k_M^A \mathrm{D_{tot}}}{\Omega}$ ($\bar{\tau}^0_{\mathrm{D_{tot}}} = \tau^0_{\mathrm{D_{tot}}} \frac{k_M^A \mathrm{D_{tot}}}{\Omega}$), $\tau^0_{\mathrm{D_{tot}}}$ in the regime $\epsilon \ll 1$ can be re-written as follows:

$$\bar{\tau}^0_{\mathrm{D_{tot}}} = \bar{\tau}_R \approx \frac{K_R}{\mu\mu^{'}\epsilon^2}\left(1 + \sum_{i=1}^{\mathrm{D_{tot}}-1} \frac{K_R^i}{h_1^i(\mu\mu^{'})}\right), \tag{174}$$

with h_1^i an increasing function, $h_1^i(0) = 0$, K_R and K_R^i functions independent of ϵ, $\mu^{'}$ and μ, and in which we redefine $\bar{\tau}^0_{\mathrm{D_{tot}}}$ as $\bar{\tau}_R$ to simplify the notation.

Similarly we can determine the time to memory loss of the fully activated gene state, $\tau_0^{\mathrm{D_{tot}}}$, by using the formula (139) derived in Section 2.4, that is

$$\tau_0^{\mathrm{D_{tot}}} = \frac{\tilde{r}_{\mathrm{D_{tot}}-1}}{\alpha_0}\left(1 + \sum_{j=1}^{\mathrm{D_{tot}}-1} \frac{1}{\tilde{r}_i}\right) + \frac{1}{\alpha_{\mathrm{D_{tot}}-1}} + \sum_{i=2}^{\mathrm{D_{tot}}-1}\left[\frac{\tilde{r}_{i-1}}{\alpha_{\mathrm{D_{tot}}-i}}\left(1 + \sum_{j=1}^{i-1} \frac{1}{\tilde{r}_j}\right)\right], \tag{175}$$

with α_i and γ_i as defined in (173) and $\tilde{r}_j = \frac{\gamma_{\mathrm{D_{tot}}-1}\gamma_{\mathrm{D_{tot}}-2}...\gamma_{\mathrm{D_{tot}}-j}}{\alpha_{\mathrm{D_{tot}}-1}\alpha_{\mathrm{D_{tot}}-2}...\alpha_{\mathrm{D_{tot}}-j}}$, $\tau_0^{\mathrm{D_{tot}}}$. In the absence of external input stimuli and assuming that $\epsilon^{'} \neq 0$, it is possible to notice that, for $\epsilon \ll 1$, the dominant term of $\tau_0^{\mathrm{D_{tot}}}$ is the first addend in (175). Then, by normalizing the time to memory loss with respect $\frac{k_M^A \mathrm{D_{tot}}}{\Omega}$ ($\bar{\tau}_0^{\mathrm{D_{tot}}} = \tau_0^{\mathrm{D_{tot}}} \frac{k_M^A \mathrm{D_{tot}}}{\Omega}$), $\tau_0^{\mathrm{D_{tot}}}$ in the regime $\epsilon \ll 1$ can be re-written as follows:

$$\bar{\tau}_0^{\mathrm{D_{tot}}} = \bar{\tau}_A \approx \frac{K_A}{\epsilon}\left(1 + \sum_{i=1}^{\mathrm{D_{tot}}-1} \frac{h_2^i(\mu\mu^{'})}{K_A^i}\right), \tag{176}$$

with h_2^i an increasing function, $h_2^i(0) = 0$, K_A and K_A^i functions independent of ϵ, $\mu^{'}$ and μ, and in which we redefine $\bar{\tau}_0^{\mathrm{D_{tot}}}$ as $\bar{\tau}_A$ to simplify the notation.

3.5 Effect of $\epsilon^{'}$ on the stationary distributions

From Figs Q-T it is possible to notice that the parameter $\epsilon^{'}$ does not substantially affect the trend with which ϵ, $\mu^{'}$, μ and the inputs affect the distribution. If $\epsilon^{'} \gg 1$, the only states characterized by high probability are the ones with either activating or repressive marks. The reason is that in the parameter regime $\epsilon^{'} \gg 1$ (that is the specifically recruited erasure reaction is fast compared to the auto and cross-catalysis reactions), since the recruited erasure is very fast, the system tends to reach a state in which the activating marks erased completely the repressive marks or *viceversa* (the states are on the axes). When $\epsilon^{'}$ decreases compared to ϵ, the peaks of the distribution become less concentrated and in the extreme case where $\epsilon' \ll \epsilon$, the distribution becomes unimodal (Fig U)

4 Derivation of the transcriptional regulation model

Transcription from nucleosome state $\mathrm{D^R}$. H3K9me3 is recognized and bound to by the chromodomain of the HP1α protein, which once bound contributes to nucleosomal compaction [5](Chapter 3) through a mechanism where HP1α dimerizes and cross-links nucleosomes [5](Chapter 1),[62]. Concerning DNA methylation, it has been shown that it is essential for cell differentiation and embryonic development, playing an important role in mediating gene expression [63]. In particular, some studies have been conducted in order to determine the DNA methylation level in the region (1kb-5kb window) around the transcription start sites (TSS), showing a high correlation between the level of methylated CpG and the gene repression [64]. Consistent with these observations, in

our model we consider D_1^R, D_2^R and D_{12}^R (nucleosome with repressive modifications that lead to a more compacted structure of the chromatin) to be inaccessible to general TFs, and thus to be transcriptionally "off" (silent).

Transcription from nucleosome state $\mathrm{D^A}$. Transcriptional activators recruit remodeling complexes, such as the SWI/SNF complex, which become activated by the binding of their bromodomain with acetylated lysines. These remodelers open up nucleosomes for RNA pol II and basal TFs to bind and initiate transcription and then transcript elongation [5](Chapter 21), [1](Chapters 3-5). Histone acetylation is essential for transcriptional machinery to make its way on the DNA despite the presence of nucleosomes. Also, H3K4me3 can interact with TFIID complex, which is implicated in the recruitment of transcriptional machinery [14](Chapter 11), thus potentially enhancing transcription rate. Therefore, the DNA wrapped around a nucleosome with activating histone modification, $\mathrm{D^A}$, will be transcribed by the basal transcriptional machinery as it is largely accessible to it. Enhanced transcription from $\mathrm{D^A}$ will occur if A_n binds, creating the complex $\mathrm{\bar{C}_A^A}$, and recruits to it the basal transcriptional machinery more efficiently [1](Chapter 4).

Transcription from nucleosome state D. Nucleosome state D can also allow some (possibly zero) basal transcription, lower than the one obtained by $\mathrm{D^A}$. In fact D is missing repressive histone marks and, although not as accessible to basal transcriptional machinery and remodeling factors as $\mathrm{D^A}$, it can in principle still allow for non-specific targeting by chromatin remodelers, many of which contain DNA-binding domains [65]. This enables the formation of the pre-initiation complex and consequent transcriptional initiation and elongation [1]. However, it has been shown that, once RNA Pol II initiates transcription it can recruit SETs, which methylate H3K4 [15], which in turn can further recruit HATs by the Thryotorax complex [8]. This promotes downstream acetylation and hence further recruitment of remodeling complexes, thus allowing transcriptional elongation to proceed [5](Chapter 21). This implies that transcription by RNA Pol II of DNA wrapped around an unmodified nucleosome, D, occurs concurrently with the deposition of H3K4me3 and hence the conversion of D to $\mathrm{D^A}$. We capture this in our model by allowing protein production only by the DNA wrapped around a nucleosome with activating histone modification, $\mathrm{D^A}$.

In light of these observations, we can write the binding reactions related to $\mathrm{\bar{C}_R^A}$ and $\mathrm{\bar{C}_R^A}$ as follows:

$$\mathrm{A}_n + \mathrm{D^A} \underset{\bar{d}_A^{'}}{\overset{\bar{a}_A^{'}}{\rightleftharpoons}} \mathrm{\bar{C}_A^A}, \quad \mathrm{R}_n + \mathrm{D^A} \underset{\bar{d}_R^{'}}{\overset{\bar{a}_R^{'}}{\rightleftharpoons}} \mathrm{\bar{C}_R^A}, \tag{177}$$

in which $\bar{a}_A^{'}$, $\bar{a}_R^{'}$, and $\bar{d}_A^{'}$, $\bar{d}_R^{'}$. Furthermore, denoting the expressed protein with X and lumping for simplicity transcription and translation in a one-step reaction, we can write the set of gene expression reactions as follows:

$$\mathrm{D^A} \xrightarrow{\alpha_b^A} \mathrm{D^A} + \mathrm{X},\ \mathrm{\bar{C}_A^A} \xrightarrow{\alpha_b^A + \bar{\alpha}_b^A} \mathrm{\bar{C}_A^A} + \mathrm{X},\ \mathrm{D^A} \xrightarrow{\alpha_b^A} \mathrm{D^A} + \mathrm{X},\ \mathrm{\bar{C}_R^A} \xrightarrow{\alpha_b^A} \mathrm{\bar{C}_R^A} + \mathrm{X}, \tag{178}$$

in which α_b^A and $\bar{\alpha}_b^A$ are the basal and the active protein production rate constants. The ODE for X is then given by

$$\dot{X} = \alpha_b^A(D^A + \bar{C}_A^A + \bar{C}_R^A) + \bar{\alpha}_b^A \bar{C}_A^A + \alpha_b^A \bar{C}_R^A - \gamma_x X \tag{179}$$

in which γ_x represent the rate of degradation and dilution of X. Now, let us introduce D_{tot}^A, which is the total concentration of nucleosome characterized by activating histone modification, free or bound by A_n or R_n ($D_{tot}^A = D^A + \bar{C}_A^A + \bar{C}_R^A$), $K^A = \frac{d^A}{a^A}$ and $K^R = \frac{d^R}{a^R}$ as the dissociation constants of the first reactions in (7) and (9), respectively, $K_A^A = \frac{\bar{d}_A^{'}}{\bar{a}_A^{'}}$ and $K_R^A = \frac{\bar{d}_A^{'}}{\bar{a}_A^{'}}$ as the dissociation constants of the reactions in (177). Then, considering as before the binding reactions much faster

than the other reactions and then setting the complexes dynamics to the QSS ($\bar{C}_A^A = \frac{A_n D^A}{K_A^A}$ and $\bar{C}_R^A = \frac{R_n D^A}{K_R^A}$), (179) becomes

$$\dot{X} = (\alpha_b^A + \frac{\bar{\alpha}_b^A \frac{A^n}{K_A K_A^A}}{1 + \frac{A^n}{K_A K_A^A} + \frac{R^n}{K_R K_R^A}})D^A - \gamma_x X \tag{180}$$

in which, with abuse of notation, we indicate D_{tot}^A with D^A. Defining $\alpha_0 = \alpha_b^A$, $\alpha_1 = \bar{\alpha}_b^A$, $K_{AA}^A = K_A K_A^A$, $K_{RR}^A = K_R K_R^A$, we can re-write (180) as follows:

$$\dot{X} = (\alpha_0 + \frac{\alpha_1 \frac{A^n}{K_{AA}^A}}{1 + \frac{A^n}{K_{AA}^A} + \frac{R^n}{K_{RR}^A}})D^A - \gamma_x X = \alpha_x D^A - \gamma_x X \tag{181}$$

in which we define $\alpha_x = \alpha_0 + \frac{\alpha_1 \frac{A^n}{K_{AA}^A}}{1+\frac{A^n}{K_{AA}^A}+\frac{R^n}{K_{RR}^A}}$. Now, let us define $\bar{D}^A = D^A/D_{tot} = n^A/\mathrm{D_{tot}}$, $\bar{X} = X/D_{tot} = n^X/\mathrm{D_{tot}}$, the normalized time $\tau = tk_M^A D_{tot}$, and the non-dimensional parameters $\bar{\gamma}_x = \gamma_x/(k_M^A D_{tot})$, $\bar{\alpha}_x = \alpha_x/(k_M^A D_{tot})$. With these definitions and letting $\dot{x} := dx/d\tau$, we can rewrite the system model in terms of non-dimensional variables and non-dimensional parameters as follows:

$$\dot{\bar{X}} = \bar{\alpha}_x \bar{D}^A - \bar{\gamma}_x \bar{X}. \tag{182}$$

5 Figures

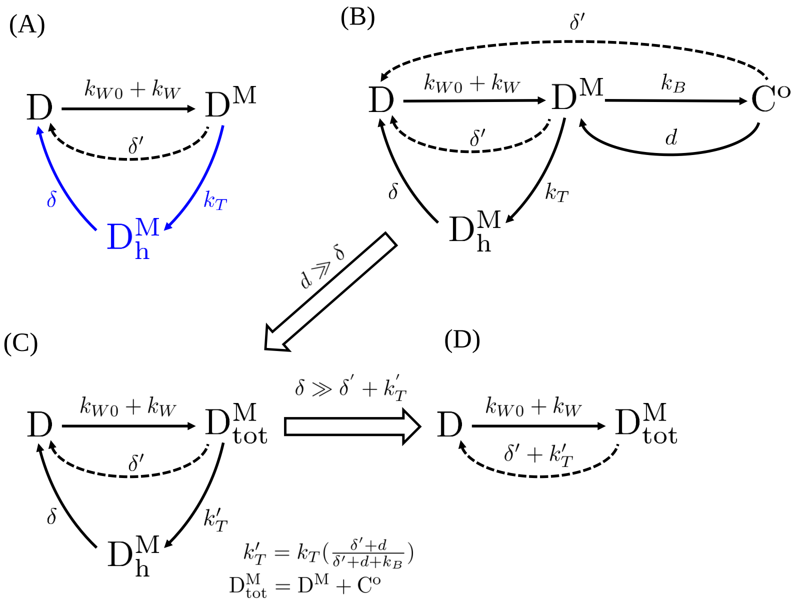

Figure A: **Establishment, erasure, and maintenance of DNA methylation**. (A) Diagram representing the reactions making up the DNA methylation system as consistent with earlier models [31] (black arrows). The key processes are *de novo* methylation, maintenance methylation and dilution due to DNA replication/ cell division. In addition to passive erasure, DNA methylation can be actively removed through the TET enzymatic pathway [14](Chapter 17) (blue arrows). Here, D represents a nucleosome with the DNA wrapped around characterized by an unmethylated single CpG, D^M represents a nucleosome with the DNA wrapped around characterized by a methylated single CpG and D_h^M represents a nucleosome with the DNA wrapped around characterized by a hydroxilmethylated CpG which is not recognized by DNMT1. Rate constants are defined in the main text. (B) Diagram representing the reactions making up the DNA methylation system, accounting for the mutual protection mechanism between MBD proteins and TET binding to DNA. (C) Simplified diagram shows an effective catalytic rate constant of TET $k_T^{'}$ which is substantially smaller than the theoretical one quantified *in vitro*. (D) Final simplified diagram obtained by introducing the fact that $\delta^{'} + k_T^{'} \ll \delta$.

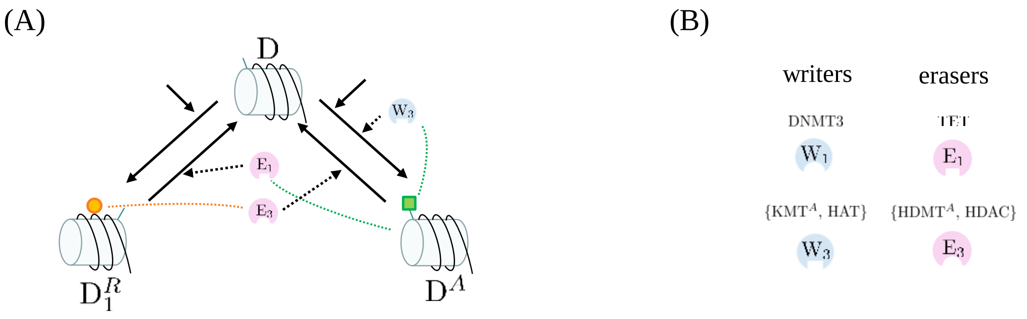

Figure B: **Competitive interactions between activating histone modifications D^A and DNA methylation D_1^R.** (A) Interaction diagram between D_1^R and D^A (nucleosome with an activating histone modification, H3K4me3 or H3Kac). DNA methylation recruits erasers of H3K4me3/ac and, in turn, H3K4me3 recruits TET enzymes for active removal of DNA methylation (see main text). We use colored dotted lines to depict the recruitment process done by H3K4me3/ac (green lines), and CpGme (orange lines) and we use dotted black arrows to depict the consequent effect on writing/erasing. The solid black arrow represents the nucleosome modification. (B) Enzymes that write (writers) and erase (erasers) each modification as explained in the main text. The socket on each of these enzymes represents a domain that binds to protein readers of the corresponding modification, enabling the process by which each modification recruits writers or erasers to nearby histones.

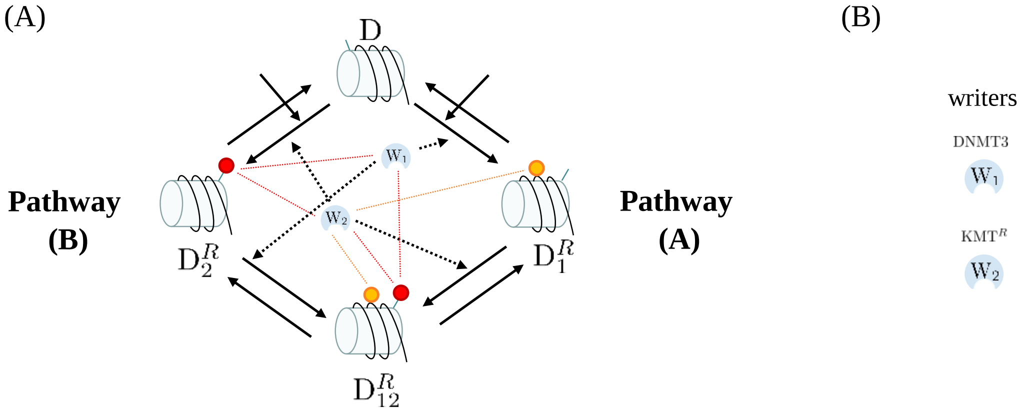

Figure C: **Cooperative interactions between DNA methylation D_1^R and repressive histone modification D_2^R.** (A) Diagram of the chemical reaction model, in which the two possible cooperation pathways, described in Section "Models" and Section 1.4, are highlighted. The species involved are D (unmodified nucleosome), D_1^R (nucleosome without any histone modification but with CpGme), D_2^R (nucleosome with a repressive histone modification, H3K9me3, but without methylated CpG) and D_{12}^R (nucleosome with both H3K9me3 and CpGme). We use colored dotted lines to depict the recruitment process done by H3K9me3 (red lines), and CpGme (orange lines) and we use dotted black arrows to depict the consequent effect on writing. The solid black arrow represents the nucleosome modification. (B) Enzymes that write (writers) each modification as explained in the main text. The socket on each of these enzymes represents a domain that binds to protein readers of the corresponding modification, enabling the process by which each modification recruits writers to nearby histones.

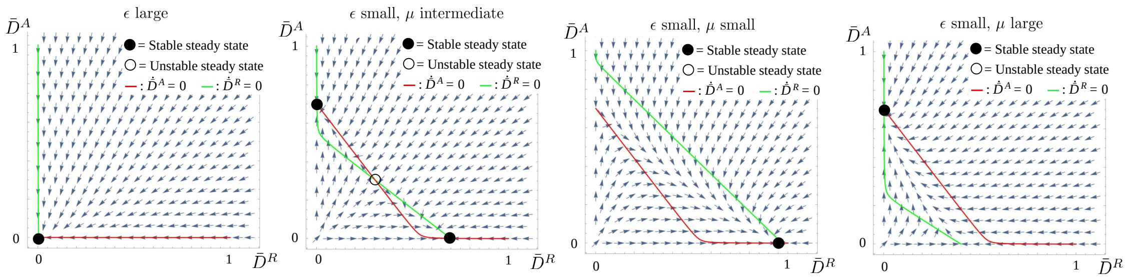

Figure D: **Competition between activating (Act) and repressive (Rep) histone modifications**. Analysis of number and stability of steady states for the ODE model in (29) with no external input ($\bar{u}^A = u_0^A$, $\bar{u}^R = u_0^R$). In the plots, the arrows represent the vector field, the green line represents the nullcline $\dot{\bar{D}}^R = 0$, the red line represents the nullcline $\dot{\bar{D}}^A = 0$ and their intersections, highlighted by a circle, represent the steady states of the system. There are four different plots, one for each qualitatively different parameter regime: ϵ large ($\epsilon > max\{1, \frac{\alpha}{\mu b}\}$), ϵ small - μ intermediate ($\epsilon < min\{1, \frac{\alpha}{\mu b}\}$, $\mu_1 < \mu < \mu_2$ with μ_1 and μ_2 defined in (91)), ϵ small - μ small ($\epsilon < min\{1, \frac{\alpha}{\mu b}\}$, $\mu < \mu_1$) and ϵ small - μ large ($\epsilon < min\{1, \frac{\alpha}{\mu b}\}$, $\mu > \mu_2$). The parameter values of each regime are listed in Table D.

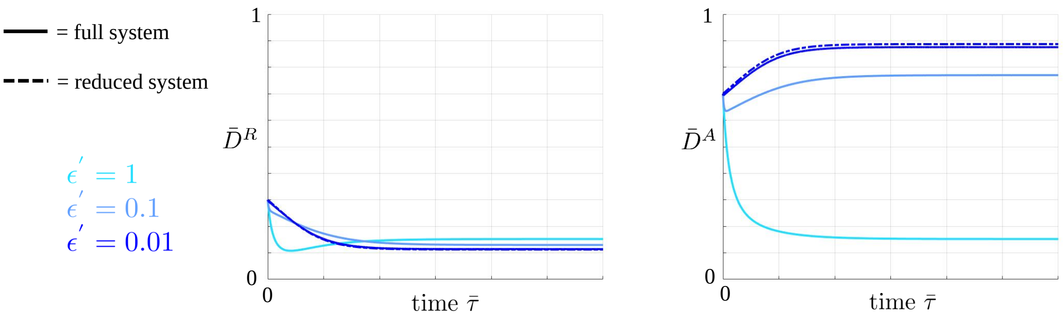

Figure E: **Trajectories of $\bar{D}^R$ and $\bar{D}^A$ of the full and the reduced system become close as $\epsilon^{'}$ decreases.** Trajectories of $\bar{D}^R$ and $\bar{D}^A$ of the full system (95), solid lines, and of the reduced system (112), dashed lines. We set $(\bar{D}^R(0), \bar{D}^A(0)) = (0.3, 0.7)$ as initial conditions and we use three different values for $\epsilon^{'}$, that is, from the lighter to the darker curve, $\epsilon^{'} = 1, 0.1, 0.01$. The values of the other parameters are listed in Table E.

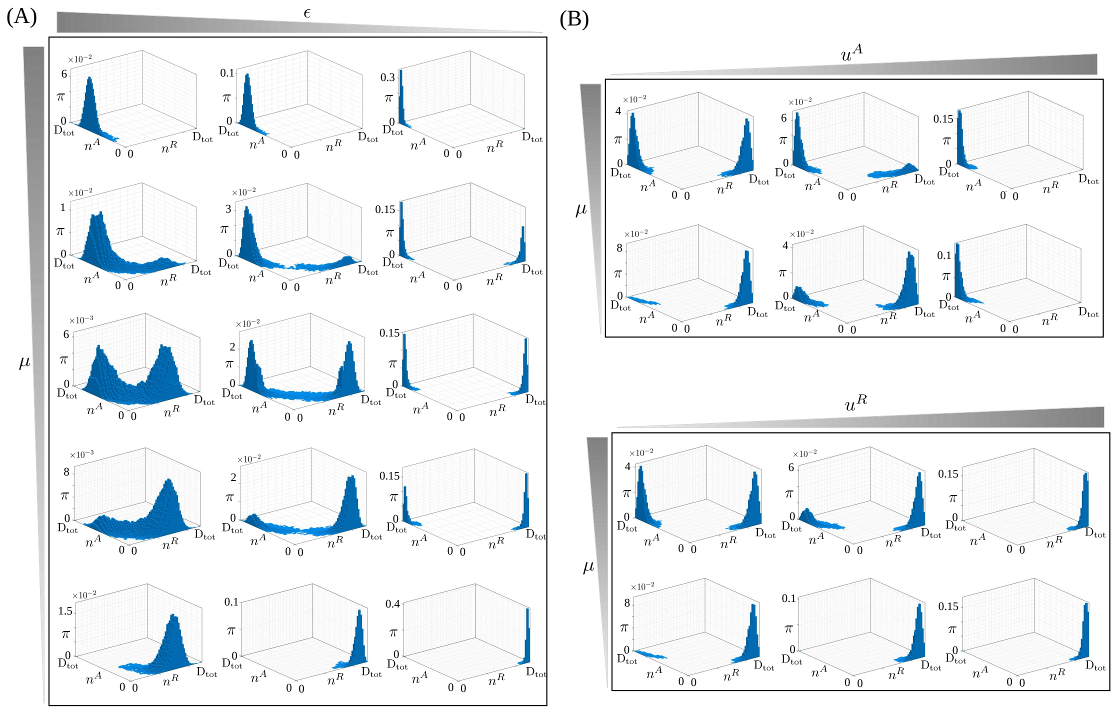

Figure F: **How the parameters ϵ, μ, u^A and u^R affect the stationary probability distribution of the histone modification circuit.** The stationary distribution of our system, represented in Fig 1C, obtained computationally. The stationary distributions are obtained by simulating the system of reactions listed in the blue box of Fig 2 with the SSA. (A) We consider three values of ϵ ($\epsilon = 0.4, 0.2, 0.02$) and five values of μ ($\mu = 10, 1.2, 1, 0.83, 0.1$). (B) We consider $\epsilon = 0.1$, two values of μ ($\mu = 1, 0.83$) and, in the plots above, three values of u^A ($u^A = 0, 0.1, 1$) and in the plots below three values of u^R ($u^R = 0, 0.1, 1$). The parameter values of each regime are listed in Table F. For all the simulations we consider $\alpha = 1$, $\epsilon^{'} = 1$ (Figs H-I show different values of $\epsilon^{'}$) and we decrease ϵ by decreasing $\delta + \bar{k}_E^A$ (similar results can be obtained if we change ϵ by varying k_M^A as shown in Fig G).

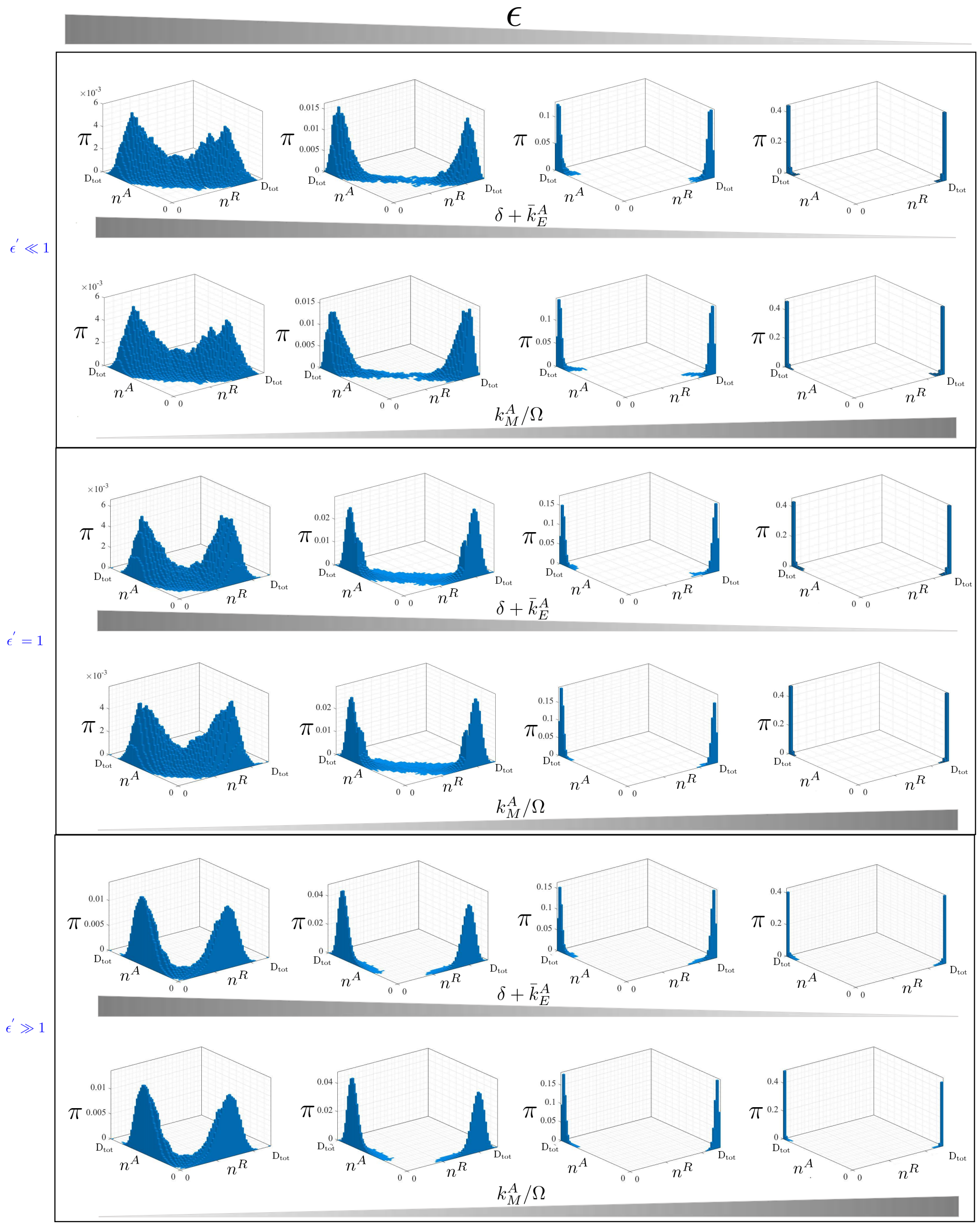

Figure G: **How the parameter ϵ affects the stationary probability distribution of the histone modification circuit.** The stationary distribution of our system, represented in Fig 1C, obtained computationally. The stationary distributions are obtained by simulating the system of reactions listed in Table G with the SSA. In particular, defining the reaction volume as Ω, we consider three different cases, $\epsilon^{'} \ll 1$, $\epsilon^{'} = 1$ and $\epsilon^{'} \gg 1$, and for each case we determine how decreasing ϵ (by decreasing $\delta + \bar{k}_E^A$ or increasing k_M^A/Ω) affects the stationary distribution of the system. The parameter values of each regime are listed in Table G. In particular, for the parameter regime $\epsilon^{'} \ll 1$ we consider $\epsilon = 0.2, 0.1, 0.02, 0.002$ and $\epsilon^{'} = 0.2$ for the distribution in which we decrease $\delta + \bar{k}_E^A$ and $\epsilon = 0.2, 0.1, 0.02, 0.002$ and $\epsilon^{'} = 0.2$ for the distribution in which we increase k_M^A/Ω; for the parameter regime $\epsilon^{'} = 1$ we consider $\epsilon = 0.4, 0.2, 0.02, 0.002$ and $\epsilon^{'} = 1$ for both cases and for the parameter regime $\epsilon^{'} \gg 1$ we consider $\epsilon = 0.5, 0.2, 0.02, 0.002$ and $\epsilon^{'} = 10$ for both cases.

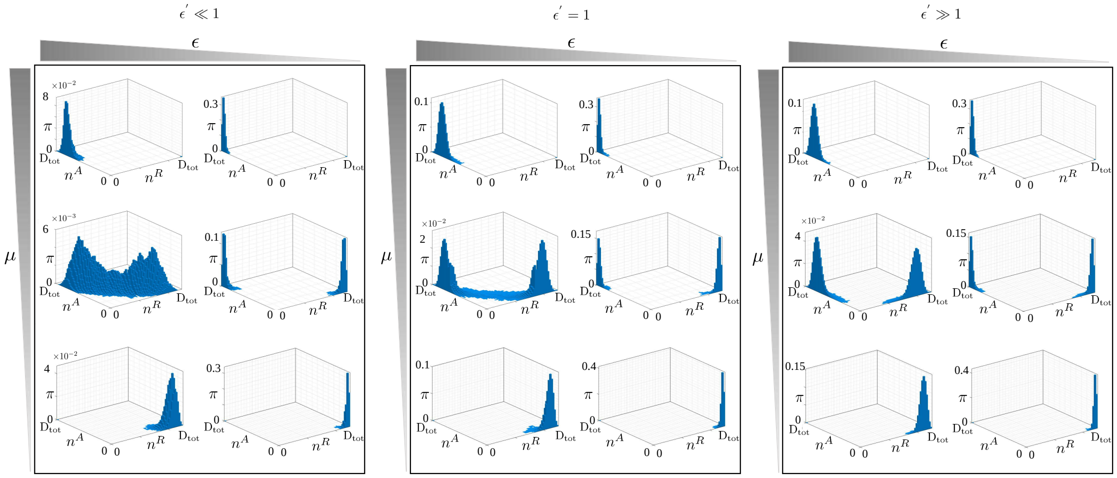

Figure H: **How the parameter μ affects the stationary probability distribution of the histone modification circuit for different values of $\epsilon^{'}$.** The stationary distribution of our system, represented in Fig 1C, obtained computationally. The stationary distributions are obtained by simulating the system of reactions listed in Table H with the SSA. We consider three different cases, $\epsilon^{'} \ll 1$, $\epsilon^{'} = 1$ and $\epsilon^{'} \gg 1$, and for each case we determine, for two values of ϵ ($\epsilon = 0.2, 0.02$), how μ affect the stationary distribution of the system. In particular, we consider three values of μ ($\mu = 0.1, 1, 10$). The parameter values of each regime are listed in Table H.

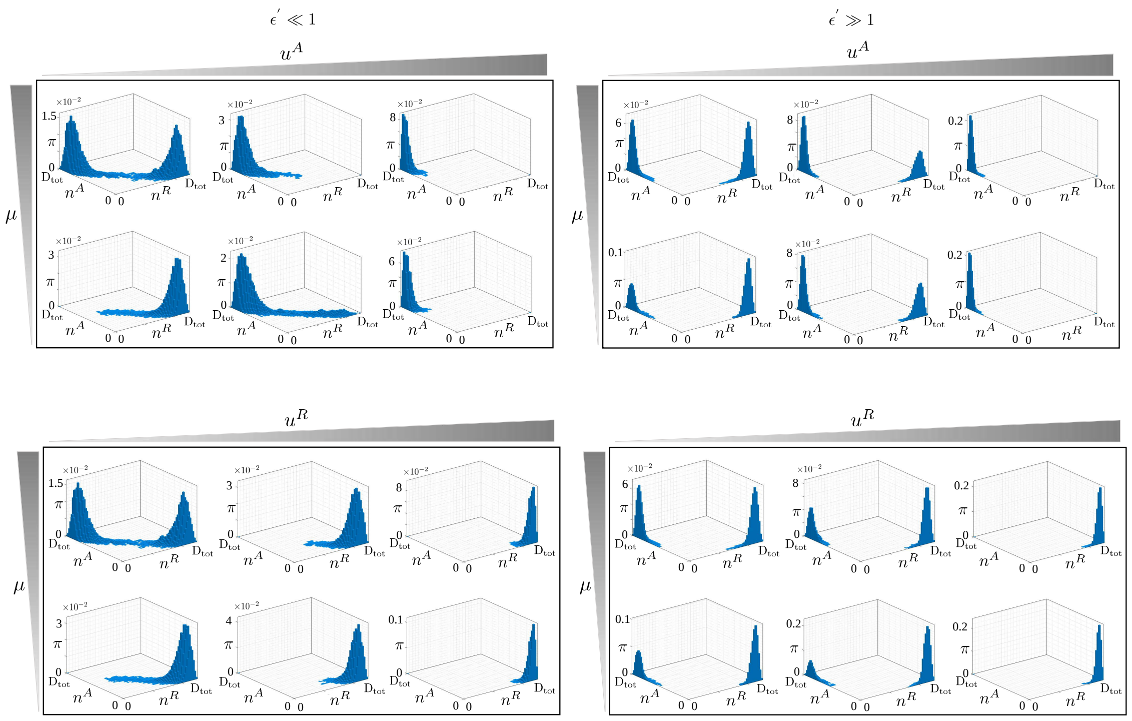

Figure I: **How the parameter u^A and u^R affect the stationary probability distribution of the histone modification circuit for different values of $\epsilon^{'}$.** The stationary distribution of our system, represented in Fig 1C, obtained computationally. The stationary distributions are obtained by simulating the system of reactions listed in Table I with the SSA. We consider, for two different regimes of $\epsilon^{'}$ ($\epsilon^{'} = 0.2$ and $\epsilon^{'} = 10$), $\epsilon = 0.1$, two values of μ ($\mu = 1, 0.83$) and, in the plots above, three values of u^A ($u^A = 0, 0.1, 1$) and in the plots below three values of u^R ($u^R = 0, 0.1, 1$). The parameter values of each regime are listed in Table I.

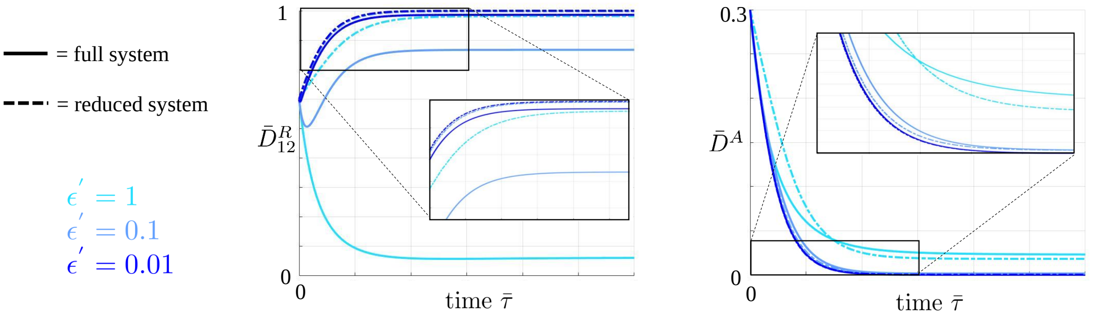

Figure J: **Trajectories of $\bar{D}^R_{12}$ and $\bar{D}^A$ of the full and the reduced system are close as $\epsilon^{'}$ decreases.** Trajectories of $\bar{D}^R_{12}$ and $\bar{D}^A$ of the full system (145), solid lines, and of the reduced system (155), dotted lines. We set $(\bar{D}^R_{12}(0), \bar{D}^A(0)) = (0.7, 0.3)$ as initial conditions and we use three different values for $\epsilon^{'}$, that is, from the lighter to the darker curve, $\epsilon^{'} = 1, 0.1, 0.01$. The values of the other parameters are listed in Table J.

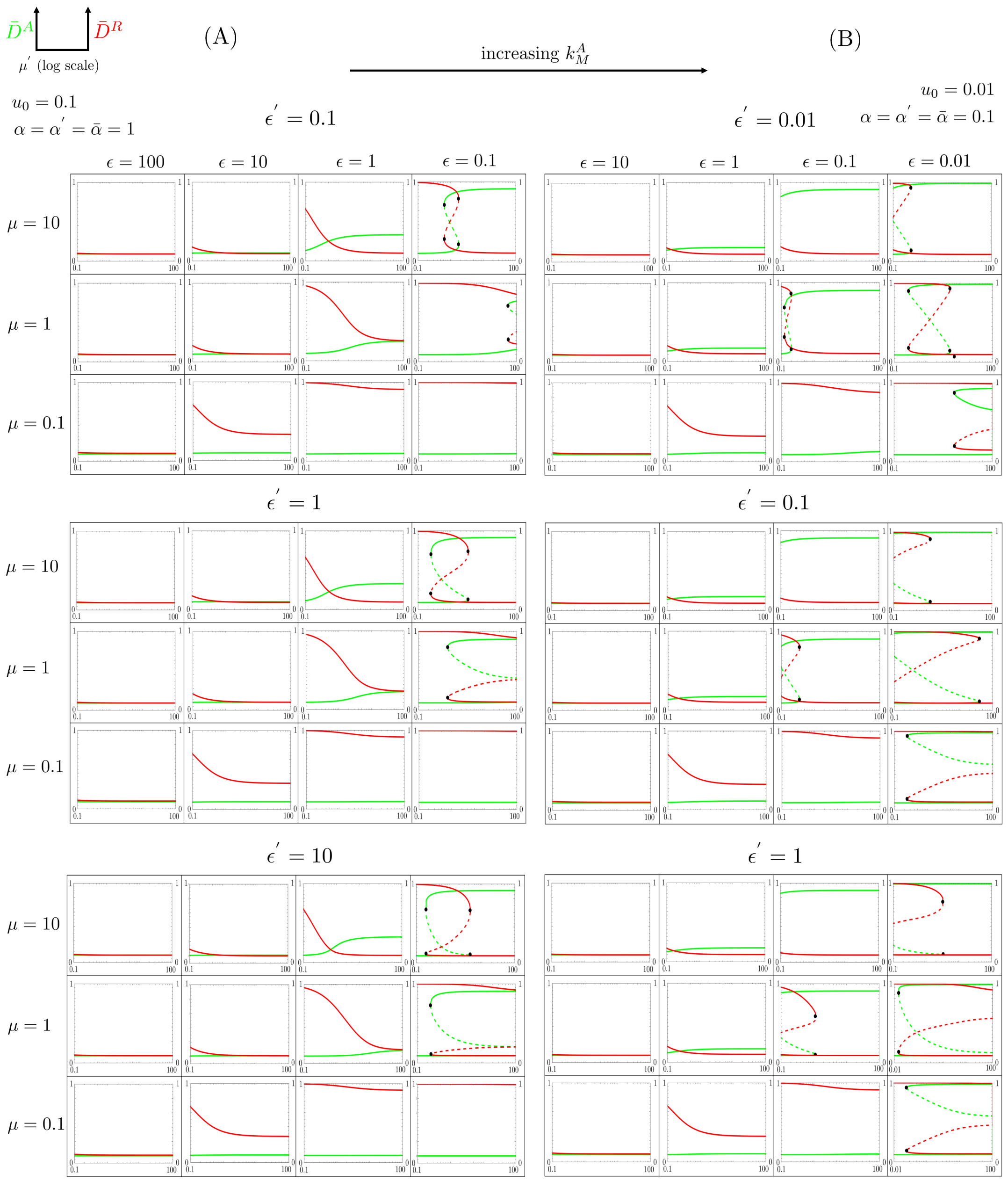

Figure K: **Bifurcation plots related to system Main Text: Eqs (3) with no external inputs ($u^A = u_1^R = u_2^R = 0$ and $u_0^A = u_{10}^R = u_{20}^R = u_0$ small).** Here, defining $n^R = n_1^R + n_2^R + n_{12}^R$, $\bar{D}^A := n^A/\mathrm{D_{tot}}$ and $\bar{D}^R := n^R/\mathrm{D_{tot}}$ represent the fractions of nucleosomes with activating or repressive modifications within the gene with a total of $\mathrm{D_{tot}}$ nucleosomes. On the y axis we have $\bar{D}^A$ (green) and $\bar{D}^R$ (red) and on the x axis we have $\mu^{'}$ (log scale). The solid line represents stable steady states, the dotted line represents unstable steady states and the black circle represents the bifurcation point (saddle-node bifurcation). (A) On the left side, we realize several bifurcation plots for different values of ϵ ($\epsilon = 0.1, 1, 10, 100$), different values of μ ($\mu = 0.1, 1, 10$) and different values of $\epsilon^{'}$ ($\epsilon^{'} = 0.1, 1, 10$). (B) On the right side we increase k_M^A of one order of magnitude, and this, based on the definition of the dimensionless parameters as done in Main Text: Eqs (3), leads to decrease ϵ, $\epsilon^{'}$, u_0, α, $\alpha^{'}$ and $\bar{\alpha}$ of one order of magnitude. A higher k_M^A increases the stability of the active state.

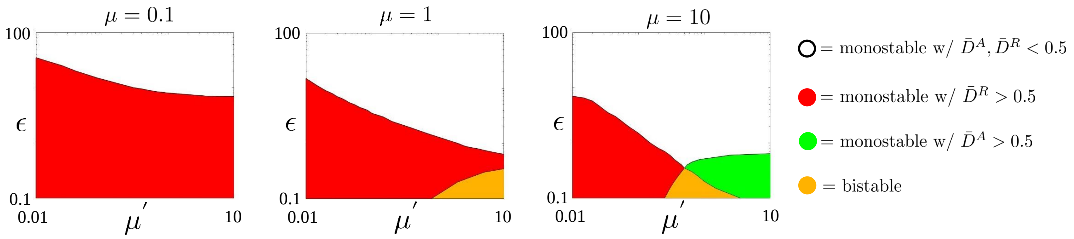

Figure L: **Charts depicting the (ϵ, μ') combinations that result in a monostable (red, green or white) or bistable (yellow) system for different values of μ.** Here, consider $\epsilon' = 1$ and three different values of μ ($\mu = 0.1, 1, 10$).

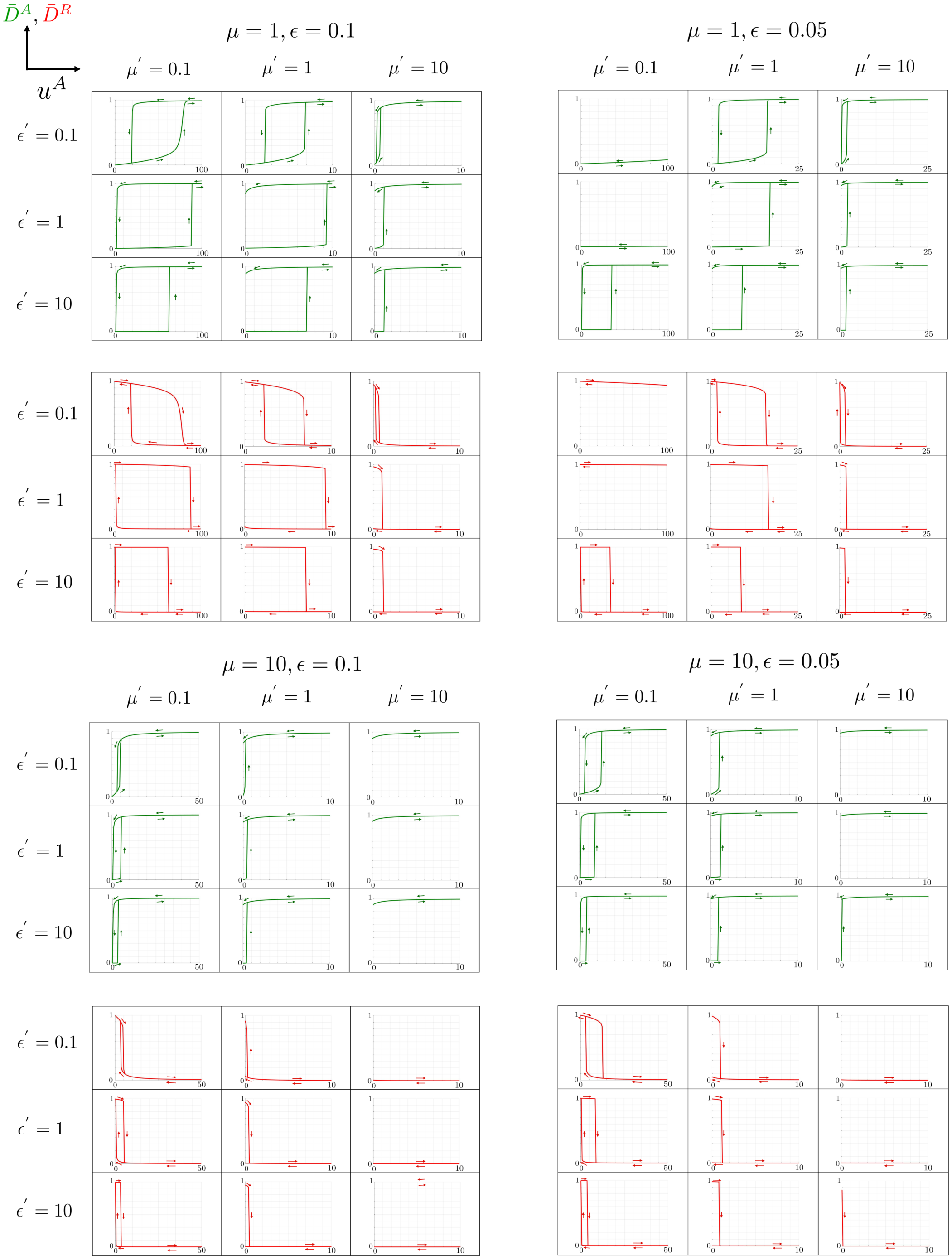

Figure M: **Input/output steady state characteristics displaying hysteresis related to system Main Text: Eqs (3) with u^A as input**. On the y axis we have $\bar{D}^A$ (green) or $\bar{D}^R$ (red) and on the x axis we have the external input u^A. In particular, we set $u_0^A = u_{10}^R = u_{20}^R = u_0 = 0.1$, $u^R = 0$, as initial conditions we consider $(\bar{D}^R, \bar{D}^A) = (1, 0)$ and we realize several I/O plots for three values of μ' ($\mu' = 0.1, 1, 10$), two values of μ ($\mu = 1, 10$), two values of ϵ ($\epsilon = 0.1, 0.05$), and three values of ϵ' ($\epsilon' = 0.1, 1, 10$). All the other parameters are set equal to 1. In all plots $u^A := k_W^A/(k_M^A D_{tot})$, $u_i^R := k_W^i/(k_M^A D_{tot})$ for $i \in \{1, 2\}$.

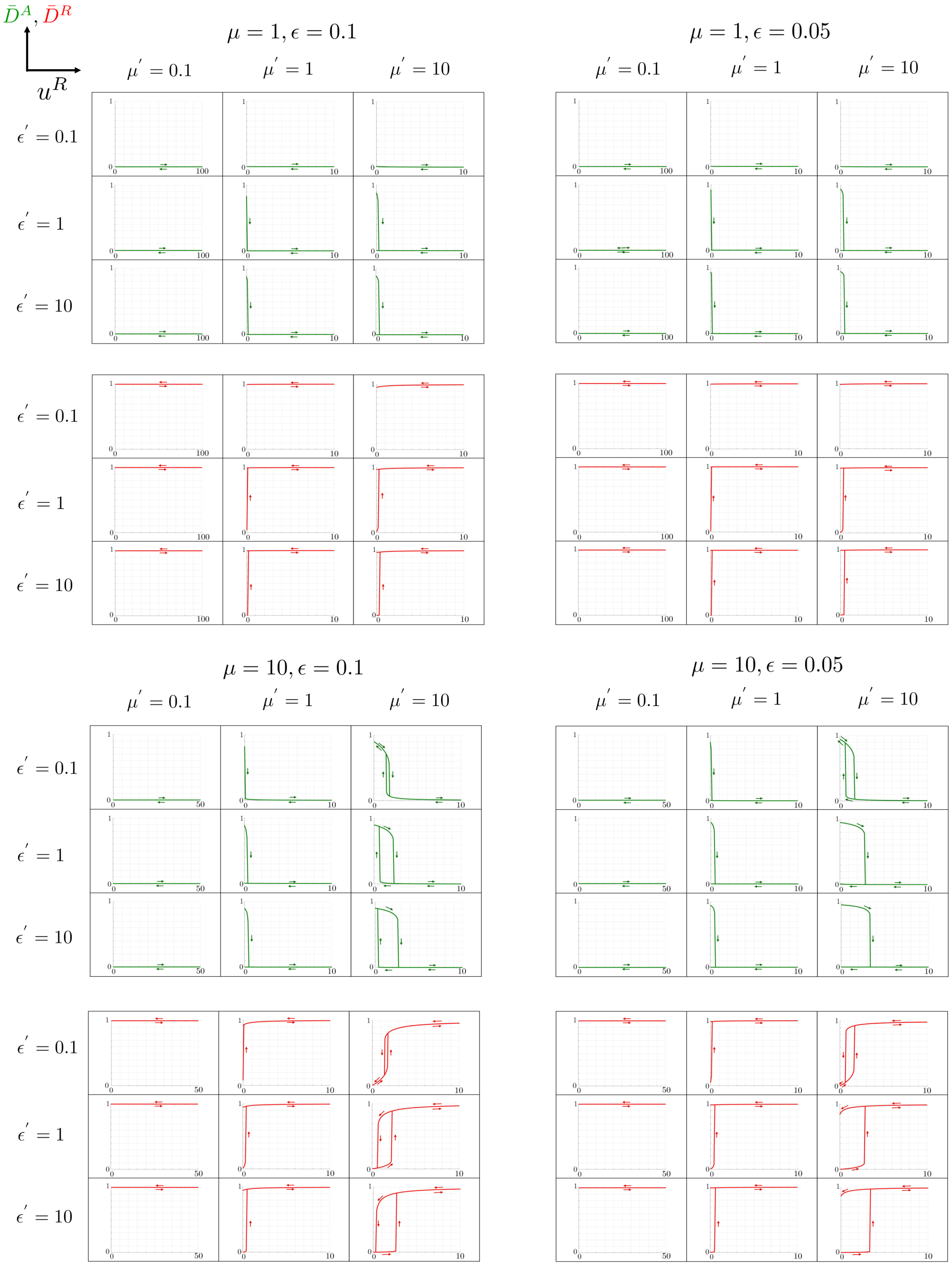

Figure N: **Input/output steady state characteristics displaying hysteresis related to system Main Text: Eqs (3) with u^R as input**. On the y axis we have $\bar{D}^A$ (green) or $\bar{D}^R$ (red) and on the x axis we have the external input u^R, with u^R defined as $u^R = u_1^R = u_2^R$. In particular, we set $u_0^A = u_{10}^R = u_{20}^R = u_0 = 0.1$, $u^A = 0$, as initial conditions we consider $(\bar{D}^R, \bar{D}^A) = (0, 1)$ and and we realize several I/O plots for three values of $\mu^{'}$ ($\mu^{'} = 0.1, 1, 10$), two values of μ ($\mu = 1, 10$), two values of ϵ ($\epsilon = 0.1, 0.05$), and three values of $\epsilon^{'}$ ($\epsilon^{'} = 0.1, 1, 10$). All the other parameters are set equal to 1. In all plots $u^A := k_W^A/(k_M^A D_{tot})$, $u_i^R := k_W^i/(k_M^A D_{tot})$ for $i \in \{1, 2\}$.

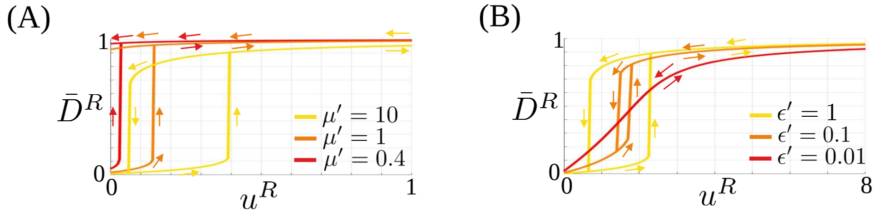

Figure O: **Input/output steady state characteristics for the $(u^R, \bar{D}^R)$ pair for different values of $\epsilon^{'}$ and $\mu^{'}$ obtained from simulations of system Main Text: Eqs (3).** (A) We consider $(\bar{D}^R, \bar{D}^A) = (0, 1)$ as initial conditions and we set $u^A = 0$, $\epsilon = 0.07$, $u_0^A = u_{10}^R = u_{20}^R = 0.1$, $\alpha = \bar{\alpha} = \alpha^{'} = 0.1$, and all the other parameters equal to 1. (B) We consider $(\bar{D}^R, \bar{D}^A) = (0, 1)$ as initial conditions and we set $u^A = 0$, $\epsilon = 0.1$, $\mu = 10$, $\mu^{'} = 10$, $u_0^A = u_{10}^R = u_{20}^R = 0.1$ and all the other parameters equal to 1. In all plots $u^A = k_W^A/(k_M^A D_{tot})$, $u_i^R = k_W^i/(k_M^A D_{tot})$ for $i \in \{1, 2\}$.

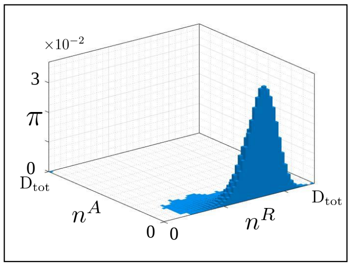

Figure P: **Stationary probability distribution of the chromatin modification circuit with no external inputs ($u^A = u_1^R = u_2^R = 0$ and $u_0^A = u_{10}^R = u_{20}^R = u_0$ small) and all the parameters with the same order of magnitude.** The stationary distribution of our system, represented by the circuit in Fig 3A, obtained computationally. The stationary distributions are obtained by simulating the system of reactions listed in Table K with the SSA and we indicate with n^R the total number of nucleosomes characterized by repressive chromatin modifications, that is $n^R = n_1^R + n_2^R + n_{12}^R$. The parameter values of each regime are listed in Table K. In particular, we set $u^A = u_1^R = u_2^R = 0$ and $u_0^A = u_{10}^R = u_{20}^R = 0.1$, $\epsilon = 1$, $\epsilon^{'} = 1$, $\mu = 1$, $\mu^{'} = 1$ and $\alpha = \bar{\alpha} = \alpha^{'} = 1$.

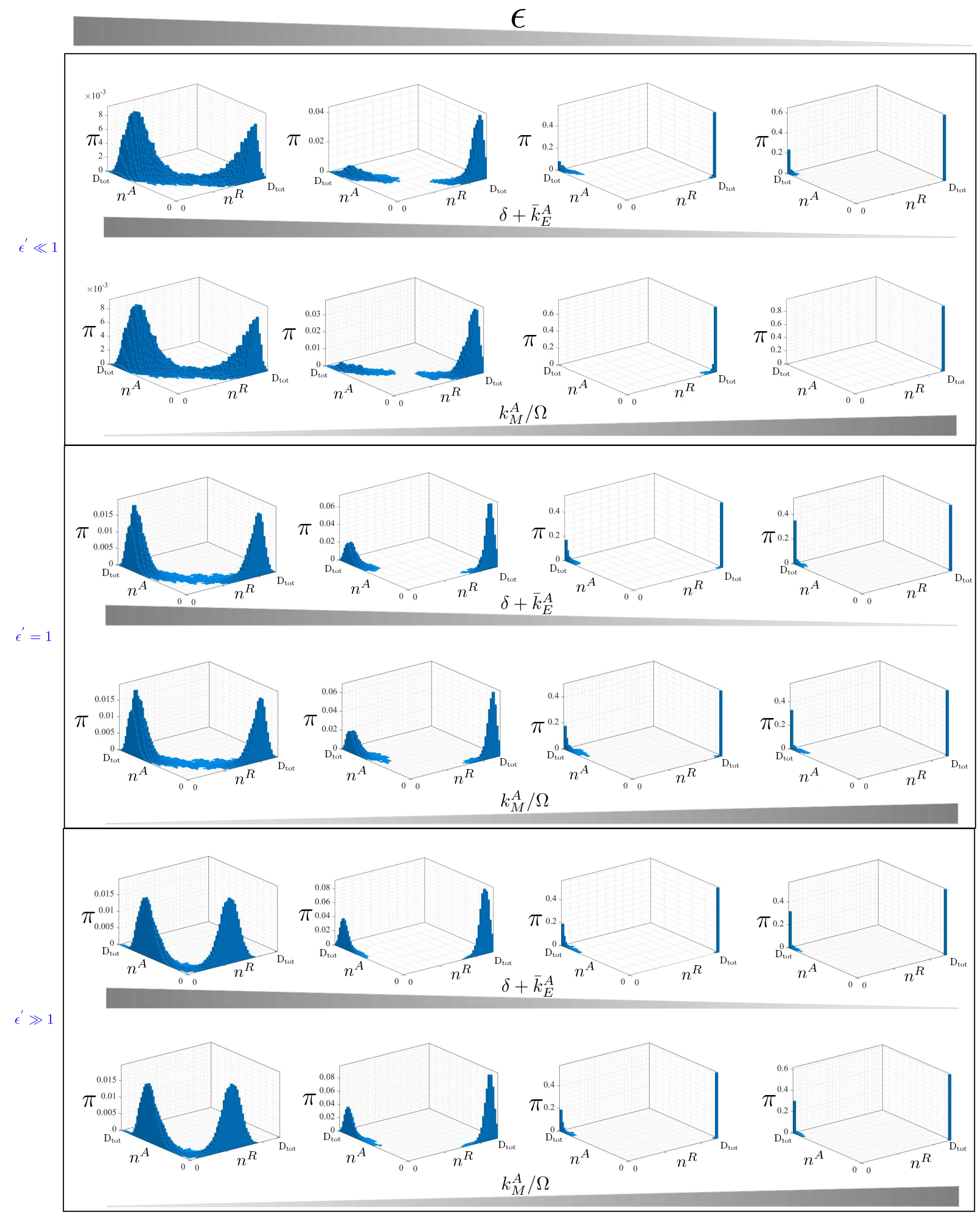

Figure Q: **How the parameter ϵ affects the stationary probability distribution of the chromatin modification circuit.** The stationary distribution of our system, represented by the circuit in Fig 3A, obtained computationally. The stationary distributions are obtained by simulating the system of reactions listed in Table L with the SSA and we indicate with n^R the total number of nucleosomes characterized by repressive chromatin modifications, that is $n^R = n_1^R + n_2^R + n_{12}^R$. In particular, defining the reaction volume as Ω, we consider three different cases, $\epsilon^{'} \ll 1$, $\epsilon^{'} = 1$ and $\epsilon^{'} \gg 1$, and for each case we determine how decreasing ϵ (by decreasing $\delta + \bar{k}_E^A$ or increasing k_M^A/Ω) affect the stationary probability distribution of the system. The parameter values of each regime are listed in Table L. In particular, for the parameter regime $\epsilon^{'} \ll 1$ we consider $\epsilon = 0.16, 0.12, 0.02, 0.002$ and $\epsilon^{'} = 0.2$ for the distribution in which we decrease $\delta + \bar{k}_E^A$ and $\epsilon = 0.16, 0.12, 0.02, 0.002$ and $\epsilon^{'} = 0.2, 0.17, 0.025, 0.0025$ for the distribution in which we increase k_M^A/Ω; for the parameter regime $\epsilon^{'} = 1$ we consider $\epsilon = 0.19, 0.12, 0.02, 0.002$ and $\epsilon^{'} = 1$ for both cases and for the parameter regime $\epsilon^{'} \gg 1$ we consider $\epsilon = 0.36, 0.12, 0.02, 0.002$ and $\epsilon^{'} = 10$ for both cases.

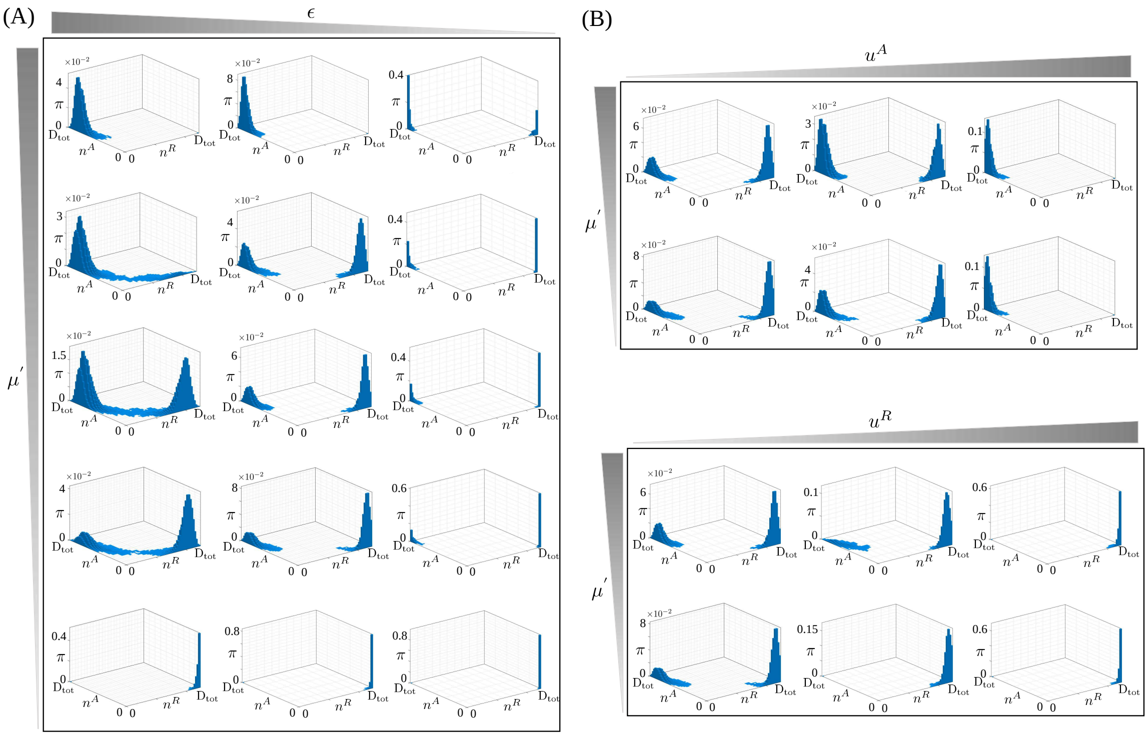

Figure R: **How the parameters ϵ, μ', u^A and u^R affect the stationary probability distribution of the chromatin modification circuit.** The stationary distributions are obtained by simulating the system of reactions listed in Fig 3A with the SSA and we indicate with n^R the total number of nucleosomes characterized by repressive chromatin modifications, that is $n^R = n_1^R + n_2^R + n_{12}^R$. (A) We consider three values of ϵ ($\epsilon = 0.19, 0.12, 0.02$) and five values of μ' ($\mu = 10, 1.2, 1, 0.8, 0.1$). (B) We consider $\epsilon = 0.12$, two values of μ' ($\mu = 1, 0.8$) and, in the plots above, three values of $u^A = 0, 0.07, 1$ and in the plots below three values of u^R ($u^R = 0, 0.05, 1$), in which $u^R = u_1^R = u_2^R$. The parameter values of each regime are listed in Table M. For all the simulations we consider $\alpha = \bar{\alpha} = \alpha' = 0.2$, $\epsilon' = 1$ and $\mu = 1$ (Figs S-T show different values of ϵ' and μ) and we decrease ϵ by decreasing $\delta + \bar{k}_E^A$ (similar results can be obtained if we change ϵ by varying k_M^A as shown in Fig Q).

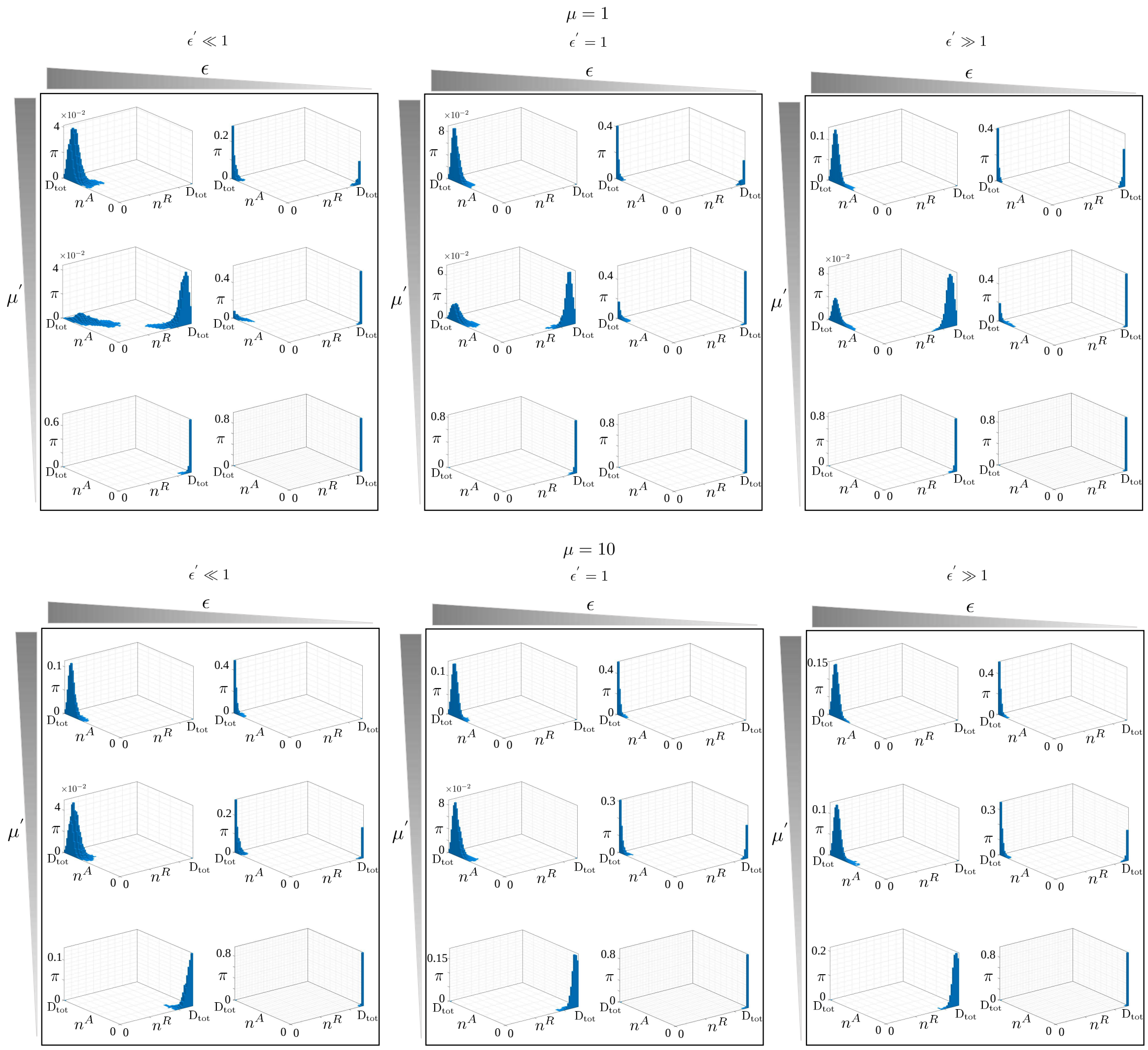

Figure S: **How the parameters μ and $\mu^{'}$ affect the stationary probability distribution of the chromatin modification circuit for different values of $\epsilon^{'}$.** The stationary distribution of our system, represented by the circuit in Fig 3A, obtained computationally. The stationary distributions are obtained by simulating the system of reactions listed in Table N with the SSA and we indicate with n^R the total number of nucleosomes characterized by repressive chromatin modifications, that is $n^R = n_1^R + n_2^R + n_{12}^R$. We consider three different cases, $\epsilon^{'} \ll 1$, $\epsilon^{'} = 1$ and $\epsilon^{'} \gg 1$, and for each case we determine, for two values of ϵ ($\epsilon = 0.12, 0.02$), how μ and $\mu^{'}$ affect the stationary probability distribution of the system. In particular, we consider two values of μ ($\mu = 1, 10$) and three values of $\mu^{'}$ ($\mu = 0.1, 1, 10$). For all the simulations we consider $\alpha = \bar{\alpha} = \alpha^{'} = 0.2$. The parameter values of each regime are listed in Table N.

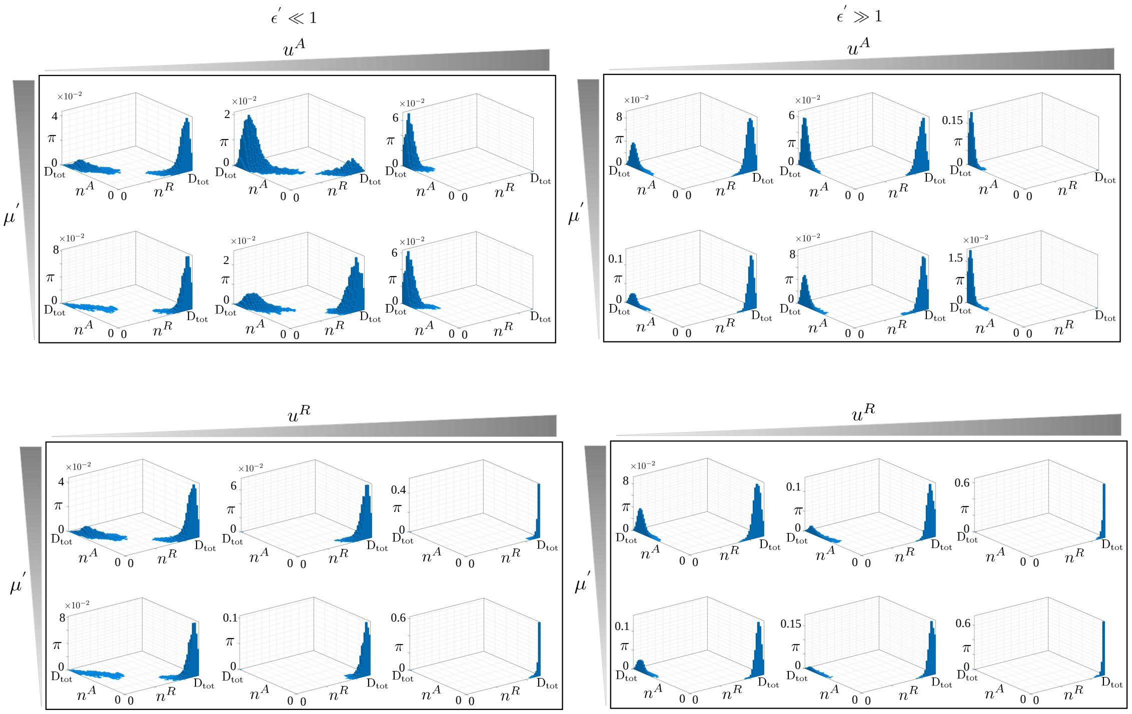

Figure T: **How the parameter u^A and u^R affect the stationary probability distribution of the chromatin modification circuit for different values of $\epsilon^{'}$.** The stationary distribution of our system, represented by the circuit in Fig 3A, obtained computationally. The stationary distributions are obtained by simulating the system of reactions listed in Table O with the SSA and we indicate with n^R the total number of nucleosomes characterized by repressive chromatin modifications, that is $n^R = n_1^R + n_2^R + n_{12}^R$. We consider, for two different regimes of $\epsilon^{'}$ ($\epsilon^{'} = 0.2$ and $\epsilon^{'} = 10$), $\epsilon = 0.12$, $\mu = 1$, two values of $\mu^{'}$ ($\mu = 1, 0.8$) and, in the plots above, three values of u^A ($u^A = 0, 0.07, 1$) and in the plots below three values of u^R ($u^R = 0, 0.05, 1$). For all the simulations we consider $\alpha = \bar{\alpha} = \alpha^{'} = 0.2$. The parameter values of each regime are listed in Table O.

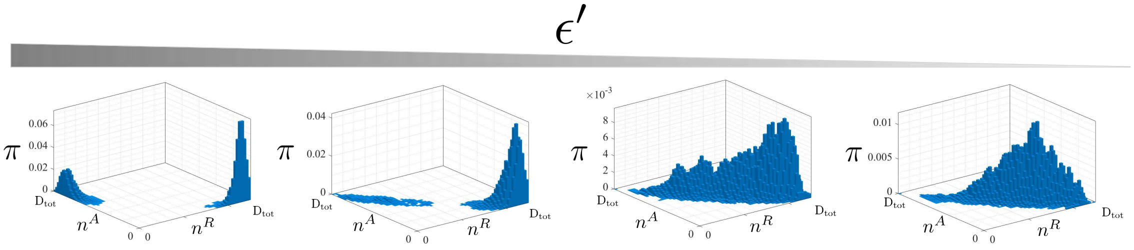

Figure U: **The effect of $\epsilon^{'} \ll \epsilon$ on the stationary probability distribution of the chromatin modification circuit.** The stationary distribution of our system, represented by the circuit in Fig 3A, obtained computationally. The stationary distributions are obtained by simulating the system of reactions listed in Table P with the SSA and we indicate with n^R the total number of nucleosomes characterized by repressive chromatin modifications, that is $n^R = n_1^R + n_2^R + n_{12}^R$. We set $\epsilon = 0.12$, $\mu^{'} = 1$, $\mu = 1$ and we consider four values of $\epsilon^{'}$ ($\epsilon^{'} = 1, 0.12, 0.01, 0.001$). The parameter values of each regime are listed in Table P.

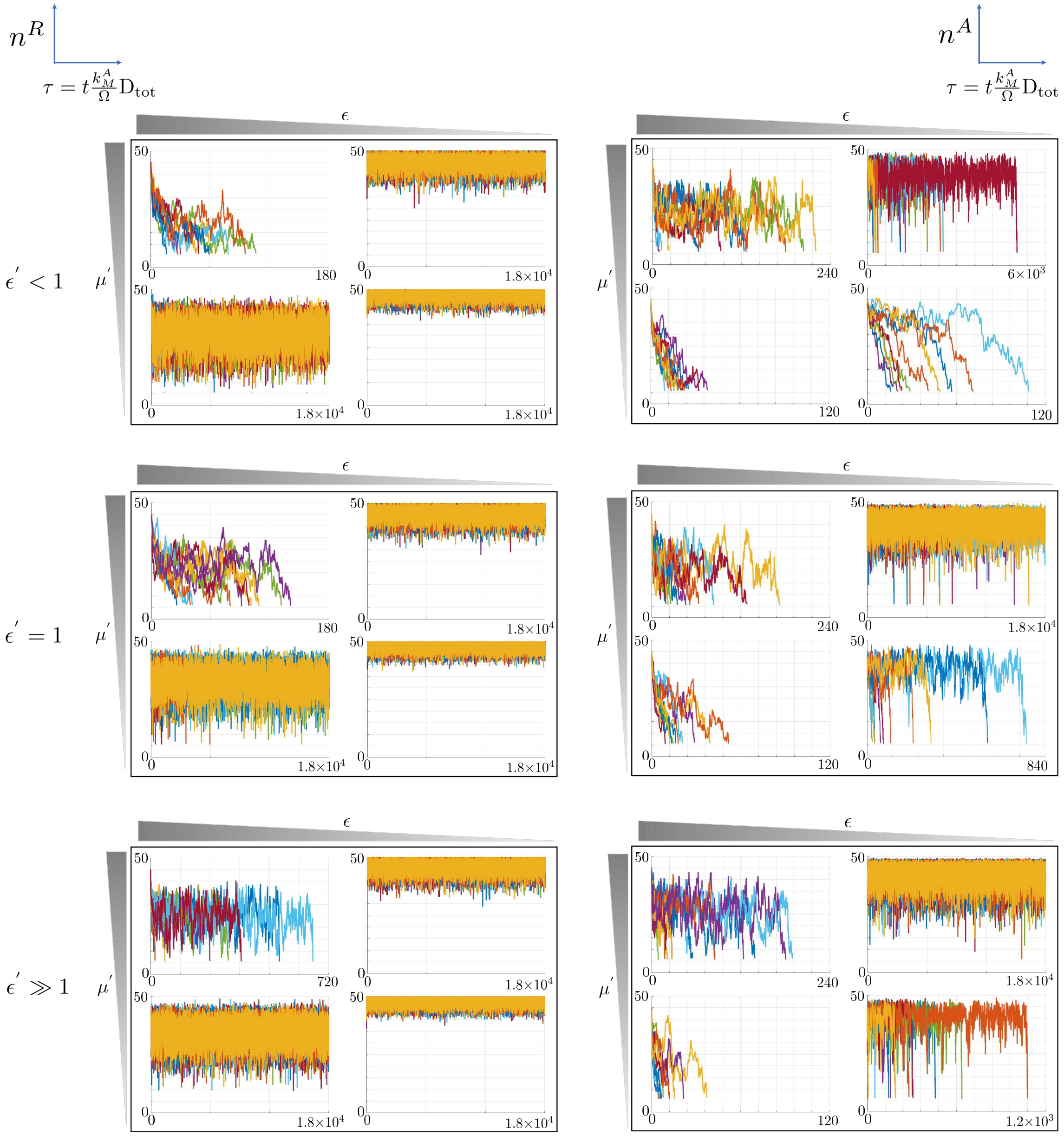

Figure V: **How the key parameters affect the time to memory loss of the active and repressed state of the chromatin modification circuit for different values of ϵ'.** The plots on the left side are related to the time to memory loss of the repressed state. In particular, we indicate with n^R the total number of nucleosomes characterized by repressive chromatin modifications, that is $n^R = n_1^R + n_2^R + n_{12}^R$ and we plot, for several values of ϵ, μ' and ϵ', the time trajectories of system starting from a repressed chromatin state $n^A = 5$, $n_1^R = n_2^R = n_{12}^R = 15$ and we stop the simulation when the trajectory reaches $n^R = 6$ for the first time. The plots on the right side are related to the time to memory loss of the active state and in this case we plot, for several values of ϵ, μ' and ϵ', the time trajectories of system starting from an active chromatin state $n^A = 45$, $n_{12}^R = 5$ and we stop the simulation when the trajectory reaches $n^A = 6$ for the first time. In all the plots on the x axis we have the time normalized with respect to $\frac{k_M^A}{\Omega}\mathrm{D_{tot}}$. The parameter values of each panel are listed in Table Q. In particular, we consider $\epsilon = 0.36, 0.12$, $\mu' = 1, 0.5$, $\mu = 1$ and $\epsilon' = 0.4, 1, 10$. In each panel, the number of trajectories plotted is 10.

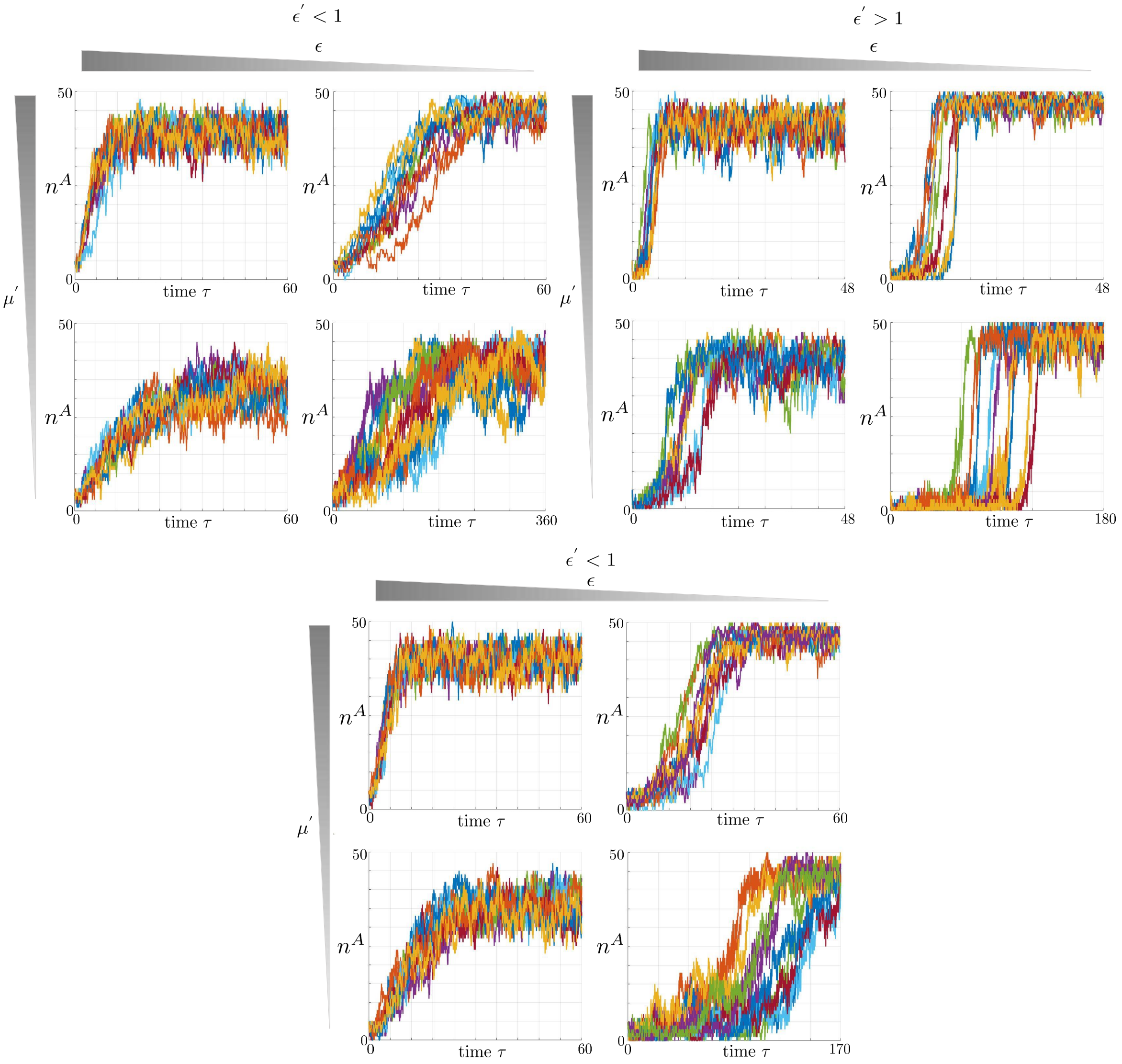

Figure W: **How the key parameters affect the reactivation of repressed chromatin state for different values of $\epsilon^{'}$.** Time trajectories of system starting from $n^R = 45$,$n^A = 5$ and considering an input u^A that, at steady state, leads to a unimodal distribution in the proximity of the active chromatin state $n^A = \mathrm{D_{tot}}$. The parameter values of each panel are listed in Table R. In particular, we set $u^A = 1.62$ and we consider two values of $\mu^{'}$ ($\mu^{'} = 0.6, 0.2$), two values of ϵ ($\epsilon = 0.48, 0.16$) and three values of $\epsilon^{'}$ ($\epsilon^{'} = 5, 1, 0.3$). In each panel, the number of trajectories plotted is 10.

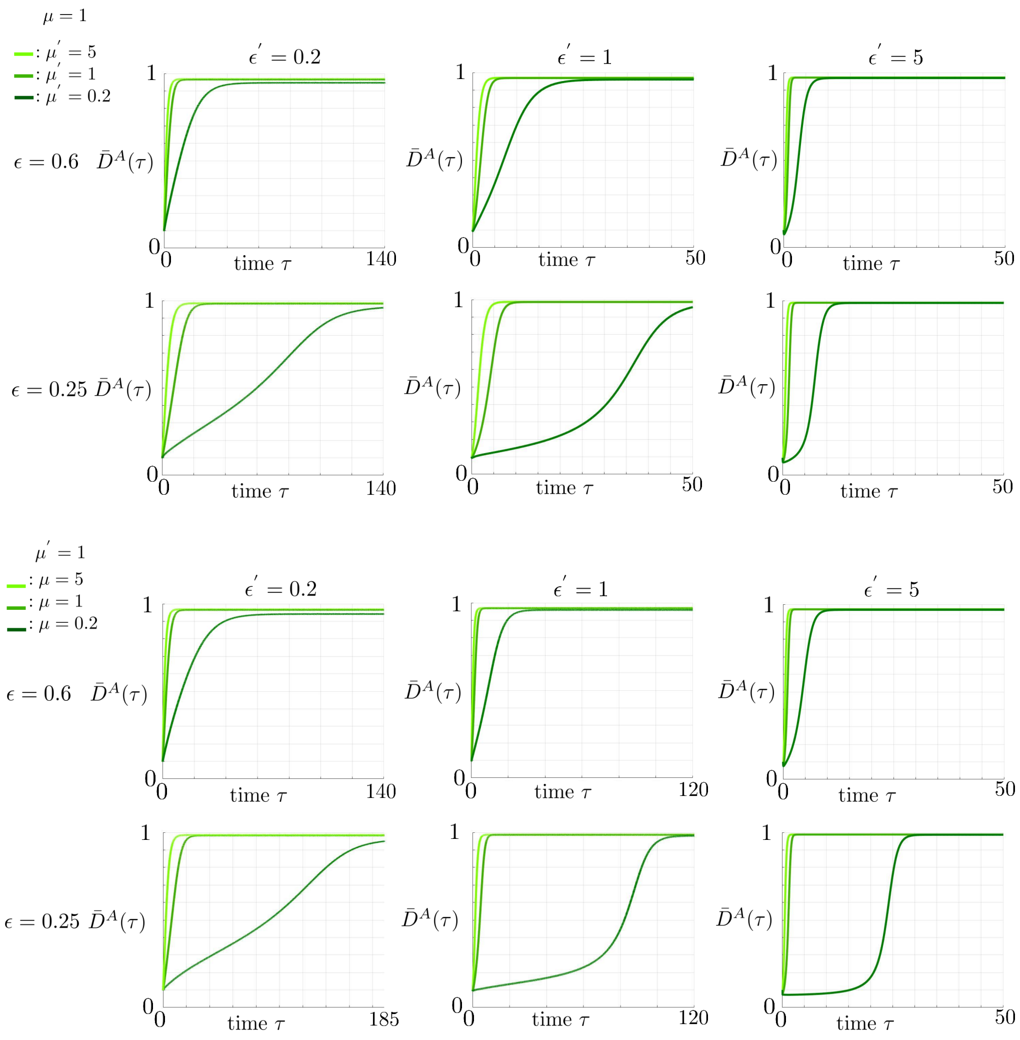

Figure X: **Time trajectories of $\bar{D}^A$ for different values of ϵ', μ' and μ.** We set $u^A = 20$, $u_0^A = u_{10}^R = u_{20}^R = 0.1$, $\alpha = \bar{\alpha} = \alpha' = 1$ and realize several time trajectories of $\bar{D}^A$ for different values of μ', μ, ϵ and ϵ', starting from $\bar{D}^A(0) = 0.1$, $\bar{D}^R(0) = 0.9$. In all the plots on the x axis we have the normalized time $\tau = t\frac{k_M^A}{\Omega}\mathrm{D_{tot}}$.

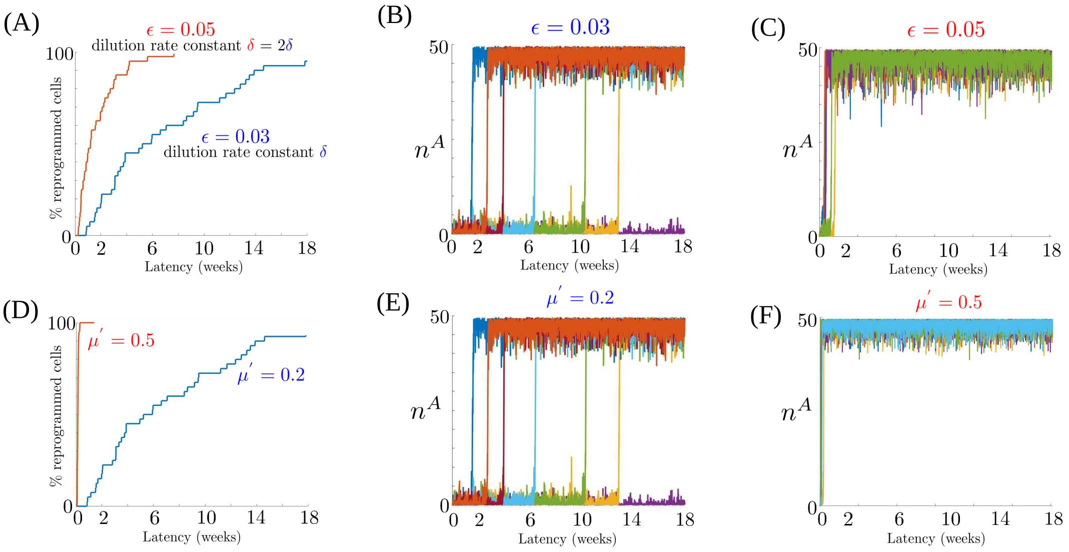

Figure Y: **Effect of ϵ and $\mu^{'}$ on the the silencing and reactivation processes**. (A) Graph representing the % of reprogrammed cells (% of time trajectories, starting from $n^A = 0$, that reach $n^A > 40$) as function of time for two different values of ϵ. (B)-(C) Time trajectories obtained by simulating the system of reactions listed in Table S with the SSA, starting from $n^R = 0, n^A = 50$. The parameter values of each panel are listed in Table S. In particular, we set $\mu^{'} = 0.2$, $\mu = 1$ and $\epsilon^{'} = 1$, $\epsilon = 0.03$ in (B) and $\epsilon = 0.05$ in (C). (D) Graph representing the % of reprogrammed cells (% of time trajectories, starting from $n^A = 0$, that reach $n^A > 40$) as function of time for two different values of $\mu^{'}$. (E)-(F) Time trajectories obtained by simulating the system of reactions listed in Table S with the SSA, starting from $n^R = 0, n^A = 50$. The parameter values of each panel are listed in Table S. In particular, we set $\epsilon = 0.03$, $\mu = 1$ and $\epsilon^{'} = 1$, $\mu^{'} = 0.2$ in (E) and $\mu^{'} = 0.5$ in (F).

6 Tables

It is important to point out that D_{tot} represents the total number of nucleosomes in a gene. Since we can assume about one nucleosome per 200 pb [66](Chapter 4) and we can assume that an average gene spans 10,000–20,000 bp [67], D_{tot} can be considered on average between 50 and 100. In particular, in our computational analysis we consider $D_{tot} = 50$.

R_j	Reaction	Prop.Func.(a_j)	Param.	Value (h^{-1})<br>Fig 4A	Value (h^{-1})<br>Fig 4B
1	$D \xrightarrow{k^A_{W0}} D^A$	$a_1 = k^A_{W0} n^D$	k^A_{W0}	3.5	3.5
2	$D \xrightarrow{k^A_W} D^A$	$a_2 = k^A_W n^D$	k^A_W	0<br>0	0, 50 (upper plots)<br>0 (lower plots)
3	$D^A \xrightarrow{\bar{k}^A_E} D$	$a_3 = \bar{k}^A_E n^A$	$\bar{k}^A_E$	4.7, 0.5	3
4	$D^A \xrightarrow{\delta} D$	$a_4 = \delta n^A$	δ	4.7, 0.5	3
5	$D + D^A \xrightarrow{k^A_M} D^A + D^A$	$a_5 = \frac{k^A_M}{\Omega} n^D n^A$	$\frac{k^A_M}{\Omega}$	1	1
6	$D^A + D^R_1 \xrightarrow{k^A_E} D + D^R_1$	$a_6 = \frac{k^A_E}{\Omega} n^A n^R_1$	$\frac{k^A_E}{\Omega}$	1	1
7	$D^A + D^R_{12} \xrightarrow{k^A_E} D + D^R_{12}$	$a_7 = \frac{k^A_E}{\Omega} n^A n^R_{12}$	$\frac{k^A_E}{\Omega}$	1	1
8	$D^A + D^R_2 \xrightarrow{k^A_E} D + D^R_2$	$a_8 = \frac{k^A_E}{\Omega} n^A n^R_2$	$\frac{k^A_E}{\Omega}$	1	1
9	$D^A + D^R_{12} \xrightarrow{k^A_E} D + D^R_{12}$	$a_9 = \frac{k^A_E}{\Omega} n^A n^R_{12}$	$\frac{k^A_E}{\Omega}$	1	1
10	$D \xrightarrow{k^1_{W0}} D^R_1$	$a_{10} = k^1_{W0} n^D$	k^1_{W0}	3.5	3.5
11	$D \xrightarrow{k^1_W} D^R_1$	$a_{11} = k^1_W n^D$	k^1_W	0<br>0	0 (upper plots)<br>0, 50 (lower plots)
12	$D^R_1 \xrightarrow{k'_T} D$	$a_{12} = k'_T n^R_1$	k'_T	4.7, 0.5 (upper plots)<br>4.7, 0.47 (lower plots)	2.4
13	$D^R_1 \xrightarrow{\delta'} D$	$a_{13} = \delta' n^R_1$	δ'	4.7, 0.5 (upper plots)<br>4.7, 0.47 (lower plots)	2.4
14	$D + D^R_2 \xrightarrow{k'_M} D^R_1 + D^R_2$	$a_{14} = \frac{k'_M}{\Omega} n^D n^R_2$	$\frac{k'_M}{\Omega}$	0.2	0.2
15	$D + D^R_{12} \xrightarrow{k'_M} D^R_1 + D^R_{12}$	$a_{15} = \frac{k'_M}{\Omega} n^D n^R_{12}$	$\frac{k'_M}{\Omega}$	0.2	0.2
16	$D^R_1 + D^A \xrightarrow{k'^*_T} D + D^A$	$a_{16} = \frac{k'^*_T}{\Omega} n^R_1 n^A$	$\frac{k'^*_T}{\Omega}$	1 (upper plots)<br>1,0.1 (lower plots)	1, 0.8
17	$D \xrightarrow{k^2_{W0}} D^R_2$	$a_{17} = k^2_{W0} n^D$	k^2_{W0}	3.5	3.5
18	$D \xrightarrow{k^2_W} D^R_2$	$a_{18} = k^2_W n^D$	k^2_W	0<br>0	0 (upper plots)<br>0, 50 (lower plots)
19	$D^R_2 \xrightarrow{\bar{k}^R_E} D$	$a_{19} = \bar{k}^R_E n^R_2$	$\bar{k}^R_E$	4.7, 0.5 (upper plots)<br>4.7 (lower plots)	3
20	$D^R_2 \xrightarrow{\delta} D$	$a_{20} = \delta n^R_2$	δ	4.7, 0.5 (upper plots)<br>4.7 (lower plots)	3
21	$D + D^R_2 \xrightarrow{k_M} D^R_2 + D^R_2$	$a_{21} = \frac{k_M}{\Omega} n^D n^R_2$	$\frac{k_M}{\Omega}$	0.2	0.2
22	$D + D^R_{12} \xrightarrow{k_M} D^R_2 + D^R_{12}$	$a_{22} = \frac{k_M}{\Omega} n^D n^R_{12}$	$\frac{k_M}{\Omega}$	0.2	0.2
23	$D + D^R_1 \xrightarrow{\bar{k}_M} D^R_2 + D^R_1$	$a_{23} = \frac{\bar{k}_M}{\Omega} n^D n^R_1$	$\frac{\bar{k}_M}{\Omega}$	0.2	0.2
24	$D + D^R_{12} \xrightarrow{\bar{k}_M} D^R_2 + D^R_{12}$	$a_{24} = \frac{\bar{k}_M}{\Omega} n^D n^R_{12}$	$\frac{\bar{k}_M}{\Omega}$	0.2	0.2
25	$D^R_2 + D^A \xrightarrow{k^R_E} D + D^A$	$a_{25} = \frac{k^R_E}{\Omega} n^R_2 n^A$	$\frac{k^R_E}{\Omega}$	1	1
26	$D^R_1 \xrightarrow{k^2_{W0}} D^R_{12}$	$a_{26} = k^2_{W0} n^R_1$	k^2_{W0}	3.5	3.5
27	$D^R_{12} \xrightarrow{\bar{k}^R_E} D^R_1$	$a_{27} = \bar{k}^R_E n^R_{12}$	$\bar{k}^R_E$	4.7, 0.5 (upper plots)<br>4.7 (lower plots)	3
28	$D^R_{12} \xrightarrow{\delta} D^R_1$	$a_{28} = \delta n^R_{12}$	δ	4.7, 0.5 (upper plots)<br>4.7 (lower plots)	3
29	$D^R_1 + D^R_2 \xrightarrow{k_M} D^R_{12} + D^R_2$	$a_{29} = \frac{k_M}{\Omega} n^R_1 n^R_2$	$\frac{k_M}{\Omega}$	0.2	0.2
30	$D^R_1 + D^R_{12} \xrightarrow{k_M} D^R_{12} + D^R_{12}$	$a_{30} = \frac{k_M}{\Omega} n^R_1 n^R_{12}$	$\frac{k_M}{\Omega}$	0.2	0.2
31	$D^R_1 + D^R_1 \xrightarrow{\bar{k}_M} D^R_{12} + D^R_1$	$a_{31} = \frac{\bar{k}_M}{\Omega} \frac{n^R_1(n^R_1-1)}{2}$	$\frac{\bar{k}_M}{\Omega}$	0.2	0.2
32	$D^R_1 + D^R_{12} \xrightarrow{\bar{k}_M} D^R_{12} + D^R_{12}$	$a_{32} = \frac{\bar{k}_M}{\Omega} n^R_1 n^R_{12}$	$\frac{\bar{k}_M}{\Omega}$	0.2	0.2
33	$D^R_{12} + D^A \xrightarrow{k^R_E} D^R_1 + D^A$	$a_{33} = \frac{k^R_E}{\Omega} n^R_{12} n^A$	$\frac{k^R_E}{\Omega}$	1	1
34	$D^R_2 \xrightarrow{k^1_{W0}} D^R_{12}$	$a_{34} = k^1_{W0} n^R_2$	k^1_{W0}	3.5	3.5
35	$D^R_{12} \xrightarrow{k'_T} D^R_2$	$a_{35} = k'_T n^R_{12}$	k'_T	4.7, 0.5 (upper plots)<br>4.7, 0.47 (lower plots)	2.4
36	$D^R_{12} \xrightarrow{\delta'} D^R_2$	$a_{36} = \delta' n^R_{12}$	δ'	4.7, 0.5 (upper plots)<br>4.7, 0.47 (lower plots)	2.4
37	$D^R_2 + D^R_2 \xrightarrow{k'_M} D^R_{12} + D^R_2$	$a_{37} = \frac{k'_M}{\Omega} \frac{n^R_2(n^R_2-1)}{2}$	$\frac{k'_M}{\Omega}$	0.2	0.2
38	$D^R_2 + D^R_{12} \xrightarrow{k'_M} D^R_{12} + D^R_{12}$	$a_{38} = \frac{k'_M}{\Omega} n^R_2 n^R_{12}$	$\frac{k'_M}{\Omega}$	0.2	0.2
39	$D^R_{12} + D^A \xrightarrow{k'^*_T} D^R_2 + D^A$	$a_{39} = \frac{k'^*_T}{\Omega} n^R_{12} n^A$	$\frac{k'^*_T}{\Omega}$	1 (upper plots)<br>1,0.1 (lower plots)	1, 0.8

Table A: **Reactions and parameter values used to generate the plots in Fig 4A,4B.**

R_j	**Reaction**	**Prop.Func.**(a_j)	**Param.**	**Value** (h^{-1}) Fig 4C	**Value** (h^{-1}) Fig 4D
1	$D \xrightarrow{k_{W0}^A} D^A$	$a_1 = k_{W0}^A n^D$	k_{W0}^A	5	5
2	$D \xrightarrow{k_W^A} D^A$	$a_2 = k_W^A n^D$	k_W^A	0	0
3	$D^A \xrightarrow{\bar{k}_E^A} D$	$a_3 = \bar{k}_E^A n^A$	$\bar{k}_E^A$	9, 3	9, 3
4	$D^A \xrightarrow{\delta} D$	$a_4 = \delta n^A$	δ	9, 3	9, 3
5	$D + D^A \xrightarrow{k_M^A} D^A + D^A$	$a_5 = \frac{k_M^A}{\Omega} n^D n^A$	$\frac{k_M^A}{\Omega}$	1	1
6	$D^A + D_1^R \xrightarrow{k_E^A} D + D_1^R$	$a_6 = \frac{k_E^A}{\Omega} n^A n_1^R$	$\frac{k_E^A}{\Omega}$	1	1
7	$D^A + D_{12}^R \xrightarrow{k_E^A} D + D_{12}^R$	$a_7 = \frac{k_E^A}{\Omega} n^A n_{12}^R$	$\frac{k_E^A}{\Omega}$	1	1
8	$D^A + D_2^R \xrightarrow{k_E^A} D + D_2^R$	$a_8 = \frac{k_E^A}{\Omega} n^A n_2^R$	$\frac{k_E^A}{\Omega}$	1	1
9	$D^A + D_{12}^R \xrightarrow{k_E^A} D + D_{12}^R$	$a_9 = \frac{k_E^A}{\Omega} n^A n_{12}^R$	$\frac{k_E^A}{\Omega}$	1	1
10	$D \xrightarrow{k_{W0}^1} D_1^R$	$a_{10} = k_{W0}^1 n^D$	k_{W0}^1	5	5
11	$D \xrightarrow{k_W^1} D_1^R$	$a_{11} = k_W^1 n^D$	k_W^1	0	0
12	$D_1^R \xrightarrow{k_T^{'}} D$	$a_{12} = k_T^{'} n_1^R$	$k_T^{'}$	9, 4.5<br>3, 1.5	9, 4.5 (left side)<br>3, 1.5 (right side)
13	$D_1^R \xrightarrow{\delta^{'}} D$	$a_{13} = \delta^{'} n_1^R$	$\delta^{'}$	9, 4.5<br>3, 1.5	9, 4.5 (left side)<br>3, 1.5 (right side)
14	$D + D_2^R \xrightarrow{k_M^{'}} D_1^R + D_2^R$	$a_{14} = \frac{k_M^{'}}{\Omega} n^D n_2^R$	$\frac{k_M^{'}}{\Omega}$	0.2	0.2
15	$D + D_{12}^R \xrightarrow{k_M^{'}} D_1^R + D_{12}^R$	$a_{15} = \frac{k_M^{'}}{\Omega} n^D n_{12}^R$	$\frac{k_M^{'}}{\Omega}$	0.2	0.2
16	$D_1^R + D^A \xrightarrow{k_T^{'*}} D + D^A$	$a_{16} = \frac{k_T^{'*}}{\Omega} n_1^R n^A$	$\frac{k_T^{'*}}{\Omega}$	1,0.5	1,0.5
17	$D \xrightarrow{k_{W0}^2} D_2^R$	$a_{17} = k_{W0}^2 n^D$	k_{W0}^2	5	5
18	$D \xrightarrow{k_W^2} D_2^R$	$a_{18} = k_W^2 n^D$	k_W^2	0	0
19	$D_2^R \xrightarrow{\bar{k}_E^R} D$	$a_{19} = \bar{k}_E^R n_2^R$	$\bar{k}_E^R$	9, 3	9, 3
20	$D_2^R \xrightarrow{\delta} D$	$a_{20} = \delta n_2^R$	δ	9, 3	9, 3
21	$D + D_2^R \xrightarrow{k_M} D_2^R + D_2^R$	$a_{21} = \frac{k_M}{\Omega} n^D n_2^R$	$\frac{k_M}{\Omega}$	0.2	0.2
22	$D + D_{12}^R \xrightarrow{k_M} D_2^R + D_{12}^R$	$a_{22} = \frac{k_M}{\Omega} n^D n_{12}^R$	$\frac{k_M}{\Omega}$	0.2	0.2
23	$D + D_1^R \xrightarrow{\bar{k}_M} D_2^R + D_1^R$	$a_{23} = \frac{\bar{k}_M}{\Omega} n^D n_1^R$	$\frac{\bar{k}_M}{\Omega}$	0.2	0.2
24	$D + D_{12}^R \xrightarrow{\bar{k}_M} D_2^R + D_{12}^R$	$a_{24} = \frac{\bar{k}_M}{\Omega} n^D n_{12}^R$	$\frac{\bar{k}_M}{\Omega}$	0.2	0.2
25	$D_2^R + D^A \xrightarrow{k_E^R} D + D^A$	$a_{25} = \frac{k_E^R}{\Omega} n_2^R n^A$	$\frac{k_E^R}{\Omega}$	1	1
26	$D_1^R \xrightarrow{k_{W0}^2} D_{12}^R$	$a_{26} = k_{W0}^2 n_1^R$	k_{W0}^2	5	5
27	$D_{12}^R \xrightarrow{\bar{k}_E^R} D_1^R$	$a_{27} = \bar{k}_E^R n_{12}^R$	$\bar{k}_E^R$	9, 3	9, 3
28	$D_{12}^R \xrightarrow{\delta} D_1^R$	$a_{28} = \delta n_{12}^R$	δ	9, 3	9, 3
29	$D_1^R + D_2^R \xrightarrow{k_M} D_{12}^R + D_2^R$	$a_{29} = \frac{k_M}{\Omega} n_1^R n_2^R$	$\frac{k_M}{\Omega}$	0.2	0.2
30	$D_1^R + D_{12}^R \xrightarrow{k_M} D_{12}^R + D_{12}^R$	$a_{30} = \frac{k_M}{\Omega} n_1^R n_{12}^R$	$\frac{k_M}{\Omega}$	0.2	0.2
31	$D_1^R + D_1^R \xrightarrow{\bar{k}_M} D_{12}^R + D_1^R$	$a_{31} = \frac{\bar{k}_M}{\Omega} \frac{n_1^R(n_1^R-1)}{2}$	$\frac{\bar{k}_M}{\Omega}$	0.2	0.2
32	$D_1^R + D_{12}^R \xrightarrow{\bar{k}_M} D_{12}^R + D_{12}^R$	$a_{32} = \frac{\bar{k}_M}{\Omega} n_1^R n_{12}^R$	$\frac{\bar{k}_M}{\Omega}$	0.2	0.2
33	$D_{12}^R + D^A \xrightarrow{k_E^R} D_1^R + D^A$	$a_{33} = \frac{k_E^R}{\Omega} n_{12}^R n^A$	$\frac{k_E^R}{\Omega}$	1	1
34	$D_2^R \xrightarrow{k_{W0}^1} D_{12}^R$	$a_{34} = k_{W0}^1 n_2^R$	k_{W0}^1	5	5
35	$D_{12}^R \xrightarrow{k_T^{'}} D_2^R$	$a_{35} = k_T^{'} n_{12}^R$	$k_T^{'}$	9, 4.5<br>3, 1.5	9, 4.5 (left side)<br>3, 1.5 (right side)
36	$D_{12}^R \xrightarrow{\delta^{'}} D_2^R$	$a_{36} = \delta^{'} n_{12}^R$	$\delta^{'}$	9, 4.5<br>3, 1.5	9, 4.5 (left side)<br>3, 1.5 (right side)
37	$D_2^R + D_2^R \xrightarrow{k_M^{'}} D_{12}^R + D_2^R$	$a_{37} = \frac{k_M^{'}}{\Omega} \frac{n_2^R(n_2^R-1)}{2}$	$\frac{k_M^{'}}{\Omega}$	0.2	0.2
38	$D_2^R + D_{12}^R \xrightarrow{k_M^{'}} D_{12}^R + D_{12}^R$	$a_{38} = \frac{k_M^{'}}{\Omega} n_2^R n_{12}^R$	$\frac{k_M^{'}}{\Omega}$	0.2	0.2
39	$D_{12}^R + D^A \xrightarrow{k_T^{'*}} D_2^R + D^A$	$a_{39} = \frac{k_T^{'*}}{\Omega} n_{12}^R n^A$	$\frac{k_T^{'*}}{\Omega}$	1,0.5	1,0.5

Table B: **Reactions and parameter values used to generate the plots in Fig 4C,4D.**

R_j	Reaction	Prop.Func.(a_j)	Param.	Value (h^{-1})
1	$D \xrightarrow{k_{W0}^A} D^A$	$a_1 = k_{W0}^A n^D$	k_{W0}^A	5
2	$D \xrightarrow{k_W^A} D^A$	$a_2 = k_W^A n^D$	k_W^A	160
3	$D^A \xrightarrow{\bar{k}_E^A} D$	$a_3 = \bar{k}_E^A n^A$	$\bar{k}_E^A$	4,6
4	$D^A \xrightarrow{\delta} D$	$a_4 = \delta n^A$	δ	4,6
5	$D + D^A \xrightarrow{k_M^A} D^A + D^A$	$a_5 = \frac{k_M^A}{\Omega} n^D n^A$	$\frac{k_M^A}{\Omega}$	1
6	$D^A + D_1^R \xrightarrow{k_E^A} D + D_1^R$	$a_6 = \frac{k_E^A}{\Omega} n^A n_1^R$	$\frac{k_E^A}{\Omega}$	1
7	$D^A + D_{12}^R \xrightarrow{k_E^A} D + D_{12}^R$	$a_7 = \frac{k_E^A}{\Omega} n^A n_{12}^R$	$\frac{k_E^A}{\Omega}$	1
8	$D^A + D_2^R \xrightarrow{k_E^A} D + D_2^R$	$a_8 = \frac{k_E^A}{\Omega} n^A n_2^R$	$\frac{k_E^A}{\Omega}$	1
9	$D^A + D_{12}^R \xrightarrow{k_E^A} D + D_{12}^R$	$a_9 = \frac{k_E^A}{\Omega} n^A n_{12}^R$	$\frac{k_E^A}{\Omega}$	1
10	$D \xrightarrow{k_{W0}^1} D_1^R$	$a_{10} = k_{W0}^1 n^D$	k_{W0}^1	5
11	$D \xrightarrow{k_W^1} D_1^R$	$a_{11} = k_W^1 n^D$	k_W^1	0
12	$D_1^R \xrightarrow{k_T^{'}} D$	$a_{12} = k_T^{'} n_1^R$	$k_T^{'}$	0.4,0.6
13	$D_1^R \xrightarrow{\delta^{'}} D$	$a_{13} = \delta^{'} n_1^R$	$\delta^{'}$	0.4,0.6
14	$D + D_2^R \xrightarrow{k_M^{'}} D_1^R + D_2^R$	$a_{14} = \frac{k_M^{'}}{\Omega} n^D n_2^R$	$\frac{k_M^{'}}{\Omega}$	0.2
15	$D + D_{12}^R \xrightarrow{k_M^{'}} D_1^R + D_{12}^R$	$a_{15} = \frac{k_M^{'}}{\Omega} n^D n_{12}^R$	$\frac{k_M^{'}}{\Omega}$	0.2
16	$D_1^R + D^A \xrightarrow{k_T^{'*}} D + D^A$	$a_{16} = \frac{k_T^{'*}}{\Omega} n_1^R n^A$	$\frac{k_T^{'*}}{\Omega}$	0.1
17	$D \xrightarrow{k_{W0}^2} D_2^R$	$a_{17} = k_{W0}^2 n^D$	k_{W0}^2	5
18	$D \xrightarrow{k_W^2} D_2^R$	$a_{18} = k_W^2 n^D$	k_W^2	0
19	$D_2^R \xrightarrow{\bar{k}_E^R} D$	$a_{19} = \bar{k}_E^R n_2^R$	$\bar{k}_E^R$	4,6
20	$D_2^R \xrightarrow{\delta} D$	$a_{20} = \delta n_2^R$	δ	4.6
21	$D + D_2^R \xrightarrow{k_M} D_2^R + D_2^R$	$a_{21} = \frac{k_M}{\Omega} n^D n_2^R$	$\frac{k_M}{\Omega}$	0.2
22	$D + D_{12}^R \xrightarrow{k_M} D_2^R + D_{12}^R$	$a_{22} = \frac{k_M}{\Omega} n^D n_{12}^R$	$\frac{k_M}{\Omega}$	0.2
23	$D + D_1^R \xrightarrow{\bar{k}_M} D_2^R + D_1^R$	$a_{23} = \frac{\bar{k}_M}{\Omega} n^D n_1^R$	$\frac{\bar{k}_M}{\Omega}$	0.2
24	$D + D_{12}^R \xrightarrow{\bar{k}_M} D_2^R + D_{12}^R$	$a_{24} = \frac{\bar{k}_M}{\Omega} n^D n_{12}^R$	$\frac{\bar{k}_M}{\Omega}$	0.2
25	$D_2^R + D^A \xrightarrow{k_E^R} D + D^A$	$a_{25} = \frac{k_E^R}{\Omega} n_2^R n^A$	$\frac{k_E^R}{\Omega}$	1
26	$D_1^R \xrightarrow{k_{W0}^2} D_{12}^R$	$a_{26} = k_{W0}^2 n_1^R$	k_{W0}^2	5
27	$D_{12}^R \xrightarrow{\bar{k}_E^R} D_1^R$	$a_{27} = \bar{k}_E^R n_{12}^R$	$\bar{k}_E^R$	4,6
28	$D_{12}^R \xrightarrow{\delta} D_1^R$	$a_{28} = \delta n_{12}^R$	δ	4,6
29	$D_1^R + D_2^R \xrightarrow{k_M} D_{12}^R + D_2^R$	$a_{29} = \frac{k_M}{\Omega} n_1^R n_2^R$	$\frac{k_M}{\Omega}$	0.2
30	$D_1^R + D_{12}^R \xrightarrow{k_M} D_{12}^R + D_{12}^R$	$a_{30} = \frac{k_M}{\Omega} n_1^R n_{12}^R$	$\frac{k_M}{\Omega}$	0.2
31	$D_1^R + D_1^R \xrightarrow{\bar{k}_M} D_{12}^R + D_1^R$	$a_{31} = \frac{\bar{k}_M}{\Omega} \frac{n_1^R(n_1^R-1)}{2}$	$\frac{\bar{k}_M}{\Omega}$	0.2
32	$D_1^R + D_{12}^R \xrightarrow{\bar{k}_M} D_{12}^R + D_{12}^R$	$a_{32} = \frac{\bar{k}_M}{\Omega} n_1^R n_{12}^R$	$\frac{\bar{k}_M}{\Omega}$	0.2
33	$D_{12}^R + D^A \xrightarrow{k_E^R} D_1^R + D^A$	$a_{33} = \frac{k_E^R}{\Omega} n_{12}^R n^A$	$\frac{k_E^R}{\Omega}$	1
34	$D_2^R \xrightarrow{k_{W0}^1} D_{12}^R$	$a_{34} = k_{W0}^1 n_2^R$	k_{W0}^1	5
35	$D_{12}^R \xrightarrow{k_T^{'}} D_2^R$	$a_{35} = k_T^{'} n_{12}^R$	$k_T^{'}$	0.4,0.6
36	$D_{12}^R \xrightarrow{\delta^{'}} D_2^R$	$a_{36} = \delta^{'} n_{12}^R$	$\delta^{'}$	0.4,0.6
37	$D_2^R + D_2^R \xrightarrow{k_M^{'}} D_{12}^R + D_2^R$	$a_{37} = \frac{k_M^{'}}{\Omega} \frac{n_2^R(n_2^R-1)}{2}$	$\frac{k_M^{'}}{\Omega}$	0.2
38	$D_2^R + D_{12}^R \xrightarrow{k_M^{'}} D_{12}^R + D_{12}^R$	$a_{38} = \frac{k_M^{'}}{\Omega} n_2^R n_{12}^R$	$\frac{k_M^{'}}{\Omega}$	0.2
39	$D_{12}^R + D^A \xrightarrow{k_T^{'*}} D_2^R + D^A$	$a_{39} = \frac{k_T^{'*}}{\Omega} n_{12}^R n^A$	$\frac{k_T^{'*}}{\Omega}$	0.1

Table C: **Reactions and parameter values used to generate the plots in Fig 4F.**

**Param.**	**Value** 1st plot	**Value** 2nd plot	**Value** 3rd plot	**Value** 4th plot
u_0^A	0.001	0.001	0.001	0.001
u^A	0	0	0	0
u_0^R	0.001	0.001	0.001	0.001
u^R	0	0	0	0
α	1	1	1	1
ϵ	3	0.3	0.3	0.3
$\epsilon^{'}$	0.3	0.3	0.3	0.3
b	1	1	1	1
μ	1	1	0.1	2

Table D: **Parameter values relative to the plots in Fig D, going from left to right.**

**Param.**	**Value**
$\bar{D}^R(0)$	0.3
$\bar{D}^A(0)$	0.7
u_0^A	0.1
u^A	0
u_0^R	0.1
u^R	0
α	1
$\epsilon^{'}$	0, 0.1, 0.01
c	1
b	1
μ	1

Table E: **Parameter values relative to the plots in Fig E.**

R_j	Reaction	Prop.Func.(a_j)	Param.	Value (h^{-1}) Fig FA	Value (h^{-1}) Fig FB
1	$D \xrightarrow{k^A_{W0}} D^A$	$a_1 = k^A_{W0} n^D$	k^A_{W0}	5	5
2	$D \xrightarrow{k^A_W} D^A$	$a_2 = k^A_W n^D$	k^A_W	0<br>0	0, 5, 50 (upper plots)<br>0 (lower plots)
3	$D^A \xrightarrow{\bar{k}^A_E} D$	$a_3 = \bar{k}^A_E n^A$	$\bar{k}^A_E$	10,5,0.5	2.5
4	$D^A \xrightarrow{\delta} D$	$a_4 = \delta n^A$	δ	10,5,0.5	2.5
5	$D^A + D^R \xrightarrow{k^A_E} D + D^R$	$a_5 = \frac{k^A_E}{\Omega} n^A n^R$	$\frac{k^A_E}{\Omega}$	1	1
6	$D + D^A \xrightarrow{k^A_M} D^A + D^A$	$a_6 = \frac{k^A_M}{\Omega} n^D n^A$	$\frac{k^A_M}{\Omega}$	1	1
7	$D \xrightarrow{k^R_{W0}} D^R$	$a_7 = k^R_{W0} n^D$	k^R_{W0}	5	5
8	$D \xrightarrow{k^R_W} D^R$	$a_8 = k^R_W n^D$	k^R_W	0<br>0	0 (upper plots)<br>0, 5, 50 (lower plots)
9	$D^R \xrightarrow{\bar{k}^R_E} D$	$a_9 = \bar{k}^R_E n^R$	$\bar{k}^R_E$	100, 12, 10, 8.3, 0.1(left plots)<br>50, 6.5, 5, 4.15, 0.05(central plots)<br>5, 0.65, 0.5, 0.415, 0.005(right plots)	2.5, 2.075
10	$D^R \xrightarrow{\delta} D$	$a_{10} = \delta n^R$	δ	10,5,0.5	2.5, 2.075
11	$D^R + D^A \xrightarrow{k^R_E} D + D^A$	$a_{11} = \frac{k^R_E}{\Omega} n^R n^A$	$\frac{k^R_E}{\Omega}$	10, 1.2, 1, 0.83, 0.1	1, 0.83
12	$D + D^R \xrightarrow{k^R_M} D^R + D^R$	$a_{12} = \frac{k^R_M}{\Omega} n^D n^R$	$\frac{k^R_M}{\Omega}$	1	1

Table F: **Reactions and parameter values used to generate the plots in Fig F.**

R_j	Reaction	Prop.Func.(a_j)	Param.	Value (h^{-1}) 1st plots	Value (h^{-1}) 2nd plots	Value (h^{-1}) 3rd plots	Value (h^{-1}) 4th plots	Value (h^{-1}) 5th plots	Value (h^{-1}) 6th plots
1	$D \xrightarrow{k^A_{W0}} D^A$	$a_1 = k^A_{W0} n^D$	k^A_{W0}	5	5	5	5	5	5
2	$D \xrightarrow{k^A_W} D^A$	$a_2 = k^A_W n^D$	k^A_W	0	0	0	0	0	0
3	$D^A \xrightarrow{\bar{k}^A_E} D$	$a_3 = \bar{k}^A_E n^A$	$\bar{k}^A_E$	5, 2.5, 0.5, 0.05	5	10, 5, 0.5, 0.05	5	12.5, 5, 0.5, 0.05	5
4	$D^A \xrightarrow{\delta} D$	$a_4 = \delta n^A$	δ	5, 2.5, 0.5, 0.05	5	10, 5, 0.5, 0.05	5	12.5, 5, 0.5, 0.05	5
5	$D^A + D^R \xrightarrow{k^A_E} D + D^R$	$a_5 = \frac{k^A_E}{\Omega} n^A n^R$	$\frac{k^A_E}{\Omega}$	0.2	0.2	1	0.5, 1, 10, 100	10	4, 10, 100, 1000
6	$D + D^A \xrightarrow{k^A_M} D^A + D^A$	$a_6 = \frac{k^A_M}{\Omega} n^D n^A$	$\frac{k^A_M}{\Omega}$	1	1, 2, 10, 100	1	0.5, 1, 10, 100	1	0.4, 1, 10, 100
7	$D \xrightarrow{k^R_{W0}} D^R$	$a_7 = k^R_{W0} n^D$	k^R_{W0}	5	5	5	5	5	5
8	$D \xrightarrow{k^R_W} D^R$	$a_8 = k^R_W n^D$	k^R_W	0	0	0	0	0	0
9	$D^R \xrightarrow{\bar{k}^R_E} D$	$a_9 = \bar{k}^R_E n^R$	$\bar{k}^R_E$	5, 2.5, 0.5, 0.05	5	10, 5, 0.5, 0.05	5	5, 0.5, 0.05	5
10	$D^R \xrightarrow{\delta} D$	$a_{10} = \delta n^R$	δ	5, 2.5, 0.5, 0.05	5	10, 5, 0.5, 0.05	5	12.5, 5, 0.5, 0.05	5
11	$D^R + D^A \xrightarrow{k^R_E} D + D^A$	$a_{11} = \frac{k^R_E}{\Omega} n^R n^A$	$\frac{k^R_E}{\Omega}$	0.2	0.2	1	0.5, 1, 10, 100	10	4, 10, 100, 1000
12	$D + D^R \xrightarrow{k^R_M} D^R + D^R$	$a_{12} = \frac{k^R_M}{\Omega} n^D n^R$	$\frac{k^R_M}{\Omega}$	1	1, 2, 10, 100	1	0.5, 1, 10, 100	1	0.4, 1, 10, 100

Table G: **Reactions and parameter values used to generate the plots in Fig G, going from the top to the bottom.**

R_j	Reaction	Prop.Func.(a_j)	Param.	Value (h^{-1}) left plots	Value (h^{-1}) central plots	Value (h^{-1}) right plots
1	$D \xrightarrow{k_{W0}^A} D^A$	$a_1 = k_{W0}^A n^D$	k_{W0}^A	5	5	5
2	$D \xrightarrow{k_W^A} D^A$	$a_2 = k_W^A n^D$	k_W^A	0	0	0
3	$D^A \xrightarrow{\bar{k}_E^A} D$	$a_3 = \bar{k}_E^A n^A$	$\bar{k}_E^A$	5, 0.5	5, 0.5	5, 0.5
4	$D^A \xrightarrow{\delta} D$	$a_4 = \delta n^A$	δ	5, 0.5	5, 0.5	5, 0.5
5	$D^A + D^R \xrightarrow{k_E^A} D + D^R$	$a_5 = \frac{k_E^A}{\Omega} n^A n^R$	$\frac{k_E^A}{\Omega}$	0.2	1	10
6	$D + D^A \xrightarrow{k_M^A} D^A + D^A$	$a_6 = \frac{k_M^A}{\Omega} n^D n^A$	$\frac{k_M^A}{\Omega}$	1	1	1
7	$D \xrightarrow{k_{W0}^R} D^R$	$a_7 = k_{W0}^R n^D$	k_{W0}^R	5	5	5
8	$D \xrightarrow{k_W^R} D^R$	$a_8 = k_W^R n^D$	k_W^R	0	0	0
9	$D^R \xrightarrow{\bar{k}_E^R} D$	$a_9 = \bar{k}_E^R n^R$	$\bar{k}_E^R$	50, 5, 0.5<br>5, 0.5, 0.05	50, 5, 0.5<br>5, 0.5, 0.05	50, 5, 0.5 (left plots)<br>5, 0.5, 0.05 (right plots)
10	$D^R \xrightarrow{\delta} D$	$a_{10} = \delta n^R$	δ	5, 0.5	5, 0.5	5, 0.5
11	$D^R + D^A \xrightarrow{k_E^R} D + D^A$	$a_{11} = \frac{k_E^R}{\Omega} n^R n^A$	$\frac{k_E^R}{\Omega}$	2, 0.2, 0.02	10, 1, 0.1	100, 10, 1
12	$D + D^R \xrightarrow{k_M^R} D^R + D^R$	$a_{12} = \frac{k_M^R}{\Omega} n^D n^R$	$\frac{k_M^R}{\Omega}$	1	1	1

Table H: **Reactions and parameter values used to generate the plots in Fig H.**

R_j	Reaction	Prop.Func.(a_j)	Param.	Value (h^{-1}) left plots	Value (h^{-1}) right plots
1	$D \xrightarrow{k_{W0}^A} D^A$	$a_1 = k_{W0}^A n^D$	k_{W0}^A	5	5
2	$D \xrightarrow{k_W^A} D^A$	$a_2 = k_W^A n^D$	k_W^A	0, 5, 50<br>0	0, 5, 50 (upper plots)<br>0 (lower plots)
3	$D^A \xrightarrow{\bar{k}_E^A} D$	$a_3 = \bar{k}_E^A n^A$	$\bar{k}_E^A$	2.5	2.5
4	$D^A \xrightarrow{\delta} D$	$a_4 = \delta n^A$	δ	2.5	2.5
5	$D^A + D^R \xrightarrow{k_E^A} D + D^R$	$a_5 = \frac{k_E^A}{\Omega} n^A n^R$	$\frac{k_E^A}{\Omega}$	0.2	10
6	$D + D^A \xrightarrow{k_M^A} D^A + D^A$	$a_6 = \frac{k_M^A}{\Omega} n^D n^A$	$\frac{k_M^A}{\Omega}$	1	1
7	$D \xrightarrow{k_{W0}^R} D^R$	$a_7 = k_{W0}^R n^D$	k_{W0}^R	5	5
8	$D \xrightarrow{k_W^R} D^R$	$a_8 = k_W^R n^D$	k_W^R	0<br>0, 5, 50	0 (upper plots)<br>0, 5, 50 (lower plots)
9	$D^R \xrightarrow{\bar{k}_E^R} D$	$a_9 = \bar{k}_E^R n^R$	$\bar{k}_E^R$	2.5,2.075	2.5,2.075
10	$D^R \xrightarrow{\delta} D$	$a_{10} = \delta n^R$	δ	2.5	2.5
11	$D^R + D^A \xrightarrow{k_E^R} D + D^A$	$a_{11} = \frac{k_E^R}{\Omega} n^R n^A$	$\frac{k_E^R}{\Omega}$	0.2,0.166	10,8.3
12	$D + D^R \xrightarrow{k_M^R} D^R + D^R$	$a_{12} = \frac{k_M^R}{\Omega} n^D n^R$	$\frac{k_M^R}{\Omega}$	1	1

Table I: **Reactions and parameter values used to generate the plots in Fig I.**

**Param.**	**Value**
$\bar{D}^R_{12}(0)$	0.7
$\bar{D}^A(0)$	0.3
u^A_0	0.1
u^A	0
u^R_{10}	0.1
u^R_1	0
u^R_{20}	0.1
u^R_2	0
α	1
$\bar{\alpha}$	1
$\alpha^{'}$	1
$\epsilon^{'}$	1, 0.1, 0.01
c	1
b	1
μ	1
β	1
$\mu^{'}$	1

Table J: **Parameter values relative to the plots in Fig J.**

R_j	Reaction	Prop.Func.(a_j)	Param.	Value (h^{-1})
1	$\mathrm{D} \xrightarrow{k_{W0}^A} \mathrm{D^A}$	$a_1 = k_{W0}^A n^D$	k_{W0}^A	3.5
2	$\mathrm{D} \xrightarrow{k_W^A} \mathrm{D^A}$	$a_2 = k_W^A n^D$	k_W^A	0
3	$\mathrm{D^A} \xrightarrow{\bar{k}_E^A} \mathrm{D}$	$a_3 = \bar{k}_E^A n^A$	$\bar{k}_E^A$	25
4	$\mathrm{D^A} \xrightarrow{\delta} \mathrm{D}$	$a_4 = \delta n^A$	δ	25
5	$\mathrm{D} + \mathrm{D^A} \xrightarrow{k_M^A} \mathrm{D^A} + \mathrm{D^A}$	$a_5 = \frac{k_M^A}{\Omega} n^D n^A$	$\frac{k_M^A}{\Omega}$	1
6	$\mathrm{D^A} + \mathrm{D_1^R} \xrightarrow{k_E^A} \mathrm{D} + \mathrm{D_1^R}$	$a_6 = \frac{k_E^A}{\Omega} n^A n_1^R$	$\frac{k_E^A}{\Omega}$	1
7	$\mathrm{D^A} + \mathrm{D_{12}^R} \xrightarrow{k_E^A} \mathrm{D} + \mathrm{D_{12}^R}$	$a_7 = \frac{k_E^A}{\Omega} n^A n_{12}^R$	$\frac{k_E^A}{\Omega}$	1
8	$\mathrm{D^A} + \mathrm{D_2^R} \xrightarrow{k_E^A} \mathrm{D} + \mathrm{D_2^R}$	$a_8 = \frac{k_E^A}{\Omega} n^A n_2^R$	$\frac{k_E^A}{\Omega}$	1
9	$\mathrm{D^A} + \mathrm{D_{12}^R} \xrightarrow{k_E^A} \mathrm{D} + \mathrm{D_{12}^R}$	$a_9 = \frac{k_E^A}{\Omega} n^A n_{12}^R$	$\frac{k_E^A}{\Omega}$	1
10	$\mathrm{D} \xrightarrow{k_{W0}^1} \mathrm{D_1^R}$	$a_{10} = k_{W0}^1 n^D$	k_{W0}^1	3.5
11	$\mathrm{D} \xrightarrow{k_W^1} \mathrm{D_1^R}$	$a_{11} = k_W^1 n^D$	k_W^1	0
12	$\mathrm{D_1^R} \xrightarrow{k_T^{'}} \mathrm{D}$	$a_{12} = k_T^{'} n_1^R$	$k_T^{'}$	25
13	$\mathrm{D_1^R} \xrightarrow{\delta^{'}} \mathrm{D}$	$a_{13} = \delta^{'} n_1^R$	$\delta^{'}$	25
14	$\mathrm{D} + \mathrm{D_2^R} \xrightarrow{k_M^{'}} \mathrm{D_1^R} + \mathrm{D_2^R}$	$a_{14} = \frac{k_M^{'}}{\Omega} n^D n_2^R$	$\frac{k_M^{'}}{\Omega}$	1
15	$\mathrm{D} + \mathrm{D_{12}^R} \xrightarrow{k_M^{'}} \mathrm{D_1^R} + \mathrm{D_{12}^R}$	$a_{15} = \frac{k_M^{'}}{\Omega} n^D n_{12}^R$	$\frac{k_M^{'}}{\Omega}$	1
16	$\mathrm{D_1^R} + \mathrm{D^A} \xrightarrow{k_T^{'*}} \mathrm{D} + \mathrm{D^A}$	$a_{16} = \frac{k_T^{'*}}{\Omega} n_1^R n^A$	$\frac{k_T^{'*}}{\Omega}$	1
17	$\mathrm{D} \xrightarrow{k_{W0}^2} \mathrm{D_2^R}$	$a_{17} = k_{W0}^2 n^D$	k_{W0}^2	3.5
18	$\mathrm{D} \xrightarrow{k_W^2} \mathrm{D_2^R}$	$a_{18} = k_W^2 n^D$	k_W^2	0
19	$\mathrm{D_2^R} \xrightarrow{\bar{k}_E^R} \mathrm{D}$	$a_{19} = \bar{k}_E^R n_2^R$	$\bar{k}_E^R$	25
20	$\mathrm{D_2^R} \xrightarrow{\delta} \mathrm{D}$	$a_{20} = \delta n_2^R$	δ	25
21	$\mathrm{D} + \mathrm{D_2^R} \xrightarrow{k_M} \mathrm{D_2^R} + \mathrm{D_2^R}$	$a_{21} = \frac{k_M}{\Omega} n^D n_2^R$	$\frac{k_M}{\Omega}$	1
22	$\mathrm{D} + \mathrm{D_{12}^R} \xrightarrow{k_M} \mathrm{D_2^R} + \mathrm{D_{12}^R}$	$a_{22} = \frac{k_M}{\Omega} n^D n_{12}^R$	$\frac{k_M}{\Omega}$	1
23	$\mathrm{D} + \mathrm{D_1^R} \xrightarrow{\bar{k}_M} \mathrm{D_2^R} + \mathrm{D_1^R}$	$a_{23} = \frac{\bar{k}_M}{\Omega} n^D n_1^R$	$\frac{\bar{k}_M}{\Omega}$	1
24	$\mathrm{D} + \mathrm{D_{12}^R} \xrightarrow{\bar{k}_M} \mathrm{D_2^R} + \mathrm{D_{12}^R}$	$a_{24} = \frac{\bar{k}_M}{\Omega} n^D n_{12}^R$	$\frac{\bar{k}_M}{\Omega}$	1
25	$\mathrm{D_2^R} + \mathrm{D^A} \xrightarrow{k_E^R} \mathrm{D} + \mathrm{D^A}$	$a_{25} = \frac{k_E^R}{\Omega} n_2^R n^A$	$\frac{k_E^R}{\Omega}$	1
26	$\mathrm{D_1^R} \xrightarrow{k_{W0}^2} \mathrm{D_{12}^R}$	$a_{26} = k_{W0}^2 n_1^R$	k_{W0}^2	3.5
27	$\mathrm{D_{12}^R} \xrightarrow{\bar{k}_E^R} \mathrm{D_1^R}$	$a_{27} = \bar{k}_E^R n_{12}^R$	$\bar{k}_E^R$	25
28	$\mathrm{D_{12}^R} \xrightarrow{\delta} \mathrm{D_1^R}$	$a_{28} = \delta n_{12}^R$	δ	25
29	$\mathrm{D_1^R} + \mathrm{D_2^R} \xrightarrow{k_M} \mathrm{D_{12}^R} + \mathrm{D_2^R}$	$a_{29} = \frac{k_M}{\Omega} n_1^R n_2^R$	$\frac{k_M}{\Omega}$	0.2
30	$\mathrm{D_1^R} + \mathrm{D_{12}^R} \xrightarrow{k_M} \mathrm{D_{12}^R} + \mathrm{D_{12}^R}$	$a_{30} = \frac{k_M}{\Omega} n_1^R n_{12}^R$	$\frac{k_M}{\Omega}$	1
31	$\mathrm{D_1^R} + \mathrm{D_1^R} \xrightarrow{\bar{k}_M} \mathrm{D_{12}^R} + \mathrm{D_1^R}$	$a_{31} = \frac{\bar{k}_M}{\Omega} \frac{n_1^R(n_1^R-1)}{2}$	$\frac{\bar{k}_M}{\Omega}$	1
32	$\mathrm{D_1^R} + \mathrm{D_{12}^R} \xrightarrow{\bar{k}_M} \mathrm{D_{12}^R} + \mathrm{D_{12}^R}$	$a_{32} = \frac{\bar{k}_M}{\Omega} n_1^R n_{12}^R$	$\frac{\bar{k}_M}{\Omega}$	1
33	$\mathrm{D_{12}^R} + \mathrm{D^A} \xrightarrow{k_E^R} \mathrm{D_1^R} + \mathrm{D^A}$	$a_{33} = \frac{k_E^R}{\Omega} n_{12}^R n^A$	$\frac{k_E^R}{\Omega}$	1
34	$\mathrm{D_2^R} \xrightarrow{k_{W0}^1} \mathrm{D_{12}^R}$	$a_{34} = k_{W0}^1 n_2^R$	k_{W0}^1	3.5
35	$\mathrm{D_{12}^R} \xrightarrow{k_T^{'}} \mathrm{D_2^R}$	$a_{35} = k_T^{'} n_{12}^R$	$k_T^{'}$	25
36	$\mathrm{D_{12}^R} \xrightarrow{\delta^{'}} \mathrm{D_2^R}$	$a_{36} = \delta^{'} n_{12}^R$	$\delta^{'}$	25
37	$\mathrm{D_2^R} + \mathrm{D_2^R} \xrightarrow{k_M^{'}} \mathrm{D_{12}^R} + \mathrm{D_2^R}$	$a_{37} = \frac{k_M^{'}}{\Omega} \frac{n_2^R(n_2^R-1)}{2}$	$\frac{k_M^{'}}{\Omega}$	1
38	$\mathrm{D_2^R} + \mathrm{D_{12}^R} \xrightarrow{k_M^{'}} \mathrm{D_{12}^R} + \mathrm{D_{12}^R}$	$a_{38} = \frac{k_M^{'}}{\Omega} n_2^R n_{12}^R$	$\frac{k_M^{'}}{\Omega}$	1
39	$\mathrm{D_{12}^R} + \mathrm{D^A} \xrightarrow{k_T^{'*}} \mathrm{D_2^R} + \mathrm{D^A}$	$a_{39} = \frac{k_T^{'*}}{\Omega} n_{12}^R n^A$	$\frac{k_T^{'*}}{\Omega}$	1

Table K: **Reactions and parameter values used to generate the plots in Fig P.**

$\mathbf{R}_j$	**Reaction**	**Prop.Func.**(a_j)	**Param.**	**Value** ($\mathbf{h}^{-1}$) 1st plots	**Value** ($\mathbf{h}^{-1}$) 2nd plots	**Value** ($\mathbf{h}^{-1}$) 3rd plots	**Value** ($\mathbf{h}^{-1}$) 4th plots	**Value** ($\mathbf{h}^{-1}$) 5th plots	**Value** ($\mathbf{h}^{-1}$) 6th plots
1	$\mathrm{D} \xrightarrow{k_{W0}^{A}} \mathrm{D^A}$	$a_1 = k_{W0}^{A} n^D$	k_{W0}^{A}	3.5	3.5	3.5	3.5	3.5	3.5
2	$\mathrm{D} \xrightarrow{k_{W}^{A}} \mathrm{D^A}$	$a_2 = k_{W}^{A} n^D$	k_{W}^{A}	0	0	0	0	0	0
3	$\mathrm{D^A} \xrightarrow{\bar{k}_{E}^{A}} \mathrm{D}$	$a_3 = \bar{k}_{E}^{A} n^A$	$\bar{k}_{E}^{A}$	4, 3, 0.5, 0.05	4	4.7, 3, 0.5, 0.05	4.7	9, 3, 0.5, 0.05	9
4	$\mathrm{D^A} \xrightarrow{\delta} \mathrm{D}$	$a_4 = \delta n^A$	δ	4, 3, 0.5, 0.05	4	4.7, 3, 0.5, 0.05	4.7	9, 3, 0.5, 0.05	9
5	$\mathrm{D} + \mathrm{D^A} \xrightarrow{k_{M}^{A}} \mathrm{D^A} + \mathrm{D^A}$	$a_5 = \frac{k_M^A}{\Omega} n^D n^A$	$\frac{k_M^A}{\Omega}$	1	1, 1.33, 8, 80	1	1, 1.57, 9.4, 94	1	1, 3, 18, 180
6	$\mathrm{D^A} + \mathrm{D^R_1} \xrightarrow{k_{E}^{A}} \mathrm{D} + \mathrm{D^R_1}$	$a_6 = \frac{k_E^A}{\Omega} n^A n_1^R$	$\frac{k_E^A}{\Omega}$	0.2	0.2	1	1, 1.57, 9.4, 94	10	10, 30, 180, 1800
7	$\mathrm{D^A} + \mathrm{D^R_{12}} \xrightarrow{k_{E}^{A}} \mathrm{D} + \mathrm{D^R_{12}}$	$a_7 = \frac{k_E^A}{\Omega} n^A n_{12}^R$	$\frac{k_E^A}{\Omega}$	0.2	0.2	1	1, 1.57, 9.4, 94	10	10, 30, 180, 1800
8	$\mathrm{D^A} + \mathrm{D^R_2} \xrightarrow{k_{E}^{A}} \mathrm{D} + \mathrm{D^R_2}$	$a_8 = \frac{k_E^A}{\Omega} n^A n_2^R$	$\frac{k_E^A}{\Omega}$	0.2	0.2	1	1, 1.57, 9.4, 94	10	10, 30, 180, 1800
9	$\mathrm{D^A} + \mathrm{D^R_{12}} \xrightarrow{k_{E}^{A}} \mathrm{D} + \mathrm{D^R_{12}}$	$a_9 = \frac{k_E^A}{\Omega} n^A n_{12}^R$	$\frac{k_E^A}{\Omega}$	0.2	0.2	1	1, 1.57, 9.4, 94	10	10, 30, 180, 1800
10	$\mathrm{D} \xrightarrow{k_{W0}^{1}} \mathrm{D^R_1}$	$a_{10} = k_{W0}^{1} n^D$	k_{W0}^{1}	3.5	3.5	3.5	3.5	3.5	3.5
11	$\mathrm{D} \xrightarrow{k_{W}^{1}} \mathrm{D^R_1}$	$a_{11} = k_{W}^{1} n^D$	k_{W}^{1}	0	0	0	0	0	0
12	$\mathrm{D^R_1} \xrightarrow{k_T^{'}} \mathrm{D}$	$a_{12} = k_T^{'} n_1^R$	$k_T^{'}$	4, 3, 0.5, 0.05	4	4.7, 3, 0.5, 0.05	4.7	9, 3, 0.5, 0.05	9
13	$\mathrm{D^R_1} \xrightarrow{\delta^{'}} \mathrm{D}$	$a_{13} = \delta^{'} n_1^R$	$\delta^{'}$	4, 3, 0.5, 0.05	4	4.7, 3, 0.5, 0.05	4.7	9, 3, 0.5, 0.05	9
14	$\mathrm{D} + \mathrm{D^R_2} \xrightarrow{k_M^{'}} \mathrm{D^R_1} + \mathrm{D^R_2}$	$a_{14} = \frac{k_M^{'}}{\Omega} n^D n_2^R$	$\frac{k_M^{'}}{\Omega}$	0.2	0.2, 0.266, 1.6, 16	0.2	0.2, 0.314, 1.88, 18.8	0.2	0.2, 0.6, 3.6, 36
15	$\mathrm{D} + \mathrm{D^R_{12}} \xrightarrow{k_M^{'}} \mathrm{D^R_1} + \mathrm{D^R_{12}}$	$a_{15} = \frac{k_M^{'}}{\Omega} n^D n_{12}^R$	$\frac{k_M^{'}}{\Omega}$	0.2	0.2, 0.266, 1.6, 16	0.2	0.2, 0.314, 1.88, 18.8	0.2	0.2, 0.6, 3.6, 36
16	$\mathrm{D^R_1} + \mathrm{D^A} \xrightarrow{k_T^{'*}} \mathrm{D} + \mathrm{D^A}$	$a_{16} = \frac{k_T^{'*}}{\Omega} n_1^R n^A$	$\frac{k_T^{'*}}{\Omega}$	0.2	0.2	1	1, 1.57, 9.4, 94	10	10, 30, 180, 1800
17	$\mathrm{D} \xrightarrow{k_{W0}^{2}} \mathrm{D^R_2}$	$a_{17} = k_{W0}^{2} n^D$	k_{W0}^{2}	3.5	3.5	3.5	3.5	3.5	3.5
18	$\mathrm{D} \xrightarrow{k_{W}^{2}} \mathrm{D^R_2}$	$a_{18} = k_{W}^{2} n^D$	k_{W}^{2}	0	0	0	0	0	0
19	$\mathrm{D^R_2} \xrightarrow{\bar{k}_{E}^{R}} \mathrm{D}$	$a_{19} = \bar{k}_{E}^{R} n_2^R$	$\bar{k}_{E}^{R}$	4, 3, 0.5, 0.05	4	4.7, 3, 0.5, 0.05	4.7	9, 3, 0.5, 0.05	9
20	$\mathrm{D^R_2} \xrightarrow{\delta} \mathrm{D}$	$a_{20} = \delta n_2^R$	δ	4, 3, 0.5, 0.05	4	4.7, 3, 0.5, 0.05	4.7	9, 3, 0.5, 0.05	9
21	$\mathrm{D} + \mathrm{D^R_2} \xrightarrow{k_M} \mathrm{D^R_2} + \mathrm{D^R_2}$	$a_{21} = \frac{k_M}{\Omega} n^D n_2^R$	$\frac{k_M}{\Omega}$	0.2	0.2, 0.266, 1.6, 16	0.2	0.2, 0.314, 1.88, 18.8	0.2	0.2, 0.6, 3.6, 36
22	$\mathrm{D} + \mathrm{D^R_{12}} \xrightarrow{k_M} \mathrm{D^R_2} + \mathrm{D^R_{12}}$	$a_{22} = \frac{k_M}{\Omega} n^D n_{12}^R$	$\frac{k_M}{\Omega}$	0.2	0.2, 0.266, 1.6, 16	0.2	0.2, 0.314, 1.88, 18.8	0.2	0.2, 0.6, 3.6, 36
23	$\mathrm{D} + \mathrm{D^R_1} \xrightarrow{\bar{k}_M} \mathrm{D^R_2} + \mathrm{D^R_1}$	$a_{23} = \frac{\bar{k}_M}{\Omega} n^D n_1^R$	$\frac{\bar{k}_M}{\Omega}$	0.2	0.2, 0.266, 1.6, 16	0.2	0.2, 0.314, 1.88, 18.8	0.2	0.2, 0.6, 3.6, 36
24	$\mathrm{D} + \mathrm{D^R_{12}} \xrightarrow{\bar{k}_M} \mathrm{D^R_2} + \mathrm{D^R_{12}}$	$a_{24} = \frac{\bar{k}_M}{\Omega} n^D n_{12}^R$	$\frac{\bar{k}_M}{\Omega}$	0.2	0.2, 0.266, 1.6, 16	0.2	0.2, 0.314, 1.88, 18.8	0.2	0.2, 0.6, 3.6, 36
25	$\mathrm{D^R_2} + \mathrm{D^A} \xrightarrow{k_{E}^{R}} \mathrm{D} + \mathrm{D^A}$	$a_{25} = \frac{k_E^R}{\Omega} n_2^R n^A$	$\frac{k_E^R}{\Omega}$	0.2	0.2	1	1, 1.57, 9.4, 94	10	10, 30, 180, 1800
26	$\mathrm{D^R_1} \xrightarrow{k_{W0}^{2}} \mathrm{D^R_{12}}$	$a_{26} = k_{W0}^{2} n_1^R$	k_{W0}^{2}	3.5	3.5	3.5	3.5	3.5	3.5
27	$\mathrm{D^R_{12}} \xrightarrow{\bar{k}_{E}^{R}} \mathrm{D^R_1}$	$a_{27} = \bar{k}_{E}^{R} n_{12}^R$	$\bar{k}_{E}^{R}$	4, 3, 0.5, 0.05	4	4.7, 3, 0.5, 0.05	4.7	9, 3, 0.5, 0.05	9
28	$\mathrm{D^R_{12}} \xrightarrow{\delta} \mathrm{D^R_1}$	$a_{28} = \delta n_{12}^R$	δ	4, 3, 0.5, 0.05	4	4.7, 3, 0.5, 0.05	4.7	9, 3, 0.5, 0.05	9
29	$\mathrm{D^R_1} + \mathrm{D^R_2} \xrightarrow{k_M} \mathrm{D^R_{12}} + \mathrm{D^R_2}$	$a_{29} = \frac{k_M}{\Omega} n_1^R n_2^R$	$\frac{k_M}{\Omega}$	0.2	0.2, 0.266, 1.6, 16	0.2	0.2, 0.314, 1.88, 18.8	0.2	0.2, 0.6, 3.6, 36
30	$\mathrm{D^R_1} + \mathrm{D^R_{12}} \xrightarrow{k_M} \mathrm{D^R_{12}} + \mathrm{D^R_{12}}$	$a_{30} = \frac{k_M}{\Omega} n_1^R n_{12}^R$	$\frac{k_M}{\Omega}$	0.2	0.2, 0.266, 1.6, 16	0.2	0.2, 0.314, 1.88, 18.8	0.2	0.2, 0.6, 3.6, 36
31	$\mathrm{D^R_1} + \mathrm{D^R_1} \xrightarrow{\bar{k}_M} \mathrm{D^R_{12}} + \mathrm{D^R_1}$	$a_{31} = \frac{\bar{k}_M}{\Omega} \frac{n_1^R(n_1^R-1)}{2}$	$\frac{\bar{k}_M}{\Omega}$	0.2	0.2, 0.266, 1.6, 16	0.2	0.2, 0.314, 1.88, 18.8	0.2	0.2, 0.6, 3.6, 36
32	$\mathrm{D^R_1} + \mathrm{D^R_{12}} \xrightarrow{\bar{k}_M} \mathrm{D^R_{12}} + \mathrm{D^R_{12}}$	$a_{32} = \frac{\bar{k}_M}{\Omega} n_1^R n_{12}^R$	$\frac{\bar{k}_M}{\Omega}$	0.2	0.2, 0.266, 1.6, 16	0.2	0.2, 0.314, 1.88, 18.8	0.2	0.2, 0.6, 3.6, 36
33	$\mathrm{D^R_{12}} + \mathrm{D^A} \xrightarrow{k_{E}^{R}} \mathrm{D^R_1} + \mathrm{D^A}$	$a_{33} = \frac{k_E^R}{\Omega} n_{12}^R n^A$	$\frac{k_E^R}{\Omega}$	0.2	0.2	1	1, 1.57, 9.4, 94	10	10, 30, 180, 1800
34	$\mathrm{D^R_2} \xrightarrow{k_{W0}^{1}} \mathrm{D^R_{12}}$	$a_{34} = k_{W0}^{1} n_2^R$	k_{W0}^{1}	3.5	3.5	3.5	3.5	3.5	3.5
35	$\mathrm{D^R_{12}} \xrightarrow{k_T^{'}} \mathrm{D^R_2}$	$a_{35} = k_T^{'} n_{12}^R$	$k_T^{'}$	4, 3, 0.5, 0.05	4	4.7, 3, 0.5, 0.05	4.7	9, 3, 0.5, 0.05	9
36	$\mathrm{D^R_{12}} \xrightarrow{\delta^{'}} \mathrm{D^R_2}$	$a_{36} = \delta^{'} n_{12}^R$	$\delta^{'}$	4, 3, 0.5, 0.05	4	4.7, 3, 0.5, 0.05	4.7	9, 3, 0.5, 0.05	9
37	$\mathrm{D^R_2} + \mathrm{D^R_2} \xrightarrow{k_M^{'}} \mathrm{D^R_{12}} + \mathrm{D^R_2}$	$a_{37} = \frac{k_M^{'}}{\Omega} \frac{n_2^R(n_2^R-1)}{2}$	$\frac{k_M^{'}}{\Omega}$	0.2	0.2, 0.266, 1.6, 16	0.2	0.2, 0.314, 1.88, 18.8	0.2	0.2, 0.6, 3.6, 36
38	$\mathrm{D^R_2} + \mathrm{D^R_{12}} \xrightarrow{k_M^{'}} \mathrm{D^R_{12}} + \mathrm{D^R_{12}}$	$a_{38} = \frac{k_M^{'}}{\Omega} n_2^R n_{12}^R$	$\frac{k_M^{'}}{\Omega}$	0.2	0.2, 0.266, 1.6, 16	0.2	0.2, 0.314, 1.88, 18.8	0.2	0.2, 0.6, 3.6, 36
39	$\mathrm{D^R_{12}} + \mathrm{D^A} \xrightarrow{k_T^{'*}} \mathrm{D^R_2} + \mathrm{D^A}$	$a_{39} = \frac{k_T^{'*}}{\Omega} n_{12}^R n^A$	$\frac{k_T^{'*}}{\Omega}$	0.2	0.2	1	1, 1.57, 9.4, 94	10	10, 30, 180, 1800

Table L: **Reactions and parameter values used to generate the plots in Fig Q, going from the top to the bottom.**

$\mathbf{R}_j$	**Reaction**	**Prop.Func.**(a_j)	**Param.**	**Value** $(\mathbf{h}^{-1})$ Fig RA	**Value** $(\mathbf{h}^{-1})$ Fig RB
1	$\mathrm{D} \xrightarrow{k^A_{W0}} \mathrm{D^A}$	$a_1 = k^A_{W0} n^D$	k^A_{W0}	3.5	3.5
2	$\mathrm{D} \xrightarrow{k^A_{W}} \mathrm{D^A}$	$a_2 = k^A_{W} n^D$	k^A_{W}	0<br>0	0, 3.5, 50 (upper plots)<br>0 (lower plots)
3	$\mathrm{D^A} \xrightarrow{\bar{k}^A_{E}} \mathrm{D}$	$a_3 = \bar{k}^A_{E} n^A$	$\bar{k}^A_{E}$	4.7, 3, 0.5	3
4	$\mathrm{D^A} \xrightarrow{\delta} \mathrm{D}$	$a_4 = \delta n^A$	δ	4.7, 3, 0.5	3
5	$\mathrm{D} + \mathrm{D^A} \xrightarrow{k^A_{M}} \mathrm{D^A} + \mathrm{D^A}$	$a_5 = \frac{k^A_M}{\Omega} n^D n^A$	$\frac{k^A_M}{\Omega}$	1	1
6	$\mathrm{D^A} + \mathrm{D^R_1} \xrightarrow{k^A_{E}} \mathrm{D} + \mathrm{D^R_1}$	$a_6 = \frac{k^A_E}{\Omega} n^A n^R_1$	$\frac{k^A_E}{\Omega}$	1	1
7	$\mathrm{D^A} + \mathrm{D^R_{12}} \xrightarrow{k^A_{E}} \mathrm{D} + \mathrm{D^R_{12}}$	$a_7 = \frac{k^A_E}{\Omega} n^A n^R_{12}$	$\frac{k^A_E}{\Omega}$	1	1
8	$\mathrm{D^A} + \mathrm{D^R_2} \xrightarrow{k^A_{E}} \mathrm{D} + \mathrm{D^R_2}$	$a_8 = \frac{k^A_E}{\Omega} n^A n^R_2$	$\frac{k^A_E}{\Omega}$	1	1
9	$\mathrm{D^A} + \mathrm{D^R_{12}} \xrightarrow{k^A_{E}} \mathrm{D} + \mathrm{D^R_{12}}$	$a_9 = \frac{k^A_E}{\Omega} n^A n^R_{12}$	$\frac{k^A_E}{\Omega}$	1	1
10	$\mathrm{D} \xrightarrow{k^1_{W0}} \mathrm{D^R_1}$	$a_{10} = k^1_{W0} n^D$	k^1_{W0}	3.5	3.5
11	$\mathrm{D} \xrightarrow{k^1_{W}} \mathrm{D^R_1}$	$a_{11} = k^1_{W} n^D$	k^1_{W}	0<br>0	0 (upper plots)<br>0, 2.5, 50 (lower plots)
12	$\mathrm{D^R_1} \xrightarrow{k'_T} \mathrm{D}$	$a_{12} = k'_T n^R_1$	k'_T	47, 5.64, 4.7, 3.76, 0.47 (left plots)<br>30, 3.6, 3, 2.4, 0.3 (central plots)<br>5, 0.6, 0.5, 0.4, 0.05 (right plots)	3, 2.4
13	$\mathrm{D^R_1} \xrightarrow{\delta'} \mathrm{D}$	$a_{13} = \delta' n^R_1$	δ'	47, 5.64, 4.7, 3.76, 0.47 (left plots)<br>30, 3.6, 3, 2.4, 0.3 (central plots)<br>5, 0.6, 0.5, 0.4, 0.05 (right plots)	3, 2.4
14	$\mathrm{D} + \mathrm{D^R_2} \xrightarrow{k'_M} \mathrm{D^R_1} + \mathrm{D^R_2}$	$a_{14} = \frac{k'_M}{\Omega} n^D n^R_2$	$\frac{k'_M}{\Omega}$	0.2	0.2
15	$\mathrm{D} + \mathrm{D^R_{12}} \xrightarrow{k'_M} \mathrm{D^R_1} + \mathrm{D^R_{12}}$	$a_{15} = \frac{k'_M}{\Omega} n^D n^R_{12}$	$\frac{k'_M}{\Omega}$	0.2	0.2
16	$\mathrm{D^R_1} + \mathrm{D^A} \xrightarrow{k'^*_T} \mathrm{D} + \mathrm{D^A}$	$a_{16} = \frac{k'^*_T}{\Omega} n^R_1 n^A$	$\frac{k'^*_T}{\Omega}$	10,1.2,1,0.8,0.1	1, 0.8
17	$\mathrm{D} \xrightarrow{k^2_{W0}} \mathrm{D^R_2}$	$a_{17} = k^2_{W0} n^D$	k^2_{W0}	3.5	3.5
18	$\mathrm{D} \xrightarrow{k^2_{W}} \mathrm{D^R_2}$	$a_{18} = k^2_{W} n^D$	k^2_{W}	0<br>0	0 (upper plots)<br>0, 2.5, 50 (lower plots)
19	$\mathrm{D^R_2} \xrightarrow{\bar{k}^R_E} \mathrm{D}$	$a_{19} = \bar{k}^R_E n^R_2$	$\bar{k}^R_E$	4.7, 3, 0.5	3
20	$\mathrm{D^R_2} \xrightarrow{\delta} \mathrm{D}$	$a_{20} = \delta n^R_2$	δ	4.7, 3, 0.5	3
21	$\mathrm{D} + \mathrm{D^R_2} \xrightarrow{k_M} \mathrm{D^R_2} + \mathrm{D^R_2}$	$a_{21} = \frac{k_M}{\Omega} n^D n^R_2$	$\frac{k_M}{\Omega}$	0.2	0.2
22	$\mathrm{D} + \mathrm{D^R_{12}} \xrightarrow{k_M} \mathrm{D^R_2} + \mathrm{D^R_{12}}$	$a_{22} = \frac{k_M}{\Omega} n^D n^R_{12}$	$\frac{k_M}{\Omega}$	0.2	0.2
23	$\mathrm{D} + \mathrm{D^R_1} \xrightarrow{\bar{k}_M} \mathrm{D^R_2} + \mathrm{D^R_1}$	$a_{23} = \frac{\bar{k}_M}{\Omega} n^D n^R_1$	$\frac{\bar{k}_M}{\Omega}$	0.2	0.2
24	$\mathrm{D} + \mathrm{D^R_{12}} \xrightarrow{\bar{k}_M} \mathrm{D^R_2} + \mathrm{D^R_{12}}$	$a_{24} = \frac{\bar{k}_M}{\Omega} n^D n^R_{12}$	$\frac{\bar{k}_M}{\Omega}$	0.2	0.2
25	$\mathrm{D^R_2} + \mathrm{D^A} \xrightarrow{k^R_E} \mathrm{D} + \mathrm{D^A}$	$a_{25} = \frac{k^R_E}{\Omega} n^R_2 n^A$	$\frac{k^R_E}{\Omega}$	1	1
26	$\mathrm{D^R_1} \xrightarrow{k^2_{W0}} \mathrm{D^R_{12}}$	$a_{26} = k^2_{W0} n^R_1$	k^2_{W0}	3.5	3.5
27	$\mathrm{D^R_{12}} \xrightarrow{\bar{k}^R_E} \mathrm{D^R_1}$	$a_{27} = \bar{k}^R_E n^R_{12}$	$\bar{k}^R_E$	4.7, 3, 0.5	3
28	$\mathrm{D^R_{12}} \xrightarrow{\delta} \mathrm{D^R_1}$	$a_{28} = \delta n^R_{12}$	δ	4.7, 3, 0.5	3
29	$\mathrm{D^R_1} + \mathrm{D^R_2} \xrightarrow{k_M} \mathrm{D^R_{12}} + \mathrm{D^R_2}$	$a_{29} = \frac{k_M}{\Omega} n^R_1 n^R_2$	$\frac{k_M}{\Omega}$	0.2	0.2
30	$\mathrm{D^R_1} + \mathrm{D^R_{12}} \xrightarrow{k_M} \mathrm{D^R_{12}} + \mathrm{D^R_{12}}$	$a_{30} = \frac{k_M}{\Omega} n^R_1 n^R_{12}$	$\frac{k_M}{\Omega}$	0.2	0.2
31	$\mathrm{D^R_1} + \mathrm{D^R_1} \xrightarrow{\bar{k}_M} \mathrm{D^R_{12}} + \mathrm{D^R_1}$	$a_{31} = \frac{\bar{k}_M}{\Omega} \frac{n^R_1(n^R_1-1)}{2}$	$\frac{\bar{k}_M}{\Omega}$	0.2	0.2
32	$\mathrm{D^R_1} + \mathrm{D^R_{12}} \xrightarrow{\bar{k}_M} \mathrm{D^R_{12}} + \mathrm{D^R_{12}}$	$a_{32} = \frac{\bar{k}_M}{\Omega} n^R_1 n^R_{12}$	$\frac{\bar{k}_M}{\Omega}$	0.2	0.2
33	$\mathrm{D^R_{12}} + \mathrm{D^A} \xrightarrow{k^R_E} \mathrm{D^R_1} + \mathrm{D^A}$	$a_{33} = \frac{k^R_E}{\Omega} n^R_{12} n^A$	$\frac{k^R_E}{\Omega}$	1	1
34	$\mathrm{D^R_2} \xrightarrow{k^1_{W0}} \mathrm{D^R_{12}}$	$a_{34} = k^1_{W0} n^R_2$	k^1_{W0}	3.5	3.5
35	$\mathrm{D^R_{12}} \xrightarrow{k'_T} \mathrm{D^R_2}$	$a_{35} = k'_T n^R_{12}$	k'_T	47, 5.64, 4.7, 3.76, 0.47 (left plots)<br>30, 3.6, 3, 2.4, 0.3 (central plots)<br>5, 0.6, 0.5, 0.4, 0.05 (right plots)	3, 2.4
36	$\mathrm{D^R_{12}} \xrightarrow{\delta'} \mathrm{D^R_2}$	$a_{36} = \delta' n^R_{12}$	δ'	47, 5.64, 4.7, 3.76, 0.47 (left plots)<br>30, 3.6, 3, 2.4, 0.3 (central plots)<br>5, 0.6, 0.5, 0.4, 0.05 (right plots)	3, 2.4
37	$\mathrm{D^R_2} + \mathrm{D^R_2} \xrightarrow{k'_M} \mathrm{D^R_{12}} + \mathrm{D^R_2}$	$a_{37} = \frac{k'_M}{\Omega} \frac{n^R_2(n^R_2-1)}{2}$	$\frac{k'_M}{\Omega}$	0.2	0.2
38	$\mathrm{D^R_2} + \mathrm{D^R_{12}} \xrightarrow{k'_M} \mathrm{D^R_{12}} + \mathrm{D^R_{12}}$	$a_{38} = \frac{k'_M}{\Omega} n^R_2 n^R_{12}$	$\frac{k'_M}{\Omega}$	0.2	0.2
39	$\mathrm{D^R_{12}} + \mathrm{D^A} \xrightarrow{k'^*_T} \mathrm{D^R_2} + \mathrm{D^A}$	$a_{39} = \frac{k'^*_T}{\Omega} n^R_{12} n^A$	$\frac{k'^*_T}{\Omega}$	10,1.2,1,0.8,0.1	1, 0.8

Table M: **Reactions and parameter values used to generate the plots in Fig R.**

R_j	Reaction	Prop.Func.(a_j)	Param.	Value (h^{-1})<br>upper side<br>left plots	Value (h^{-1})<br>upper side<br>central plots	Value (h^{-1})<br>upper side<br>right plots	Value (h^{-1})<br>lower side<br>left plots	Value (h^{-1})<br>lower side<br>central plots	Value (h^{-1})<br>lower side<br>right plots
1	$\mathrm{D} \xrightarrow{k_{W0}^A} \mathrm{D^A}$	$a_1 = k_{W0}^A n^D$	k_{W0}^A	3.5	3.5	3.5	3.5	3.5	3.5
2	$\mathrm{D} \xrightarrow{k_W^A} \mathrm{D^A}$	$a_2 = k_W^A n^D$	k_W^A	0	0	0	0	0	0
3	$\mathrm{D^A} \xrightarrow{\bar{k}_E^A} \mathrm{D}$	$a_3 = \bar{k}_E^A n^A$	$\bar{k}_E^A$	3, 0.5	3, 0.5	3, 0.5	3, 0.5	3, 0.5	3, 0.5
4	$\mathrm{D^A} \xrightarrow{\delta} \mathrm{D}$	$a_4 = \delta n^A$	δ	3, 0.5	3, 0.5	3, 0.5	3, 0.5	3, 0.5	3, 0.5
5	$\mathrm{D} + \mathrm{D^A} \xrightarrow{k_M^A} \mathrm{D^A} + \mathrm{D^A}$	$a_5 = \frac{k_M^A}{\Omega} n^D n^A$	$\frac{k_M^A}{\Omega}$	1	1	1	1	1	1
6	$\mathrm{D^A} + \mathrm{D_1^R} \xrightarrow{k_E^A} \mathrm{D} + \mathrm{D_1^R}$	$a_6 = \frac{k_E^A}{\Omega} n^A n_1^R$	$\frac{k_E^A}{\Omega}$	0.2	1	10	0.2	1	10
7	$\mathrm{D^A} + \mathrm{D_{12}^R} \xrightarrow{k_E^A} \mathrm{D} + \mathrm{D_{12}^R}$	$a_7 = \frac{k_E^A}{\Omega} n^A n_{12}^R$	$\frac{k_E^A}{\Omega}$	0.2	1	10	0.2	1	10
8	$\mathrm{D^A} + \mathrm{D_2^R} \xrightarrow{k_E^A} \mathrm{D} + \mathrm{D_2^R}$	$a_8 = \frac{k_E^A}{\Omega} n^A n_2^R$	$\frac{k_E^A}{\Omega}$	0.2	1	10	0.2	1	10
9	$\mathrm{D^A} + \mathrm{D_{12}^R} \xrightarrow{k_E^A} \mathrm{D} + \mathrm{D_{12}^R}$	$a_9 = \frac{k_E^A}{\Omega} n^A n_{12}^R$	$\frac{k_E^A}{\Omega}$	0.2	1	10	0.2	1	10
10	$\mathrm{D} \xrightarrow{k_{W0}^1} \mathrm{D_1^R}$	$a_{10} = k_{W0}^1 n^D$	3.5	3.5	3.5	3.5	3.5	3.5	3.5
11	$\mathrm{D} \xrightarrow{k_W^1} \mathrm{D_1^R}$	$a_{11} = k_W^1 n^D$	k_W^1	0	0	0	0	0	0
12	$\mathrm{D_1^R} \xrightarrow{k_T^{'}} \mathrm{D}$	$a_{12} = k_T^{'} n_1^R$	$k_T^{'}$	30, 3, 0.3<br>5, 0.5, 0.05	30, 3, 0.3<br>5, 0.5, 0.05	30, 3, 0.3<br>5, 0.5, 0.05	30, 3, 0.3<br>5, 0.5, 0.05	30, 3, 0.3<br>5, 0.5, 0.05	30, 3, 0.3 (left side)<br>5, 0.5, 0.05 (right side)
13	$\mathrm{D_1^R} \xrightarrow{\delta^{'}} \mathrm{D}$	$a_{13} = \delta^{'} n_1^R$	$\delta^{'}$	30, 3, 0.3<br>5, 0.5, 0.05	30, 3, 0.3<br>5, 0.5, 0.05	30, 3, 0.3<br>5, 0.5, 0.05	30, 3, 0.3<br>5, 0.5, 0.05	30, 3, 0.3<br>5, 0.5, 0.05	30, 3, 0.3 (left side)<br>5, 0.5, 0.05 (right side)
14	$\mathrm{D} + \mathrm{D_2^R} \xrightarrow{k_M^{'}} \mathrm{D_1^R} + \mathrm{D_2^R}$	$a_{14} = \frac{k_M^{'}}{\Omega} n^D n_2^R$	$\frac{k_M^{'}}{\Omega}$	0.2	0.2	0.2	0.2	0.2	0.2
15	$\mathrm{D} + \mathrm{D_{12}^R} \xrightarrow{k_M^{'}} \mathrm{D_1^R} + \mathrm{D_{12}^R}$	$a_{15} = \frac{k_M^{'}}{\Omega} n^D n_{12}^R$	$\frac{k_M^{'}}{\Omega}$	0.2	0.2	0.2	0.2	0.2	0.2
16	$\mathrm{D_1^R} + \mathrm{D^A} \xrightarrow{k_T^{'*}} \mathrm{D} + \mathrm{D^A}$	$a_{16} = \frac{k_T^{'*}}{\Omega} n_1^R n^A$	$\frac{k_T^{'*}}{\Omega}$	2,0.2,0.02	10,1,0.1	100,10,1	2,0.2,0.02	10,1,0.1	100,10,1
17	$\mathrm{D} \xrightarrow{k_{W0}^2} \mathrm{D_2^R}$	$a_{17} = k_{W0}^2 n^D$	k_{W0}^2	3.5	3.5	3.5	3.5	3.5	3.5
18	$\mathrm{D} \xrightarrow{k_W^2} \mathrm{D_2^R}$	$a_{18} = k_W^2 n^D$	k_W^2	0	0	0	0	0	0
19	$\mathrm{D_2^R} \xrightarrow{\bar{k}_E^R} \mathrm{D}$	$a_{19} = \bar{k}_E^R n_2^R$	$\bar{k}_E^R$	3, 0.5	3, 0.5	3, 0.5	30, 5	30, 5	30, 5
20	$\mathrm{D_2^R} \xrightarrow{\delta} \mathrm{D}$	$a_{20} = \delta n_2^R$	δ	3, 0.5	3, 0.5	3, 0.5	3, 0.5	3, 0.5	3, 0.5
21	$\mathrm{D} + \mathrm{D_2^R} \xrightarrow{k_M} \mathrm{D_2^R} + \mathrm{D_2^R}$	$a_{21} = \frac{k_M}{\Omega} n^D n_2^R$	$\frac{k_M}{\Omega}$	0.2	0.2	0.2	0.2	0.2	0.2
22	$\mathrm{D} + \mathrm{D_{12}^R} \xrightarrow{k_M} \mathrm{D_2^R} + \mathrm{D_{12}^R}$	$a_{22} = \frac{k_M}{\Omega} n^D n_{12}^R$	$\frac{k_M}{\Omega}$	0.2	0.2	0.2	0.2	0.2	0.2
23	$\mathrm{D} + \mathrm{D_1^R} \xrightarrow{\bar{k}_M} \mathrm{D_2^R} + \mathrm{D_1^R}$	$a_{23} = \frac{\bar{k}_M}{\Omega} n^D n_1^R$	$\frac{\bar{k}_M}{\Omega}$	0.2	0.2	0.2	0.2	0.2	0.2
24	$\mathrm{D} + \mathrm{D_{12}^R} \xrightarrow{\bar{k}_M} \mathrm{D_2^R} + \mathrm{D_{12}^R}$	$a_{24} = \frac{\bar{k}_M}{\Omega} n^D n_{12}^R$	$\frac{\bar{k}_M}{\Omega}$	0.2	0.2	0.2	0.2	0.2	0.2
25	$\mathrm{D_2^R} + \mathrm{D^A} \xrightarrow{k_E^R} \mathrm{D} + \mathrm{D^A}$	$a_{25} = \frac{k_E^R}{\Omega} n_2^R n^A$	$\frac{k_E^R}{\Omega}$	0.2	1	10	2	10	100
26	$\mathrm{D_1^R} \xrightarrow{k_{W0}^2} \mathrm{D_{12}^R}$	$a_{26} = k_{W0}^2 n_1^R$	k_{W0}^2	3.5	3.5	3.5	3.5	3.5	3.5
27	$\mathrm{D_{12}^R} \xrightarrow{\bar{k}_E^R} \mathrm{D_1^R}$	$a_{27} = \bar{k}_E^R n_{12}^R$	$\bar{k}_E^R$	3, 0.5	3, 0.5	3, 0.5	30, 5	30, 5	30, 5
28	$\mathrm{D_{12}^R} \xrightarrow{\delta} \mathrm{D_1^R}$	$a_{28} = \delta n_{12}^R$	δ	3, 0.5	3, 0.5	3, 0.5	3, 0.5	3, 0.5	3, 0.5
29	$\mathrm{D_1^R} + \mathrm{D_2^R} \xrightarrow{k_M} \mathrm{D_{12}^R} + \mathrm{D_2^R}$	$a_{29} = \frac{k_M}{\Omega} n_1^R n_2^R$	$\frac{k_M}{\Omega}$	0.2	0.2	0.2	0.2	0.2	0.2
30	$\mathrm{D_1^R} + \mathrm{D_{12}^R} \xrightarrow{k_M} \mathrm{D_{12}^R} + \mathrm{D_{12}^R}$	$a_{30} = \frac{k_M}{\Omega} n_1^R n_{12}^R$	$\frac{k_M}{\Omega}$	0.2	0.2	0.2	0.2	0.2	0.2
31	$\mathrm{D_1^R} + \mathrm{D_1^R} \xrightarrow{\bar{k}_M} \mathrm{D_{12}^R} + \mathrm{D_1^R}$	$a_{31} = \frac{\bar{k}_M}{\Omega} \frac{n_1^R(n_1^R-1)}{2}$	$\frac{\bar{k}_M}{\Omega}$	0.2	0.2	0.2	0.2	0.2	0.2
32	$\mathrm{D_1^R} + \mathrm{D_{12}^R} \xrightarrow{\bar{k}_M} \mathrm{D_{12}^R} + \mathrm{D_{12}^R}$	$a_{32} = \frac{\bar{k}_M}{\Omega} n_1^R n_{12}^R$	$\frac{\bar{k}_M}{\Omega}$	0.2	0.2	0.2	0.2	0.2	0.2
33	$\mathrm{D_{12}^R} + \mathrm{D^A} \xrightarrow{k_E^R} \mathrm{D_1^R} + \mathrm{D^A}$	$a_{33} = \frac{k_E^R}{\Omega} n_{12}^R n^A$	$\frac{k_E^R}{\Omega}$	0.2	1	10	2	10	100
34	$\mathrm{D_2^R} \xrightarrow{k_{W0}^1} \mathrm{D_{12}^R}$	$a_{34} = k_{W0}^1 n_2^R$	k_{W0}^1	3.5	3.5	3.5	3.5	3.5	3.5
35	$\mathrm{D_{12}^R} \xrightarrow{k_T^{'}} \mathrm{D_2^R}$	$a_{35} = k_T^{'} n_{12}^R$	$k_T^{'}$	30, 3, 0.3<br>5, 0.5, 0.05	30, 3, 0.3<br>5, 0.5, 0.05	30, 3, 0.3<br>5, 0.5, 0.05	30, 3, 0.3<br>5, 0.5, 0.05	30, 3, 0.3<br>5, 0.5, 0.05	30, 3, 0.3 (left side)<br>5, 0.5, 0.05 (right side)
36	$\mathrm{D_{12}^R} \xrightarrow{\delta^{'}} \mathrm{D_2^R}$	$a_{36} = \delta^{'} n_{12}^R$	$\delta^{'}$	30, 3, 0.3<br>5, 0.5, 0.05	30, 3, 0.3<br>5, 0.5, 0.05	30, 3, 0.3<br>5, 0.5, 0.05	30, 3, 0.3<br>5, 0.5, 0.05	30, 3, 0.3<br>5, 0.5, 0.05	30, 3, 0.3 (left side)<br>5, 0.5, 0.05 (right side)
37	$\mathrm{D_2^R} + \mathrm{D_2^R} \xrightarrow{k_M^{'}} \mathrm{D_{12}^R} + \mathrm{D_2^R}$	$a_{37} = \frac{k_M^{'}}{\Omega} \frac{n_2^R(n_2^R-1)}{2}$	$\frac{k_M^{'}}{\Omega}$	0.2	0.2	0.2	0.2	0.2	0.2
38	$\mathrm{D_2^R} + \mathrm{D_{12}^R} \xrightarrow{k_M^{'}} \mathrm{D_{12}^R} + \mathrm{D_{12}^R}$	$a_{38} = \frac{k_M^{'}}{\Omega} n_2^R n_{12}^R$	$\frac{k_M^{'}}{\Omega}$	0.2	0.2	0.2	0.2	0.2	0.2
39	$\mathrm{D_{12}^R} + \mathrm{D^A} \xrightarrow{k_T^{'*}} \mathrm{D_2^R} + \mathrm{D^A}$	$a_{39} = \frac{k_T^{'*}}{\Omega} n_{12}^R n^A$	$\frac{k_T^{'*}}{\Omega}$	2,0.2,0.02	10,1,0.1	100,10,1	2,0.2,0.02	10,1,0.1	100,10,1

Table N: **Reactions and parameter values used to generate the plots in Fig S.**

$\mathbf{R}_j$	**Reaction**	**Prop.Func.(a_j)**	**Param.**	**Value ($\mathbf{h}^{-1}$)** left plots	**Value ($\mathbf{h}^{-1}$)** right plots
1	$\mathrm{D} \xrightarrow{k_{W0}^A} \mathrm{D}^\mathrm{A}$	$a_1 = k_{W0}^A n^D$	k_{W0}^A	3.5	3.5
2	$\mathrm{D} \xrightarrow{k_W^A} \mathrm{D}^\mathrm{A}$	$a_2 = k_W^A n^D$	k_W^A	0, 3.5, 50<br>0	0, 3.5, 50 (upper plots)<br>0 (lower plots)
3	$\mathrm{D}^\mathrm{A} \xrightarrow{\bar{k}_E^A} \mathrm{D}$	$a_3 = \bar{k}_E^A n^A$	$\bar{k}_E^A$	3	3
4	$\mathrm{D}^\mathrm{A} \xrightarrow{\delta} \mathrm{D}$	$a_4 = \delta n^A$	δ	3	3
5	$\mathrm{D} + \mathrm{D}^\mathrm{A} \xrightarrow{k_M^A} \mathrm{D}^\mathrm{A} + \mathrm{D}^\mathrm{A}$	$a_5 = \frac{k_M^A}{\Omega} n^D n^A$	$\frac{k_M^A}{\Omega}$	1	1
6	$\mathrm{D}^\mathrm{A} + \mathrm{D}_1^\mathrm{R} \xrightarrow{k_E^A} \mathrm{D} + \mathrm{D}_1^\mathrm{R}$	$a_6 = \frac{k_E^A}{\Omega} n^A n_1^R$	$\frac{k_E^A}{\Omega}$	0.2	10
7	$\mathrm{D}^\mathrm{A} + \mathrm{D}_{12}^\mathrm{R} \xrightarrow{k_E^A} \mathrm{D} + \mathrm{D}_{12}^\mathrm{R}$	$a_7 = \frac{k_E^A}{\Omega} n^A n_{12}^R$	$\frac{k_E^A}{\Omega}$	0.2	10
8	$\mathrm{D}^\mathrm{A} + \mathrm{D}_2^\mathrm{R} \xrightarrow{k_E^A} \mathrm{D} + \mathrm{D}_2^\mathrm{R}$	$a_8 = \frac{k_E^A}{\Omega} n^A n_2^R$	$\frac{k_E^A}{\Omega}$	0.2	10
9	$\mathrm{D}^\mathrm{A} + \mathrm{D}_{12}^\mathrm{R} \xrightarrow{k_E^A} \mathrm{D} + \mathrm{D}_{12}^\mathrm{R}$	$a_9 = \frac{k_E^A}{\Omega} n^A n_{12}^R$	$\frac{k_E^A}{\Omega}$	0.2	10
10	$\mathrm{D} \xrightarrow{k_{W0}^1} \mathrm{D}_1^\mathrm{R}$	$a_{10} = k_{W0}^1 n^D$	k_{W0}^1	3.5	3.5
11	$\mathrm{D} \xrightarrow{k_W^1} \mathrm{D}_1^\mathrm{R}$	$a_{11} = k_W^1 n^D$	k_W^1	0<br>0, 2.5, 50	0 (upper plots)<br>0, 2.5, 50 (lower plots)
12	$\mathrm{D}_1^\mathrm{R} \xrightarrow{k_T^{'}} \mathrm{D}$	$a_{12} = k_T^{'} n_1^R$	$k_T^{'}$	3, 2.4	3, 2.4
13	$\mathrm{D}_1^\mathrm{R} \xrightarrow{\delta^{'}} \mathrm{D}$	$a_{13} = \delta^{'} n_1^R$	$\delta^{'}$	3, 2.4	3, 2.4
14	$\mathrm{D} + \mathrm{D}_2^\mathrm{R} \xrightarrow{k_M^{'}} \mathrm{D}_1^\mathrm{R} + \mathrm{D}_2^\mathrm{R}$	$a_{14} = \frac{k_M^{'}}{\Omega} n^D n_2^R$	$\frac{k_M^{'}}{\Omega}$	0.2	0.2
15	$\mathrm{D} + \mathrm{D}_{12}^\mathrm{R} \xrightarrow{k_M^{'}} \mathrm{D}_1^\mathrm{R} + \mathrm{D}_{12}^\mathrm{R}$	$a_{15} = \frac{k_M^{'}}{\Omega} n^D n_{12}^R$	$\frac{k_M^{'}}{\Omega}$	0.2	0.2
16	$\mathrm{D}_1^\mathrm{R} + \mathrm{D}^\mathrm{A} \xrightarrow{k_T^{'*}} \mathrm{D} + \mathrm{D}^\mathrm{A}$	$a_{16} = \frac{k_T^{'*}}{\Omega} n_1^R n^A$	$\frac{k_T^{'*}}{\Omega}$	0.2, 0.16	10, 8
17	$\mathrm{D} \xrightarrow{k_{W0}^2} \mathrm{D}_2^\mathrm{R}$	$a_{17} = k_{W0}^2 n^D$	k_{W0}^2	3.5	3.5
18	$\mathrm{D} \xrightarrow{k_W^2} \mathrm{D}_2^\mathrm{R}$	$a_{18} = k_W^2 n^D$	k_W^2	0<br>0, 2.5, 50	0 (upper plots)<br>0, 2.5, 50 (lower plots)
19	$\mathrm{D}_2^\mathrm{R} \xrightarrow{\bar{k}_E^R} \mathrm{D}$	$a_{19} = \bar{k}_E^R n_2^R$	$\bar{k}_E^R$	3	3
20	$\mathrm{D}_2^\mathrm{R} \xrightarrow{\delta} \mathrm{D}$	$a_{20} = \delta n_2^R$	δ	3	3
21	$\mathrm{D} + \mathrm{D}_2^\mathrm{R} \xrightarrow{k_M} \mathrm{D}_2^\mathrm{R} + \mathrm{D}_2^\mathrm{R}$	$a_{21} = \frac{k_M}{\Omega} n^D n_2^R$	$\frac{k_M}{\Omega}$	0.2	0.2
22	$\mathrm{D} + \mathrm{D}_{12}^\mathrm{R} \xrightarrow{k_M} \mathrm{D}_2^\mathrm{R} + \mathrm{D}_{12}^\mathrm{R}$	$a_{22} = \frac{k_M}{\Omega} n^D n_{12}^R$	$\frac{k_M}{\Omega}$	0.2	0.2
23	$\mathrm{D} + \mathrm{D}_1^\mathrm{R} \xrightarrow{\bar{k}_M} \mathrm{D}_2^\mathrm{R} + \mathrm{D}_1^\mathrm{R}$	$a_{23} = \frac{\bar{k}_M}{\Omega} n^D n_1^R$	$\frac{\bar{k}_M}{\Omega}$	0.2	0.2
24	$\mathrm{D} + \mathrm{D}_{12}^\mathrm{R} \xrightarrow{\bar{k}_M} \mathrm{D}_2^\mathrm{R} + \mathrm{D}_{12}^\mathrm{R}$	$a_{24} = \frac{\bar{k}_M}{\Omega} n^D n_{12}^R$	$\frac{\bar{k}_M}{\Omega}$	0.2	0.2
25	$\mathrm{D}_2^\mathrm{R} + \mathrm{D}^\mathrm{A} \xrightarrow{k_E^R} \mathrm{D} + \mathrm{D}^\mathrm{A}$	$a_{25} = \frac{k_E^R}{\Omega} n_2^R n^A$	$\frac{k_E^R}{\Omega}$	0.2	10
26	$\mathrm{D}_1^\mathrm{R} \xrightarrow{k_{W0}^2} \mathrm{D}_{12}^\mathrm{R}$	$a_{26} = k_{W0}^2 n_1^R$	k_{W0}^2	3.5	3.5
27	$\mathrm{D}_{12}^\mathrm{R} \xrightarrow{\bar{k}_E^R} \mathrm{D}_1^\mathrm{R}$	$a_{27} = \bar{k}_E^R n_{12}^R$	$\bar{k}_E^R$	3	3
28	$\mathrm{D}_{12}^\mathrm{R} \xrightarrow{\delta} \mathrm{D}_1^\mathrm{R}$	$a_{28} = \delta n_{12}^R$	δ	3	3
29	$\mathrm{D}_1^\mathrm{R} + \mathrm{D}_2^\mathrm{R} \xrightarrow{k_M} \mathrm{D}_{12}^\mathrm{R} + \mathrm{D}_2^\mathrm{R}$	$a_{29} = \frac{k_M}{\Omega} n_1^R n_2^R$	$\frac{k_M}{\Omega}$	0.2	0.2
30	$\mathrm{D}_1^\mathrm{R} + \mathrm{D}_{12}^\mathrm{R} \xrightarrow{k_M} \mathrm{D}_{12}^\mathrm{R} + \mathrm{D}_{12}^\mathrm{R}$	$a_{30} = \frac{k_M}{\Omega} n_1^R n_{12}^R$	$\frac{k_M}{\Omega}$	0.2	0.2
31	$\mathrm{D}_1^\mathrm{R} + \mathrm{D}_1^\mathrm{R} \xrightarrow{\bar{k}_M} \mathrm{D}_{12}^\mathrm{R} + \mathrm{D}_1^\mathrm{R}$	$a_{31} = \frac{\bar{k}_M}{\Omega} \frac{n_1^R(n_1^R-1)}{2}$	$\frac{\bar{k}_M}{\Omega}$	0.2	0.2
32	$\mathrm{D}_1^\mathrm{R} + \mathrm{D}_{12}^\mathrm{R} \xrightarrow{\bar{k}_M} \mathrm{D}_{12}^\mathrm{R} + \mathrm{D}_{12}^\mathrm{R}$	$a_{32} = \frac{\bar{k}_M}{\Omega} n_1^R n_{12}^R$	$\frac{\bar{k}_M}{\Omega}$	0.2	0.2
33	$\mathrm{D}_{12}^\mathrm{R} + \mathrm{D}^\mathrm{A} \xrightarrow{k_E^R} \mathrm{D}_1^\mathrm{R} + \mathrm{D}^\mathrm{A}$	$a_{33} = \frac{k_E^R}{\Omega} n_{12}^R n^A$	$\frac{k_E^R}{\Omega}$	0.2	10
34	$\mathrm{D}_2^\mathrm{R} \xrightarrow{k_{W0}^1} \mathrm{D}_{12}^\mathrm{R}$	$a_{34} = k_{W0}^1 n_2^R$	k_{W0}^1	3.5	3.5
35	$\mathrm{D}_{12}^\mathrm{R} \xrightarrow{k_T^{'}} \mathrm{D}_2^\mathrm{R}$	$a_{35} = k_T^{'} n_{12}^R$	$k_T^{'}$	3, 2.4	3, 2.4
36	$\mathrm{D}_{12}^\mathrm{R} \xrightarrow{\delta^{'}} \mathrm{D}_2^\mathrm{R}$	$a_{36} = \delta^{'} n_{12}^R$	$\delta^{'}$	3, 2.4	3, 2.4
37	$\mathrm{D}_2^\mathrm{R} + \mathrm{D}_2^\mathrm{R} \xrightarrow{k_M^{'}} \mathrm{D}_{12}^\mathrm{R} + \mathrm{D}_2^\mathrm{R}$	$a_{37} = \frac{k_M^{'}}{\Omega} \frac{n_2^R(n_2^R-1)}{2}$	$\frac{k_M^{'}}{\Omega}$	0.2	0.2
38	$\mathrm{D}_2^\mathrm{R} + \mathrm{D}_{12}^\mathrm{R} \xrightarrow{k_M^{'}} \mathrm{D}_{12}^\mathrm{R} + \mathrm{D}_{12}^\mathrm{R}$	$a_{38} = \frac{k_M^{'}}{\Omega} n_2^R n_{12}^R$	$\frac{k_M^{'}}{\Omega}$	0.2	0.2
39	$\mathrm{D}_{12}^\mathrm{R} + \mathrm{D}^\mathrm{A} \xrightarrow{k_T^{'*}} \mathrm{D}_2^\mathrm{R} + \mathrm{D}^\mathrm{A}$	$a_{39} = \frac{k_T^{'*}}{\Omega} n_{12}^R n^A$	$\frac{k_T^{'*}}{\Omega}$	0.2, 0.16	10, 8

Table O: **Reactions and parameter values used to generate the plots in Fig T.**

$\mathbf{R}_j$	**Reaction**	**Prop.Func.(a_j)**	**Param.**	**Value ($\mathbf{h^{-1}}$)**
1	$\mathrm{D} \xrightarrow{k^A_{W0}} \mathrm{D^A}$	$a_1 = k^A_{W0} n^D$	k^A_{W0}	3.5
2	$\mathrm{D} \xrightarrow{k^A_{W}} \mathrm{D^A}$	$a_2 = k^A_{W} n^D$	k^A_{W}	0
3	$\mathrm{D^A} \xrightarrow{\bar{k}^A_E} \mathrm{D}$	$a_3 = \bar{k}^A_E n^A$	$\bar{k}^A_E$	3
4	$\mathrm{D^A} \xrightarrow{\delta} \mathrm{D}$	$a_4 = \delta n^A$	δ	3
5	$\mathrm{D} + \mathrm{D^A} \xrightarrow{k^A_M} \mathrm{D^A} + \mathrm{D^A}$	$a_5 = \frac{k^A_M}{\Omega} n^D n^A$	$\frac{k^A_M}{\Omega}$	1
6	$\mathrm{D^A} + \mathrm{D^R_1} \xrightarrow{k^A_E} \mathrm{D} + \mathrm{D^R_1}$	$a_6 = \frac{k^A_E}{\Omega} n^A n^R_1$	$\frac{k^A_E}{\Omega}$	1,0.12,0.01,0.001
7	$\mathrm{D^A} + \mathrm{D^R_{12}} \xrightarrow{k^A_E} \mathrm{D} + \mathrm{D^R_{12}}$	$a_7 = \frac{k^A_E}{\Omega} n^A n^R_{12}$	$\frac{k^A_E}{\Omega}$	1,0.12,0.01,0.001
8	$\mathrm{D^A} + \mathrm{D^R_2} \xrightarrow{k^A_E} \mathrm{D} + \mathrm{D^R_2}$	$a_8 = \frac{k^A_E}{\Omega} n^A n^R_2$	$\frac{k^A_E}{\Omega}$	1,0.12,0.01,0.001
9	$\mathrm{D^A} + \mathrm{D^R_{12}} \xrightarrow{k^A_E} \mathrm{D} + \mathrm{D^R_{12}}$	$a_9 = \frac{k^A_E}{\Omega} n^A n^R_{12}$	$\frac{k^A_E}{\Omega}$	1,0.12,0.01,0.001
10	$\mathrm{D} \xrightarrow{k^1_{W0}} \mathrm{D^R_1}$	$a_{10} = k^1_{W0} n^D$	k^1_{W0}	3.5
11	$\mathrm{D} \xrightarrow{k^1_{W}} \mathrm{D^R_1}$	$a_{11} = k^1_{W} n^D$	k^1_{W}	0
12	$\mathrm{D^R_1} \xrightarrow{k'_T} \mathrm{D}$	$a_{12} = k'_T n^R_1$	k'_T	3
13	$\mathrm{D^R_1} \xrightarrow{\delta'} \mathrm{D}$	$a_{13} = \delta' n^R_1$	δ'	3
14	$\mathrm{D} + \mathrm{D^R_2} \xrightarrow{k'_M} \mathrm{D^R_1} + \mathrm{D^R_2}$	$a_{14} = \frac{k'_M}{\Omega} n^D n^R_2$	$\frac{k'_M}{\Omega}$	0.2
15	$\mathrm{D} + \mathrm{D^R_{12}} \xrightarrow{k'_M} \mathrm{D^R_1} + \mathrm{D^R_{12}}$	$a_{15} = \frac{k'_M}{\Omega} n^D n^R_{12}$	$\frac{k'_M}{\Omega}$	0.2
16	$\mathrm{D^R_1} + \mathrm{D^A} \xrightarrow{k'^*_T} \mathrm{D} + \mathrm{D^A}$	$a_{16} = \frac{k'^*_T}{\Omega} n^R_1 n^A$	$\frac{k'^*_T}{\Omega}$	1,0.12,0.01,0.001
17	$\mathrm{D} \xrightarrow{k^2_{W0}} \mathrm{D^R_2}$	$a_{17} = k^2_{W0} n^D$	k^2_{W0}	3.5
18	$\mathrm{D} \xrightarrow{k^2_{W}} \mathrm{D^R_2}$	$a_{18} = k^2_{W} n^D$	k^2_{W}	0
19	$\mathrm{D^R_2} \xrightarrow{\bar{k}^R_E} \mathrm{D}$	$a_{19} = \bar{k}^R_E n^R_2$	$\bar{k}^R_E$	3
20	$\mathrm{D^R_2} \xrightarrow{\delta} \mathrm{D}$	$a_{20} = \delta n^R_2$	δ	3
21	$\mathrm{D} + \mathrm{D^R_2} \xrightarrow{k_M} \mathrm{D^R_2} + \mathrm{D^R_2}$	$a_{21} = \frac{k_M}{\Omega} n^D n^R_2$	$\frac{k_M}{\Omega}$	0.2
22	$\mathrm{D} + \mathrm{D^R_{12}} \xrightarrow{k_M} \mathrm{D^R_2} + \mathrm{D^R_{12}}$	$a_{22} = \frac{k_M}{\Omega} n^D n^R_{12}$	$\frac{k_M}{\Omega}$	0.2
23	$\mathrm{D} + \mathrm{D^R_1} \xrightarrow{\bar{k}_M} \mathrm{D^R_2} + \mathrm{D^R_1}$	$a_{23} = \frac{\bar{k}_M}{\Omega} n^D n^R_1$	$\frac{\bar{k}_M}{\Omega}$	0.2
24	$\mathrm{D} + \mathrm{D^R_{12}} \xrightarrow{\bar{k}_M} \mathrm{D^R_2} + \mathrm{D^R_{12}}$	$a_{24} = \frac{\bar{k}_M}{\Omega} n^D n^R_{12}$	$\frac{\bar{k}_M}{\Omega}$	0.2
25	$\mathrm{D^R_2} + \mathrm{D^A} \xrightarrow{k^R_E} \mathrm{D} + \mathrm{D^A}$	$a_{25} = \frac{k^R_E}{\Omega} n^R_2 n^A$	$\frac{k^R_E}{\Omega}$	1,0.12,0.01,0.001
26	$\mathrm{D^R_1} \xrightarrow{k^2_{W0}} \mathrm{D^R_{12}}$	$a_{26} = k^2_{W0} n^R_1$	k^2_{W0}	3.5
27	$\mathrm{D^R_{12}} \xrightarrow{\bar{k}^R_E} \mathrm{D^R_1}$	$a_{27} = \bar{k}^R_E n^R_{12}$	$\bar{k}^R_E$	3
28	$\mathrm{D^R_{12}} \xrightarrow{\delta} \mathrm{D^R_1}$	$a_{28} = \delta n^R_{12}$	δ	3
29	$\mathrm{D^R_1} + \mathrm{D^R_2} \xrightarrow{k_M} \mathrm{D^R_{12}} + \mathrm{D^R_2}$	$a_{29} = \frac{k_M}{\Omega} n^R_1 n^R_2$	$\frac{k_M}{\Omega}$	0.2
30	$\mathrm{D^R_1} + \mathrm{D^R_{12}} \xrightarrow{k_M} \mathrm{D^R_{12}} + \mathrm{D^R_{12}}$	$a_{30} = \frac{k_M}{\Omega} n^R_1 n^R_{12}$	$\frac{k_M}{\Omega}$	0.2
31	$\mathrm{D^R_1} + \mathrm{D^R_1} \xrightarrow{\bar{k}_M} \mathrm{D^R_{12}} + \mathrm{D^R_1}$	$a_{31} = \frac{\bar{k}_M}{\Omega} \frac{n^R_1(n^R_1-1)}{2}$	$\frac{\bar{k}_M}{\Omega}$	0.2
32	$\mathrm{D^R_1} + \mathrm{D^R_{12}} \xrightarrow{\bar{k}_M} \mathrm{D^R_{12}} + \mathrm{D^R_{12}}$	$a_{32} = \frac{\bar{k}_M}{\Omega} n^R_1 n^R_{12}$	$\frac{\bar{k}_M}{\Omega}$	0.2
33	$\mathrm{D^R_{12}} + \mathrm{D^A} \xrightarrow{k^R_E} \mathrm{D^R_1} + \mathrm{D^A}$	$a_{33} = \frac{k^R_E}{\Omega} n^R_{12} n^A$	$\frac{k^R_E}{\Omega}$	1,0.12,0.01,0.001
34	$\mathrm{D^R_2} \xrightarrow{k^1_{W0}} \mathrm{D^R_{12}}$	$a_{34} = k^1_{W0} n^R_2$	k^1_{W0}	3.5
35	$\mathrm{D^R_{12}} \xrightarrow{k'_T} \mathrm{D^R_2}$	$a_{35} = k'_T n^R_{12}$	k'_T	3
36	$\mathrm{D^R_{12}} \xrightarrow{\delta'} \mathrm{D^R_2}$	$a_{36} = \delta' n^R_{12}$	δ'	3
37	$\mathrm{D^R_2} + \mathrm{D^R_2} \xrightarrow{k'_M} \mathrm{D^R_{12}} + \mathrm{D^R_2}$	$a_{37} = \frac{k'_M}{\Omega} \frac{n^R_2(n^R_2-1)}{2}$	$\frac{k'_M}{\Omega}$	0.2
38	$\mathrm{D^R_2} + \mathrm{D^R_{12}} \xrightarrow{k'_M} \mathrm{D^R_{12}} + \mathrm{D^R_{12}}$	$a_{38} = \frac{k'_M}{\Omega} n^R_2 n^R_{12}$	$\frac{k'_M}{\Omega}$	0.2
39	$\mathrm{D^R_{12}} + \mathrm{D^A} \xrightarrow{k'^*_T} \mathrm{D^R_2} + \mathrm{D^A}$	$a_{39} = \frac{k'^*_T}{\Omega} n^R_{12} n^A$	$\frac{k'^*_T}{\Omega}$	1,0.12,0.01,0.001

Table P: **Reactions and parameter values used to generate the plots in Fig U. The parameter values with $\frac{k^A_E}{\Omega} = \frac{k'^*_T}{\Omega} = \frac{k^R_E}{\Omega} = 1, 0.001$ h^{-1} are also relative to the simulations in Fig 4E.**

R_j	Reaction	Prop.Func.(a_j)	Param.	Value (h^{-1})<br>left plots	Value (h^{-1})<br>right plots
1	$D \xrightarrow{k_{W0}^A} D^A$	$a_1 = k_{W0}^A n^D$	k_{W0}^A	5	5
2	$D \xrightarrow{k_W^A} D^A$	$a_2 = k_W^A n^D$	k_W^A	0	0
3	$D^A \xrightarrow{\bar{k}_E^A} D$	$a_3 = \bar{k}_E^A n^A$	$\bar{k}_E^A$	9, 3	9, 3
4	$D^A \xrightarrow{\delta} D$	$a_4 = \delta n^A$	δ	9, 3	9, 3
5	$D + D^A \xrightarrow{k_M^A} D^A + D^A$	$a_5 = \frac{k_M^A}{\Omega} n^D n^A$	$\frac{k_M^A}{\Omega}$	1	1
6	$D^A + D_1^R \xrightarrow{k_E^A} D + D_1^R$	$a_6 = \frac{k_E^A}{\Omega} n^A n_1^R$	$\frac{k_E^A}{\Omega}$	0.4, 1, 10	0.4, 1, 10
7	$D^A + D_{12}^R \xrightarrow{k_E^A} D + D_{12}^R$	$a_7 = \frac{k_E^A}{\Omega} n^A n_{12}^R$	$\frac{k_E^A}{\Omega}$	0.4, 1, 10	0.4, 1, 10
8	$D^A + D_2^R \xrightarrow{k_E^A} D + D_2^R$	$a_8 = \frac{k_E^A}{\Omega} n^A n_2^R$	$\frac{k_E^A}{\Omega}$	0.4, 1, 10	0.4, 1, 10
9	$D^A + D_{12}^R \xrightarrow{k_E^A} D + D_{12}^R$	$a_9 = \frac{k_E^A}{\Omega} n^A n_{12}^R$	$\frac{k_E^A}{\Omega}$	0.4, 1, 10	0.4, 1, 10
10	$D \xrightarrow{k_{W0}^1} D_1^R$	$a_{10} = k_{W0}^1 n^D$	k_{W0}^1	5	5
11	$D \xrightarrow{k_W^1} D_1^R$	$a_{11} = k_W^1 n^D$	k_W^1	0	0
12	$D_1^R \xrightarrow{k_T^{'}} D$	$a_{12} = k_T^{'} n_1^R$	$k_T^{'}$	9, 4.5<br>3, 1.5	9, 4.5 (left side)<br>3, 1.5 (right side)
13	$D_1^R \xrightarrow{\delta^{'}} D$	$a_{13} = \delta^{'} n_1^R$	$\delta^{'}$	9, 4.5<br>3, 1.5	9, 4.5 (left side)<br>3, 1.5 (right side)
14	$D + D_2^R \xrightarrow{k_M^{'}} D_1^R + D_2^R$	$a_{14} = \frac{k_M^{'}}{\Omega} n^D n_2^R$	$\frac{k_M^{'}}{\Omega}$	0.2	0.2
15	$D + D_{12}^R \xrightarrow{k_M^{'}} D_1^R + D_{12}^R$	$a_{15} = \frac{k_M^{'}}{\Omega} n^D n_{12}^R$	$\frac{k_M^{'}}{\Omega}$	0.2	0.2
16	$D_1^R + D^A \xrightarrow{k_T^{'*}} D + D^A$	$a_{16} = \frac{k_T^{'*}}{\Omega} n_1^R n^A$	$\frac{k_T^{'*}}{\Omega}$	0.4, 0.2 - 1,0.5 - 10,5	0.4, 0.2 - 1,0.5 - 10,5
17	$D \xrightarrow{k_{W0}^2} D_2^R$	$a_{17} = k_{W0}^2 n^D$	k_{W0}^2	5	5
18	$D \xrightarrow{k_W^2} D_2^R$	$a_{18} = k_W^2 n^D$	k_W^2	0	0
19	$D_2^R \xrightarrow{\bar{k}_E^R} D$	$a_{19} = \bar{k}_E^R n_2^R$	$\bar{k}_E^R$	9, 3	9, 3
20	$D_2^R \xrightarrow{\delta} D$	$a_{20} = \delta n_2^R$	δ	9, 3	9, 3
21	$D + D_2^R \xrightarrow{k_M} D_2^R + D_2^R$	$a_{21} = \frac{k_M}{\Omega} n^D n_2^R$	$\frac{k_M}{\Omega}$	0.2	0.2
22	$D + D_{12}^R \xrightarrow{k_M} D_2^R + D_{12}^R$	$a_{22} = \frac{k_M}{\Omega} n^D n_{12}^R$	$\frac{k_M}{\Omega}$	0.2	0.2
23	$D + D_1^R \xrightarrow{\bar{k}_M} D_2^R + D_1^R$	$a_{23} = \frac{\bar{k}_M}{\Omega} n^D n_1^R$	$\frac{\bar{k}_M}{\Omega}$	0.2	0.2
24	$D + D_{12}^R \xrightarrow{\bar{k}_M} D_2^R + D_{12}^R$	$a_{24} = \frac{\bar{k}_M}{\Omega} n^D n_{12}^R$	$\frac{\bar{k}_M}{\Omega}$	0.2	0.2
25	$D_2^R + D^A \xrightarrow{k_E^R} D + D^A$	$a_{25} = \frac{k_E^R}{\Omega} n_2^R n^A$	$\frac{k_E^R}{\Omega}$	0.4, 1, 10	0.4, 1, 10
26	$D_1^R \xrightarrow{k_{W0}^2} D_{12}^R$	$a_{26} = k_{W0}^2 n_1^R$	k_{W0}^2	5	5
27	$D_{12}^R \xrightarrow{\bar{k}_E^R} D_1^R$	$a_{27} = \bar{k}_E^R n_{12}^R$	$\bar{k}_E^R$	9, 3	9, 3
28	$D_{12}^R \xrightarrow{\delta} D_1^R$	$a_{28} = \delta n_{12}^R$	δ	9, 3	9, 3
29	$D_1^R + D_2^R \xrightarrow{k_M} D_{12}^R + D_2^R$	$a_{29} = \frac{k_M}{\Omega} n_1^R n_2^R$	$\frac{k_M}{\Omega}$	0.2	0.2
30	$D_1^R + D_{12}^R \xrightarrow{k_M} D_{12}^R + D_{12}^R$	$a_{30} = \frac{k_M}{\Omega} n_1^R n_{12}^R$	$\frac{k_M}{\Omega}$	0.2	0.2
31	$D_1^R + D_1^R \xrightarrow{\bar{k}_M} D_{12}^R + D_1^R$	$a_{31} = \frac{\bar{k}_M}{\Omega} \frac{n_1^R(n_1^R-1)}{2}$	$\frac{\bar{k}_M}{\Omega}$	0.2	0.2
32	$D_1^R + D_{12}^R \xrightarrow{\bar{k}_M} D_{12}^R + D_{12}^R$	$a_{32} = \frac{\bar{k}_M}{\Omega} n_1^R n_{12}^R$	$\frac{\bar{k}_M}{\Omega}$	0.2	0.2
33	$D_{12}^R + D^A \xrightarrow{k_E^R} D_1^R + D^A$	$a_{33} = \frac{k_E^R}{\Omega} n_{12}^R n^A$	$\frac{k_E^R}{\Omega}$	0.4, 1, 10	0.4, 1, 10
34	$D_2^R \xrightarrow{k_{W0}^1} D_{12}^R$	$a_{34} = k_{W0}^1 n_2^R$	k_{W0}^1	5	5
35	$D_{12}^R \xrightarrow{k_T^{'}} D_2^R$	$a_{35} = k_T^{'} n_{12}^R$	$k_T^{'}$	9, 4.5<br>3, 1.5	9, 4.5 (left side)<br>3, 1.5 (right side)
36	$D_{12}^R \xrightarrow{\delta^{'}} D_2^R$	$a_{36} = \delta^{'} n_{12}^R$	$\delta^{'}$	9, 4.5<br>3, 1.5	9, 4.5 (left side)<br>3, 1.5 (right side)
37	$D_2^R + D_2^R \xrightarrow{k_M^{'}} D_{12}^R + D_2^R$	$a_{37} = \frac{k_M^{'}}{\Omega} \frac{n_2^R(n_2^R-1)}{2}$	$\frac{k_M^{'}}{\Omega}$	0.2	0.2
38	$D_2^R + D_{12}^R \xrightarrow{k_M^{'}} D_{12}^R + D_{12}^R$	$a_{38} = \frac{k_M^{'}}{\Omega} n_2^R n_{12}^R$	$\frac{k_M^{'}}{\Omega}$	0.2	0.2
39	$D_{12}^R + D^A \xrightarrow{k_T^{'*}} D_2^R + D^A$	$a_{39} = \frac{k_T^{'*}}{\Omega} n_{12}^R n^A$	$\frac{k_T^{'*}}{\Omega}$	0.4, 0.2 - 1,0.5 - 10,5	0.4, 0.2 - 1,0.5 - 10,5

Table Q: **Reactions and parameter values used to generate the plots in Fig V.**

$\mathbf{R}_j$	**Reaction**	**Prop.Func.**(a_j)	**Param.**	**Value** ($\mathbf{h}^{-1}$)<br>lower plots	**Value** ($\mathbf{h}^{-1}$)<br>upper plots<br>left plots	**Value** ($\mathbf{h}^{-1}$)<br>upper plots<br>right plots
1	$\mathrm{D} \xrightarrow{k_{W0}^A} \mathrm{D^A}$	$a_1 = k_{W0}^A n^D$	k_{W0}^A	5	5	5
2	$\mathrm{D} \xrightarrow{k_W^A} \mathrm{D^A}$	$a_2 = k_W^A n^D$	k_W^A	81	81	81
3	$\mathrm{D^A} \xrightarrow{\bar{k}_E^A} \mathrm{D}$	$a_3 = \bar{k}_E^A n^A$	$\bar{k}_E^A$	12, 4	12, 4	12, 4
4	$\mathrm{D^A} \xrightarrow{\delta} \mathrm{D}$	$a_4 = \delta n^A$	δ	12, 4	12, 4	12, 4
5	$\mathrm{D} + \mathrm{D^A} \xrightarrow{k_M^A} \mathrm{D^A} + \mathrm{D^A}$	$a_5 = \frac{k_M^A}{\Omega} n^D n^A$	$\frac{k_M^A}{\Omega}$	1	1	1
6	$\mathrm{D^A} + \mathrm{D_1^R} \xrightarrow{k_E^A} \mathrm{D} + \mathrm{D_1^R}$	$a_6 = \frac{k_E^A}{\Omega} n^A n_1^R$	$\frac{k_E^A}{\Omega}$	1	0.3	5
7	$\mathrm{D^A} + \mathrm{D_{12}^R} \xrightarrow{k_E^A} \mathrm{D} + \mathrm{D_{12}^R}$	$a_7 = \frac{k_E^A}{\Omega} n^A n_{12}^R$	$\frac{k_E^A}{\Omega}$	1	0.3	5
8	$\mathrm{D^A} + \mathrm{D_2^R} \xrightarrow{k_E^A} \mathrm{D} + \mathrm{D_2^R}$	$a_8 = \frac{k_E^A}{\Omega} n^A n_2^R$	$\frac{k_E^A}{\Omega}$	1	0.3	5
9	$\mathrm{D^A} + \mathrm{D_{12}^R} \xrightarrow{k_E^A} \mathrm{D} + \mathrm{D_{12}^R}$	$a_9 = \frac{k_E^A}{\Omega} n^A n_{12}^R$	$\frac{k_E^A}{\Omega}$	1	0.3	5
10	$\mathrm{D} \xrightarrow{k_{W0}^1} \mathrm{D_1^R}$	$a_{10} = k_{W0}^1 n^D$	k_{W0}^1	5	5	5
11	$\mathrm{D} \xrightarrow{k_W^1} \mathrm{D_1^R}$	$a_{11} = k_W^1 n^D$	k_W^1	0	0	0
12	$\mathrm{D_1^R} \xrightarrow{k_T^{'}} \mathrm{D}$	$a_{12} = k_T^{'} n_1^R$	$k_T^{'}$	7.2, 2.4<br>2.4, 0.8	7.2, 2.4<br>2.4, 0.8	7.2, 2.4 (left side)<br>2.4, 0.8 (right side)
13	$\mathrm{D_1^R} \xrightarrow{\delta^{'}} \mathrm{D}$	$a_{13} = \delta^{'} n_1^R$	$\delta^{'}$	7.2, 2.4<br>2.4, 0.8	7.2, 2.4<br>2.4, 0.8	7.2, 2.4 (left side)<br>2.4, 0.8 (right side)
14	$\mathrm{D} + \mathrm{D_2^R} \xrightarrow{k_M^{'}} \mathrm{D_1^R} + \mathrm{D_2^R}$	$a_{14} = \frac{k_M^{'}}{\Omega} n^D n_2^R$	$\frac{k_M^{'}}{\Omega}$	0.2	0.2	0.2
15	$\mathrm{D} + \mathrm{D_{12}^R} \xrightarrow{k_M^{'}} \mathrm{D_1^R} + \mathrm{D_{12}^R}$	$a_{15} = \frac{k_M^{'}}{\Omega} n^D n_{12}^R$	$\frac{k_M^{'}}{\Omega}$	0.2	0.2	0.2
16	$\mathrm{D_1^R} + \mathrm{D^A} \xrightarrow{k_T^{'*}} \mathrm{D} + \mathrm{D^A}$	$a_{16} = \frac{k_T^{'*}}{\Omega} n_1^R n^A$	$\frac{k_T^{'*}}{\Omega}$	0.6, 0.2	0.18, 0.06	3, 1
17	$\mathrm{D} \xrightarrow{k_{W0}^2} \mathrm{D_2^R}$	$a_{17} = k_{W0}^2 n^D$	k_{W0}^2	5	5	5
18	$\mathrm{D} \xrightarrow{k_W^2} \mathrm{D_2^R}$	$a_{18} = k_W^2 n^D$	k_W^2	0	0	0
19	$\mathrm{D_2^R} \xrightarrow{\bar{k}_E^R} \mathrm{D}$	$a_{19} = \bar{k}_E^R n_2^R$	$\bar{k}_E^R$	12, 4	12, 4	12, 4
20	$\mathrm{D_2^R} \xrightarrow{\delta} \mathrm{D}$	$a_{20} = \delta n_2^R$	δ	12, 4	12, 4	12, 4
21	$\mathrm{D} + \mathrm{D_2^R} \xrightarrow{k_M} \mathrm{D_2^R} + \mathrm{D_2^R}$	$a_{21} = \frac{k_M}{\Omega} n^D n_2^R$	$\frac{k_M}{\Omega}$	0.2	0.2	0.2
22	$\mathrm{D} + \mathrm{D_{12}^R} \xrightarrow{k_M} \mathrm{D_2^R} + \mathrm{D_{12}^R}$	$a_{22} = \frac{k_M}{\Omega} n^D n_{12}^R$	$\frac{k_M}{\Omega}$	0.2	0.2	0.2
23	$\mathrm{D} + \mathrm{D_1^R} \xrightarrow{\bar{k}_M} \mathrm{D_2^R} + \mathrm{D_1^R}$	$a_{23} = \frac{\bar{k}_M}{\Omega} n^D n_1^R$	$\frac{\bar{k}_M}{\Omega}$	0.2	0.2	0.2
24	$\mathrm{D} + \mathrm{D_{12}^R} \xrightarrow{\bar{k}_M} \mathrm{D_2^R} + \mathrm{D_{12}^R}$	$a_{24} = \frac{\bar{k}_M}{\Omega} n^D n_{12}^R$	$\frac{\bar{k}_M}{\Omega}$	0.2	0.2	0.2
25	$\mathrm{D_2^R} + \mathrm{D^A} \xrightarrow{k_E^R} \mathrm{D} + \mathrm{D^A}$	$a_{25} = \frac{k_E^R}{\Omega} n_2^R n^A$	$\frac{k_E^R}{\Omega}$	1	0.3	5
26	$\mathrm{D_1^R} \xrightarrow{k_{W0}^2} \mathrm{D_{12}^R}$	$a_{26} = k_{W0}^2 n_1^R$	k_{W0}^2	5	5	5
27	$\mathrm{D_{12}^R} \xrightarrow{\bar{k}_E^R} \mathrm{D_1^R}$	$a_{27} = \bar{k}_E^R n_{12}^R$	$\bar{k}_E^R$	12, 4	12, 4	12, 4
28	$\mathrm{D_{12}^R} \xrightarrow{\delta} \mathrm{D_1^R}$	$a_{28} = \delta n_{12}^R$	δ	12, 4	12, 4	12, 4
29	$\mathrm{D_1^R} + \mathrm{D_2^R} \xrightarrow{k_M} \mathrm{D_{12}^R} + \mathrm{D_2^R}$	$a_{29} = \frac{k_M}{\Omega} n_1^R n_2^R$	$\frac{k_M}{\Omega}$	0.2	0.2	0.2
30	$\mathrm{D_1^R} + \mathrm{D_{12}^R} \xrightarrow{k_M} \mathrm{D_{12}^R} + \mathrm{D_{12}^R}$	$a_{30} = \frac{k_M}{\Omega} n_1^R n_{12}^R$	$\frac{k_M}{\Omega}$	0.2	0.2	0.2
31	$\mathrm{D_1^R} + \mathrm{D_1^R} \xrightarrow{\bar{k}_M} \mathrm{D_{12}^R} + \mathrm{D_1^R}$	$a_{31} = \frac{\bar{k}_M}{\Omega} \frac{n_1^R(n_1^R-1)}{2}$	$\frac{\bar{k}_M}{\Omega}$	0.2	0.2	0.2
32	$\mathrm{D_1^R} + \mathrm{D_{12}^R} \xrightarrow{\bar{k}_M} \mathrm{D_{12}^R} + \mathrm{D_{12}^R}$	$a_{32} = \frac{\bar{k}_M}{\Omega} n_1^R n_{12}^R$	$\frac{\bar{k}_M}{\Omega}$	0.2	0.2	0.2
33	$\mathrm{D_{12}^R} + \mathrm{D^A} \xrightarrow{k_E^R} \mathrm{D_1^R} + \mathrm{D^A}$	$a_{33} = \frac{k_E^R}{\Omega} n_{12}^R n^A$	$\frac{k_E^R}{\Omega}$	1	0.3	5
34	$\mathrm{D_2^R} \xrightarrow{k_{W0}^1} \mathrm{D_{12}^R}$	$a_{34} = k_{W0}^1 n_2^R$	k_{W0}^1	5	5	5
35	$\mathrm{D_{12}^R} \xrightarrow{k_T^{'}} \mathrm{D_2^R}$	$a_{35} = k_T^{'} n_{12}^R$	$k_T^{'}$	7.2, 2.4<br>2.4, 0.8	7.2, 2.4<br>2.4, 0.8	7.2, 2.4 (left side)<br>2.4, 0.8 (right side)
36	$\mathrm{D_{12}^R} \xrightarrow{\delta^{'}} \mathrm{D_2^R}$	$a_{36} = \delta^{'} n_{12}^R$	$\delta^{'}$	7.2, 2.4<br>2.4, 0.8	7.2, 2.4<br>2.4, 0.8	7.2, 2.4 (left side)<br>2.4, 0.8 (right side)
37	$\mathrm{D_2^R} + \mathrm{D_2^R} \xrightarrow{k_M^{'}} \mathrm{D_{12}^R} + \mathrm{D_2^R}$	$a_{37} = \frac{k_M^{'}}{\Omega} \frac{n_2^R(n_2^R-1)}{2}$	$\frac{k_M^{'}}{\Omega}$	0.2	0.2	0.2
38	$\mathrm{D_2^R} + \mathrm{D_{12}^R} \xrightarrow{k_M^{'}} \mathrm{D_{12}^R} + \mathrm{D_{12}^R}$	$a_{38} = \frac{k_M^{'}}{\Omega} n_2^R n_{12}^R$	$\frac{k_M^{'}}{\Omega}$	0.2	0.2	0.2
39	$\mathrm{D_{12}^R} + \mathrm{D^A} \xrightarrow{k_T^{'*}} \mathrm{D_2^R} + \mathrm{D^A}$	$a_{39} = \frac{k_T^{'*}}{\Omega} n_{12}^R n^A$	$\frac{k_T^{'*}}{\Omega}$	0.6, 0.2	0.18, 0.06	3, 1

Table R: **Reactions and parameter values used to generate the plots in Fig W.**

R_j	**Reaction**	**Prop.Func.**(a_j)	**Param.**	**Value** (h^{-1}) Fig YB	**Value** (h^{-1}) Fig YC	**Value** (h^{-1}) Fig YE	**Value** (h^{-1}) Fig YF
1	$\mathrm{D} \xrightarrow{k_{W0}^{A}} \mathrm{D^A}$	$a_1 = k_{W0}^{A} n^D$	k_{W0}^{A}	0.14	0.14	0.14	0.14
2	$\mathrm{D} \xrightarrow{k_{W}^{A}} \mathrm{D^A}$	$a_2 = k_{W}^{A} n^D$	k_{W}^{A}	0.7280	0.7280	0.7280	0.7280
3	$\mathrm{D^A} \xrightarrow{\bar{k}_{E}^{A}} \mathrm{D}$	$a_3 = \bar{k}_{E}^{A} n^A$	$\bar{k}_{E}^{A}$	0.0788	0.0788	0.0788	0.0788
4	$\mathrm{D^A} \xrightarrow{\delta} \mathrm{D}$	$a_4 = \delta n^A$	δ	0.0263	0.0526	0.0263	0.0263
5	$\mathrm{D} + \mathrm{D^A} \xrightarrow{k_{M}^{A}} \mathrm{D^A} + \mathrm{D^A}$	$a_5 = \frac{k_{M}^{A}}{\Omega} n^D n^A$	$\frac{k_{M}^{A}}{\Omega}$	0.07	0.07	0.07	0.07
6	$\mathrm{D^A} + \mathrm{D_1^R} \xrightarrow{k_{E}^{A}} \mathrm{D} + \mathrm{D_1^R}$	$a_6 = \frac{k_{E}^{A}}{\Omega} n^A n_1^R$	$\frac{k_{E}^{A}}{\Omega}$	0.07	0.07	0.07	0.07
7	$\mathrm{D^A} + \mathrm{D_{12}^R} \xrightarrow{k_{E}^{A}} \mathrm{D} + \mathrm{D_{12}^R}$	$a_7 = \frac{k_{E}^{A}}{\Omega} n^A n_{12}^R$	$\frac{k_{E}^{A}}{\Omega}$	0.07	0.07	0.07	0.07
8	$\mathrm{D^A} + \mathrm{D_2^R} \xrightarrow{k_{E}^{A}} \mathrm{D} + \mathrm{D_2^R}$	$a_8 = \frac{k_{E}^{A}}{\Omega} n^A n_2^R$	$\frac{k_{E}^{A}}{\Omega}$	0.07	0.07	0.07	0.07
9	$\mathrm{D^A} + \mathrm{D_{12}^R} \xrightarrow{k_{E}^{A}} \mathrm{D} + \mathrm{D_{12}^R}$	$a_9 = \frac{k_{E}^{A}}{\Omega} n^A n_{12}^R$	$\frac{k_{E}^{A}}{\Omega}$	0.07	0.07	0.07	0.07
10	$\mathrm{D} \xrightarrow{k_{W0}^{1}} \mathrm{D_1^R}$	$a_{10} = k_{W0}^{1} n^D$	k_{W0}^{1}	0.14	0.14	0.14	0.14
11	$\mathrm{D} \xrightarrow{k_{W}^{1}} \mathrm{D_1^R}$	$a_{11} = k_{W}^{1} n^D$	k_{W}^{1}	0	0	0	0
12	$\mathrm{D_1^R} \xrightarrow{k_{T}^{'}} \mathrm{D}$	$a_{12} = k_{T}^{'} n_1^R$	$k_{T}^{'}$	0.0158	0.0158	0.0158	0.0394
13	$\mathrm{D_1^R} \xrightarrow{\delta^{'}} \mathrm{D}$	$a_{13} = \delta^{'} n_1^R$	$\delta^{'}$	0.0053	0.0106	0.0053	0.0132
14	$\mathrm{D} + \mathrm{D_2^R} \xrightarrow{k_{M}^{'}} \mathrm{D_1^R} + \mathrm{D_2^R}$	$a_{14} = \frac{k_{M}^{'}}{\Omega} n^D n_2^R$	$\frac{k_{M}^{'}}{\Omega}$	0.0014	0.0014	0.0014	0.0014
15	$\mathrm{D} + \mathrm{D_{12}^R} \xrightarrow{k_{M}^{'}} \mathrm{D_1^R} + \mathrm{D_{12}^R}$	$a_{15} = \frac{k_{M}^{'}}{\Omega} n^D n_{12}^R$	$\frac{k_{M}^{'}}{\Omega}$	0.0014	0.0014	0.0014	0.0014
16	$\mathrm{D_1^R} + \mathrm{D^A} \xrightarrow{k_{T}^{'*}} \mathrm{D} + \mathrm{D^A}$	$a_{16} = \frac{k_{T}^{'*}}{\Omega} n_1^R n^A$	$\frac{k_{T}^{'*}}{\Omega}$	0.0014	0.0014	0.0014	0.0014
17	$\mathrm{D} \xrightarrow{k_{W0}^{2}} \mathrm{D_2^R}$	$a_{17} = k_{W0}^{2} n^D$	k_{W0}^{2}	0.14	0.14	0.14	0.14
18	$\mathrm{D} \xrightarrow{k_{W}^{2}} \mathrm{D_2^R}$	$a_{18} = k_{W}^{2} n^D$	k_{W}^{2}	0	0	0	0
19	$\mathrm{D_2^R} \xrightarrow{\bar{k}_{E}^{R}} \mathrm{D}$	$a_{19} = \bar{k}_{E}^{R} n_2^R$	$\bar{k}_{E}^{R}$	0.0788	0.0788	0.0788	0.0788
20	$\mathrm{D_2^R} \xrightarrow{\delta} \mathrm{D}$	$a_{20} = \delta n_2^R$	δ	0.0263	0.0526	0.0263	0.0263
21	$\mathrm{D} + \mathrm{D_2^R} \xrightarrow{k_{M}} \mathrm{D_2^R} + \mathrm{D_2^R}$	$a_{21} = \frac{k_{M}}{\Omega} n^D n_2^R$	$\frac{k_{M}}{\Omega}$	0.0014	0.0014	0.0014	0.0014
22	$\mathrm{D} + \mathrm{D_{12}^R} \xrightarrow{k_{M}} \mathrm{D_2^R} + \mathrm{D_{12}^R}$	$a_{22} = \frac{k_{M}}{\Omega} n^D n_{12}^R$	$\frac{k_{M}}{\Omega}$	0.0014	0.0014	0.0014	0.0014
23	$\mathrm{D} + \mathrm{D_1^R} \xrightarrow{\bar{k}_{M}} \mathrm{D_2^R} + \mathrm{D_1^R}$	$a_{23} = \frac{\bar{k}_{M}}{\Omega} n^D n_1^R$	$\frac{\bar{k}_{M}}{\Omega}$	0.0014	0.0014	0.0014	0.0014
24	$\mathrm{D} + \mathrm{D_{12}^R} \xrightarrow{\bar{k}_{M}} \mathrm{D_2^R} + \mathrm{D_{12}^R}$	$a_{24} = \frac{\bar{k}_{M}}{\Omega} n^D n_{12}^R$	$\frac{\bar{k}_{M}}{\Omega}$	0.0014	0.0014	0.0014	0.0014
25	$\mathrm{D_2^R} + \mathrm{D^A} \xrightarrow{k_{E}^{R}} \mathrm{D} + \mathrm{D^A}$	$a_{25} = \frac{k_{E}^{R}}{\Omega} n_2^R n^A$	$\frac{k_{E}^{R}}{\Omega}$	0.07	0.07	0.07	0.07
26	$\mathrm{D_1^R} \xrightarrow{k_{W0}^{2}} \mathrm{D_{12}^R}$	$a_{26} = k_{W0}^{2} n_1^R$	k_{W0}^{2}	0.14	0.14	0.14	0.14
27	$\mathrm{D_{12}^R} \xrightarrow{\bar{k}_{E}^{R}} \mathrm{D_1^R}$	$a_{27} = \bar{k}_{E}^{R} n_{12}^R$	$\bar{k}_{E}^{R}$	0.0788	0.0788	0.0788	0.0788
28	$\mathrm{D_{12}^R} \xrightarrow{\delta} \mathrm{D_1^R}$	$a_{28} = \delta n_{12}^R$	δ	0.0263	0.0526	0.0263	0.0263
29	$\mathrm{D_1^R} + \mathrm{D_2^R} \xrightarrow{k_{M}} \mathrm{D_{12}^R} + \mathrm{D_2^R}$	$a_{29} = \frac{k_{M}}{\Omega} n_1^R n_2^R$	$\frac{k_{M}}{\Omega}$	0.0014	0.0014	0.0014	0.0014
30	$\mathrm{D_1^R} + \mathrm{D_{12}^R} \xrightarrow{k_{M}} \mathrm{D_{12}^R} + \mathrm{D_{12}^R}$	$a_{30} = \frac{k_{M}}{\Omega} n_1^R n_{12}^R$	$\frac{k_{M}}{\Omega}$	0.0014	0.0014	0.0014	0.0014
31	$\mathrm{D_1^R} + \mathrm{D_1^R} \xrightarrow{\bar{k}_{M}} \mathrm{D_{12}^R} + \mathrm{D_1^R}$	$a_{31} = \frac{\bar{k}_{M}}{\Omega} \frac{n_1^R(n_1^R-1)}{2}$	$\frac{\bar{k}_{M}}{\Omega}$	0.0014	0.0014	0.0014	0.0014
32	$\mathrm{D_1^R} + \mathrm{D_{12}^R} \xrightarrow{\bar{k}_{M}} \mathrm{D_{12}^R} + \mathrm{D_{12}^R}$	$a_{32} = \frac{\bar{k}_{M}}{\Omega} n_1^R n_{12}^R$	$\frac{\bar{k}_{M}}{\Omega}$	0.0014	0.0014	0.0014	0.0014
33	$\mathrm{D_{12}^R} + \mathrm{D^A} \xrightarrow{k_{E}^{R}} \mathrm{D_1^R} + \mathrm{D^A}$	$a_{33} = \frac{k_{E}^{R}}{\Omega} n_{12}^R n^A$	$\frac{k_{E}^{R}}{\Omega}$	0.07	0.07	0.07	0.07
34	$\mathrm{D_2^R} \xrightarrow{k_{W0}^{1}} \mathrm{D_{12}^R}$	$a_{34} = k_{W0}^{1} n_2^R$	k_{W0}^{1}	0.14	0.14	0.14	0.14
35	$\mathrm{D_{12}^R} \xrightarrow{k_{T}^{'}} \mathrm{D_2^R}$	$a_{35} = k_{T}^{'} n_{12}^R$	$k_{T}^{'}$	0.0158	0.0158	0.0158	0.0394
36	$\mathrm{D_{12}^R} \xrightarrow{\delta^{'}} \mathrm{D_2^R}$	$a_{36} = \delta^{'} n_{12}^R$	$\delta^{'}$	0.0053	0.0106	0.0053	0.0132
37	$\mathrm{D_2^R} + \mathrm{D_2^R} \xrightarrow{k_{M}^{'}} \mathrm{D_{12}^R} + \mathrm{D_2^R}$	$a_{37} = \frac{k_{M}^{'}}{\Omega} \frac{n_2^R(n_2^R-1)}{2}$	$\frac{k_{M}^{'}}{\Omega}$	0.0014	0.0014	0.0014	0.0014
38	$\mathrm{D_2^R} + \mathrm{D_{12}^R} \xrightarrow{k_{M}^{'}} \mathrm{D_{12}^R} + \mathrm{D_{12}^R}$	$a_{38} = \frac{k_{M}^{'}}{\Omega} n_2^R n_{12}^R$	$\frac{k_{M}^{'}}{\Omega}$	0.0014	0.0014	0.0014	0.0014
39	$\mathrm{D_{12}^R} + \mathrm{D^A} \xrightarrow{k_{T}^{'*}} \mathrm{D_2^R} + \mathrm{D^A}$	$a_{39} = \frac{k_{T}^{'*}}{\Omega} n_{12}^R n^A$	$\frac{k_{T}^{'*}}{\Omega}$	0.0014	0.0014	0.0014	0.0014

Table S: **Reactions and parameter values used to generate the plots in Fig Y.**

References

[1] A. J. Courey. *Mechanisms in Trancriptional Regulation.* Blackwell Publishing, 2008.

[2] R. E. Kingston and J. W. Tamkun. “Transcriptional Regulation by Trithorax-Group Proteins”. In: *Cold Spring Harbor Laboratory Press* 6 (2014).

[3] Legube Gaëlle and Didier Trouche. “Regulating histone acetyltransferases and deacetylases”. In: *EMBO reports* 4 (2003).

[4] Paola Cruz-Tapias et al. “The H3K9 MethylationWriter SETDB1 and Its Reader MPP8 Cooperate to Silence Satellite DNA Repeats in Mouse Embryonic Stem Cells”. In: *Genes* 10 (2019).

[5] C. D. Allis et al. *Epigenetics.* Cold Spring Harbor Laboratory Press, Second Edition, 2015.

[6] Nathaniel A. Hathaway et al. “Dynamics and Memory of Heterochromatin in Living Cells”. In: *Cell* 149 (2012).

[7] M. Trerotola et al. “Epigenetic inheritance and the missing heritability”. In: *Human Genomics* 9 (2015).

[8] Tianyi Zhang, Sarah Cooper, and Neil Brockdorff. “The interplay of histone modifications – writers that read”. In: *EMBO Reports* 16 (2015).

[9] E. I. Campos, J. M. Stafford, and D. Reinberg. “Epigenetic Inheritance: Histone Bookmarks Across Generations”. In: *Trends Cell Biol* 24 (2014).

[10] C. Alabert et al. “Two distinct modes for propagation of histone PTMs across the cell cycle”. In: *Research Communication, CSHL* (2015).

[11] Ian B. Dodd et al. “Theoretical Analysis of Epigenetic Cell Memory by Nucleosome Modification”. In: *Cell* 129 (2007).

[12] Diana David-Rus et al. “Inheritance ofepigeneticchromatinsilencing”. In: *J. Theor. Biol.* (2009).

[13] Katharina Müller-Ott et al. “Specificity, propagation, and memory of pericentric heterochromatin”. In: *Mol. Syst. Biol.* (2014).

[14] S. Huang, M. Litt, and C. A. Blakey. *Epigenetic Gene Expression and Regulation.* Academic Press, 2015.

[15] TaeSoo Kim and Stephen Buratowski. “Dimethylation of H3K4 by Set1 Recruits the Set3 Histone Deacetylase Complex to 50 Transcribed Regions”. In: *Cell* 137 (2009).

[16] Yen-Sin Ang et al. “Wdr5 Mediates Self-Renewal and Reprogramming via the Embryonic Stem Cell Core Transcriptional Network”. In: *Cell* 145 (2011).

[17] Tomas Stopka et al. “PU.1 inhibits the erythroid program by binding to GATA-1 on DNA and creating a repressive chromatin structure”. In: *The EMBO Journal* 24 (2005).

[18] Joanna Wysocka et al. “Human Sin3 deacetylase and trithorax-related Set1/Ash2 histone H3-K4 methyltransferase are tethered together selectively by the cell-proliferation factor HCF-1”. In: *GENES & DEVELOPMENT* 17 (2003).

[19] Olivier Binda et al. “Trimethylation of histone H3 lysine 4 impairs methylation of histone H3 lysine 9”. In: *Epigenetics* 5 (2010).

[20] Anup K. Upadhyay et al. “Coordinated Methyl-lysine Erasure: Structural and Functional Linkage of a Jumonji demethylasedomain and a Reader domain”. In: *Curr Opin Struct Biol* 21 (2011).

[21] Makiko Iwafuchi-Doi and Kenneth S. Zaret. “Pioneer transcription factors in cell reprogramming”. In: *GENES & DEVELOPMENT* 28 (2014).

[22] Andrew P.McGovern, BenjaminE.Powell, and TimothyJ.T.Chevassut. “A dynamic multi-compartmental model of DNAmethylation with demonstrable predictive value in hematological malignancies”. In: *J. Theoretical Biol.* 310 (2012).

[23] Ferdinand von Meyenn et al. “Impairment of DNA Methylation Maintenance Is the Main Cause of Global Demethylation in Naive Embryonic Stem Cells”. In: *Mol. Cell* 62 (2016).

[24] Laura B. Sontag, Matthew C. Lorincz, and E. Georg Luebeckc. “Dynamics, stability and inheritance of somatic DNA methylation imprints”. In: *J. of Theoretical Biology* 242 (2006).

[25] Sarah P. Otto and Virginia Walbot. “DNA Methylation in Eukaryotes: Kinetics of Demethylation and de Novo Methylation During the Life Cycle”. In: *Genetics* 124 (1990).

[26] Melany Jackson et al. “Severe Global DNA Hypomethylation Blocks Differentiation and Induces Histone Hyperacetylation in Embryonic Stem Cells”. In: *MOLECULAR AND CELLULAR BIOLOGY* (2004).

[27] Shinsuke Ito et al. “Tet proteins can convert 5-methylcytosine to 5-formylcytosine and 5-carboxylcytosine”. In: *Science* 333 (2011).

[28] Anne K. Ludwig et al. “Binding of MBD proteins to DNA blocks Tet1 function thereby modulating transcriptional noise”. In: *Nucleic Acid Research* 45 (2017).

[29] Yoach Rais1 et al. “Deterministic direct reprogramming of somatic cells to pluripotency”. In: *Nature* (2013).

[30] MIN LUO et al. “NuRD Blocks Reprogramming of Mouse Somatic Cells into Pluripotent Stem Cells”. In: *Stem Cells* 31 (2013).

[31] Bergstrom CT Genereux DP Miner BE and Laird CD. “A population-epigenetic model to infer site-specific methylation rates from double-stranded DNA methylation patterns”. In: *Proc. Natl. Acad. Sci* 102 (2005).

[32] Masaki Okano et al. “DNA Methyltransferases Dnmt3a and Dnmt3b Are Essential for De Novo Methylation and Mammalian Development”. In: *Cel* 99 (1999).

[33] Cartron PF Hervouet E Vallette FM. “Dnmt3/transcription factor interactions as crucial players in targeted DNA methylation”. In: *Epigeneticsi* 4 (2009).

[34] C. Brenner, R. Deplus, and C. Didelot et al. “Myc represses transcription through recruitment of DNA methyltransferase corepressor”. In: *EMBO J* 24 (2005).

[35] Xu J Zhang W. “DNA methyltransferases and their roles in tumorigenesis”. In: *Biomark Res.* 5 (2017).

[36] Tom Latham, Nick Gilbert, and Bernard Ramsahoye. “DNA methylation in mouse embryonic stem cells and development”. In: *Cell Tissue Research* 331 (2008).

[37] Kasper Dindler Rasmussen and Kristian Helin. “Role of TET enzymes in DNA methylation, development, and cancer”. In: *GENES & DEVELOPMENT* 30 (2016).

[38] Chunlei Jin et al. “TET1 is a maintenance DNA demethylase that prevents methylation spreading in differentiated cells”. In: *Nucleic Acids Research* 42 (2014).

[39] Melany Jackson et al. “Severe Global DNA Hypomethylation Blocks Differentiation and Induces Histone Hyperacetylation in Embryonic Stem Cells”. In: *MOLECULAR AND CELLULAR BIOLOGY* 24 (2004).

[40] Hao Wu and Yi Zhang. "Mechanisms and functions of Tet proteinmediated 5-methylcytosine oxidation". In: *GENES & DEVELOPMENT* 25 (2011).

[41] Wenhao Zhang et al. "Isoform Switch of TET1 Regulates DNA Demethylation and Mouse Development". In: *Molecular Cell* 64 (2016).

[42] Dieter Egli, Garrett Birkhoff, and Kevin Eggan. "Mediators of reprogramming: transcription factors and transitions through mitosis". In: *Nature Reviews* 9 (2008).

[43] L Lopez-Serra and M Esteller. "Proteins that bind methylated DNA and human cancer: reading the wrong words". In: *British Journal of Cancer* 98 (2008).

[44] Francois Fuks et al. "The Methyl-CpG-binding Protein MeCP2 Links DNA Methylation to Histone Methylation". In: *The J. of Biological Chemistry* 278 (2003).

[45] FrancË̈ois Fuks et al. "The DNA methyltransferases associate with HP1 and the SUV39H1 histone methyltransferase". In: *Nucleic Acids Research* 31 (2003).

[46] Steen K. T. Ooi et al. "DNMT3L connects unmethylated lysine 4 of histone H3 to de novo methylation of DNA". In: *Nature* 448 (2007).

[47] X. et al. Nan. "Transcriptional repression by the methyl-CpG-binding protein MeCP2 involves a histone deacetylase complex". In: *Nature* 393 (1998).

[48] P.L. et al.. Jones. "Methylated DNA and MeCP2 recruit histone deacetylase to repress transcription". In: *Nature Genetics* 19 (1998).

[49] D. Del Vecchio and R. M. Murray. *Biomolecular Feedback Systems.* Princeton University Press, 2014.

[50] B. K. Shivamoggi. *Perturbation methods for differential equations.* Springer Science+Business Media New York, 2003.

[51] Jerrold E. Marsden. *Elementary CLassical Analysis.* W.H. Freeman and Company, 1974.

[52] R.A. Horn and C.R. Johnson. *Matrix Analysis, 2nd edition.* Cambridge University Press, 2013.

[53] Texier B. "Basic matrix perturbation theory". In: *L'Enseignement Mathématiquel* 64 (2018).

[54] V. Sobolev. *7. Geometry of Singular Perturbations: Critical Cases.* SIAM, 2005, pp. 153–206.

[55] Stephen Wiggins. *Introduction to Applied Nonlinear Dynamical Systems and Chaos.* 2ed., Springer, 2003.

[56] M. P. Mortell E. Shchepakina. V. Sobolev. *Singular Singularly Perturbed Systems.* Springer, 2014.

[57] Sobolev V.A. Kononenco L.I. "Asymptotic decomposition of slow integral manifolds". In: *Siberian Mathematical Journal* 35 (1994).

[58] V.A. Sobolev. "Integral manifolds and decomposition of singularly perturbed systems". In: *Systems & Control Letters* 5 (1984).

[59] Daniel Henry. *Geometric Theory of Semilinear Parabolic Equations.* Springer, Berlin, Heidelberg, 1981.

[60] C. W. Gardiner. *Handbook of stochastic methods for physics, chemistry and the natural sciences.* Springer-Verlag, 1994.

[61] J. R. Norris. *Markov Chains.* Cambridge University Press, 1997.

[62] Daniele Canzio et al. “Chromodomain-mediated oligomerization of HP1 suggests a nucleosome bridging mechanism for heterochromatin assembly”. In: *Mol Cell* 41 (2011).

[63] T Phillips. “The role of methylation in gene expression”. In: *Nature Educatio* 1 (2008).

[64] R. Tirado-Magallanes et al. “Whole genome DNA methylation: beyond genes silencing”. In: *Oncotarget* 8 (2017).

[65] Liling Tang, Eva Nogales, and Claudio Ciferri. “Structure and Function of SWI/SNF Chromatin Remodeling Complexes and Mechanistic Implications for Transcription”. In: *Prog Biophys Mol Biol.* 102 (2010).

[66] G. Felsenfeld and M. Groudine. “Controlling the double helix”. In: *Nature* 421 (2003).

[67] Cooper GM. *The Cell: A Molecular Approach.* Sunderland (MA): Sinauer Associates, 2000.
