# Supplementary material for: Epigenetic cell memory: The gene’s inner chromatin modification circuit
Source: PLoS Comput Biol. 2022 Apr 6;18(4):e1009961. doi: 10.1371/journal.pcbi.1009961 (PMC8985953; doi:10.1371/journal.pcbi.1009961)
Supplement: S2 File — Fig A: effect of system parameters on the deterministic behavior of the positively autoregulated circuit. Figs B-E: effect of system parameters on the stochastic behavior of the positively autoregulated circuit. Fig F: effect of system parameters on the deterministic behavior of the mutual repression circuit. Figs: G-J: effect of system parameters on the stochastic behavior of the mutual repression circuit. Table A: parameter values relative to Fig 5C. Table B: reactions and parameter values used to generate the plots in Figs 5D and B. Table C: reactions and parameter values used to generate the plots in Figs 5E and D. Table D: reactions and parameter values used to generate the plots in Fig 5F. Tables E and F: reactions and parameter values used to generate the plots in Figs 6C and I. Tables G and H: reactions and parameter values used to generate the plots in Figs 6D and J. Table I: reactions and parameter values used to generate the plots in Fig C. Table J: reactions and parameter values used to generate the plots in Fig E. Tables K and L: reactions and parameter values used to generate the plots in Figs G and H. (PDF) [file pcbi.1009961.s002.pdf]

Epigenetic cell memory:
The gene's inner chromatin modification circuit

Simone Bruno[1], Ruth J. Williams[2], and Domitilla Del Vecchio[1]

[1]*Department of Mechanical Engineering, Massachusetts Institute of Technology, 77 Massachusetts Avenue, Cambridge, MA 02139. Emails:* `(sbruno,ddv)@mit.edu`

[2]*Department of Mathematics, University of California, San Diego, 9500 Gilman Drive, La Jolla CA 92093-0112. Email:* `rjwilliams@ucsd.edu`

Supporting information: S2 File

1 Detailed analysis of the positively autoregulated gene model

1.1 ODE model of the positive autoregulation system

By combining the ODEs of the chromatin modification circuit Main Text: Eqs (3) with those of gene expression Main Text: Eq (8) and defining $X := n^X/\mathrm{D_{tot}}$, we obtain the ODE model of the positive autoregulation system:

$$
\begin{aligned}
\dot{\bar{D}}_1^R &= (\bar{u}_1^R + \alpha^{'}(\bar{D}_2^R + \bar{D}_{12}^R))\bar{D} + \mu(b\epsilon + \epsilon^{'}\bar{D}^A)\bar{D}_{12}^R - (u_{20}^R + \alpha(\bar{D}_2^R + \bar{D}_{12}^R) + \bar{\alpha}(\bar{D}_1^R + \bar{D}_{12}^R))\bar{D}_1^R \\
&\quad - \mu^{'}(\beta\epsilon + \epsilon^{'}\bar{D}^A)\bar{D}_1^R \\
\dot{\bar{D}}_2^R &= (\bar{u}_R^2 + \alpha(\bar{D}_2^R + \bar{D}_{12}^R) + \bar{\alpha}(\bar{D}_1^R + \bar{D}_{12}^R))\bar{D} + \mu^{'}(\beta\epsilon + \epsilon^{'}\bar{D}^A)\bar{D}_{12}^R - (u_{10}^R + \alpha^{'}(\bar{D}_2^R + \bar{D}_{12}^R))\bar{D}_2^R \\
&\quad - \mu(b\epsilon + \epsilon^{'}\bar{D}^A)\bar{D}_2^R \qquad (1) \\
\dot{\bar{D}}_{12}^R &= (u_{10}^R + \alpha^{'}(\bar{D}_2^R + \bar{D}_{12}^R))\bar{D}_2^R + (u_{20}^R + \alpha(\bar{D}_2^R + \bar{D}_{12}^R) + \bar{\alpha}(\bar{D}_1^R + \bar{D}_{12}^R))\bar{D}_1^R \\
&\quad - (\mu'(\beta\epsilon + \epsilon^{'}\bar{D}^A) + \mu(b\epsilon + \epsilon^{'}\bar{D}^A))\bar{D}_{12}^R \\
\dot{\bar{D}}^A &= (u_0^A + u^A + \bar{D}^A)\bar{D} - (\epsilon + \epsilon^{'}(\bar{D}_1^R + \bar{D}_{12}^R) + \epsilon^{'}(\bar{D}_2^R + \bar{D}_{12}^R))\bar{D}^A \\
\dot{\bar{X}} &= \bar{\alpha}_x\bar{D}^A - \bar{\gamma}_x\bar{X},
\end{aligned}
$$

in which $\bar{D} = (1 - \bar{D}_1^R - \bar{D}_2^R - \bar{D}_{12}^R - \bar{D}^A)$. Here, we let $u^A = \tilde{u}^A\bar{X}$ with $\tilde{u}^A$ a constant defined in Eq (84) in S1 File.

1.2 Qualitative understanding of the impact of the positive autoregulation

If we multiply both sides of the ODEs in (1) by $D_{tot}(k_M^A D_{tot})$, system (1) can be rewritten in a dimensional way:

$$\begin{aligned}
\dot{D}_1^R &= (k_{W0}^1 + k_W^1 + k_M^{'}(D_2^R + D_{12}^R))D + (\delta + \bar{k}_E^R + k_E^R D^A)D_{12}^R \\
&\quad - (k_{W0}^2 + k_M(D_2^R + D_{12}^R) + \bar{k}_M(D_1^R + D_{12}^R) + \delta^{'} + k_T^{'} + k_T^{'*} D^A)D_1^R \\
\dot{D}_2^R &= (k_{W0}^2 + k_W^2 + k_M(D_2^R + D_{12}^R) + \bar{k}_M(D_1^R + D_{12}^R))D + (\delta^{'} + k_T^{'} + k_T^{'*} D^A)D_{12}^R \\
&\quad - (k_{W0}^1 + k_M^{'}(D_2^R + D_{12}^R) + \delta + \bar{k}_E^R + k_E^R D^A)D_2^R \\
\dot{D}_{12}^R &= (k_{W0}^1 + k_M^{'}(D_2^R + D_{12}^R))D_2^R + (k_{W0}^2 + k_M(D_2^R + D_{12}^R) + \bar{k}_M(D_1^R + D_{12}^R))D_1^R \\
&\quad - (\delta^{'} + k_T^{'} + k_T^{'*} D^A + \delta + \bar{k}_E^R + k_E^R D^A)D_{12}^R \\
\dot{D}^A &= (k_{W0}^A + k_W^A + k_M^A D^A)D - (\delta + \bar{k}_E^A + k_E^A(D_2^R + D_{12}^R) + k_E^A(D_1^R + D_{12}^R))D^A \\
\dot{X} &= \alpha_x D^A - \gamma_x X,
\end{aligned} \tag{2}$$

in which all the parameters are defined as done for Eqs (79) in S1 File. In particular, $k_W^A = \tilde{k}_W^A X$. Now, in order to gain a qualitative understanding of the impact of self-activation on the stability of the system, let us approximate first the reactions involving X are fast and then we can set the protein dynamics to the QSS (i.e, $X = p_x D^A$ with $p_x = \alpha_x/\gamma_x$),the ODEs (2) can be re-written as follows:

$$\begin{aligned}
\dot{D}_1^R &= (k_{W0}^1 + k_W^1 + k_M^{'}(D_2^R + D_{12}^R))D + (\delta + \bar{k}_E^R + k_E^R D^A)D_{12}^R \\
&\quad - (k_{W0}^2 + k_M(D_2^R + D_{12}^R) + \bar{k}_M(D_1^R + D_{12}^R) + \delta^{'} + k_T^{'} + k_T^{'*} D^A)D_1^R \\
\dot{D}_2^R &= (k_{W0}^2 + k_W^2 + k_M(D_2^R + D_{12}^R) + \bar{k}_M(D_1^R + D_{12}^R))D + (\delta^{'} + k_T^{'} + k_T^{'*} D^A)D_{12}^R \\
&\quad - (k_{W0}^1 + k_M^{'}(D_2^R + D_{12}^R) + \delta + \bar{k}_E^R + k_E^R D^A)D_2^R \\
\dot{D}_{12}^R &= (k_{W0}^1 + k_M^{'}(D_2^R + D_{12}^R))D_2^R + (k_{W0}^2 + k_M(D_2^R + D_{12}^R) + \bar{k}_M(D_1^R + D_{12}^R))D_1^R \\
&\quad - (\delta^{'} + k_T^{'} + k_T^{'*} D^A + \delta + \bar{k}_E^R + k_E^R D^A)D_{12}^R \\
\dot{D}^A &= (k_{W0}^A + (\tilde{k}_W^A + k_M^A)D^A)D - (\delta + \bar{k}_E^A + k_E^A(D_2^R + D_{12}^R) + k_E^A(D_1^R + D_{12}^R))D^A.
\end{aligned} \tag{3}$$

Comparing these ODEs with the ones related to the chromatin modification circuit alone in Eqs (79) in S1 File, it is possible to see that the introduction of the positive autoregulation leads to an increase of the auto-catalysis rate constant of the activating chromatin marks (that is, in the last ODE, before it was k_M^A, while now it is $(\tilde{k}_W^A + k_M^A)$). Based on the bifurcation plots realized for the chromatin modification circuit (Fig K in S1 File), we know that a higher k_M^A can restore the stability of the active state.

1.3 Derivation of the stationary probability distribution formula for the positively autoregulated gene

Following the same procedure used to obtain the one-dimensional reduced model for the chromatin dynamics circuit S1 File: reactions (158) (Section 3.2 in S1 File), we obtain the following chemical

reaction system:

$$\mathrm{D^A} \xrightarrow{k_{AR}} \mathrm{D^R_{12}}, \quad k_{AR} = \left(\frac{(\delta + \bar{k}^A_E + 2k^A_E D^R_{12})(k^2_{W0} + k^2_W + k^1_{W0} + k^1_W + (k_M + \bar{k}_M + k^{'}_M)D^R_{12})}{k^A_{W0} + k^A_W + k^A_M D^A + k^2_{W0} + k^2_W + k^1_{W0} + k^1_W + (k_M + \bar{k}_M + k^{'}_M)D^R_{12}} \right);$$

$$\mathrm{D^R_{12}} \xrightarrow{k_{RA}} \mathrm{D^A}, \quad k_{RA} = \left(\frac{(\delta + \bar{k}^R_E + k^R_E D^A)(\delta' + k^{'}_T + k^{'*}_T D^A)\bar{K}_{dim}(k^A_{W0} + k^A_W + k^A_M D^A)}{k^A_{W0} + k^A_W + k^A_M D^A + k^2_{W0} + k^2_W + k^1_{W0} + k^1_W + (k_M + \bar{k}_M + k^{'}_M)D^R_{12}} \right). \tag{4}$$

with $\bar{K}_{dim} = \frac{1}{k^1_{W0} + k^{'}_M D^R_{12}} + \frac{1}{k^2_{W0} + (k_M + \bar{k}_M)D^R_{12}}$ and $k^A_W = \tilde{k}^A_W p_x D^A$. Now, in order to derive the formula for the stationary distribution, we introduce $x = n^R_{12}$. Then, since the reactions (4) has the same form of the ones related to the chromatin modification system (S1 File: reactions (158)), in which $k^A_W = \tilde{k}^A_W p_x D^A$, we can use the same formula for $\pi_{\epsilon \ll 1}(x)$ given by Eq (169) in S1 File, in which we substitute u^A with $u^A = \frac{\tilde{k}^A_W}{k^A_M} p_x \frac{(\mathrm{D_{tot}} - x)}{\mathrm{D_{tot}}} = \tilde{u}^A p_x \frac{(\mathrm{D_{tot}} - x)}{\mathrm{D_{tot}}}$. Then, the stationary probability distribution under the condition $\epsilon \ll 1$ can be written as follows:

$$\pi_{\epsilon \ll 1}(x) \approx \begin{cases} \frac{1}{1+P} & \text{if } x = 0 \\ 0 & \text{if } x \neq 0, \mathrm{D_{tot}} \\ \frac{P}{1+P} & \text{if } x = \mathrm{D_{tot}} \end{cases} \tag{5}$$

with

$$P = \frac{(u_{tot} + \alpha + \bar{\alpha} + \alpha^{'})}{(u_{tot} + \tilde{u}^A p_x + 1)} \cdot \prod_{i=1}^{\mathrm{D_{tot}}-1} \left(\frac{2(\bar{u}^R_{12} + (\alpha + \bar{\alpha} + \alpha^{'})\frac{i}{\mathrm{D_{tot}}})}{\mu\mu'\epsilon' \frac{(\mathrm{D_{tot}}-i)}{\mathrm{D_{tot}}} \bar{K}_i (u^A_0 + (\tilde{u}^A p_x + 1)\frac{(\mathrm{D_{tot}}-i)}{\mathrm{D_{tot}}})} \right) \cdot \frac{(\bar{u}^R_{12})}{\mu\mu' b\beta\epsilon \bar{K}_{\mathrm{D_{tot}}}(u^A_0)}, \tag{6}$$

in which $x = n^R$, $u_{tot} = u^A_0 + \bar{u}^R_{12}$, $\bar{u}^R_{12} = u^R_1 + u^R_{10} + u^R_2 + u^R_{20}$ and $\bar{K}_i$ defined in Eq (161) in S1 File.

1.4 Derivation of time to memory loss formula for the positively autoregulated gene

Now, let us derive the formula for the time to memory loss of the active gene state, $\tau_0^{\mathrm{D_{tot}}}$. In particular, for the Markov chain related to the S1 File: reactions (158) we obtain the formula Eq (175) in S1 File for $\tau_0^{\mathrm{D_{tot}}}$. Now, since our current reactions (4) differ from those in S1 File: reactions (158) by $k^A_W = \tilde{k}^A_W p_x (D_{tot} - D^R_{12})$, we can directly use the formula for $\tau_0^{\mathrm{D_{tot}}}$ previously computed in Eq (175) in S1 File and substitute $u^A = \frac{k^A_W}{k^A_M D_{tot}}$ with $u^A = \frac{\tilde{k}^A_W}{k^A_M} p_x \frac{(D_{tot} - D^R_{12})}{D_{tot}} = \tilde{u}^A p_x \frac{(D_{tot} - D^R_{12})}{D_{tot}}$. We then obtain that $\tau_0^{\mathrm{D_{tot}}}$ can be defined as follows:

$$\tau_0^{\mathrm{D_{tot}}} = \frac{\tilde{r}_{\mathrm{D_{tot}}-1}}{\alpha_0} \left(1 + \sum_{j=1}^{\mathrm{D_{tot}}-1} \frac{1}{\tilde{r}_i} \right) + \frac{1}{\alpha_{\mathrm{D_{tot}}-1}} + \sum_{i=2}^{\mathrm{D_{tot}}-1} \left[\frac{\tilde{r}_{i-1}}{\alpha_{\mathrm{D_{tot}}-i}} \left(1 + \sum_{j=1}^{i-1} \frac{1}{\tilde{r}_j} \right) \right] \tag{7}$$

in which α_i and γ_i are defined as

$$\alpha_i = \left(\frac{(\epsilon + 2\epsilon' \frac{i}{\mathrm{D_{tot}}})(k^2_W + k^2_{W0} + k^1_W + k^1_{W0} + \frac{(k_M + \bar{k}_M + k^{'}_M)}{\Omega} i)}{u^A_0 + (\tilde{u}^A p_x + 1)\frac{(\mathrm{D_{tot}}-i)}{\mathrm{D_{tot}}} + u^R_2 + u^R_{20} + u^R_1 + u^R_{10} + (\alpha + \bar{\alpha} + \alpha')\frac{i}{\mathrm{D_{tot}}}} \right) (\mathrm{D_{tot}} - i)$$

$$\gamma_i = \left(\frac{\mu(b\epsilon + \epsilon' \frac{(\mathrm{D_{tot}}-i)}{\mathrm{D_{tot}}})\mu'(\beta\epsilon + \epsilon' \frac{(\mathrm{D_{tot}}-i)}{\mathrm{D_{tot}}})\bar{K}_i(k^A_{W0} + (\frac{\tilde{k}^A_W}{\Omega} p_x + \frac{k^A_M}{\Omega})(\mathrm{D_{tot}} - i))}{u^A_0 + (\tilde{u}^A p_x + 1)\frac{(\mathrm{D_{tot}}-i)}{\mathrm{D_{tot}}} + u^R_2 + u^R_{20} + u^R_1 + u^R_{10} + (\alpha + \bar{\alpha} + \alpha')\frac{i}{\mathrm{D_{tot}}}} \right) i, \tag{8}$$

and $\tilde{r}_j = \frac{\gamma_{\mathrm{D_{tot}}-1}\gamma_{\mathrm{D_{tot}}-2}\cdots\gamma_{\mathrm{D_{tot}}-j}}{\alpha_{\mathrm{D_{tot}}-1}\alpha_{\mathrm{D_{tot}}-2}\cdots\alpha_{\mathrm{D_{tot}}-j}}$. Then, assuming that $\epsilon^{'} \neq 0$, it is possible to notice that, for $\epsilon \ll 1$, the dominant term of $\tau_0^{\mathrm{D_{tot}}}$ is the first addend in (7).

Then, by normalizing the time to memory loss with respect $\frac{k_M^A \mathrm{D_{tot}}}{\Omega}$ ($\bar{\tau}_0^{\mathrm{D_{tot}}} = \tau_0^{\mathrm{D_{tot}}} \frac{k_M^A \mathrm{D_{tot}}}{\Omega}$), $\tau_0^{\mathrm{D_{tot}}}$ in the regime $\epsilon \ll 1$ can be re-written as follows:

$$\bar{\tau}_0^{\mathrm{D_{tot}}} = \bar{\tau}_A \approx \frac{f_2(p_x)}{\epsilon}\left(1 + \sum_{i=1}^{\mathrm{D_{tot}}-1} \frac{h_2^i(p_x, \mu\mu^{'})}{K_A^i}\right), \tag{9}$$

in which f_2 and h_2^i are increasing functions of their arguments, $h_2^i(p_x, 0) = 0$, K_A^i are functions independent of ϵ, $\mu^{'}$, μ and p_x, and in which we redefine $\bar{\tau}_0^{\mathrm{D_{tot}}}$ as $\bar{\tau}_A$ to simplify the notation.

2 Detailed analysis of the mutual repression circuit model

2.1 Expressions of the $k_W^{1,\ell}$, $k_W^{2,\ell}$ and $k_W^{A,\ell}$ with $\ell = X, Z$

Based on the formula of k_W^A, k_W^1 and k_W^2 given in Eqs (82) and (83) in S1 File, $k_W^{1,\ell}$, $k_W^{2,\ell}$ and $k_W^{A,\ell}$ with $\ell = X, Z$ can be written as follows:

$$\begin{aligned} k_W^{A,X} &= \frac{\tilde{k}_W^A}{\Omega}\frac{n^X}{1+\frac{n^X}{\Omega K}+\frac{n^Z}{\Omega K}}; \; k_W^{1,X} = \frac{\tilde{k}_W^1}{\Omega}\frac{n^Z}{1+\frac{n^X}{\Omega K}+\frac{n^Z}{\Omega K}}; \; k_W^{2,X} = \frac{\tilde{k}_W^2}{\Omega}\frac{n^Z}{1+\frac{n^X}{\Omega K}+\frac{n^Z}{\Omega K}} \\ k_W^{A,Z} &= \frac{\tilde{k}_W^A}{\Omega}\frac{n^Z}{1+\frac{n^X}{\Omega K}+\frac{n^Z}{\Omega K}}; \; k_W^{1,Z} = \frac{\tilde{k}_W^1}{\Omega}\frac{n^X}{1+\frac{n^X}{\Omega K}+\frac{n^Z}{\Omega K}}; \; k_W^{2,Z} = \frac{\tilde{k}_W^2}{\Omega}\frac{n^X}{1+\frac{n^X}{\Omega K}+\frac{n^Z}{\Omega K}}. \end{aligned} \tag{10}$$

in which we set

$$K_{XX} = K_{ZZ} = K. \tag{11}$$

Now, let us define $u^{A,\ell}$, $u_1^{R,\ell}$ and $u_2^{R,\ell}$ as

$$u^{A,\ell} = k_W^{A,\ell}/(k_M^A D_{tot}); \quad u_1^{R,\ell} = k_W^{1,\ell}/(k_M^A D_{tot}); \quad u_2^{R,\ell} = k_W^{2,\ell}/(k_M^A D_{tot}), \tag{12}$$

with $\ell = X, Z$. Then, if we assume $n^X/\Omega \ll K$ and $n^Z/\Omega \ll K$, $u^{A,\ell}$, $u_1^{R,\ell}$ and $u_2^{R,\ell}$ can be written as

$$\begin{aligned} u_A^X &= \frac{\tilde{k}_W^A n^X}{\Omega k_M^A D_{tot}} = \frac{\tilde{k}_W^A n^X}{k_M^A \mathrm{D_{tot}}} = \frac{\tilde{k}_W^A}{k_M^A}\bar{X} = \tilde{u}^A \bar{X}; \; u_1^{R,X} = \frac{\tilde{k}_W^1 n^Z}{\Omega k_M^A D_{tot}} = \frac{\tilde{k}_W^1 n^Z}{k_M^A \mathrm{D_{tot}}} = \frac{\tilde{k}_W^1}{k_M^A}\bar{Z} = \tilde{u}_1^R \bar{Z}; \\ u_2^{R,X} &= \frac{\tilde{k}_W^2 n^Z}{\Omega k_M^A D_{tot}} = \frac{\tilde{k}_W^2 n^Z}{k_M^A \mathrm{D_{tot}}} = \frac{\tilde{k}_W^2}{k_M^A}\bar{Z} = \tilde{u}_2^R \bar{Z}; \; u_A^Z = \frac{\tilde{k}_W^A n^Z}{\Omega k_M^A D_{tot}} = \frac{\tilde{k}_W^A n^Z}{k_M^A \mathrm{D_{tot}}} = \frac{\tilde{k}_W^A}{k_M^A}\bar{Z} = \tilde{u}^A \bar{Z}; \\ u_1^{R,Z} &= \frac{\tilde{k}_W^1 n^X}{\Omega k_M^A D_{tot}} = \frac{\tilde{k}_W^1 n^X}{k_M^A \mathrm{D_{tot}}} = \frac{\tilde{k}_W^1}{k_M^A}\bar{X} = \tilde{u}_1^R \bar{X}; \; u_2^{R,Z} = \frac{\tilde{k}_W^2 n^X}{\Omega k_M^A D_{tot}} = \frac{\tilde{k}_W^2 n^X}{k_M^A \mathrm{D_{tot}}} = \frac{\tilde{k}_W^2}{k_M^A}\bar{X} = \tilde{u}_2^R \bar{X}, \end{aligned} \tag{13}$$

in which we define $X := n^X/\mathrm{D_{tot}}$, $Z := n^Z/\mathrm{D_{tot}}$, $\tilde{u}^A = \tilde{k}_W^A/k_M^A$, $\tilde{u}_1^R = \tilde{k}_W^1/k_M^A$ and $\tilde{u}_2^R = \tilde{k}_W^2/k_M^A$.

2.2 ODE model of the mutual repression system

The ODEs of the system are obtained by combining the ODEs of the chromatin modification circuit and those of the gene expression circuit (Main Text: Eqs (3) and (8), respectively) for each gene and by properly setting the inputs according to Eqs (13). In particular, we assume equal

parameters for the two chromatin modification circuits, we let X and Z denote the genes' products, and indicate the species within each of the corresponding chromatin modification circuits by "X" and "Z" superscripts, respectively. Furthermore, we define $X := n^X/\mathrm{D_{tot}}$, $Z := n^Z/\mathrm{D_{tot}}$. Thus, the ODE model in terms of non-dimensional variables and non-dimensional parameters can be written as follows:

$$
\begin{aligned}
\dot{\bar{D}}_1^{R,X} &= (u_{10}^R + u_1^{R,X} + \alpha'(\bar{D}_2^{R,X} + \bar{D}_{12}^{R,X}))\bar{D}^X + \mu(b\epsilon + \epsilon'\bar{D}^{A,X})D_{12}^{R,X} \\
&\quad - (u_{20}^R + \alpha(\bar{D}_2^{R,X} + \bar{D}_{12}^{R,X}) + \bar{\alpha}(\bar{D}_1^{R,X} + \bar{D}_{12}^{R,X}) + \mu'(\beta\epsilon + \epsilon'\bar{D}^{A,X}))\bar{D}_1^{R,X} \\
\dot{\bar{D}}_2^{R,X} &= (u_{20}^R + u_2^{R,Z} + \alpha(\bar{D}_2^{R,X} + \bar{D}_{12}^{R,X}) + \bar{\alpha}(\bar{D}_1^{R,X} + \bar{D}_{12}^{R,X}))\bar{D}^X + \mu'(\beta\epsilon + \epsilon'\bar{D}^{A,X})\bar{D}_{12}^{R,X} \\
&\quad - (u_{10}^R + \alpha'(\bar{D}_2^{R,X} + \bar{D}_{12}^{R,X}) + \mu(b\epsilon + \epsilon'\bar{D}^{A,X}))\bar{D}_2^{R,X} \\
\dot{\bar{D}}_{12}^{R,X} &= (u_{10}^R + \alpha'(\bar{D}_2^{R,X} + \bar{D}_{12}^{R,X}))\bar{D}_2^{R,X} + (u_{20}^R + \alpha(\bar{D}_2^{R,X} + \bar{D}_{12}^{R,X}) + \bar{\alpha}(\bar{D}_1^{R,X} + \bar{D}_{12}^{R,X}))\bar{D}_1^{R,X} \\
&\quad - (\mu(b\epsilon + \epsilon'\bar{D}^{A,X}) + \mu'(\beta\epsilon + \epsilon'\bar{D}^{A,X}))\bar{D}_{12}^{R,X} \\
\dot{\bar{D}}^{A,X} &= (u_0^A + u_A^X + \bar{D}^{A,X})\bar{D}^X - (\epsilon + \epsilon'(\bar{D}_2^{R,X} + \bar{D}_{12}^{R,X}) + \epsilon'(\bar{D}_1^{R,X} + \bar{D}_{12}^{R,X}))\bar{D}^{A,X} \\
\dot{\bar{X}} &= \bar{\alpha}_x\bar{D}^{A,X} - \bar{\gamma}_x\bar{X} \\
\dot{\bar{D}}_1^{R,Z} &= (u_{10}^R + u_1^{R,Z} + \alpha'(\bar{D}_2^{R,Z} + \bar{D}_{12}^{R,Z}))\bar{D}^Z + \mu(b\epsilon + \epsilon'\bar{D}^{A,Z})D_{12}^{R,Z} \\
&\quad - (u_{20}^R + \alpha(\bar{D}_2^{R,Z} + \bar{D}_{12}^{R,Z}) + \bar{\alpha}(\bar{D}_1^{R,Z} + \bar{D}_{12}^{R,Z}) + \mu'(\beta\epsilon + \epsilon'\bar{D}^{A,Z}))\bar{D}_1^{R,Z} \\
\dot{\bar{D}}_2^{R,Z} &= (u_{20}^R + u_2^{R,Z} + \alpha(\bar{D}_2^{R,Z} + \bar{D}_{12}^{R,Z}) + \bar{\alpha}(\bar{D}_1^{R,Z} + \bar{D}_{12}^{R,Z}))\bar{D}^Z + \mu'(\beta\epsilon + \epsilon'\bar{D}^{A,Z})\bar{D}_{12}^{R,Z} \\
&\quad - (u_{10}^R + \alpha'(\bar{D}_2^{R,Z} + \bar{D}_{12}^{R,Z}) + \mu(b\epsilon + \epsilon'\bar{D}^{A,Z}))\bar{D}_2^{R,Z} \\
\dot{\bar{D}}_{12}^{R,Z} &= (u_{10}^R + \alpha'(\bar{D}_2^{R,Z} + \bar{D}_{12}^{R,Z}))\bar{D}_2^{R,Z} + (u_{20}^R + \alpha(\bar{D}_2^{R,Z} + \bar{D}_{12}^{R,Z}) + \bar{\alpha}(\bar{D}_1^{R,Z} + \bar{D}_{12}^{R,Z}))\bar{D}_1^{R,Z} \\
&\quad - (\mu(b\epsilon + \epsilon'\bar{D}^{A,Z}) + \mu'(\beta\epsilon + \epsilon'\bar{D}^{A,Z}))\bar{D}_{12}^{R,Z} \\
\dot{\bar{D}}^{A,Z} &= (u_0^A + u_Z^A + \bar{D}^{A,Z})\bar{D}^Z - (\epsilon + \epsilon'(\bar{D}_2^{R,Z} + \bar{D}_{12}^{R,Z}) + \epsilon'(\bar{D}_1^{R,Z} + \bar{D}_{12}^{R,Z}))\bar{D}^{A,Z} \\
\dot{\bar{Z}} &= \bar{\alpha}_z\bar{D}^{A,Z} - \bar{\gamma}_z\bar{Z},
\end{aligned}
\tag{14}
$$

in which $\bar{D}^X = (1 - \bar{D}_1^{R,X} - \bar{D}_2^{R,X} - \bar{D}_{12}^{R,X} - \bar{D}^{A,X})$ and $\bar{D}^Z = (1 - \bar{D}_1^{R,Z} - \bar{D}_2^{R,Z} - \bar{D}_{12}^{R,Z} - \bar{D}^{A,Z})$. Based on the expression for $k_W^{1,\ell}$, $k_W^{2,\ell}$ and $k_W^{A,\ell}$ given in Eqs (10), we approximate $u^{A,\ell} = \tilde{u}^A\ell$ and, for $i \in \{1,2\}$, $u_i^{R,\ell} = \tilde{u}_i^R j$, with $\ell, j = X, Z$ and $\ell \neq j$ (Section (2.1), Eqs (11),(13)).

2.3 Deterministic analysis

For this deterministic analysis, we exploit the results obtained for the positive autoregulation circuit viewed as an input/output system. In fact, the block diagram in Fig 6B makes it explicit that the mutual repression circuit is the input/output composition of two positively autoregulated genes, in which the output of one gene, n^X or n^Z, is used as an input to the other gene by increasing $k_W^{R,Z}$ or $k_W^{R,X}$, respectively. Defining $p_x = \bar{\alpha}_x/\bar{\gamma}_x$ and $p_z = \bar{\alpha}_z/\bar{\gamma}_z$, at steady state $\bar{X} = p_x\bar{D}^{A,X}$ and $\bar{Z} = p_z\bar{D}^{A,Z}$. Then for a state of system (14) to be a steady state, the $(\bar{D}^{A,X}, \bar{D}^{A,Z})$ pair must lie on the intersection of the input/output steady state characteristics of the X gene and of the Z gene as shown in Fig F. In particular, we assume $\tilde{u}_1^R = \tilde{u}_2^R = \tilde{u}^R$ and we consider, for the X gene, the $(u^{R,X}, \bar{D}^{A,X})$ input/output steady state characteristic, with $u^{R,X} = u_1^{R,X} = u_2^{R,X} = \tilde{u}^R\bar{Z}$, that, at steady state, can be written as $u^{R,X} = \tilde{u}^R p_z\bar{D}^{A,Z}$. Similarly, for the Z gene, we consider the $(u^{R,Z}, \bar{D}^{A,Z})$ input/output steady state characteristic, with $u^{R,Z} = u_1^{R,Z} = u_2^{R,Z} = \tilde{u}^R\bar{X}$, that, at steady state, can be written as $u^{R,Z} = \tilde{u}^R p_x\bar{D}^{A,X}$. It is also possible to show that for each of the intersections shown in Fig F, there is a unique combination of positive values for the remaining

variables such that the system is at steady state. We determined stability of these steady states numerically by evaluating the eigenvalues of the Jacobian of the system.

In summary, for low p values, the system has a unique stable steady state about the origin. By increasing p, the system acquires two stable steady states, in which one gene is "on" and the other is "off". That is, one equilibrium with $\bar{D}^{A,X} \gg \bar{D}^{A,Z}$ and the other equilibrium with $\bar{D}^{A,X} \ll \bar{D}^{A,Z}$. When ϵ is large, also the steady state about the origin is stable, while when ϵ is small, it is not. Thus, when p is large (high expression rate) and ϵ is small (small basal erasure rate), the system is tri-stable. When μ' is increased, the system can have four co-existing stable steady states (Fig F).

2.4 Analytical analysis

For this analysis, as we did for the previous system analyzed, we consider the parameter regime $\epsilon^{'} \ll 1$ and we assume that the reactions involving X and Z are fast compared to the other reactions (that is, considering $\epsilon = c\epsilon^{'}$, let us assume $\bar{\gamma}_x, \bar{\gamma}_z \gg \epsilon^{'}, \epsilon^{'}\mu, \epsilon^{'}\mu^{'}$) so that we can set the protein dynamics to the QSS ($X = p_x D^{A,X}$ and $Z = p_z D^{A,Z}$). Then, by considering the one-dimensional approximation of the chromatin dynamics circuit S1 File: reactions (158), we obtain the following chemical reaction system:

$$
\begin{aligned}
&\mathrm{D^{A,X}} \xrightarrow{k^X_{AR}} \mathrm{D^{R,X}_{12}}, \qquad && \mathrm{D^{R,X}_{12}} \xrightarrow{k^X_{RA}} \mathrm{D^{A,X}}, \\
&\mathrm{D^{A,Z}} \xrightarrow{k^Z_{AR}} \mathrm{D^{R,Z}_{12}}, \qquad && \mathrm{D^{R,Z}_{12}} \xrightarrow{k^Z_{RA}} \mathrm{D^{A,Z}},
\end{aligned}
\tag{15}
$$

with

$$
\begin{aligned}
k^X_{AR} &= \left(\frac{(\delta + \bar{k}^A_E + 2k^A_E D^{R,X}_{12})(k^2_{W0} + k^1_{W0} + k^{1,X}_W + k^{2,X}_W + (k_M + \bar{k}_M + k^{'}_M)D^{R,X}_{12})}{k^A_{W0} + k^{A,X}_W + k^A_M D^{A,X} + k^2_{W0} + k^1_{W0} + k^{1,X}_W + k^{2,X}_W + (k_M + \bar{k}_M + k^{'}_M)D^{R,X}_{12}} \right), \\
k^X_{RA} &= \left(\frac{(\delta + \bar{k}^R_E + k^R_E D^{A,X})(\delta^{'} + k^{'}_T + k^{'*}_T D^{A,X})\bar{K}^X_{dim}(k^A_{W0} + k^{A,X}_W + k^A_M D^{A,X})}{k^A_{W0} + k^{A,X}_W + k^A_M D^{A,X} + k^2_{W0} + k^1_{W0} + k^{1,X}_W + k^{2,X}_W + (k_M + \bar{k}_M + k^{'}_M)D^{R,X}_{12}} \right), \\
k^Z_{AR} &= \left(\frac{(\delta + \bar{k}^A_E + 2k^A_E D^{R,Z}_{12})(k^2_{W0} + k^1_{W0} + k^{1,Z}_W + k^{2,Z}_W + (k_M + \bar{k}_M + k^{'}_M)D^{R,Z}_{12})}{k^A_{W0} + k^{A,Z}_W + k^A_M D^{A,Z} + k^2_{W0} + k^1_{W0} + k^{1,Z}_W + k^{2,Z}_W + (k_M + \bar{k}_M + k^{'}_M)D^{R,Z}_{12}} \right), \\
k^Z_{RA} &= \left(\frac{(\delta + \bar{k}^R_E + k^R_E D^{A,Z})(\delta^{'} + k^{'}_T + k^{'*}_T D^{A,Z})\bar{K}^Z_{dim}(k^A_{W0} + k^{A,Z}_W + k^A_M D^{A,Z})}{k^A_{W0} + k^{A,Z}_W + k^A_M D^{A,Z} + k^2_{W0} + k^1_{W0} + k^{1,Z}_W + k^{2,Z}_W + (k_M + \bar{k}_M + k^{'}_M)D^{R,Z}_{12}} \right),
\end{aligned}
\tag{16}
$$

with $\bar{K}^i_{dim} = \frac{1}{k^1_{W0} + k^{'}_M D^{R,i}_{12}} + \frac{1}{k^2_{W0} + (k_M + \bar{k}_M)D^{R,i}_{12}}$, $k^{A,i}_W = \tilde{k}^{A,i}_W p_i(D_{tot} - D^{R,i}_{12})$, $k^A_W = \tilde{k}^A_W p_x(D_{tot} - D^{R,i}_{12})$, $k^{1,i}_W = \tilde{k}^1_W p_j(D_{tot} - D^{R,j}_{12})$, $k^{2,i}_W = \tilde{k}^2_W p_j(D_{tot} - D^{R,j}_{12})$ for $i, j = X, Z$ and $i \neq j$. The reduced system (16) can be represented by a two-dimensional Markov chain in which the state is defined as $s = (v, w)$, with $v = n^{R,X}_{12}$ and $w = n^{R,Z}_{12}$. In particular, v and w can vary between zero and $\mathrm{D_{tot}}$. Furthermore, assuming $p_x = p_z$ (i.e.,the production and degradation rate constants are the same for protein X and Z) and defining $p = p_x = p_z$, $\tilde{k}^R_W = \tilde{k}^1_W + \tilde{k}^2_W$, $\tilde{u}^A = \frac{\tilde{k}^A_W}{k^A_M}$ and $\tilde{u}^R = \frac{\tilde{k}^R_W}{k^A_M}$, the

transition rates between the states can be written as follows:

$$
\begin{aligned}
q_{(v,w),(v+1,w)} &= \left(\frac{(\epsilon + 2\epsilon^{'} \frac{v}{\mathrm{D_{tot}}})(k^2_{W0} + k^1_{W0} + \frac{\tilde{k}^R_W}{\Omega} p(\mathrm{D_{tot}} - w) + \frac{(k_M + \bar{k}_M + k^{'}_M)}{\Omega} v)}{u^A_0 + (\tilde{u}^A p + 1)\frac{(\mathrm{D_{tot}} - v)}{\mathrm{D_{tot}}} + u^R_{20} + u^R_{10} + \tilde{u}^R p \frac{(\mathrm{D_{tot}} - w)}{\mathrm{D_{tot}}} + (\alpha + \bar{\alpha} + \alpha^{\prime})\frac{v}{\mathrm{D_{tot}}}} \right)(\mathrm{D_{tot}} - v) \\
q_{(v,w),(v-1,w)} &= \left(\frac{\mu(b\epsilon + \epsilon^{'} \frac{(\mathrm{D_{tot}} - v)}{\mathrm{D_{tot}}})\mu^{'}(\beta\epsilon + \epsilon^{'} \frac{(\mathrm{D_{tot}} - v)}{\mathrm{D_{tot}}})\bar{K}^v (k^A_{W0} + (\frac{\tilde{k}^A_W}{\Omega} p + \frac{k^A_M}{\Omega})(\mathrm{D_{tot}} - v))}{u^A_0 + (\tilde{u}^A p + 1)\frac{(\mathrm{D_{tot}} - v)}{\mathrm{D_{tot}}} + u^R_{20} + u^R_{10} + \tilde{u}^R p \frac{(\mathrm{D_{tot}} - w)}{\mathrm{D_{tot}}} + (\alpha + \bar{\alpha} + \alpha^{\prime})\frac{v}{\mathrm{D_{tot}}}} \right) v \\
q_{(v,w),(v,w+1)} &= \left(\frac{(\epsilon + 2\epsilon^{'} \frac{w}{\mathrm{D_{tot}}})(k^2_{W0} + k^1_{W0} + \frac{\tilde{k}^R_W}{\Omega} p(\mathrm{D_{tot}} - v) + \frac{(k_M + \bar{k}_M + k^{'}_M)}{\Omega} w)}{u^A_0 + (\tilde{u}^A p + 1)\frac{(\mathrm{D_{tot}} - w)}{\mathrm{D_{tot}}} + u^R_{20} + u^R_{10} + \tilde{u}^R p \frac{(\mathrm{D_{tot}} - v)}{\mathrm{D_{tot}}} + (\alpha + \bar{\alpha} + \alpha^{\prime})\frac{w}{\mathrm{D_{tot}}}} \right)(\mathrm{D_{tot}} - w) \\
q_{(v,w),(v,w-1)} &= \left(\frac{\mu(b\epsilon + \epsilon^{'} \frac{(\mathrm{D_{tot}} - w)}{\mathrm{D_{tot}}})\mu^{'}(\beta\epsilon + \epsilon^{'} \frac{(\mathrm{D_{tot}} - w)}{\mathrm{D_{tot}}})\bar{K}^w (k^A_{W0} + (\frac{\tilde{k}^A_W}{\Omega} p + \frac{k^A_M}{\Omega})(\mathrm{D_{tot}} - w))}{u^A_0 + (\tilde{u}^A p + 1)\frac{(\mathrm{D_{tot}} - w)}{\mathrm{D_{tot}}} + u^R_{20} + u^R_{10} + \tilde{u}^R p \frac{(\mathrm{D_{tot}} - v)}{\mathrm{D_{tot}}} + (\alpha + \bar{\alpha} + \alpha^{\prime})\frac{w}{\mathrm{D_{tot}}}} \right) w
\end{aligned}
\tag{17}
$$

in which $\bar{K}^i = \frac{1}{u^R_{10} + \alpha^{\prime} i} + \frac{1}{u^R_{20} + (\alpha + \bar{\alpha}) i}$ for $i = v, w$, and the rate of leaving the state (v, w) as

$$\bar{q}_{(v,w)} = q_{(v,w),(v+1,w)} + q_{(v,w),(v-1,w)} + q_{(v,w),(v,w+1)} + q_{(v,w),(v,w-1)}. \tag{18}$$

The total number of states characterizing the Markov chain is $(\mathrm{D_{tot}} + 1)^2$. Furthermore, let us introduce the infinitesimal generator of the Markov chain Q, that is a matrix whose $(r, l)_{th}$ entry, for $1 \leq r \neq l \leq (\mathrm{D_{tot}} + 1)^2$ is the transition rate of going to the state l, starting from the state r and the $(r, r)_{th}$ entry is the opposite of the rate of leaving the state r [1]. Then, introducing $\pi = [\pi(0, 0), \pi(0, 1), \pi(0, 2), ..., \pi(\mathrm{D_{tot}}, \mathrm{D_{tot}} - 1), \pi(\mathrm{D_{tot}}, \mathrm{D_{tot}})]$, the stationary distribution can be evaluated by solving the system of equations given by

$$\pi Q = 0. \tag{19}$$

For example, the generic r^{th} equation of the system (19) associated with the state $r = (v, w)$ is written as follows:

$$
\begin{aligned}
\bar{q}_{(v,w)}\pi(v, w) &= q_{(v,w),(v+1,w)}\pi(v - 1, w) + q_{(v,w),(v-1,w)}\pi(v + 1, w) \\
&\quad + q_{(v,w),(v,w+1)}\pi(v, w - 1) + q_{(v,w),(v,w-1)}\pi(v, w + 1).
\end{aligned}
\tag{20}
$$

Now, let us write explicitly the $\bar{q}_{(v,w)}$ for the four extremal states $(0, 0)$, $(0, \mathrm{D_{tot}})$, $(\mathrm{D_{tot}}, 0)(\mathrm{D_{tot}}, \mathrm{D_{tot}})$:

$$
\begin{aligned}
\bar{q}_{(0,0)} &= 2\left(\frac{(\epsilon)(k^2_{W0} + k^1_{W0} + \frac{\tilde{k}^R_W}{\Omega} p(\mathrm{D_{tot}}))}{u^A_0 + (\tilde{u}^A p + 1) + u^R_{20} + u^R_{10} + \tilde{u}^R p} \right) \mathrm{D_{tot}} \\
\bar{q}_{(0,\mathrm{D_{tot}})} &= \left(\frac{(\epsilon)(k^2_{W0} + k^1_{W0})}{u^A_0 + (\tilde{u}^A p + 1) + u^R_{20} + u^R_{10}} \right) \mathrm{D_{tot}} + \left(\frac{\mu(b\epsilon)\mu^{'}(\beta\epsilon)\bar{K}^{\mathrm{D_{tot}}}(k^A_{W0})}{u^A_0 + u^R_{20} + u^R_{10} + \tilde{u}^R p + (\alpha + \bar{\alpha} + \alpha^{\prime})} \right) \mathrm{D_{tot}} \\
\bar{q}_{(\mathrm{D_{tot}},0)} &= \left(\frac{(\epsilon)(k^2_{W0} + k^1_{W0})}{u^A_0 + (\tilde{u}^A p + 1) + u^R_{20} + u^R_{10}} \right) \mathrm{D_{tot}} + \left(\frac{\mu(b\epsilon)\mu^{'}(\beta\epsilon)\bar{K}^{\mathrm{D_{tot}}}(k^A_{W0})}{u^A_0 + u^R_{20} + u^R_{10} + \tilde{u}^R p + (\alpha + \bar{\alpha} + \alpha^{\prime})} \right) \mathrm{D_{tot}} \\
\bar{q}_{(\mathrm{D_{tot}},\mathrm{D_{tot}})} &= 2\left(\frac{\mu(b\epsilon)\mu^{'}(\beta\epsilon)\bar{K}^{\mathrm{D_{tot}}}(k^A_{W0})}{u^A_0 + u^R_{20} + u^R_{10} + (\alpha + \bar{\alpha} + \alpha^{\prime})} \right) \mathrm{D_{tot}}.
\end{aligned}
\tag{21}
$$

Then, if we assume $\epsilon^{'} \neq 0$ and $\epsilon \ll 1$, looking at the expressions in (21), it is possible to notice that $\bar{q}_{(0,0)}$, $\bar{q}_{(0,\mathrm{D_{tot}})}$, $\bar{q}_{(\mathrm{D_{tot}},0)}$ and $\bar{q}_{(\mathrm{D_{tot}},\mathrm{D_{tot}})}$ are very small $(\bar{q}_{(0,0)}, \bar{q}_{(0,\mathrm{D_{tot}})}, \bar{q}_{(\mathrm{D_{tot}},0)}, \bar{q}_{(\mathrm{D_{tot}},\mathrm{D_{tot}})} \approx 0)$

and then, by solving the system of equation in (19), we obtain that $\pi(s) \approx 0$ except for $s = (0,0), (0,\mathrm{D_{tot}}), (\mathrm{D_{tot}},0), (\mathrm{D_{tot}},\mathrm{D_{tot}})$. Since $\sum_{s=1}^{(\mathrm{D_{tot}}+1)^2} \pi(s) = 1$, we can conclude that, under the condition ϵ, $\pi(0,0) + \pi(0,\mathrm{D_{tot}}) + \pi(\mathrm{D_{tot}},0) + \pi(\mathrm{D_{tot}},\mathrm{D_{tot}}) \approx 1$. This implies that, for a sufficiently small ϵ, the peaks of the distribution are concentrated in the four extremal states and the probability of finding the state outside of these states approaches zero. Now, let us determine the effect of p on the distribution: in particular, if we assume a sufficiently high p ($p : \frac{1}{p} \ll \epsilon \ll 1$), it is possible to notice by comparing the expressions in (21) that $\bar{q}_{(0,\mathrm{D_{tot}})}, \bar{q}_{(\mathrm{D_{tot}},0)} \ll \bar{q}_{(0,0)}, \bar{q}_{(\mathrm{D_{tot}},\mathrm{D_{tot}})}$.Then, we can assume that $\bar{q}_{(0,\mathrm{D_{tot}})}, \bar{q}_{(\mathrm{D_{tot}},0)} \approx 0$. Now, by solving (19) and using the fact that $\sum_{s=1}^{(\mathrm{D_{tot}}\mathrm{D_{tot}}+1)^2} \pi(s) = 1$, we obtain that $\pi(s) \approx 0$ except for $s = (0,\mathrm{D_{tot}}), (\mathrm{D_{tot}},0)$ and then $\pi(0,\mathrm{D_{tot}}) + \pi(\mathrm{D_{tot}},0) \approx 1$. This implies that, for a sufficiently high p, the peaks of the distribution are concentrated only in correspondence of the two states $(0,\mathrm{D_{tot}})$ and $(\mathrm{D_{tot}},0)$.

3 Figures

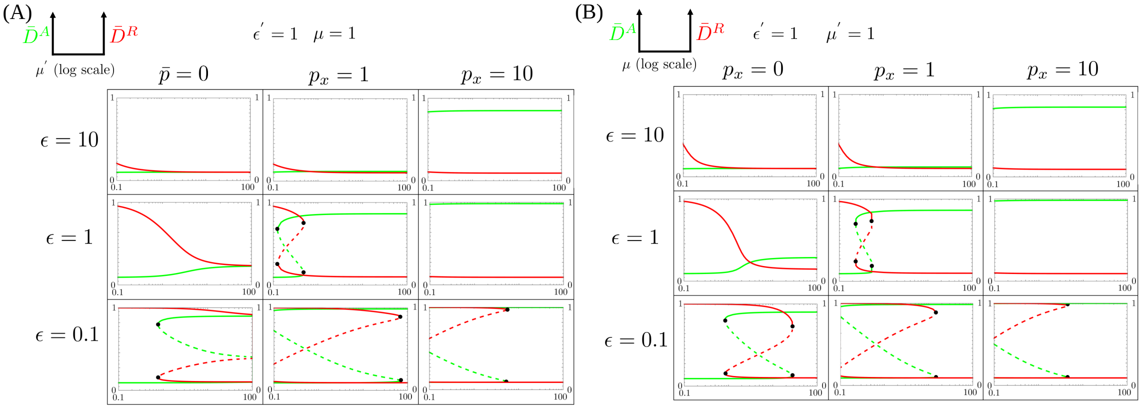

Figure A: **Bifurcation plots for different parameter values.** Bifurcation plots related to system (1) with no external inputs ($u^A = u_1^R = u_2^R = 0$ and $u_0^A = u_{10}^R = u_{20}^R = u_0$ small). For all the plots, solid lines represent stable steady states, dotted lines represent unstable steady states and the black circle represents the bifurcation point. In this case we have a saddle-node bifurcation. Furthermore, we set $\alpha = \bar{\alpha} = \alpha^{'} = 1$ and $\bar{\gamma}_x = 1$. (A) On the y axis we have $\bar{D}^A$ (green) and $\bar{D}^R = \bar{D}_1^R + \bar{D}_2^R + \bar{D}_{12}^R$ (red) and on the x axis we have $\mu^{'}$ (log scale). We realize several bifurcation plots for different values of ϵ ($\epsilon = 0.1, 1, 10$), different values of p_x ($p_x = 0, 1, 10$) and set $\epsilon^{'} = 1$ and $\mu = 1$. (B) On the y axis we have $\bar{D}^A$ (green) and $\bar{D}^R = \bar{D}_1^R + \bar{D}_2^R + \bar{D}_{12}^R$ (red) and on the x axis we have μ (log scale). We realize several bifurcation plots for different values of ϵ ($\epsilon = 0.1, 1, 10$), different values of p_x ($p_x = 0, 1, 10$) and set $\epsilon^{'} = 1$ and $\mu^{'} = 1$.

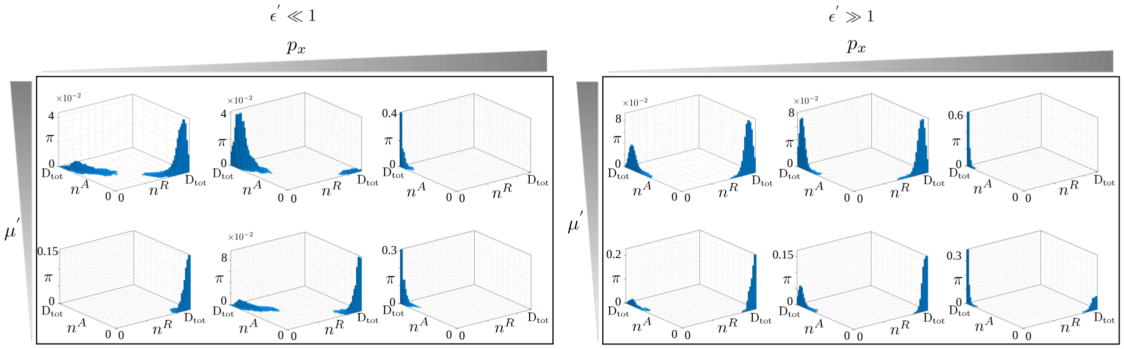

Figure B: **How the parameter p_x affects the stationary probability distribution for different values of ϵ'.** The stationary distribution of our system, represented by the circuit in Fig 5A, obtained computationally. The stationary distributions are obtained by simulating the system of reactions listed in Table B with the SSA and we indicate with n^R the total number of nucleosomes characterized by repressive chromatin modifications, that is $n^R = n_1^R + n_2^R + n_{12}^R$. We consider two different cases, $\epsilon' \ll 1$ and $\epsilon' \gg 1$, and for each case we determine how varying μ' and p_x affect the stationary probability distribution of the system. The parameter values of each regime are listed in Table B. In particular, we set $\epsilon = 0.12$, $\mu = 1$ and we consider two values of ϵ' ($\epsilon' = 10, 0.2$), three values of p_x ($p_x = 0, 0.1, 10$) and two values of μ' ($\mu' = 1, 0.5$).

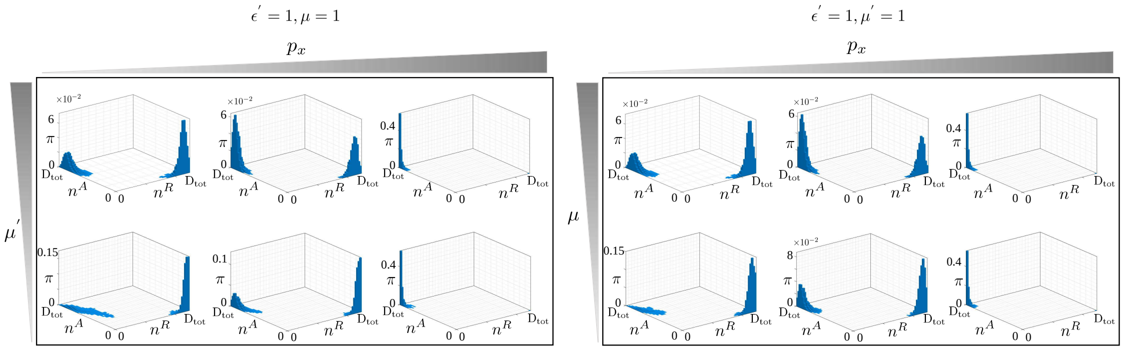

Figure C: **How the parameter p_x affects the stationary probability distribution for different values of μ.** The stationary distribution of our system, represented by the circuit in Fig 5A, obtained computationally. The stationary distributions are obtained by simulating the system of reactions listed in Table I with the SSA and we indicate with n^R the total number of nucleosomes characterized by repressive chromatin modifications, that is $n^R = n_1^R + n_2^R + n_{12}^R$. We consider two different cases: in the first one (left side) we set $\mu = 1$ and vary μ' (i.e.,$\mu' = 1, 0.5$) and in the second one (right side) we set $\mu' = 1$ and vary μ (i.e.,$\mu = 1, 0.5$). The parameter values of each regime are listed in Table I. In particular, for both cases we set $\epsilon = 0.12$, $\epsilon' = 1$ and we consider three values of p_x ($p_x = 0, 0.1, 10$).

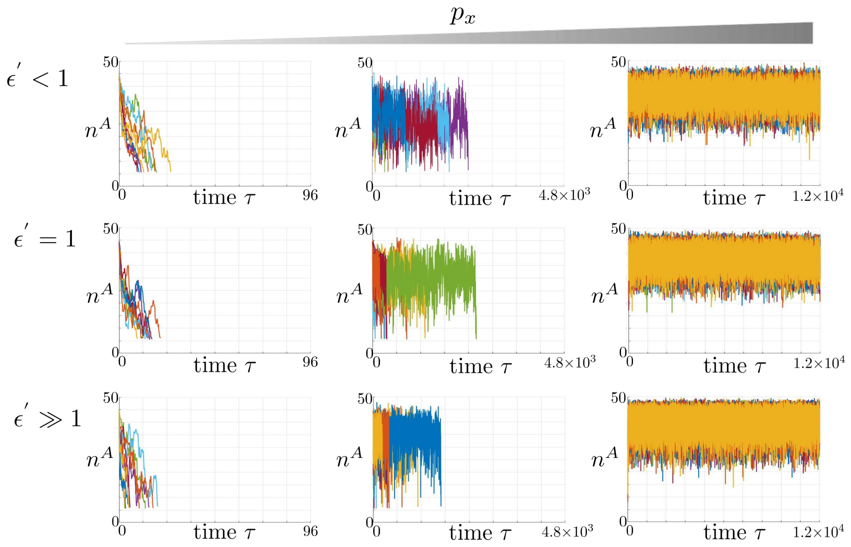

Figure D: **How the positive autoregulation affects the time to memory loss of the active state for different values of $\epsilon^{'}$.** We consider $\epsilon = 0.36$, $\mu^{'} = 0.5$, $\mu = 1$, three different values of $\epsilon^{'}$ ($\epsilon^{'} = 0.4, 1, 10$), three different values of p_x ($p_x = 0, 0.2, 5$) and we realize several time trajectories of the system starting with initial conditions $n^A = 45$, $n^R_{12} = 5$ and $n^X = p_x n^A$. Simulations are stopped the first time at which $n^A = 6$. In all plots, time is normalized according to$\tau = t\frac{k^A_M}{\Omega}\mathrm{D_{tot}}$. The parameter values of each panel are listed in Table C. In each panel, the number of trajectories plotted is 10.

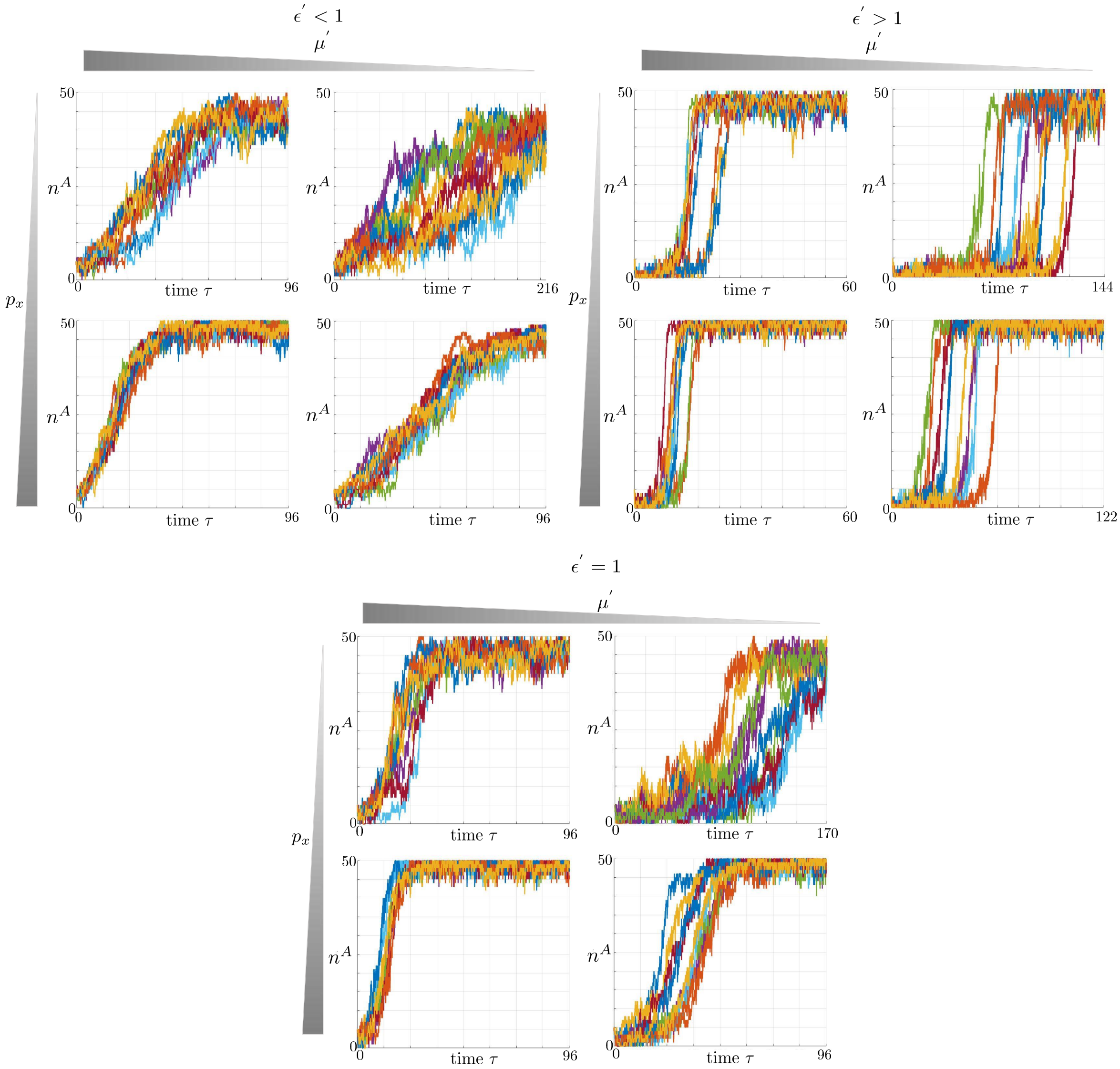

Figure E: **How the key parameters affect the reactivation of repressed state for different values of $\epsilon^{'}$.** Time trajectories of system starting from $n^R = 45$,$n^A = 5$,$n^X = p_x 5$ and considering an input u_0^A that leads to a unimodal distribution in correspondence of the active gene state. The parameter values of each panel are listed in Table J. In particular, we set $u^A = 1.62$, $\mu = 1$, $\epsilon = 0.16$ and we consider two values of $\mu^{'}$ ($\mu^{'} = 0.4, 0.2$), two values of p_x ($p_x = 0, 10$) and three values of $\epsilon^{'}$ ($\epsilon^{'} = 5, 1, 0.3$). In each panel, the number of trajectories plotted is 10.

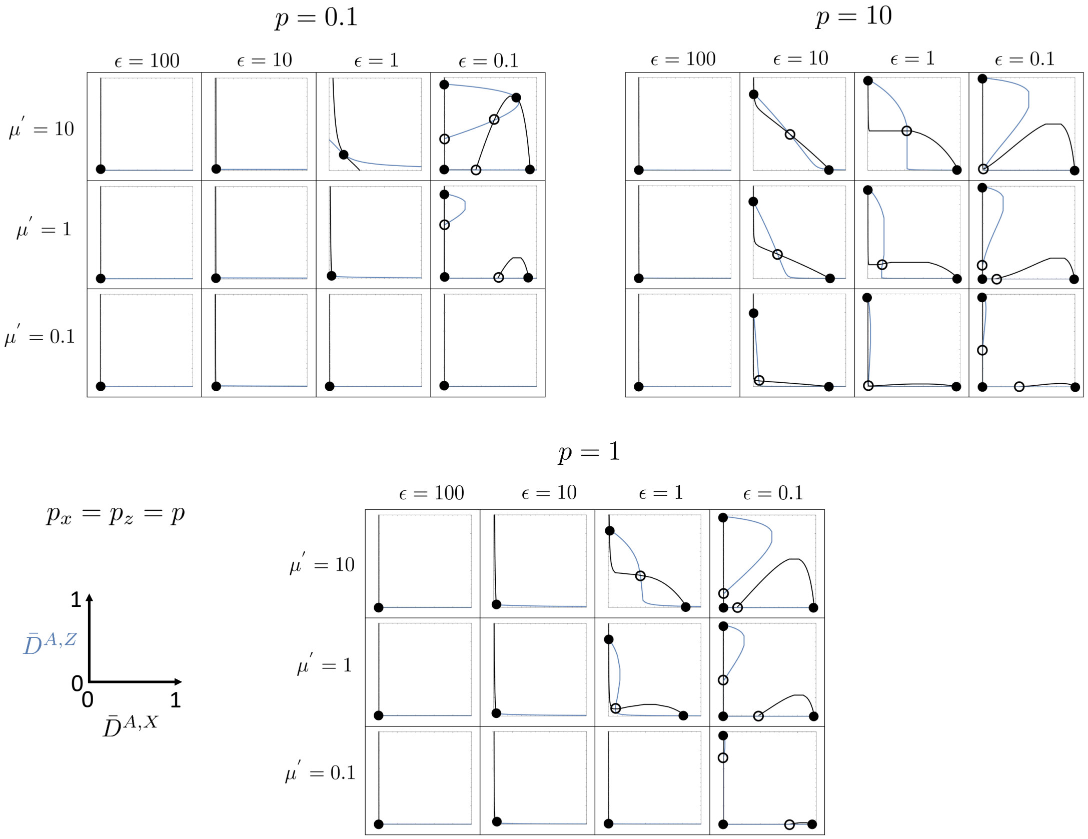

Figure F: **Location and stability of the equilibria as p_x, p_z, ϵ and μ' are changed.** Here, $p_x = p_z = p$ as indicated and we assume $\tilde{u}_1^R = \tilde{u}_2^R = \tilde{u}^R$. The filled circles represent stable steady states while the open circles represent unstable steady states. The blue line depicts the $(u^{R,Z}, \bar{D}^{A,Z})$ input/output steady state characteristic for the Z gene, with $u^{R,Z} = u_1^{R,Z} = u_2^{R,Z} = \tilde{u}^R \bar{X}$, that, at steady state, can be written as $u^{R,Z} = \tilde{u}^R p_x \bar{D}^{A,X}$ and the black line represents the $(u^{R,X}, \bar{D}^{A,X})$ input/output steady state characteristic of the X gene, with $u^{R,X} = u_1^{R,X} = u_2^{R,X} = \tilde{u}^R \bar{Z}$, that, at steady state, can be written as $u^{R,X} = \tilde{u}^R p_z \bar{D}^{A,Z}$. We consider four values of ϵ ($\epsilon = 100, 10, 1, 0.1$), three values of μ' ($\mu' = 10, 1, 0.1$), three values of p ($p = 10, 1, 0.1$), we set $u_0^A = u_{10}^R = u_{20}^R = u_0 = 0.1$ and all the other parameters are set equal to 1.

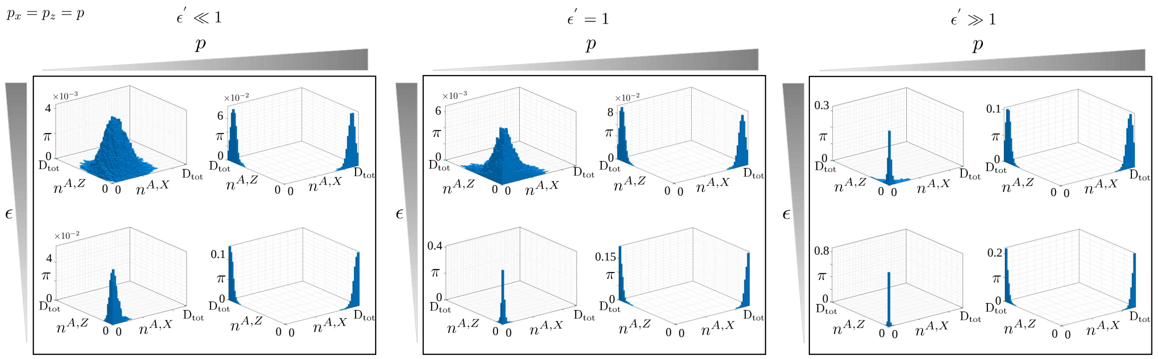

Figure G: **How the parameter p_x and p_z affect the stationary probability distribution for different values of $\epsilon^{'}$.** The stationary distribution of our system, represented by the circuit in Fig 6A, obtained computationally. The stationary distributions are obtained by simulating the system of reactions listed in Tables K-L with the SSA and we indicate with $n^{A,\ell}$ with $\ell = X, Z$ the total number of nucleosomes in each gene characterized by activating chromatin modifications. In particular, we consider $p_x = p_z$ (i.e.,the production and degradation rate constants are the same for protein X and Z) and we define p as $p = p_X = p_z$. Furthermore, we consider three different cases, $\epsilon^{'} \ll 1$ and $\epsilon^{'} = 1$ and $\epsilon^{'} \gg 1$, and for each case we determine how decreasing ϵ and increasing p affect the stationary probability distribution of the system. The parameter values of each regime are listed in Tables K-L. In particular, we set $\mu = 1$, $\mu^{'} = 0.6$ and we consider three values of $\epsilon^{'}$ ($\epsilon^{'} = 0.2, 1, 10$), two values of ϵ ($\epsilon = 0.48, 0.2$) and two values of p (i.e, $p = 0, 10$).

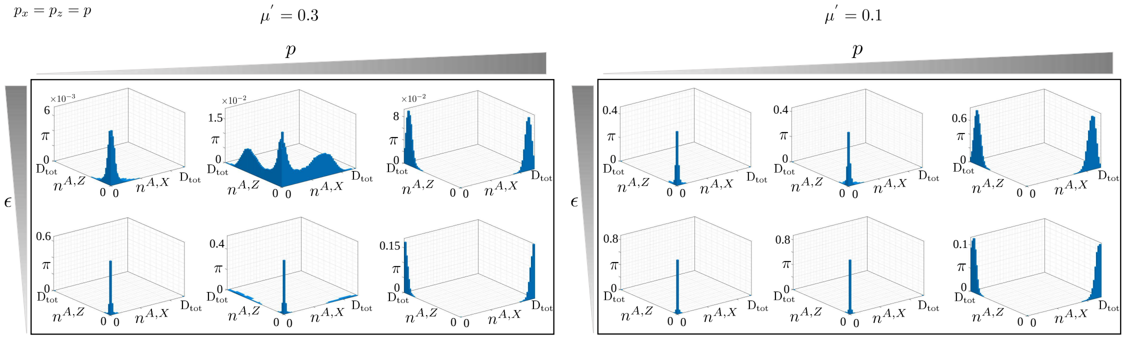

Figure H: **How the parameter p_x and p_z affect the stationary probability distribution for different values of $\mu^{'}$.** The stationary distribution of our system, represented by the circuit in Fig 6A, obtained computationally. The stationary distributions are obtained by simulating the system of reactions listed in Tables K-L with the SSA and we indicate with $n^{A,\ell}$ with $\ell = X, Z$ the total number of nucleosomes in each gene characterized by activating chromatin modifications. In particular, we consider $p_x = p_z$ (i.e.,the production and degradation rate constants are the same for protein X and Z) and we define p as $p = p_x = p_z$. Furthermore, we consider two different cases, $\mu^{'} = 0.3$ and $\mu^{'} = 0.1$, and for each case we determine how decreasing ϵ and increasing p affect the stationary probability distribution of the system. The parameter values of each regime are listed in Tables K-L. In particular, we set $\epsilon^{'} = 1$, $\mu = 1$ and we consider two values of ϵ ($\epsilon = 0.48, 0.2$) and three values of p (i.e, $p = 0, 0.1, 10$).

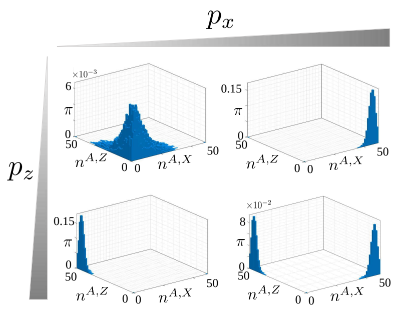

Figure I: **How the parameter p_x and p_z affect the stationary probability distribution.** The stationary distribution of the system obtained by simulating the reactions listed in Tables E-F with the SSA, in which by $n^{A,\ell}$ with $\ell = X, Z$ we denote the number of nucleosomes in each gene with activating histone modifications. Here, $\epsilon = 0.48$, $p_z = 0, 10$, and $p_x = 0, 10$. The parameter values of each plot are listed in Tables E-F. For all simulations we have $\mu = 1$, $\mu^{'} = 0.6$, and $\epsilon^{'} = 1$.

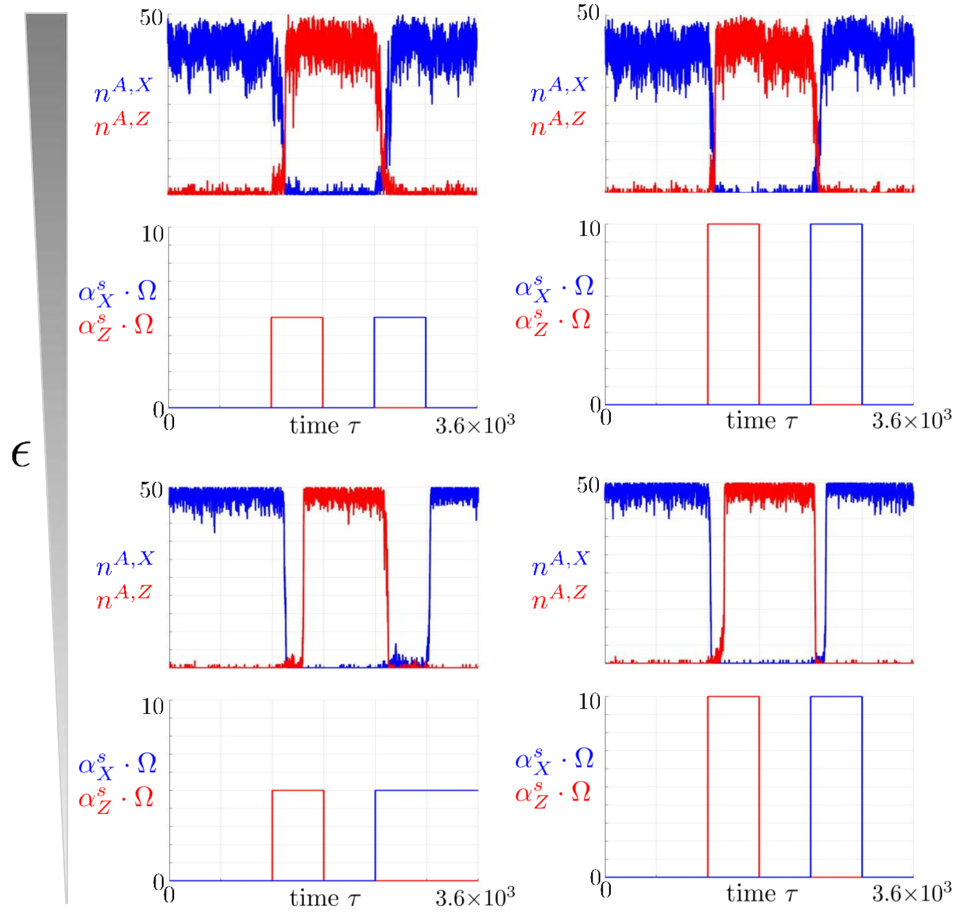

Figure J: **Mutual repression circuit enables long-term, yet reconfigurable memory of multiple gene expression patterns.** Time trajectories of $n^{A,X}$ and $n^{A,Z}$ starting from $n^{A,X} = \mathrm{D_{tot}}$ and $n^{A,Z} = 0$. Time is still normalized with respect to $\frac{k^A_M}{\Omega}\mathrm{D_{tot}}$. The reactions and parameter values are listed in Tables G-H at which we add the reactions $\emptyset \xrightarrow{\alpha^s_i} \mathrm{i}$ with $i =$ X,Z. The rate constants $\alpha^s_X \cdot \Omega$ and $\alpha^s_Z \cdot \Omega$ vary as shown in the Figure. In particular, $p = 0.15$, $\mu^{'} = 0.6$, $\mu = 1$, $\epsilon^{'} = 1$ and $\epsilon = 0.3, 0.07$. In all plots we assume equal parameters for both chromatin circuits.

4 Tables

It is important to point out that D_{tot} represents the total number of nucleosomes in a gene. Since we can assume about one nucleosome per 200 pb [2](Chapter 4) and we can assume that an average gene spans 10,000–20,000 bp [3], D_{tot} can be considered on average between 50 and 100. In particular, in our computational analysis we consider $D_{tot} = 50$.

**Param.**	**Value**
u_0^A	0.1
u_{10}^R	0.1
u_{20}^R	0.1
u_1^R	0
u_2^R	0
α	0.1
$\bar{\alpha}$	0.1
$\alpha^{'}$	0.1
ϵ	0.1
$\epsilon^{'}$	1
b	1
β	1
μ	1
$\mu^{'}$	0.7
$\tilde{u}^A$	6
$\bar{\alpha}_x$	0.5,1,5
$\bar{\gamma}_x$	1
$\bar{D}^R(0)$	0
$\bar{D}^A(0)$	1
$\bar{X}(0)$	$\frac{\bar{\alpha}_x}{\bar{\gamma}_x}$

Table A: **Parameter values relative to the plots in Fig 5C.**

$\mathbf{R}_j$	**Reaction**	**Prop.Func.**(a_j)	**Param.**	**Value** $(\mathbf{h}^{-1})$ Fig 5D	**Value** $(\mathbf{h}^{-1})$ Fig B left plots	**Value** $(\mathbf{h}^{-1})$ Fig B right plots
1	$\mathrm{D}+\mathrm{X} \xrightarrow{a} \mathrm{C_A}$	$a_1 = \frac{a}{\Omega} n^D n^X$	$\frac{a}{\Omega}$	10	10	10
2	$\mathrm{C_A} \xrightarrow{d} \mathrm{D}+\mathrm{X}$	$a_2 = d n_A^C$	d	10	10	10
3	$\mathrm{D} \xrightarrow{k_{W0}^A} \mathrm{D^A}$	$a_3 = k_{W0}^A n^D$	k_{W0}^A	3.5	3.5	3.5
4	$\mathrm{C_A} \xrightarrow{k_{W0}^A} \mathrm{D^A}+\mathrm{X}$	$a_4 = k_{W0}^A n^D$	k_{W0}^A	3.5	3.5	3.5
5	$\mathrm{C_A} \xrightarrow{k_W^A} \mathrm{D^A}+\mathrm{X}$	$a_5 = k_W^A n_A^C$	k_W^A	300	300	300
6	$\mathrm{D^A} \xrightarrow{\bar{k}_E^A} \mathrm{D}$	$a_6 = \bar{k}_E^A n^A$	$\bar{k}_E^A$	3	3	3
7	$\mathrm{D^A} \xrightarrow{\delta} \mathrm{D}$	$a_7 = \delta n^A$	δ	3	3	3
8	$\mathrm{D}+\mathrm{D^A} \xrightarrow{k_M^A} \mathrm{D^A}+\mathrm{D^A}$	$a_8 = \frac{k_M^A}{\Omega} n^D n^A$	$\frac{k_M^A}{\Omega}$	1	1	1
9	$\mathrm{C_A}+\mathrm{D^A} \xrightarrow{k_M^A} \mathrm{D^A}+\mathrm{D^A}+\mathrm{X}$	$a_9 = \frac{k_M^A}{\Omega} n_A^C n^A$	$\frac{k_M^A}{\Omega}$	1	1	1
10	$\mathrm{D^A}+\mathrm{D_1^R} \xrightarrow{k_E^A} \mathrm{D}+\mathrm{D_1^R}$	$a_{10} = \frac{k_E^A}{\Omega} n^A n_1^R$	$\frac{k_E^A}{\Omega}$	1	0.2	10
11	$\mathrm{D^A}+\mathrm{D_{12}^R} \xrightarrow{k_E^A} \mathrm{D}+\mathrm{D_{12}^R}$	$a_{11} = \frac{k_E^A}{\Omega} n^A n_{12}^R$	$\frac{k_E^A}{\Omega}$	1	0.2	10
12	$\mathrm{D^A}+\mathrm{D_2^R} \xrightarrow{k_E^A} \mathrm{D}+\mathrm{D_2^R}$	$a_{12} = \frac{k_E^A}{\Omega} n^A n_2^R$	$\frac{k_E^A}{\Omega}$	1	0.2	10
13	$\mathrm{D^A}+\mathrm{D_{12}^R} \xrightarrow{k_E^A} \mathrm{D}+\mathrm{D_{12}^R}$	$a_{13} = \frac{k_E^A}{\Omega} n^A n_{12}^R$	$\frac{k_E^A}{\Omega}$	1	0.2	10
14	$\mathrm{D} \xrightarrow{k_{W0}^1} \mathrm{D_1^R}$	$a_{14} = k_{W0}^1 n^D$	k_{W0}^1	3.5	3.5	3.5
15	$\mathrm{C_A} \xrightarrow{k_{W0}^1} \mathrm{D_1^R}+\mathrm{X}$	$a_{15} = k_{W0}^1 n^D$	k_{W0}^1	3.5	3.5	3.5
16	$\mathrm{D_1^R} \xrightarrow{k_T'} \mathrm{D}$	$a_{16} = k_T' n_1^R$	k_T'	1.5	3, 1.5	3, 1.5
17	$\mathrm{D_1^R} \xrightarrow{\delta'} \mathrm{D}$	$a_{17} = \delta' n_1^R$	δ'	1.5	3, 1.5	3, 1.5
18	$\mathrm{D}+\mathrm{D_2^R} \xrightarrow{k_M'} \mathrm{D_1^R}+\mathrm{D_2^R}$	$a_{18} = \frac{k_M'}{\Omega} n^D n_2^R$	$\frac{k_M'}{\Omega}$	0.2	0.2	0.2
19	$\mathrm{C_A}+\mathrm{D_2^R} \xrightarrow{k_M'} \mathrm{D_1^R}+\mathrm{D_2^R}+\mathrm{X}$	$a_{19} = \frac{k_M'}{\Omega} n^D n_2^R$	$\frac{k_M'}{\Omega}$	0.2	0.2	0.2
20	$\mathrm{D}+\mathrm{D_{12}^R} \xrightarrow{k_M'} \mathrm{D_1^R}+\mathrm{D_{12}^R}$	$a_{20} = \frac{k_M'}{\Omega} n^D n_{12}^R$	$\frac{k_M'}{\Omega}$	0.2	0.2	0.2
21	$\mathrm{C_A}+\mathrm{D_{12}^R} \xrightarrow{k_M'} \mathrm{D_1^R}+\mathrm{D_{12}^R}+\mathrm{X}$	$a_{21} = \frac{k_M'}{\Omega} n^D n_{12}^R$	$\frac{k_M'}{\Omega}$	0.2	0.2	0.2
22	$\mathrm{D_1^R}+\mathrm{D^A} \xrightarrow{k_T'^*} \mathrm{D}+\mathrm{D^A}$	$a_{22} = \frac{k_T'^*}{\Omega} n_1^R n^A$	$\frac{k_T'^*}{\Omega}$	0.5	0.2, 0.1	10, 5
23	$\mathrm{D} \xrightarrow{k_{W0}^2} \mathrm{D_2^R}$	$a_{23} = k_{W0}^2 n^D$	k_{W0}^2	3.5	3.5	3.5
24	$\mathrm{C_A} \xrightarrow{k_{W0}^2} \mathrm{D_2^R}+\mathrm{X}$	$a_{24} = k_{W0}^2 n^D$	k_{W0}^2	3.5	3.5	3.5
25	$\mathrm{D_2^R} \xrightarrow{\bar{k}_E^R} \mathrm{D}$	$a_{25} = \bar{k}_E^R n_2^R$	$\bar{k}_E^R$	3	3	3
26	$\mathrm{D_2^R} \xrightarrow{\delta} \mathrm{D}$	$a_{26} = \delta n_2^R$	δ	3	3	3
27	$\mathrm{D}+\mathrm{D_2^R} \xrightarrow{k_M} \mathrm{D_2^R}+\mathrm{D_2^R}$	$a_{27} = \frac{k_M}{\Omega} n^D n_2^R$	$\frac{k_M}{\Omega}$	0.2	0.2	0.2
28	$\mathrm{C_A}+\mathrm{D_2^R} \xrightarrow{k_M} \mathrm{D_2^R}+\mathrm{D_2^R}+\mathrm{X}$	$a_{28} = \frac{k_M}{\Omega} n^D n_2^R$	$\frac{k_M}{\Omega}$	0.2	0.2	0.2
29	$\mathrm{D}+\mathrm{D_{12}^R} \xrightarrow{k_M} \mathrm{D_2^R}+\mathrm{D_{12}^R}$	$a_{29} = \frac{k_M}{\Omega} n^D n_{12}^R$	$\frac{k_M}{\Omega}$	0.2	0.2	0.2
30	$\mathrm{C_A}+\mathrm{D_{12}^R} \xrightarrow{k_M} \mathrm{D_2^R}+\mathrm{D_{12}^R}+\mathrm{X}$	$a_{30} = \frac{k_M}{\Omega} n^D n_{12}^R$	$\frac{k_M}{\Omega}$	0.2	0.2	0.2
31	$\mathrm{D}+\mathrm{D_1^R} \xrightarrow{\bar{k}_M} \mathrm{D_2^R}+\mathrm{D_1^R}$	$a_{31} = \frac{\bar{k}_M}{\Omega} n^D n_1^R$	$\frac{\bar{k}_M}{\Omega}$	0.2	0.2	0.2
32	$\mathrm{C_A}+\mathrm{D_1^R} \xrightarrow{\bar{k}_M} \mathrm{D_2^R}+\mathrm{D_1^R}+\mathrm{X}$	$a_{32} = \frac{\bar{k}_M}{\Omega} n^D n_1^R$	$\frac{\bar{k}_M}{\Omega}$	0.2	0.2	0.2
33	$\mathrm{D}+\mathrm{D_{12}^R} \xrightarrow{\bar{k}_M} \mathrm{D_2^R}+\mathrm{D_{12}^R}$	$a_{33} = \frac{\bar{k}_M}{\Omega} n^D n_{12}^R$	$\frac{\bar{k}_M}{\Omega}$	0.2	0.2	0.2
34	$\mathrm{C_A}+\mathrm{D_{12}^R} \xrightarrow{\bar{k}_M} \mathrm{D_2^R}+\mathrm{D_{12}^R}+\mathrm{X}$	$a_{34} = \frac{\bar{k}_M}{\Omega} n^D n_{12}^R$	$\frac{\bar{k}_M}{\Omega}$	0.2	0.2	0.2
35	$\mathrm{D_2^R}+\mathrm{D^A} \xrightarrow{k_E^R} \mathrm{D}+\mathrm{D^A}$	$a_{35} = \frac{k_E^R}{\Omega} n_2^R n^A$	$\frac{k_E^R}{\Omega}$	1	0.2	10
36	$\mathrm{D_1^R} \xrightarrow{k_{W0}^2} \mathrm{D_{12}^R}$	$a_{36} = k_{W0}^2 n_1^R$	k_{W0}^2	3.5	3.5	3.5
37	$\mathrm{D_{12}^R} \xrightarrow{\bar{k}_E^R} \mathrm{D_1^R}$	$a_{37} = \bar{k}_E^R n_{12}^R$	$\bar{k}_E^R$	3	3	3
38	$\mathrm{D_{12}^R} \xrightarrow{\delta} \mathrm{D_1^R}$	$a_{38} = \delta n_{12}^R$	δ	3	3	3
39	$\mathrm{D_1^R}+\mathrm{D_2^R} \xrightarrow{k_M} \mathrm{D_{12}^R}+\mathrm{D_2^R}$	$a_{39} = \frac{k_M}{\Omega} n_1^R n_2^R$	$\frac{k_M}{\Omega}$	0.2	0.2	0.2
40	$\mathrm{D_1^R}+\mathrm{D_{12}^R} \xrightarrow{k_M} \mathrm{D_{12}^R}+\mathrm{D_{12}^R}$	$a_{40} = \frac{k_M}{\Omega} n_1^R n_{12}^R$	$\frac{k_M}{\Omega}$	0.2	0.2	0.2
41	$\mathrm{D_1^R}+\mathrm{D_1^R} \xrightarrow{\bar{k}_M} \mathrm{D_{12}^R}+\mathrm{D_1^R}$	$a_{41} = \frac{\bar{k}_M}{\Omega} \frac{n_1^R(n_1^R-1)}{2}$	$\frac{\bar{k}_M}{\Omega}$	0.2	0.2	0.2
42	$\mathrm{D_1^R}+\mathrm{D_{12}^R} \xrightarrow{\bar{k}_M} \mathrm{D_{12}^R}+\mathrm{D_{12}^R}$	$a_{42} = \frac{\bar{k}_M}{\Omega} n_1^R n_{12}^R$	$\frac{\bar{k}_M}{\Omega}$	0.2	0.2	0.2
43	$\mathrm{D_{12}^R}+\mathrm{D^A} \xrightarrow{k_E^R} \mathrm{D_1^R}+\mathrm{D^A}$	$a_{43} = \frac{k_E^R}{\Omega} n_{12}^R n^A$	$\frac{k_E^R}{\Omega}$	1	0.2	10
44	$\mathrm{D_2^R} \xrightarrow{k_{W0}^1} \mathrm{D_{12}^R}$	$a_{44} = k_{W0}^1 n_2^R$	k_{W0}^1	3.5	3.5	3.5
45	$\mathrm{D_{12}^R} \xrightarrow{k_T'} \mathrm{D_2^R}$	$a_{45} = k_T' n_{12}^R$	k_T'	1.5	3, 1.5	3, 1.5
46	$\mathrm{D_{12}^R} \xrightarrow{\delta'} \mathrm{D_2^R}$	$a_{46} = \delta' n_{12}^R$	δ'	1.5	3, 1.5	3, 1.5
47	$\mathrm{D_2^R}+\mathrm{D_2^R} \xrightarrow{k_M'} \mathrm{D_{12}^R}+\mathrm{D_2^R}$	$a_{47} = \frac{k_M'}{\Omega} \frac{n_2^R(n_2^R-1)}{2}$	$\frac{k_M'}{\Omega}$	0.2	0.2	0.2
48	$\mathrm{D_2^R}+\mathrm{D_{12}^R} \xrightarrow{k_M'} \mathrm{D_{12}^R}+\mathrm{D_{12}^R}$	$a_{48} = \frac{k_M'}{\Omega} n_2^R n_{12}^R$	$\frac{k_M'}{\Omega}$	0.2	0.2	0.2
49	$\mathrm{D_{12}^R}+\mathrm{D^A} \xrightarrow{k_T'^*} \mathrm{D_2^R}+\mathrm{D^A}$	$a_{49} = \frac{k_T'^*}{\Omega} n_{12}^R n^A$	$\frac{k_T'^*}{\Omega}$	0.5	0.2, 0.1	10, 5
50	$\mathrm{D^A} \xrightarrow{\alpha_x} \mathrm{D^A}+\mathrm{X}$	$a_{50} = \alpha_x n^A$	α_x	0, 0.1, 10	0, 0.1, 10	0, 0.1, 10
51	$\mathrm{X} \xrightarrow{\gamma_x} \emptyset$	$a_{51} = \gamma_x n^X$	γ_x	1	1	1
52	$\mathrm{C_A} \xrightarrow{\delta} \mathrm{D}+\mathrm{X}$	$a_{52} = \delta n^A$	δ	3	3	3

Table B: **Reactions and parameter values used to generate the plots in Figs 5D and B.**

$\mathbf{R}_j$	**Reaction**	**Prop.Func.**(a_j)	**Param.**	**Value** ($\mathbf{h}^{-1}$)
1	$\mathrm{D} + \mathrm{X} \xrightarrow{a} \mathrm{C_A}$	$a_1 = \frac{a}{\Omega} n^D n^X$	$\frac{a}{\Omega}$	10
2	$\mathrm{C_A} \xrightarrow{d} \mathrm{D} + \mathrm{X}$	$a_2 = d n_A^C$	d	10
3	$\mathrm{D} \xrightarrow{k_{W0}^A} \mathrm{D^A}$	$a_3 = k_{W0}^A n^D$	k_{W0}^A	5
4	$\mathrm{C_A} \xrightarrow{k_{W0}^A} \mathrm{D^A} + \mathrm{X}$	$a_4 = k_{W0}^A n^D$	k_{W0}^A	5
5	$\mathrm{C_A} \xrightarrow{k_W^A} \mathrm{D^A} + \mathrm{X}$	$a_5 = k_W^A n_A^C$	k_W^A	50
6	$\mathrm{D^A} \xrightarrow{\bar{k}_E^A} \mathrm{D}$	$a_6 = \bar{k}_E^A n^A$	$\bar{k}_E^A$	9
7	$\mathrm{D^A} \xrightarrow{\delta} \mathrm{D}$	$a_7 = \delta n^A$	δ	9
8	$\mathrm{D} + \mathrm{D^A} \xrightarrow{k_M^A} \mathrm{D^A} + \mathrm{D^A}$	$a_8 = \frac{k_M^A}{\Omega} n^D n^A$	$\frac{k_M^A}{\Omega}$	1
9	$\mathrm{C_A} + \mathrm{D^A} \xrightarrow{k_M^A} \mathrm{D^A} + \mathrm{D^A} + \mathrm{X}$	$a_9 = \frac{k_M^A}{\Omega} n_A^C n^A$	$\frac{k_M^A}{\Omega}$	1
10	$\mathrm{D^A} + \mathrm{D_1^R} \xrightarrow{k_E^A} \mathrm{D} + \mathrm{D_1^R}$	$a_{10} = \frac{k_E^A}{\Omega} n^A n_1^R$	$\frac{k_E^A}{\Omega}$	0.4, 1, 10
11	$\mathrm{D^A} + \mathrm{D_{12}^R} \xrightarrow{k_E^A} \mathrm{D} + \mathrm{D_{12}^R}$	$a_{11} = \frac{k_E^A}{\Omega} n^A n_{12}^R$	$\frac{k_E^A}{\Omega}$	0.4, 1, 10
12	$\mathrm{D^A} + \mathrm{D_2^R} \xrightarrow{k_E^A} \mathrm{D} + \mathrm{D_2^R}$	$a_{12} = \frac{k_E^A}{\Omega} n^A n_2^R$	$\frac{k_E^A}{\Omega}$	0.4, 1, 10
13	$\mathrm{D^A} + \mathrm{D_{12}^R} \xrightarrow{k_E^A} \mathrm{D} + \mathrm{D_{12}^R}$	$a_{13} = \frac{k_E^A}{\Omega} n^A n_{12}^R$	$\frac{k_E^A}{\Omega}$	0.4, 1, 10
14	$\mathrm{D} \xrightarrow{k_{W0}^1} \mathrm{D_1^R}$	$a_{14} = k_{W0}^1 n^D$	k_{W0}^1	5
15	$\mathrm{C_A} \xrightarrow{k_{W0}^1} \mathrm{D_1^R} + \mathrm{X}$	$a_{15} = k_{W0}^1 n^D$	k_{W0}^1	5
16	$\mathrm{D_1^R} \xrightarrow{k_T'} \mathrm{D}$	$a_{16} = k_T' n_1^R$	k_T'	4.5
17	$\mathrm{D_1^R} \xrightarrow{\delta'} \mathrm{D}$	$a_{17} = \delta' n_1^R$	δ'	4.5
18	$\mathrm{D} + \mathrm{D_2^R} \xrightarrow{k_M'} \mathrm{D_1^R} + \mathrm{D_2^R}$	$a_{18} = \frac{k_M'}{\Omega} n^D n_2^R$	$\frac{k_M'}{\Omega}$	0.2
19	$\mathrm{C_A} + \mathrm{D_2^R} \xrightarrow{k_M'} \mathrm{D_1^R} + \mathrm{D_2^R} + \mathrm{X}$	$a_{19} = \frac{k_M'}{\Omega} n^D n_2^R$	$\frac{k_M'}{\Omega}$	0.2
20	$\mathrm{D} + \mathrm{D_{12}^R} \xrightarrow{k_M'} \mathrm{D_1^R} + \mathrm{D_{12}^R}$	$a_{20} = \frac{k_M'}{\Omega} n^D n_{12}^R$	$\frac{k_M'}{\Omega}$	0.2
21	$\mathrm{C_A} + \mathrm{D_{12}^R} \xrightarrow{k_M'} \mathrm{D_1^R} + \mathrm{D_{12}^R} + \mathrm{X}$	$a_{21} = \frac{k_M'}{\Omega} n^D n_{12}^R$	$\frac{k_M'}{\Omega}$	0.2
22	$\mathrm{D_1^R} + \mathrm{D^A} \xrightarrow{k_T'^*} \mathrm{D} + \mathrm{D^A}$	$a_{22} = \frac{k_T'^*}{\Omega} n_1^R n^A$	$\frac{k_T'^*}{\Omega}$	0.2, 0.5, 5
23	$\mathrm{D} \xrightarrow{k_{W0}^2} \mathrm{D_2^R}$	$a_{23} = k_{W0}^2 n^D$	k_{W0}^2	5
24	$\mathrm{C_A} \xrightarrow{k_{W0}^2} \mathrm{D_2^R} + \mathrm{X}$	$a_{24} = k_{W0}^2 n^D$	k_{W0}^2	5
25	$\mathrm{D_2^R} \xrightarrow{\bar{k}_E^R} \mathrm{D}$	$a_{25} = \bar{k}_E^R n_2^R$	$\bar{k}_E^R$	9
26	$\mathrm{D_2^R} \xrightarrow{\delta} \mathrm{D}$	$a_{26} = \delta n_2^R$	δ	9
27	$\mathrm{D} + \mathrm{D_2^R} \xrightarrow{k_M} \mathrm{D_2^R} + \mathrm{D_2^R}$	$a_{27} = \frac{k_M}{\Omega} n^D n_2^R$	$\frac{k_M}{\Omega}$	0.2
28	$\mathrm{C_A} + \mathrm{D_2^R} \xrightarrow{k_M} \mathrm{D_2^R} + \mathrm{D_2^R} + \mathrm{X}$	$a_{28} = \frac{k_M}{\Omega} n^D n_2^R$	$\frac{k_M}{\Omega}$	0.2
29	$\mathrm{D} + \mathrm{D_{12}^R} \xrightarrow{k_M} \mathrm{D_2^R} + \mathrm{D_{12}^R}$	$a_{29} = \frac{k_M}{\Omega} n^D n_{12}^R$	$\frac{k_M}{\Omega}$	0.2
30	$\mathrm{C_A} + \mathrm{D_{12}^R} \xrightarrow{k_M} \mathrm{D_2^R} + \mathrm{D_{12}^R} + \mathrm{X}$	$a_{30} = \frac{k_M}{\Omega} n^D n_{12}^R$	$\frac{k_M}{\Omega}$	0.2
31	$\mathrm{D} + \mathrm{D_1^R} \xrightarrow{\bar{k}_M} \mathrm{D_2^R} + \mathrm{D_1^R}$	$a_{31} = \frac{\bar{k}_M}{\Omega} n^D n_1^R$	$\frac{\bar{k}_M}{\Omega}$	0.2
32	$\mathrm{C_A} + \mathrm{D_1^R} \xrightarrow{\bar{k}_M} \mathrm{D_2^R} + \mathrm{D_1^R} + \mathrm{X}$	$a_{32} = \frac{\bar{k}_M}{\Omega} n^D n_1^R$	$\frac{\bar{k}_M}{\Omega}$	0.2
33	$\mathrm{D} + \mathrm{D_{12}^R} \xrightarrow{\bar{k}_M} \mathrm{D_2^R} + \mathrm{D_{12}^R}$	$a_{33} = \frac{\bar{k}_M}{\Omega} n^D n_{12}^R$	$\frac{\bar{k}_M}{\Omega}$	0.2
34	$\mathrm{C_A} + \mathrm{D_{12}^R} \xrightarrow{\bar{k}_M} \mathrm{D_2^R} + \mathrm{D_{12}^R} + \mathrm{X}$	$a_{34} = \frac{\bar{k}_M}{\Omega} n^D n_{12}^R$	$\frac{\bar{k}_M}{\Omega}$	0.2
35	$\mathrm{D_2^R} + \mathrm{D^A} \xrightarrow{k_E^R} \mathrm{D} + \mathrm{D^A}$	$a_{35} = \frac{k_E^R}{\Omega} n_2^R n^A$	$\frac{k_E^R}{\Omega}$	0.4, 1, 10
36	$\mathrm{D_1^R} \xrightarrow{k_{W0}^2} \mathrm{D_{12}^R}$	$a_{36} = k_{W0}^2 n_1^R$	k_{W0}^2	5
37	$\mathrm{D_{12}^R} \xrightarrow{\bar{k}_E^R} \mathrm{D_1^R}$	$a_{37} = \bar{k}_E^R n_{12}^R$	$\bar{k}_E^R$	9
38	$\mathrm{D_{12}^R} \xrightarrow{\delta} \mathrm{D_1^R}$	$a_{38} = \delta n_{12}^R$	δ	9
39	$\mathrm{D_1^R} + \mathrm{D_2^R} \xrightarrow{k_M} \mathrm{D_{12}^R} + \mathrm{D_2^R}$	$a_{39} = \frac{k_M}{\Omega} n_1^R n_2^R$	$\frac{k_M}{\Omega}$	0.2
40	$\mathrm{D_1^R} + \mathrm{D_{12}^R} \xrightarrow{k_M} \mathrm{D_{12}^R} + \mathrm{D_{12}^R}$	$a_{40} = \frac{k_M}{\Omega} n_1^R n_{12}^R$	$\frac{k_M}{\Omega}$	0.2
41	$\mathrm{D_1^R} + \mathrm{D_1^R} \xrightarrow{\bar{k}_M} \mathrm{D_{12}^R} + \mathrm{D_1^R}$	$a_{41} = \frac{\bar{k}_M}{\Omega} \frac{n_1^R(n_1^R-1)}{2}$	$\frac{\bar{k}_M}{\Omega}$	0.2
42	$\mathrm{D_1^R} + \mathrm{D_{12}^R} \xrightarrow{\bar{k}_M} \mathrm{D_{12}^R} + \mathrm{D_{12}^R}$	$a_{42} = \frac{\bar{k}_M}{\Omega} n_1^R n_{12}^R$	$\frac{\bar{k}_M}{\Omega}$	0.2
43	$\mathrm{D_{12}^R} + \mathrm{D^A} \xrightarrow{k_E^R} \mathrm{D_1^R} + \mathrm{D^A}$	$a_{43} = \frac{k_E^R}{\Omega} n_{12}^R n^A$	$\frac{k_E^R}{\Omega}$	0.4, 1, 10
44	$\mathrm{D_2^R} \xrightarrow{k_{W0}^1} \mathrm{D_{12}^R}$	$a_{44} = k_{W0}^1 n_2^R$	k_{W0}^1	5
45	$\mathrm{D_{12}^R} \xrightarrow{k_T'} \mathrm{D_2^R}$	$a_{45} = k_T' n_{12}^R$	k_T'	4.5
46	$\mathrm{D_{12}^R} \xrightarrow{\delta'} \mathrm{D_2^R}$	$a_{46} = \delta' n_{12}^R$	δ'	4.5
47	$\mathrm{D_2^R} + \mathrm{D_2^R} \xrightarrow{k_M'} \mathrm{D_{12}^R} + \mathrm{D_2^R}$	$a_{47} = \frac{k_M'}{\Omega} \frac{n_2^R(n_2^R-1)}{2}$	$\frac{k_M'}{\Omega}$	0.2
48	$\mathrm{D_2^R} + \mathrm{D_{12}^R} \xrightarrow{k_M'} \mathrm{D_{12}^R} + \mathrm{D_{12}^R}$	$a_{48} = \frac{k_M'}{\Omega} n_2^R n_{12}^R$	$\frac{k_M'}{\Omega}$	0.2
49	$\mathrm{D_{12}^R} + \mathrm{D^A} \xrightarrow{k_T'^*} \mathrm{D_2^R} + \mathrm{D^A}$	$a_{49} = \frac{k_T'^*}{\Omega} n_{12}^R n^A$	$\frac{k_T'^*}{\Omega}$	0.2
50	$\mathrm{D^A} \xrightarrow{\alpha_x} \mathrm{D^A} + \mathrm{X}$	$a_{50} = \alpha_x n^A$	α_x	0, 0.2, 5
51	$\mathrm{X} \xrightarrow{\gamma_x} \emptyset$	$a_{51} = \gamma_x n^X$	γ_x	1
52	$\mathrm{C_A} \xrightarrow{\delta} \mathrm{D} + \mathrm{X}$	$a_{52} = \delta n^A$	δ	9

Table C: **Reactions and parameter values used to generate the plots in Fig D.** Furthermore, these (with $\frac{k_E^A}{\Omega} = 1$, $\frac{k_E^R}{\Omega} = 1$ and $\frac{k_T'^*}{\Omega} = 1, 0.5$) are also the parameter values used for the simulations in Fig 5E.

$\mathbf{R}_j$	**Reaction**	**Prop.Func.**(a_j)	**Param.**	**Value** ($\mathbf{h}^{-1}$)
1	$\mathrm{D}+\mathrm{X} \xrightarrow{a} \mathrm{C_A}$	$a_1 = \frac{a}{\Omega} n^D n^X$	$\frac{a}{\Omega}$	10
2	$\mathrm{C_A} \xrightarrow{d} \mathrm{D}+\mathrm{X}$	$a_2 = d n_A^C$	d	10
3	$\mathrm{D} \xrightarrow{k_{W0}^A} \mathrm{D^A}$	$a_3 = k_{W0}^A n^D$	k_{W0}^A	5
4	$\mathrm{C_A} \xrightarrow{k_{W0}^A} \mathrm{D^A}+\mathrm{X}$	$a_4 = k_{W0}^A n^D$	k_{W0}^A	5
5	$\mathrm{C_A} \xrightarrow{k_W^A} \mathrm{D^A}+\mathrm{X}$	$a_5 = k_W^A n_A^C$	k_W^A	300
6	$\mathrm{D^A} \xrightarrow{\bar{k}_E^A} \mathrm{D}$	$a_6 = \bar{k}_E^A n^A$	$\bar{k}_E^A$	10, 0.5
7	$\mathrm{D^A} \xrightarrow{\delta} \mathrm{D}$	$a_7 = \delta n^A$	δ	10, 0.5
8	$\mathrm{D}+\mathrm{D^A} \xrightarrow{k_M^A} \mathrm{D^A}+\mathrm{D^A}$	$a_8 = \frac{k_M^A}{\Omega} n^D n^A$	$\frac{k_M^A}{\Omega}$	1
9	$\mathrm{C_A}+\mathrm{D^A} \xrightarrow{k_M^A} \mathrm{D^A}+\mathrm{D^A}+\mathrm{X}$	$a_9 = \frac{k_M^A}{\Omega} n_A^C n^A$	$\frac{k_M^A}{\Omega}$	1
10	$\mathrm{D^A}+\mathrm{D_1^R} \xrightarrow{k_E^A} \mathrm{D}+\mathrm{D_1^R}$	$a_{10} = \frac{k_E^A}{\Omega} n^A n_1^R$	$\frac{k_E^A}{\Omega}$	1
11	$\mathrm{D^A}+\mathrm{D_{12}^R} \xrightarrow{k_E^A} \mathrm{D}+\mathrm{D_{12}^R}$	$a_{11} = \frac{k_E^A}{\Omega} n^A n_{12}^R$	$\frac{k_E^A}{\Omega}$	1
12	$\mathrm{D^A}+\mathrm{D_2^R} \xrightarrow{k_E^A} \mathrm{D}+\mathrm{D_2^R}$	$a_{12} = \frac{k_E^A}{\Omega} n^A n_2^R$	$\frac{k_E^A}{\Omega}$	1
13	$\mathrm{D^A}+\mathrm{D_{12}^R} \xrightarrow{k_E^A} \mathrm{D}+\mathrm{D_{12}^R}$	$a_{13} = \frac{k_E^A}{\Omega} n^A n_{12}^R$	$\frac{k_E^A}{\Omega}$	1
14	$\mathrm{D} \xrightarrow{k_{W0}^1} \mathrm{D_1^R}$	$a_{14} = k_{W0}^1 n^D$	k_{W0}^1	5
15	$\mathrm{C_A} \xrightarrow{k_{W0}^1} \mathrm{D_1^R}+\mathrm{X}$	$a_{15} = k_{W0}^1 n^D$	k_{W0}^1	5
16	$\mathrm{D_1^R} \xrightarrow{k_T'} \mathrm{D}$	$a_{16} = k_T' n_1^R$	k_T'	1, 0.05
17	$\mathrm{D_1^R} \xrightarrow{\delta'} \mathrm{D}$	$a_{17} = \delta' n_1^R$	δ'	1, 0.05
18	$\mathrm{D}+\mathrm{D_2^R} \xrightarrow{k_M'} \mathrm{D_1^R}+\mathrm{D_2^R}$	$a_{18} = \frac{k_M'}{\Omega} n^D n_2^R$	$\frac{k_M'}{\Omega}$	0.2
19	$\mathrm{C_A}+\mathrm{D_2^R} \xrightarrow{k_M'} \mathrm{D_1^R}+\mathrm{D_2^R}+\mathrm{X}$	$a_{19} = \frac{k_M'}{\Omega} n^D n_2^R$	$\frac{k_M'}{\Omega}$	0.2
20	$\mathrm{D}+\mathrm{D_{12}^R} \xrightarrow{k_M'} \mathrm{D_1^R}+\mathrm{D_{12}^R}$	$a_{20} = \frac{k_M'}{\Omega} n^D n_{12}^R$	$\frac{k_M'}{\Omega}$	0.2
21	$\mathrm{C_A}+\mathrm{D_{12}^R} \xrightarrow{k_M'} \mathrm{D_1^R}+\mathrm{D_{12}^R}+\mathrm{X}$	$a_{21} = \frac{k_M'}{\Omega} n^D n_{12}^R$	$\frac{k_M'}{\Omega}$	0.2
22	$\mathrm{D_1^R}+\mathrm{D^A} \xrightarrow{k_T'^*} \mathrm{D}+\mathrm{D^A}$	$a_{22} = \frac{k_T'^*}{\Omega} n_1^R n^A$	$\frac{k_T'^*}{\Omega}$	0.1
23	$\mathrm{D} \xrightarrow{k_{W0}^2} \mathrm{D_2^R}$	$a_{23} = k_{W0}^2 n^D$	k_{W0}^2	5
24	$\mathrm{C_A} \xrightarrow{k_{W0}^2} \mathrm{D_2^R}+\mathrm{X}$	$a_{24} = k_{W0}^2 n^D$	k_{W0}^2	5
25	$\mathrm{D_2^R} \xrightarrow{\bar{k}_E^R} \mathrm{D}$	$a_{25} = \bar{k}_E^R n_2^R$	$\bar{k}_E^R$	10, 0.5
26	$\mathrm{D_2^R} \xrightarrow{\delta} \mathrm{D}$	$a_{26} = \delta n_2^R$	δ	10, 0.5
27	$\mathrm{D}+\mathrm{D_2^R} \xrightarrow{k_M} \mathrm{D_2^R}+\mathrm{D_2^R}$	$a_{27} = \frac{k_M}{\Omega} n^D n_2^R$	$\frac{k_M}{\Omega}$	0.2
28	$\mathrm{C_A}+\mathrm{D_2^R} \xrightarrow{k_M} \mathrm{D_2^R}+\mathrm{D_2^R}+\mathrm{X}$	$a_{28} = \frac{k_M}{\Omega} n^D n_2^R$	$\frac{k_M}{\Omega}$	0.2
29	$\mathrm{D}+\mathrm{D_{12}^R} \xrightarrow{k_M} \mathrm{D_2^R}+\mathrm{D_{12}^R}$	$a_{29} = \frac{k_M}{\Omega} n^D n_{12}^R$	$\frac{k_M}{\Omega}$	0.2
30	$\mathrm{C_A}+\mathrm{D_{12}^R} \xrightarrow{k_M} \mathrm{D_2^R}+\mathrm{D_{12}^R}+\mathrm{X}$	$a_{30} = \frac{k_M}{\Omega} n^D n_{12}^R$	$\frac{k_M}{\Omega}$	0.2
31	$\mathrm{D}+\mathrm{D_1^R} \xrightarrow{\bar{k}_M} \mathrm{D_2^R}+\mathrm{D_1^R}$	$a_{31} = \frac{\bar{k}_M}{\Omega} n^D n_1^R$	$\frac{\bar{k}_M}{\Omega}$	0.2
32	$\mathrm{C_A}+\mathrm{D_1^R} \xrightarrow{\bar{k}_M} \mathrm{D_2^R}+\mathrm{D_1^R}+\mathrm{X}$	$a_{32} = \frac{\bar{k}_M}{\Omega} n^D n_1^R$	$\frac{\bar{k}_M}{\Omega}$	0.2
33	$\mathrm{D}+\mathrm{D_{12}^R} \xrightarrow{\bar{k}_M} \mathrm{D_2^R}+\mathrm{D_{12}^R}$	$a_{33} = \frac{\bar{k}_M}{\Omega} n^D n_{12}^R$	$\frac{\bar{k}_M}{\Omega}$	0.2
34	$\mathrm{C_A}+\mathrm{D_{12}^R} \xrightarrow{\bar{k}_M} \mathrm{D_2^R}+\mathrm{D_{12}^R}+\mathrm{X}$	$a_{34} = \frac{\bar{k}_M}{\Omega} n^D n_{12}^R$	$\frac{\bar{k}_M}{\Omega}$	0.2
35	$\mathrm{D_2^R}+\mathrm{D^A} \xrightarrow{k_E^R} \mathrm{D}+\mathrm{D^A}$	$a_{35} = \frac{k_E^R}{\Omega} n_2^R n^A$	$\frac{k_E^R}{\Omega}$	1
36	$\mathrm{D_1^R} \xrightarrow{k_{W0}^2} \mathrm{D_{12}^R}$	$a_{36} = k_{W0}^2 n_1^R$	k_{W0}^2	5
37	$\mathrm{D_{12}^R} \xrightarrow{\bar{k}_E^R} \mathrm{D_1^R}$	$a_{37} = \bar{k}_E^R n_{12}^R$	$\bar{k}_E^R$	10, 0.5
38	$\mathrm{D_{12}^R} \xrightarrow{\delta} \mathrm{D_1^R}$	$a_{38} = \delta n_{12}^R$	δ	10, 0.5
39	$\mathrm{D_1^R}+\mathrm{D_2^R} \xrightarrow{k_M} \mathrm{D_{12}^R}+\mathrm{D_2^R}$	$a_{39} = \frac{k_M}{\Omega} n_1^R n_2^R$	$\frac{k_M}{\Omega}$	0.2
40	$\mathrm{D_1^R}+\mathrm{D_{12}^R} \xrightarrow{k_M} \mathrm{D_{12}^R}+\mathrm{D_{12}^R}$	$a_{40} = \frac{k_M}{\Omega} n_1^R n_{12}^R$	$\frac{k_M}{\Omega}$	0.2
41	$\mathrm{D_1^R}+\mathrm{D_1^R} \xrightarrow{\bar{k}_M} \mathrm{D_{12}^R}+\mathrm{D_1^R}$	$a_{41} = \frac{\bar{k}_M}{\Omega} \frac{n_1^R(n_1^R-1)}{2}$	$\frac{\bar{k}_M}{\Omega}$	0.2
42	$\mathrm{D_1^R}+\mathrm{D_{12}^R} \xrightarrow{\bar{k}_M} \mathrm{D_{12}^R}+\mathrm{D_{12}^R}$	$a_{42} = \frac{\bar{k}_M}{\Omega} n_1^R n_{12}^R$	$\frac{\bar{k}_M}{\Omega}$	0.2
43	$\mathrm{D_{12}^R}+\mathrm{D^A} \xrightarrow{k_E^R} \mathrm{D_1^R}+\mathrm{D^A}$	$a_{43} = \frac{k_E^R}{\Omega} n_{12}^R n^A$	$\frac{k_E^R}{\Omega}$	1
44	$\mathrm{D_2^R} \xrightarrow{k_{W0}^1} \mathrm{D_{12}^R}$	$a_{44} = k_{W0}^1 n_2^R$	k_{W0}^1	5
45	$\mathrm{D_{12}^R} \xrightarrow{k_T'} \mathrm{D_2^R}$	$a_{45} = k_T' n_{12}^R$	k_T'	1, 0.05
46	$\mathrm{D_{12}^R} \xrightarrow{\delta'} \mathrm{D_2^R}$	$a_{46} = \delta' n_{12}^R$	δ'	1, 0.05
47	$\mathrm{D_2^R}+\mathrm{D_2^R} \xrightarrow{k_M'} \mathrm{D_{12}^R}+\mathrm{D_2^R}$	$a_{47} = \frac{k_M'}{\Omega} \frac{n_2^R(n_2^R-1)}{2}$	$\frac{k_M'}{\Omega}$	0.2
48	$\mathrm{D_2^R}+\mathrm{D_{12}^R} \xrightarrow{k_M'} \mathrm{D_{12}^R}+\mathrm{D_{12}^R}$	$a_{48} = \frac{k_M'}{\Omega} n_2^R n_{12}^R$	$\frac{k_M'}{\Omega}$	0.2
49	$\mathrm{D_{12}^R}+\mathrm{D^A} \xrightarrow{k_T'^*} \mathrm{D_2^R}+\mathrm{D^A}$	$a_{49} = \frac{k_T'^*}{\Omega} n_{12}^R n^A$	$\frac{k_T'^*}{\Omega}$	0.2
50	$\mathrm{D^A} \xrightarrow{\alpha_x} \mathrm{D^A}+\mathrm{X}$	$a_{50} = \alpha_x n^A$	α_x	1
51	$\mathrm{X} \xrightarrow{\gamma_x} \emptyset$	$a_{51} = \gamma_x n^X$	γ_x	1
52	$\mathrm{C_A} \xrightarrow{\delta} \mathrm{D}+\mathrm{X}$	$a_{52} = \delta n^A$	δ	10, 0.5

Table D: **Reactions and parameter values used to generate the plots in Fig 5F.**

$\mathbf{R}_k$	**Reaction**	**Prop.Func.**(a_k)	**Param.**	**Value** $(\mathbf{h}^{-1})$ Fig 6C	**Value** $(\mathbf{h}^{-1})$ Fig I
1_i	$\mathrm{D^i + i \xrightarrow{a} C^i_A}$	$a_{1_i} = \frac{a}{\Omega} n^{D,i} n^i$	$\frac{a}{\Omega}$	10	10
2_i	$\mathrm{C^i_A \xrightarrow{d} D^i + i}$	$a_{2_i} = d n_A^{C,i}$	d	10	10
3_i	$\mathrm{D^i + j \xrightarrow{a} C^i_R}$	$a_{3_i} = \frac{a}{\Omega} n^{D,i} n^j$	$\frac{a}{\Omega}$	10	10
4_i	$\mathrm{C^i_R \xrightarrow{d} D^i + j}$	$a_{4_i} = d n_R^{C,i}$	d	10	10
5_i	$\mathrm{D^i \xrightarrow{k^A_{W0}} D^{A,i}}$	$a_{5_i} = k^A_{W0} n^{D,i}$	k^A_{W0}	3.5	3.5
6_i	$\mathrm{C^i_A \xrightarrow{k^A_{W0}} D^{A,i} + i}$	$a_{6_i} = k^A_{W0} n_A^{C,i}$	k^A_{W0}	3.5	3.5
7_i	$\mathrm{C^i_R \xrightarrow{k^A_{W0}} D^{A,i} + j}$	$a_{7_i} = k^A_{W0} n_R^{C,i}$	k^A_{W0}	3.5	3.5
8_i	$\mathrm{C^i_A \xrightarrow{k^A_{W}} D^{A,i} + i}$	$a_{8_i} = k^A_{W} n_A^{C,i}$	k^A_W	300	300
9_i	$\mathrm{D^{A,i} \xrightarrow{\bar{k}^A_E} D^i}$	$a_{9_i} = \bar{k}^A_E n^{A,i}$	$\bar{k}^A_E$	12, 5	12
10_i	$\mathrm{D^{A,i} \xrightarrow{\delta} D^i}$	$a_{10_i} = \delta n^{A,i}$	δ	12, 5	12
11_i	$\mathrm{D^i + D^{A,i} \xrightarrow{k^A_M} D^{A,i} + D^{A,i}}$	$a_{11_i} = \frac{k^A_M}{\Omega} n^{D,i} n^{A,i}$	$\frac{k^A_M}{\Omega}$	1	1
12_i	$\mathrm{C^i_A + D^{A,i} \xrightarrow{k^A_M} D^{A,i} + D^{A,i} + i}$	$a_{12_i} = \frac{k^A_M}{\Omega} n_A^{C,i} n^{A,i}$	$\frac{k^A_M}{\Omega}$	1	1
13_i	$\mathrm{C^i_R + D^{A,i} \xrightarrow{k^A_M} D^{A,i} + D^{A,i} + j}$	$a_{13_i} = \frac{k^A_M}{\Omega} n_R^{C,i} n^{A,i}$	$\frac{k^A_M}{\Omega}$	1	1
14_i	$\mathrm{D^{A,i} + D^{R,i}_1 \xrightarrow{k^A_E} D^i + D^{R,i}_1}$	$a_{14_i} = \frac{k^A_E}{\Omega} n^{A,i} n_1^{R,i}$	$\frac{k^A_E}{\Omega}$	1	1
15_i	$\mathrm{D^{A,i} + D^{R,i}_{12} \xrightarrow{k^A_E} D^i + D^{R,i}_{12}}$	$a_{15_i} = \frac{k^A_E}{\Omega} n^{A,i} n_{12}^{R,i}$	$\frac{k^A_E}{\Omega}$	1	1
16_i	$\mathrm{D^{A,i} + D^{R,i}_2 \xrightarrow{k^A_E} D^i + D^{R,i}_2}$	$a_{16_i} = \frac{k^A_E}{\Omega} n^{A,i} n_2^{R,i}$	$\frac{k^A_E}{\Omega}$	1	1
17_i	$\mathrm{D^{A,i} + D^{R,i}_{12} \xrightarrow{k^A_E} D^i + D^{R,i}_{12}}$	$a_{17_i} = \frac{k^A_E}{\Omega} n^{A,i} n_{12}^{R,i}$	$\frac{k^A_E}{\Omega}$	1	1
18_i	$\mathrm{D^i \xrightarrow{k^1_{W0}} D^{R,i}_1}$	$a_{18_i} = k^1_{W0} n^{D,i}$	k^1_{W0}	3.5	3.5
19_i	$\mathrm{C^i_A \xrightarrow{k^1_{W0}} D^{R,i}_1 + i}$	$a_{19_i} = k^1_{W0} n_A^{C,i}$	k^1_{W0}	3.5	3.5
20_i	$\mathrm{C^i_R \xrightarrow{k^1_{W0}} D^{R,i}_1 + j}$	$a_{20_i} = k^1_{W0} n_R^{C,i}$	k^1_{W0}	3.5	3.5
21_i	$\mathrm{C^i_R \xrightarrow{k^1_{W}} D^{R,i}_1 + j}$	$a_{21_i} = k^1_{W} n_R^{C,i}$	k^1_W	300	300
22_i	$\mathrm{D^{R,i}_1 \xrightarrow{k'_T} D^i}$	$a_{22_i} = k'_T n_1^{R,i}$	k'_T	7.2, 3	7.2
23_i	$\mathrm{D^{R,i}_1 \xrightarrow{\delta'} D^i}$	$a_{23_i} = \delta' n_1^{R,i}$	δ'	7.2, 3	7.2
24_i	$\mathrm{D^i + D^{R,i}_2 \xrightarrow{k'_M} D^{R,i}_1 + D^{R,i}_2}$	$a_{24_i} = \frac{k'_M}{\Omega} n^{D,i} n_2^{R,i}$	$\frac{k'_M}{\Omega}$	0.2	0.2
25_i	$\mathrm{C^i_R + D^{R,i}_2 \xrightarrow{k'_M} D^{R,i}_1 + D^{R,i}_2 + j}$	$a_{25_i} = \frac{k'_M}{\Omega} n_R^{C,i} n_2^{R,i}$	$\frac{k'_M}{\Omega}$	0.2	0.2
26_i	$\mathrm{C^i_A + D^{R,i}_2 \xrightarrow{k'_M} D^{R,i}_1 + D^{R,i}_2 + i}$	$a_{26_i} = \frac{k'_M}{\Omega} n_A^{C,i} n_2^{R,i}$	$\frac{k'_M}{\Omega}$	0.2	0.2
27_i	$\mathrm{D^i + D^{R,i}_{12} \xrightarrow{k'_M} D^{R,i}_1 + D^{R,i}_{12}}$	$a_{27_i} = \frac{k'_M}{\Omega} n^{D,i} n_{12}^{R,i}$	$\frac{k'_M}{\Omega}$	0.2	0.2
28_i	$\mathrm{C^i_R + D^{R,i}_{12} \xrightarrow{k'_M} D^{R,i}_1 + D^{R,i}_{12} + j}$	$a_{28_i} = \frac{k'_M}{\Omega} n_R^{C,i} n_{12}^{R,i}$	$\frac{k'_M}{\Omega}$	0.2	0.2
29_i	$\mathrm{C^i_A + D^{R,i}_{12} \xrightarrow{k'_M} D^{R,i}_1 + D^{R,i}_{12} + i}$	$a_{29_i} = \frac{k'_M}{\Omega} n_A^{C,i} n_{12}^{R,i}$	$\frac{k'_M}{\Omega}$	0.2	0.2
30_i	$\mathrm{D^{R,i}_1 + D^{A,i} \xrightarrow{k'^*_T} D^i + D^{A,i}}$	$a_{30_i} = \frac{k'^*_T}{\Omega} n_1^{R,i} n^{A,i}$	$\frac{k'^*_T}{\Omega}$	0.6	0.6
31_i	$\mathrm{D^i \xrightarrow{k^2_{W0}} D^{R,i}_2}$	$a_{31_i} = k^2_{W0} n^{D,i}$	k^2_{W0}	3.5	3.5
32_i	$\mathrm{C^i_A \xrightarrow{k^2_{W0}} D^{R,i}_2 + i}$	$a_{32_i} = k^2_{W0} n_A^{C,i}$	k^2_{W0}	3.5	3.5
33_i	$\mathrm{C^i_R \xrightarrow{k^2_{W0}} D^{R,i}_2 + j}$	$a_{33_i} = k^2_{W0} n_R^{C,i}$	k^2_{W0}	3.5	3.5
34_i	$\mathrm{C^i_R \xrightarrow{k^2_{W}} D^{R,i}_2 + j}$	$a_{34_i} = k^2_{W} n_R^{C,i}$	k^2_W	300	300
35_i	$\mathrm{D^{R,i}_2 \xrightarrow{\bar{k}^R_E} D^i}$	$a_{35_i} = \bar{k}^R_E n_2^{R,i}$	$\bar{k}^R_E$	12, 5	12
36_i	$\mathrm{D^{R,i}_2 \xrightarrow{\delta} D^i}$	$a_{36_i} = \delta n_2^{R,i}$	δ	12, 5	12
37_i	$\mathrm{D^i + D^{R,i}_2 \xrightarrow{k_M} D^{R,i}_2 + D^{R,i}_2}$	$a_{37_i} = \frac{k_M}{\Omega} n^{D,i} n_2^{R,i}$	$\frac{k_M}{\Omega}$	0.2	0.2
38_i	$\mathrm{C^i_R + D^{R,i}_2 \xrightarrow{k_M} D^{R,i}_2 + D^{R,i}_2 + j}$	$a_{38_i} = \frac{k_M}{\Omega} n_R^{C,i} n_2^{R,i}$	$\frac{k_M}{\Omega}$	0.2	0.2
39_i	$\mathrm{C^i_A + D^{R,i}_2 \xrightarrow{k_M} D^{R,i}_2 + D^{R,i}_2 + i}$	$a_{39_i} = \frac{k_M}{\Omega} n_A^{C,i} n_2^{R,i}$	$\frac{k_M}{\Omega}$	0.2	0.2
40_i	$\mathrm{D^i + D^{R,i}_{12} \xrightarrow{k_M} D^{R,i}_2 + D^{R,i}_{12}}$	$a_{40_i} = \frac{k_M}{\Omega} n^{D,i} n_{12}^{R,i}$	$\frac{k_M}{\Omega}$	0.2	0.2
41_i	$\mathrm{C^i_R + D^{R,i}_{12} \xrightarrow{k_M} D^{R,i}_2 + D^{R,i}_{12} + j}$	$a_{41_i} = \frac{k_M}{\Omega} n_R^{C,i} n_{12}^{R,i}$	$\frac{k_M}{\Omega}$	0.2	0.2
42_i	$\mathrm{C^i_A + D^{R,i}_{12} \xrightarrow{k_M} D^{R,i}_2 + D^{R,i}_{12} + i}$	$a_{42_i} = \frac{k_M}{\Omega} n_A^{C,i} n_{12}^{R,i}$	$\frac{k_M}{\Omega}$	0.2	0.2
43_i	$\mathrm{D^i + D^{R,i}_1 \xrightarrow{\bar{k}_M} D^{R,i}_2 + D^{R,i}_1}$	$a_{43_i} = \frac{\bar{k}_M}{\Omega} n^{D,i} n_1^{R,i}$	$\frac{\bar{k}_M}{\Omega}$	0.2	0.2
44_i	$\mathrm{C^i_R + D^{R,i}_1 \xrightarrow{\bar{k}_M} D^{R,i}_2 + D^{R,i}_1 + j}$	$a_{44_i} = \frac{\bar{k}_M}{\Omega} n_R^{C,i} n_1^{R,i}$	$\frac{\bar{k}_M}{\Omega}$	0.2	0.2

Table E: **Reactions and parameter values used to generate the plots in Fig 6C and Fig I, with $i, j = X, Z$ and $i \neq j$.**

R_k	**Reaction**	**Prop.Func.**(a_k)	**Param.**	**Value** (h^{-1}) Fig 6C	**Value** (h^{-1}) Fig I
45_i	$C^{i}_{A} + D^{R,i}_{1} \xrightarrow{\bar{k}_M} D^{R,i}_{2} + D^{R,i}_{1} + i$	$a_{45_i} = \frac{\bar{k}_M}{\Omega} n^{C,i}_{A} n^{R,i}_{1}$	$\frac{\bar{k}_M}{\Omega}$	0.2	0.2
46_i	$D^{i} + D^{R,i}_{12} \xrightarrow{\bar{k}_M} D^{R,i}_{2} + D^{R,i}_{12}$	$a_{46_i} = \frac{\bar{k}_M}{\Omega} n^{D,i} n^{R,i}_{12}$	$\frac{\bar{k}_M}{\Omega}$	0.2	0.2
47_i	$C^{i}_{R} + D^{R,i}_{12} \xrightarrow{\bar{k}_M} D^{R,i}_{2} + D^{R,i}_{12} + j$	$a_{47_i} = \frac{\bar{k}_M}{\Omega} n^{C,i}_{R} n^{R,i}_{12}$	$\frac{\bar{k}_M}{\Omega}$	0.2	0.2
48_i	$C^{i}_{A} + D^{R,i}_{12} \xrightarrow{\bar{k}_M} D^{R,i}_{2} + D^{R,i}_{12} + i$	$a_{48_i} = \frac{\bar{k}_M}{\Omega} n^{C,i}_{A} n^{R,i}_{12}$	$\frac{\bar{k}_M}{\Omega}$	0.2	0.2
49_i	$D^{R,i}_{2} + D^{A,i} \xrightarrow{k^R_E} D^{i} + D^{A,i}$	$a_{49_i} = \frac{k^R_E}{\Omega} n^{R,i}_{2} n^{A,i}$	$\frac{k^R_E}{\Omega}$	1	1
50_i	$D^{R,i}_{1} \xrightarrow{k^2_{W0}} D^{R,i}_{12}$	$a_{50_i} = k^2_{W0} n^{R,i}_{1}$	k^2_{W0}	3.5	3.5
51_i	$D^{R,i}_{12} \xrightarrow{\bar{k}^R_E} D^{R,i}_{1}$	$a_{51_i} = \bar{k}^R_E n^{R,i}_{12}$	$\bar{k}^R_E$	12, 5	12
52_i	$D^{R,i}_{12} \xrightarrow{\delta} D^{R,i}_{1}$	$a_{52_i} = \delta n^{R,i}_{12}$	δ	12, 5	12
53_i	$D^{R,i}_{1} + D^{R,i}_{2} \xrightarrow{k_M} D^{R,i}_{12} + D^{R,i}_{2}$	$a_{53_i} = \frac{k_M}{\Omega} n^{R,i}_{1} n^{R,i}_{2}$	$\frac{k_M}{\Omega}$	0.2	0.2
54_i	$D^{R,i}_{1} + D^{R,i}_{12} \xrightarrow{k_M} D^{R,i}_{12} + D^{R,i}_{12}$	$a_{54_i} = \frac{k_M}{\Omega} n^{R,i}_{1} n^{R,i}_{12}$	$\frac{k_M}{\Omega}$	0.2	0.2
55_i	$D^{R,i}_{1} + D^{R,i}_{1} \xrightarrow{\bar{k}_M} D^{R,i}_{12} + D^{R,i}_{1}$	$a_{55_i} = \frac{\bar{k}_M}{\Omega} \frac{n^{R,i}_{1}(n^{R,i}_{1}-1)}{2}$	$\frac{\bar{k}_M}{\Omega}$	0.2	0.2
56_i	$D^{R,i}_{1} + D^{R,i}_{12} \xrightarrow{\bar{k}_M} D^{R,i}_{12} + D^{R,i}_{12}$	$a_{56_i} = \frac{\bar{k}_M}{\Omega} n^{R,i}_{1} n^{R,i}_{12}$	$\frac{\bar{k}_M}{\Omega}$	0.2	0.2
57_i	$D^{R,i}_{12} + D^{A,i} \xrightarrow{k^R_E} D^{R,i}_{1} + D^{A,i}$	$a_{57_i} = \frac{k^R_E}{\Omega} n^{R,i}_{12} n^{A,i}$	$\frac{k^R_E}{\Omega}$	1	1
58_i	$D^{R,i}_{2} \xrightarrow{k^1_{W0}} D^{R,i}_{12}$	$a_{58_i} = k^1_{W0} n^{R,i}_{2}$	k^1_{W0}	3.5	3.5
59_i	$D^{R,i}_{12} \xrightarrow{k'_T} D^{R,i}_{2}$	$a_{59_i} = k'_T n^{R,i}_{12}$	k'_T	7.2, 3	7.2
60_i	$D^{R,i}_{12} \xrightarrow{\delta'} D^{R,i}_{2}$	$a_{60_i} = \delta' n^{R,i}_{12}$	δ'	7.2, 3	7.2
61_i	$D^{R,i}_{2} + D^{R,i}_{2} \xrightarrow{k'_M} D^{R,i}_{12} + D^{R,i}_{2}$	$a_{61_i} = \frac{k'_M}{\Omega} \frac{n^{R,i}_{2}(n^{R,i}_{2}-1)}{2}$	$\frac{k'_M}{\Omega}$	0.2	0.2
62_i	$D^{R,i}_{2} + D^{R,i}_{12} \xrightarrow{k'_M} D^{R,i}_{12} + D^{R,i}_{12}$	$a_{62_i} = \frac{k'_M}{\Omega} n^{R,i}_{2} n^{R,i}_{12}$	$\frac{k'_M}{\Omega}$	0.2	0.2
63_i	$D^{R,i}_{12} + D^{A,i} \xrightarrow{k'^{*}_T} D^{R,i}_{2} + D^{A,i}$	$a_{63_i} = \frac{k'^{*}_T}{\Omega} n^{R}_{12} n^{A,i}$	$\frac{k'^{*}_T}{\Omega}$	0.6	0.6
64_i	$D^{A,i} \xrightarrow{\alpha_i} D^{A,i} + i$	$a_{64_i} = \alpha_i n^{A,i}$	α_i	0, 0.1, 10	0, 10
65_i	$i \xrightarrow{\gamma_i} \emptyset$	$a_{65_i} = \gamma_i n^{i}$	γ_i	1	1
66_i	$C^{i}_{A} \xrightarrow{\delta} D^{i} + i$	$a_{66_i} = \delta n^{C,i}_{A}$	δ	12, 5	12
67_i	$C^{i}_{R} \xrightarrow{\delta} D^{i} + j$	$a_{67_i} = \delta n^{C,i}_{R}$	δ	12, 5	12

Table F: **Reactions and parameter values used to generate the plots in Fig 6C and in Fig I, with $i, j = X, Z$ and $i \neq j$.**

R_k	**Reaction**	**Prop.Func.**(a_k)	**Param.**	**Value** (h^{-1})
1_i	$D^i + i \xrightarrow{a} C^i_A$	$a_{1_i} = \frac{a}{\Omega} n^{D,i} n^i$	$\frac{a}{\Omega}$	10
2_i	$C^i_A \xrightarrow{d} D^i + i$	$a_{2_i} = d n^{C,i}_A$	d	10
3_i	$D^i + j \xrightarrow{a} C^i_R$	$a_{3_i} = \frac{a}{\Omega} n^{D,i} n^j$	$\frac{a}{\Omega}$	10
4_i	$C^i_R \xrightarrow{d} D^i + j$	$a_{4_i} = d n^{C,i}_R$	d	10
5_i	$D^i \xrightarrow{k^A_{W0}} D^{A,i}$	$a_{5_i} = k^A_{W0} n^{D,i}$	k^A_{W0}	3.5
6_i	$C^i_A \xrightarrow{k^A_{W0}} D^{A,i} + i$	$a_{6_i} = k^A_{W0} n^{C,i}_A$	k^A_{W0}	3.5
7_i	$C^i_R \xrightarrow{k^A_{W0}} D^{A,i} + j$	$a_{7_i} = k^A_{W0} n^{C,i}_R$	k^A_{W0}	3.5
8_i	$C^i_A \xrightarrow{k^A_W} D^{A,i} + i$	$a_{8_i} = k^A_W n^{C,i}_A$	k^A_W	300
9_i	$D^{A,i} \xrightarrow{\bar{k}^A_E} D^i$	$a_{9_i} = \bar{k}^A_E n^{A,i}$	$\bar{k}^A_E$	17.5, 1.75
10_i	$D^{A,i} \xrightarrow{\delta} D^i$	$a_{10_i} = \delta n^{A,i}$	δ	17.5, 1.75
11_i	$D^i + D^{A,i} \xrightarrow{k^A_M} D^{A,i} + D^{A,i}$	$a_{11_i} = \frac{k^A_M}{\Omega} n^{D,i} n^{A,i}$	$\frac{k^A_M}{\Omega}$	1
12_i	$C^i_A + D^{A,i} \xrightarrow{k^A_M} D^{A,i} + D^{A,i} + i$	$a_{12_i} = \frac{k^A_M}{\Omega} n^{C,i}_A n^{A,i}$	$\frac{k^A_M}{\Omega}$	1
13_i	$C^i_R + D^{A,i} \xrightarrow{k^A_M} D^{A,i} + D^{A,i} + j$	$a_{13_i} = \frac{k^A_M}{\Omega} n^{C,i}_R n^{A,i}$	$\frac{k^A_M}{\Omega}$	1
14_i	$D^{A,i} + D^{R,i}_1 \xrightarrow{k^A_E} D^i + D^{R,i}_1$	$a_{14_i} = \frac{k^A_E}{\Omega} n^{A,i} n^{R,i}_1$	$\frac{k^A_E}{\Omega}$	1
15_i	$D^{A,i} + D^{R,i}_{12} \xrightarrow{k^A_E} D^i + D^{R,i}_{12}$	$a_{15_i} = \frac{k^A_E}{\Omega} n^{A,i} n^{R,i}_{12}$	$\frac{k^A_E}{\Omega}$	1
16_i	$D^{A,i} + D^{R,i}_2 \xrightarrow{k^A_E} D^i + D^{R,i}_2$	$a_{16_i} = \frac{k^A_E}{\Omega} n^{A,i} n^{R,i}_2$	$\frac{k^A_E}{\Omega}$	1
17_i	$D^{A,i} + D^{R,i}_{12} \xrightarrow{k^A_E} D^i + D^{R,i}_{12}$	$a_{17_i} = \frac{k^A_E}{\Omega} n^{A,i} n^{R,i}_{12}$	$\frac{k^A_E}{\Omega}$	1
18_i	$D^i \xrightarrow{k^1_{W0}} D^{R,i}_1$	$a_{18_i} = k^1_{W0} n^{D,i}$	k^1_{W0}	3.5
19_i	$C^i_A \xrightarrow{k^1_{W0}} D^{R,i}_1 + i$	$a_{19_i} = k^1_{W0} n^{C,i}_A$	k^1_{W0}	3.5
20_i	$C^i_R \xrightarrow{k^1_{W0}} D^{R,i}_1 + j$	$a_{20_i} = k^1_{W0} n^{C,i}_R$	k^1_{W0}	3.5
21_i	$C^i_R \xrightarrow{k^1_W} D^{R,i}_1 + j$	$a_{21_i} = k^1_W n^{C,i}_R$	k^1_W	300
22_i	$D^{R,i}_1 \xrightarrow{k'_T} D^i$	$a_{22_i} = k'_T n^{R,i}_1$	k'_T	10.5, 1.05
23_i	$D^{R,i}_1 \xrightarrow{\delta'} D^i$	$a_{23_i} = \delta' n^{R,i}_1$	δ'	10.5, 1.05
24_i	$D^i + D^{R,i}_2 \xrightarrow{k'_M} D^{R,i}_1 + D^{R,i}_2$	$a_{24_i} = \frac{k'_M}{\Omega} n^{D,i} n^{R,i}_2$	$\frac{k'_M}{\Omega}$	0.2
25_i	$C^i_R + D^{R,i}_2 \xrightarrow{k'_M} D^{R,i}_1 + D^{R,i}_2 + j$	$a_{25_i} = \frac{k'_M}{\Omega} n^{C,i}_R n^{R,i}_2$	$\frac{k'_M}{\Omega}$	0.2
26_i	$C^i_A + D^{R,i}_2 \xrightarrow{k'_M} D^{R,i}_1 + D^{R,i}_2 + i$	$a_{26_i} = \frac{k'_M}{\Omega} n^{C,i}_A n^{R,i}_2$	$\frac{k'_M}{\Omega}$	0.2
27_i	$D^i + D^{R,i}_{12} \xrightarrow{k'_M} D^{R,i}_1 + D^{R,i}_{12}$	$a_{27_i} = \frac{k'_M}{\Omega} n^{D,i} n^{R,i}_{12}$	$\frac{k'_M}{\Omega}$	0.2
28_i	$C^i_R + D^{R,i}_{12} \xrightarrow{k'_M} D^{R,i}_1 + D^{R,i}_{12} + j$	$a_{28_i} = \frac{k'_M}{\Omega} n^{C,i}_R n^{R,i}_{12}$	$\frac{k'_M}{\Omega}$	0.2
29_i	$C^i_A + D^{R,i}_{12} \xrightarrow{k'_M} D^{R,i}_1 + D^{R,i}_{12} + i$	$a_{29_i} = \frac{k'_M}{\Omega} n^{C,i}_A n^{R,i}_{12}$	$\frac{k'_M}{\Omega}$	0.2
30_i	$D^{R,i}_1 + D^{A,i} \xrightarrow{k'^*_T} D^i + D^{A,i}$	$a_{30_i} = \frac{k'^*_T}{\Omega} n^{R,i}_1 n^{A,i}$	$\frac{k'^*_T}{\Omega}$	0.6
31_i	$D^i \xrightarrow{k^2_{W0}} D^{R,i}_2$	$a_{31_i} = k^2_{W0} n^{D,i}$	k^2_{W0}	3.5
32_i	$C^i_A \xrightarrow{k^2_{W0}} D^{R,i}_2 + i$	$a_{32_i} = k^2_{W0} n^{C,i}_A$	k^2_{W0}	3.5
33_i	$C^i_R \xrightarrow{k^2_{W0}} D^{R,i}_2 + j$	$a_{33_i} = k^2_{W0} n^{C,i}_R$	k^2_{W0}	3.5
34_i	$C^i_R \xrightarrow{k^2_W} D^{R,i}_2 + j$	$a_{34_i} = k^2_W n^{C,i}_R$	k^2_W	300
35_i	$D^{R,i}_2 \xrightarrow{\bar{k}^R_E} D^i$	$a_{35_i} = \bar{k}^R_E n^{R,i}_2$	$\bar{k}^R_E$	17.5, 1.75
36_i	$D^{R,i}_2 \xrightarrow{\delta} D^i$	$a_{36_i} = \delta n^{R,i}_2$	δ	17.5, 1.75
37_i	$D^i + D^{R,i}_2 \xrightarrow{k_M} D^{R,i}_2 + D^{R,i}_2$	$a_{37_i} = \frac{k_M}{\Omega} n^{D,i} n^{R,i}_2$	$\frac{k_M}{\Omega}$	0.2
38_i	$C^i_R + D^{R,i}_2 \xrightarrow{k_M} D^{R,i}_2 + D^{R,i}_2 + j$	$a_{38_i} = \frac{k_M}{\Omega} n^{C,i}_R n^{R,i}_2$	$\frac{k_M}{\Omega}$	0.2
39_i	$C^i_A + D^{R,i}_2 \xrightarrow{k_M} D^{R,i}_2 + D^{R,i}_2 + i$	$a_{39_i} = \frac{k_M}{\Omega} n^{C,i}_A n^{R,i}_2$	$\frac{k_M}{\Omega}$	0.2
40_i	$D^i + D^{R,i}_{12} \xrightarrow{k_M} D^{R,i}_2 + D^{R,i}_{12}$	$a_{40_i} = \frac{k_M}{\Omega} n^{D,i} n^{R,i}_{12}$	$\frac{k_M}{\Omega}$	0.2
41_i	$C^i_R + D^{R,i}_{12} \xrightarrow{k_M} D^{R,i}_2 + D^{R,i}_{12} + j$	$a_{41_i} = \frac{k_M}{\Omega} n^{C,i}_R n^{R,i}_{12}$	$\frac{k_M}{\Omega}$	0.2
42_i	$C^i_A + D^{R,i}_{12} \xrightarrow{k_M} D^{R,i}_2 + D^{R,i}_{12} + i$	$a_{42_i} = \frac{k_M}{\Omega} n^{C,i}_A n^{R,i}_{12}$	$\frac{k_M}{\Omega}$	0.2
43_i	$D^i + D^{R,i}_1 \xrightarrow{\bar{k}_M} D^{R,i}_2 + D^{R,i}_1$	$a_{43_i} = \frac{\bar{k}_M}{\Omega} n^{D,i} n^{R,i}_1$	$\frac{\bar{k}_M}{\Omega}$	0.2
44_i	$C^i_R + D^{R,i}_1 \xrightarrow{\bar{k}_M} D^{R,i}_2 + D^{R,i}_1 + j$	$a_{44_i} = \frac{\bar{k}_M}{\Omega} n^{C,i}_R n^{R,i}_1$	$\frac{\bar{k}_M}{\Omega}$	0.2

Table G: **Reactions and parameter values used to generate the plots in Fig 6D, with $i, j = X, Z$ and $i \neq j$. These reactions and parameter values (with $\bar{k}^A_E = \bar{k}^R_E = \delta = 1.75, 7.5h^{-1}$ and $k'_T = \delta' = 1.05, 4.5h^{-1}$) are used also for simulations in Fig J.**

**R**$_k$	**Reaction**	**Prop.Func.**(a_k)	**Param.**	**Value (h**$^{-1}$**)**
45_i	$\mathrm{C^i_A} + \mathrm{D^{R,i}_1} \xrightarrow{\bar{k}_M} \mathrm{D^{R,i}_2} + \mathrm{D^{R,i}_1} + \mathrm{i}$	$a_{45_i} = \frac{\bar{k}_M}{\Omega} n^{C,i}_A n^{R,i}_1$	$\frac{\bar{k}_M}{\Omega}$	0.2
46_i	$\mathrm{D^i} + \mathrm{D^{R,i}_{12}} \xrightarrow{\bar{k}_M} \mathrm{D^{R,i}_2} + \mathrm{D^{R,i}_{12}}$	$a_{46_i} = \frac{\bar{k}_M}{\Omega} n^{D,i} n^{R,i}_{12}$	$\frac{\bar{k}_M}{\Omega}$	0.2
47_i	$\mathrm{C^i_R} + \mathrm{D^{R,i}_{12}} \xrightarrow{\bar{k}_M} \mathrm{D^{R,i}_2} + \mathrm{D^{R,i}_{12}} + \mathrm{j}$	$a_{47_i} = \frac{\bar{k}_M}{\Omega} n^{C,i}_R n^{R,i}_{12}$	$\frac{\bar{k}_M}{\Omega}$	0.2
48_i	$\mathrm{C^i_A} + \mathrm{D^{R,i}_{12}} \xrightarrow{\bar{k}_M} \mathrm{D^{R,i}_2} + \mathrm{D^{R,i}_{12}} + \mathrm{i}$	$a_{48_i} = \frac{\bar{k}_M}{\Omega} n^{C,i}_A n^{R,i}_{12}$	$\frac{\bar{k}_M}{\Omega}$	0.2
49_i	$\mathrm{D^{R,i}_2} + \mathrm{D^{A,i}} \xrightarrow{k^R_E} \mathrm{D^i} + \mathrm{D^{A,i}}$	$a_{49_i} = \frac{k^R_E}{\Omega} n^{R,i}_2 n^{A,i}$	$\frac{k^R_E}{\Omega}$	1
50_i	$\mathrm{D^{R,i}_1} \xrightarrow{k^2_{W0}} \mathrm{D^{R,i}_{12}}$	$a_{50_i} = k^2_{W0} n^{R,i}_1$	k^2_{W0}	3.5
51_i	$\mathrm{D^{R,i}_{12}} \xrightarrow{\bar{k}^R_E} \mathrm{D^{R,i}_1}$	$a_{51_i} = \bar{k}^R_E n^{R,i}_{12}$	$\bar{k}^R_E$	17.5, 1.75
52_i	$\mathrm{D^{R,i}_{12}} \xrightarrow{\delta} \mathrm{D^{R,i}_1}$	$a_{52_i} = \delta n^{R,i}_{12}$	δ	17.5, 1.75
53_i	$\mathrm{D^{R,i}_1} + \mathrm{D^{R,i}_2} \xrightarrow{k_M} \mathrm{D^{R,i}_{12}} + \mathrm{D^{R,i}_2}$	$a_{53_i} = \frac{k_M}{\Omega} n^{R,i}_1 n^{R,i}_2$	$\frac{k_M}{\Omega}$	0.2
54_i	$\mathrm{D^{R,i}_1} + \mathrm{D^{R,i}_{12}} \xrightarrow{k_M} \mathrm{D^{R,i}_{12}} + \mathrm{D^{R,i}_{12}}$	$a_{54_i} = \frac{k_M}{\Omega} n^{R,i}_1 n^{R,i}_{12}$	$\frac{k_M}{\Omega}$	0.2
55_i	$\mathrm{D^{R,i}_1} + \mathrm{D^{R,i}_1} \xrightarrow{\bar{k}_M} \mathrm{D^{R,i}_{12}} + \mathrm{D^{R,i}_1}$	$a_{55_i} = \frac{\bar{k}_M}{\Omega} \frac{n^{R,i}_1 (n^{R,i}_1 - 1)}{2}$	$\frac{\bar{k}_M}{\Omega}$	0.2
56_i	$\mathrm{D^{R,i}_1} + \mathrm{D^{R,i}_{12}} \xrightarrow{\bar{k}_M} \mathrm{D^{R,i}_{12}} + \mathrm{D^{R,i}_{12}}$	$a_{56_i} = \frac{\bar{k}_M}{\Omega} n^{R,i}_1 n^{R,i}_{12}$	$\frac{\bar{k}_M}{\Omega}$	0.2
57_i	$\mathrm{D^{R,i}_{12}} + \mathrm{D^{A,i}} \xrightarrow{k^R_E} \mathrm{D^{R,i}_1} + \mathrm{D^{A,i}}$	$a_{57_i} = \frac{k^R_E}{\Omega} n^{R,i}_{12} n^{A,i}$	$\frac{k^R_E}{\Omega}$	1
58_i	$\mathrm{D^{R,i}_2} \xrightarrow{k^1_{W0}} \mathrm{D^{R,i}_{12}}$	$a_{58_i} = k^1_{W0} n^{R,i}_2$	k^1_{W0}	3.5
59_i	$\mathrm{D^{R,i}_{12}} \xrightarrow{k'_T} \mathrm{D^{R,i}_2}$	$a_{59_i} = k'_T n^{R,i}_{12}$	k'_T	10.5, 1.05
60_i	$\mathrm{D^{R,i}_{12}} \xrightarrow{\delta'} \mathrm{D^{R,i}_2}$	$a_{60_i} = \delta' n^{R,i}_{12}$	δ'	10.5, 1.05
61_i	$\mathrm{D^{R,i}_2} + \mathrm{D^{R,i}_2} \xrightarrow{k'_M} \mathrm{D^{R,i}_{12}} + \mathrm{D^{R,i}_2}$	$a_{61_i} = \frac{k'_M}{\Omega} \frac{n^{R,i}_2 (n^{R,i}_2 - 1)}{2}$	$\frac{k'_M}{\Omega}$	0.2
62_i	$\mathrm{D^{R,i}_2} + \mathrm{D^{R,i}_{12}} \xrightarrow{k'_M} \mathrm{D^{R,i}_{12}} + \mathrm{D^{R,i}_{12}}$	$a_{62_i} = \frac{k'_M}{\Omega} n^{R,i}_2 n^{R,i}_{12}$	$\frac{k'_M}{\Omega}$	0.2
63_i	$\mathrm{D^{R,i}_{12}} + \mathrm{D^{A,i}} \xrightarrow{k'^*_T} \mathrm{D^{R,i}_2} + \mathrm{D^{A,i}}$	$a_{63_i} = \frac{k'^*_T}{\Omega} n^R_{12} n^{A,i}$	$\frac{k'^*_T}{\Omega}$	0.6
64_i	$\mathrm{D^{A,i}} \xrightarrow{\alpha_i} \mathrm{D^{A,i}} + \mathrm{i}$	$a_{64_i} = \alpha_i n^{A,i}$	α_i	0.15
65_i	$\mathrm{i} \xrightarrow{\gamma_i} \emptyset$	$a_{65_i} = \gamma_i n^i$	γ_i	1
66_i	$\mathrm{C^i_A} \xrightarrow{\delta} \mathrm{D^i} + \mathrm{i}$	$a_{66_i} = \delta n^{C,i}_A$	δ	17.5, 1.75
67_i	$\mathrm{C^i_R} \xrightarrow{\delta} \mathrm{D^i} + \mathrm{j}$	$a_{67_i} = \delta n^{C,i}_R$	δ	17.5, 1.75

Table H: **Reactions and parameter values used to generate the plots in Fig 6D, with $i, j = X, Z$ and $i \neq j$. These reactions and parameter values (with $\bar{k}^A_E = \bar{k}^R_E = \delta = 1.75, 7.5h^{-1}$ and $k'_T = \delta' = 1.05, 4.5h^{-1}$) are used also for simulations in Fig J.**

$\mathbf{R}_j$	**Reaction**	**Prop.Func.**(a_j)	**Param.**	**Value** ($\mathbf{h}^{-1}$) left plots	**Value** ($\mathbf{h}^{-1}$) right plots
1	$\mathrm{D}+\mathrm{X} \xrightarrow{a} \mathrm{C_A}$	$a_1 = \frac{a}{\Omega} n^D n^X$	$\frac{a}{\Omega}$	10	10
2	$\mathrm{C_A} \xrightarrow{d} \mathrm{D}+\mathrm{X}$	$a_2 = d n_A^C$	d	10	10
3	$\mathrm{D} \xrightarrow{k_{W0}^A} \mathrm{D^A}$	$a_3 = k_{W0}^A n^D$	k_{W0}^A	3.5	3.5
4	$\mathrm{C_A} \xrightarrow{k_{W0}^A} \mathrm{D^A}+\mathrm{X}$	$a_4 = k_{W0}^A n^D$	k_{W0}^A	3.5	3.5
5	$\mathrm{C_A} \xrightarrow{k_W^A} \mathrm{D^A}+\mathrm{X}$	$a_5 = k_W^A n_A^C$	k_W^A	300	300
6	$\mathrm{D^A} \xrightarrow{\bar{k}_E^A} \mathrm{D}$	$a_6 = \bar{k}_E^A n^A$	$\bar{k}_E^A$	3	3
7	$\mathrm{D^A} \xrightarrow{\delta} \mathrm{D}$	$a_7 = \delta n^A$	δ	3	3
8	$\mathrm{D}+\mathrm{D^A} \xrightarrow{k_M^A} \mathrm{D^A}+\mathrm{D^A}$	$a_8 = \frac{k_M^A}{\Omega} n^D n^A$	$\frac{k_M^A}{\Omega}$	1	1
9	$\mathrm{C_A}+\mathrm{D^A} \xrightarrow{k_M^A} \mathrm{D^A}+\mathrm{D^A}+\mathrm{X}$	$a_9 = \frac{k_M^A}{\Omega} n_A^C n^A$	$\frac{k_M^A}{\Omega}$	1	1
10	$\mathrm{D^A}+\mathrm{D_1^R} \xrightarrow{k_E^A} \mathrm{D}+\mathrm{D_1^R}$	$a_{10} = \frac{k_E^A}{\Omega} n^A n_1^R$	$\frac{k_E^A}{\Omega}$	1	1
11	$\mathrm{D^A}+\mathrm{D_{12}^R} \xrightarrow{k_E^A} \mathrm{D}+\mathrm{D_{12}^R}$	$a_{11} = \frac{k_E^A}{\Omega} n^A n_{12}^R$	$\frac{k_E^A}{\Omega}$	1	1
12	$\mathrm{D^A}+\mathrm{D_2^R} \xrightarrow{k_E^A} \mathrm{D}+\mathrm{D_2^R}$	$a_{12} = \frac{k_E^A}{\Omega} n^A n_2^R$	$\frac{k_E^A}{\Omega}$	1	1
13	$\mathrm{D^A}+\mathrm{D_{12}^R} \xrightarrow{k_E^A} \mathrm{D}+\mathrm{D_{12}^R}$	$a_{13} = \frac{k_E^A}{\Omega} n^A n_{12}^R$	$\frac{k_E^A}{\Omega}$	1	1
14	$\mathrm{D} \xrightarrow{k_{W0}^1} \mathrm{D_1^R}$	$a_{14} = k_{W0}^1 n^D$	k_{W0}^1	3.5	3.5
15	$\mathrm{C_A} \xrightarrow{k_{W0}^1} \mathrm{D_1^R}+\mathrm{X}$	$a_{15} = k_{W0}^1 n^D$	k_{W0}^1	3.5	3.5
16	$\mathrm{D_1^R} \xrightarrow{k_T^{'}} \mathrm{D}$	$a_{16} = k_T^{'} n_1^R$	$k_T^{'}$	3, 1.5	3
17	$\mathrm{D_1^R} \xrightarrow{\delta^{'}} \mathrm{D}$	$a_{17} = \delta^{'} n_1^R$	$\delta^{'}$	3, 1.5	3
18	$\mathrm{D}+\mathrm{D_2^R} \xrightarrow{k_M^{'}} \mathrm{D_1^R}+\mathrm{D_2^R}$	$a_{18} = \frac{k_M^{'}}{\Omega} n^D n_2^R$	$\frac{k_M^{'}}{\Omega}$	0.2	0.2
19	$\mathrm{C_A}+\mathrm{D_2^R} \xrightarrow{k_M^{'}} \mathrm{D_1^R}+\mathrm{D_2^R}+\mathrm{X}$	$a_{19} = \frac{k_M^{'}}{\Omega} n^D n_2^R$	$\frac{k_M^{'}}{\Omega}$	0.2	0.2
20	$\mathrm{D}+\mathrm{D_{12}^R} \xrightarrow{k_M^{'}} \mathrm{D_1^R}+\mathrm{D_{12}^R}$	$a_{20} = \frac{k_M^{'}}{\Omega} n^D n_{12}^R$	$\frac{k_M^{'}}{\Omega}$	0.2	0.2
21	$\mathrm{C_A}+\mathrm{D_{12}^R} \xrightarrow{k_M^{'}} \mathrm{D_1^R}+\mathrm{D_{12}^R}+\mathrm{X}$	$a_{21} = \frac{k_M^{'}}{\Omega} n^D n_{12}^R$	$\frac{k_M^{'}}{\Omega}$	0.2	0.2
22	$\mathrm{D_1^R}+\mathrm{D^A} \xrightarrow{k_T^{'*}} \mathrm{D}+\mathrm{D^A}$	$a_{22} = \frac{k_T^{'*}}{\Omega} n_1^R n^A$	$\frac{k_T^{'*}}{\Omega}$	1, 0.5	1
23	$\mathrm{D} \xrightarrow{k_{W0}^2} \mathrm{D_2^R}$	$a_{23} = k_{W0}^2 n^D$	k_{W0}^2	3.5	3.5
24	$\mathrm{C_A} \xrightarrow{k_{W0}^2} \mathrm{D_2^R}+\mathrm{X}$	$a_{24} = k_{W0}^2 n^D$	k_{W0}^2	3.5	3.5
25	$\mathrm{D_2^R} \xrightarrow{\bar{k}_E^R} \mathrm{D}$	$a_{25} = \bar{k}_E^R n_2^R$	$\bar{k}_E^R$	3	3, 1.5
26	$\mathrm{D_2^R} \xrightarrow{\delta} \mathrm{D}$	$a_{26} = \delta n_2^R$	δ	3	3
27	$\mathrm{D}+\mathrm{D_2^R} \xrightarrow{k_M} \mathrm{D_2^R}+\mathrm{D_2^R}$	$a_{27} = \frac{k_M}{\Omega} n^D n_2^R$	$\frac{k_M}{\Omega}$	0.2	0.2
28	$\mathrm{C_A}+\mathrm{D_2^R} \xrightarrow{k_M} \mathrm{D_2^R}+\mathrm{D_2^R}+\mathrm{X}$	$a_{28} = \frac{k_M}{\Omega} n^D n_2^R$	$\frac{k_M}{\Omega}$	0.2	0.2
29	$\mathrm{D}+\mathrm{D_{12}^R} \xrightarrow{k_M} \mathrm{D_2^R}+\mathrm{D_{12}^R}$	$a_{29} = \frac{k_M}{\Omega} n^D n_{12}^R$	$\frac{k_M}{\Omega}$	0.2	0.2
30	$\mathrm{C_A}+\mathrm{D_{12}^R} \xrightarrow{k_M} \mathrm{D_2^R}+\mathrm{D_{12}^R}+\mathrm{X}$	$a_{30} = \frac{k_M}{\Omega} n^D n_{12}^R$	$\frac{k_M}{\Omega}$	0.2	0.2
31	$\mathrm{D}+\mathrm{D_1^R} \xrightarrow{\bar{k}_M} \mathrm{D_2^R}+\mathrm{D_1^R}$	$a_{31} = \frac{\bar{k}_M}{\Omega} n^D n_1^R$	$\frac{\bar{k}_M}{\Omega}$	0.2	0.2
32	$\mathrm{C_A}+\mathrm{D_1^R} \xrightarrow{\bar{k}_M} \mathrm{D_2^R}+\mathrm{D_1^R}+\mathrm{X}$	$a_{32} = \frac{\bar{k}_M}{\Omega} n^D n_1^R$	$\frac{\bar{k}_M}{\Omega}$	0.2	0.2
33	$\mathrm{D}+\mathrm{D_{12}^R} \xrightarrow{\bar{k}_M} \mathrm{D_2^R}+\mathrm{D_{12}^R}$	$a_{33} = \frac{\bar{k}_M}{\Omega} n^D n_{12}^R$	$\frac{\bar{k}_M}{\Omega}$	0.2	0.2
34	$\mathrm{C_A}+\mathrm{D_{12}^R} \xrightarrow{\bar{k}_M} \mathrm{D_2^R}+\mathrm{D_{12}^R}+\mathrm{X}$	$a_{34} = \frac{\bar{k}_M}{\Omega} n^D n_{12}^R$	$\frac{\bar{k}_M}{\Omega}$	0.2	0.2
35	$\mathrm{D_2^R}+\mathrm{D^A} \xrightarrow{k_E^R} \mathrm{D}+\mathrm{D^A}$	$a_{35} = \frac{k_E^R}{\Omega} n_2^R n^A$	$\frac{k_E^R}{\Omega}$	1	1, 0.5
36	$\mathrm{D_1^R} \xrightarrow{k_{W0}^2} \mathrm{D_{12}^R}$	$a_{36} = k_{W0}^2 n_1^R$	k_{W0}^2	3.5	3.5
37	$\mathrm{D_{12}^R} \xrightarrow{\bar{k}_E^R} \mathrm{D_1^R}$	$a_{37} = \bar{k}_E^R n_{12}^R$	$\bar{k}_E^R$	3	3, 1.5
38	$\mathrm{D_{12}^R} \xrightarrow{\delta} \mathrm{D_1^R}$	$a_{38} = \delta n_{12}^R$	δ	3	3
39	$\mathrm{D_1^R}+\mathrm{D_2^R} \xrightarrow{k_M} \mathrm{D_{12}^R}+\mathrm{D_2^R}$	$a_{39} = \frac{k_M}{\Omega} n_1^R n_2^R$	$\frac{k_M}{\Omega}$	0.2	0.2
40	$\mathrm{D_1^R}+\mathrm{D_{12}^R} \xrightarrow{k_M} \mathrm{D_{12}^R}+\mathrm{D_{12}^R}$	$a_{40} = \frac{k_M}{\Omega} n_1^R n_{12}^R$	$\frac{k_M}{\Omega}$	0.2	0.2
41	$\mathrm{D_1^R}+\mathrm{D_1^R} \xrightarrow{\bar{k}_M} \mathrm{D_{12}^R}+\mathrm{D_1^R}$	$a_{41} = \frac{\bar{k}_M}{\Omega} \frac{n_1^R(n_1^R-1)}{2}$	$\frac{\bar{k}_M}{\Omega}$	0.2	0.2
42	$\mathrm{D_1^R}+\mathrm{D_{12}^R} \xrightarrow{\bar{k}_M} \mathrm{D_{12}^R}+\mathrm{D_{12}^R}$	$a_{42} = \frac{\bar{k}_M}{\Omega} n_1^R n_{12}^R$	$\frac{\bar{k}_M}{\Omega}$	0.2	0.2
43	$\mathrm{D_{12}^R}+\mathrm{D^A} \xrightarrow{k_E^R} \mathrm{D_1^R}+\mathrm{D^A}$	$a_{43} = \frac{k_E^R}{\Omega} n_{12}^R n^A$	$\frac{k_E^R}{\Omega}$	1	1, 0.5
44	$\mathrm{D_2^R} \xrightarrow{k_{W0}^1} \mathrm{D_{12}^R}$	$a_{44} = k_{W0}^1 n_2^R$	k_{W0}^1	3.5	3.5
45	$\mathrm{D_{12}^R} \xrightarrow{k_T^{'}} \mathrm{D_2^R}$	$a_{45} = k_T^{'} n_{12}^R$	$k_T^{'}$	3, 1.5	3
46	$\mathrm{D_{12}^R} \xrightarrow{\delta^{'}} \mathrm{D_2^R}$	$a_{46} = \delta^{'} n_{12}^R$	$\delta^{'}$	3, 1.5	3
47	$\mathrm{D_2^R}+\mathrm{D_2^R} \xrightarrow{k_M^{'}} \mathrm{D_{12}^R}+\mathrm{D_2^R}$	$a_{47} = \frac{k_M^{'}}{\Omega} \frac{n_2^R(n_2^R-1)}{2}$	$\frac{k_M^{'}}{\Omega}$	0.2	0.2
48	$\mathrm{D_2^R}+\mathrm{D_{12}^R} \xrightarrow{k_M^{'}} \mathrm{D_{12}^R}+\mathrm{D_{12}^R}$	$a_{48} = \frac{k_M^{'}}{\Omega} n_2^R n_{12}^R$	$\frac{k_M^{'}}{\Omega}$	0.2	0.2
49	$\mathrm{D_{12}^R}+\mathrm{D^A} \xrightarrow{k_T^{'*}} \mathrm{D_2^R}+\mathrm{D^A}$	$a_{49} = \frac{k_T^{'*}}{\Omega} n_{12}^R n^A$	$\frac{k_T^{'*}}{\Omega}$	1, 0.5	1
50	$\mathrm{D^A} \xrightarrow{\alpha_x} \mathrm{D^A}+\mathrm{X}$	$a_{50} = \alpha_x n^A$	α_x	0, 0.1, 10	0, 0.1, 10
51	$\mathrm{X} \xrightarrow{\gamma_x} \emptyset$	$a_{51} = \gamma_x n^X$	γ_x	1	1
52	$\mathrm{C_A} \xrightarrow{\delta} \mathrm{D}+\mathrm{X}$	$a_{52} = \delta n^A$	δ	3	3

Table I: **Reactions and parameter values used to generate the plots in Fig C.**

R_j	Reaction	Prop.Func.(a_j)	Param.	Value (h^{-1}) lower plots	Value (h^{-1}) upper plots left plots	Value (h^{-1}) upper plots right plots
1	$D + X \xrightarrow{a} C_A$	$a_1 = \frac{a}{\Omega} n^D n^X$	$\frac{a}{\Omega}$	10	10	10
2	$C_A \xrightarrow{d} D + X$	$a_2 = d n_A^C$	d	10	10	10
3	$D \xrightarrow{k_{W0}^A} D^A$	$a_3 = k_{W0}^A n^D$	k_{W0}^A	86	86	86
4	$C_A \xrightarrow{k_{W0}^A} D^A + X$	$a_4 = k_{W0}^A n^D$	k_{W0}^A	86	86	86
5	$C_A \xrightarrow{k_W^A} D^A + X$	$a_5 = k_W^A n_A^C$	k_W^A	300	300	300
6	$D^A \xrightarrow{\bar{k}_E^A} D$	$a_6 = \bar{k}_E^A n^A$	$\bar{k}_E^A$	4	4	4
7	$D^A \xrightarrow{\delta} D$	$a_7 = \delta n^A$	δ	4	4	4
8	$D + D^A \xrightarrow{k_M^A} D^A + D^A$	$a_8 = \frac{k_M^A}{\Omega} n^D n^A$	$\frac{k_M^A}{\Omega}$	1	1	1
9	$C_A + D^A \xrightarrow{k_M^A} D^A + D^A + X$	$a_9 = \frac{k_M^A}{\Omega} n_A^C n^A$	$\frac{k_M^A}{\Omega}$	1	1	1
10	$D^A + D_1^R \xrightarrow{k_E^A} D + D_1^R$	$a_{10} = \frac{k_E^A}{\Omega} n^A n_1^R$	$\frac{k_E^A}{\Omega}$	1	0.3	5
11	$D^A + D_{12}^R \xrightarrow{k_E^A} D + D_{12}^R$	$a_{11} = \frac{k_E^A}{\Omega} n^A n_{12}^R$	$\frac{k_E^A}{\Omega}$	1	0.3	5
12	$D^A + D_2^R \xrightarrow{k_E^A} D + D_2^R$	$a_{12} = \frac{k_E^A}{\Omega} n^A n_2^R$	$\frac{k_E^A}{\Omega}$	1	0.3	5
13	$D^A + D_{12}^R \xrightarrow{k_E^A} D + D_{12}^R$	$a_{13} = \frac{k_E^A}{\Omega} n^A n_{12}^R$	$\frac{k_E^A}{\Omega}$	1	0.3	5
14	$D \xrightarrow{k_{W0}^1} D_1^R$	$a_{14} = k_{W0}^1 n^D$	k_{W0}^1	5	5	5
15	$C_A \xrightarrow{k_{W0}^1} D_1^R + X$	$a_{15} = k_{W0}^1 n^D$	k_{W0}^1	5	5	5
16	$D_1^R \xrightarrow{k_T'} D$	$a_{16} = k_T' n_1^R$	k_T'	1.6, 0.8	1.6, 0.8	1.6, 0.8
17	$D_1^R \xrightarrow{\delta'} D$	$a_{17} = \delta' n_1^R$	δ'	1.6, 0.8	1.6, 0.8	1.6, 0.8
18	$D + D_2^R \xrightarrow{k_M'} D_1^R + D_2^R$	$a_{18} = \frac{k_M'}{\Omega} n^D n_2^R$	$\frac{k_M'}{\Omega}$	0.2	0.2	0.2
19	$C_A + D_2^R \xrightarrow{k_M'} D_1^R + D_2^R + X$	$a_{19} = \frac{k_M'}{\Omega} n^D n_2^R$	$\frac{k_M'}{\Omega}$	0.2	0.2	0.2
20	$D + D_{12}^R \xrightarrow{k_M'} D_1^R + D_{12}^R$	$a_{20} = \frac{k_M'}{\Omega} n^D n_{12}^R$	$\frac{k_M'}{\Omega}$	0.2	0.2	0.2
21	$C_A + D_{12}^R \xrightarrow{k_M'} D_1^R + D_{12}^R + X$	$a_{21} = \frac{k_M'}{\Omega} n^D n_{12}^R$	$\frac{k_M'}{\Omega}$	0.2	0.2	0.2
22	$D_1^R + D^A \xrightarrow{k_T'^*} D + D^A$	$a_{22} = \frac{k_T'^*}{\Omega} n_1^R n^A$	$\frac{k_T'^*}{\Omega}$	0.4, 0.2	0.12, 0.06	2, 1
23	$D \xrightarrow{k_{W0}^2} D_2^R$	$a_{23} = k_{W0}^2 n^D$	k_{W0}^2	5	5	5
24	$C_A \xrightarrow{k_{W0}^2} D_2^R + X$	$a_{24} = k_{W0}^2 n^D$	k_{W0}^2	5	5	5
25	$D_2^R \xrightarrow{\bar{k}_E^R} D$	$a_{25} = \bar{k}_E^R n_2^R$	$\bar{k}_E^R$	4	4	4
26	$D_2^R \xrightarrow{\delta} D$	$a_{26} = \delta n_2^R$	δ	4	4	4
27	$D + D_2^R \xrightarrow{k_M} D_2^R + D_2^R$	$a_{27} = \frac{k_M}{\Omega} n^D n_2^R$	$\frac{k_M}{\Omega}$	0.2	0.2	0.2
28	$C_A + D_2^R \xrightarrow{k_M} D_2^R + D_2^R + X$	$a_{28} = \frac{k_M}{\Omega} n^D n_2^R$	$\frac{k_M}{\Omega}$	0.2	0.2	0.2
29	$D + D_{12}^R \xrightarrow{k_M} D_2^R + D_{12}^R$	$a_{29} = \frac{k_M}{\Omega} n^D n_{12}^R$	$\frac{k_M}{\Omega}$	0.2	0.2	0.2
30	$C_A + D_{12}^R \xrightarrow{k_M} D_2^R + D_{12}^R + X$	$a_{30} = \frac{k_M}{\Omega} n^D n_{12}^R$	$\frac{k_M}{\Omega}$	0.2	0.2	0.2
31	$D + D_1^R \xrightarrow{\bar{k}_M} D_2^R + D_1^R$	$a_{31} = \frac{\bar{k}_M}{\Omega} n^D n_1^R$	$\frac{\bar{k}_M}{\Omega}$	0.2	0.2	0.2
32	$C_A + D_1^R \xrightarrow{\bar{k}_M} D_2^R + D_1^R + X$	$a_{32} = \frac{\bar{k}_M}{\Omega} n^D n_1^R$	$\frac{\bar{k}_M}{\Omega}$	0.2	0.2	0.2
33	$D + D_{12}^R \xrightarrow{\bar{k}_M} D_2^R + D_{12}^R$	$a_{33} = \frac{\bar{k}_M}{\Omega} n^D n_{12}^R$	$\frac{\bar{k}_M}{\Omega}$	0.2	0.2	0.2
34	$C_A + D_{12}^R \xrightarrow{\bar{k}_M} D_2^R + D_{12}^R + X$	$a_{34} = \frac{\bar{k}_M}{\Omega} n^D n_{12}^R$	$\frac{\bar{k}_M}{\Omega}$	0.2	0.2	0.2
35	$D_2^R + D^A \xrightarrow{k_E^R} D + D^A$	$a_{35} = \frac{k_E^R}{\Omega} n_2^R n^A$	$\frac{k_E^R}{\Omega}$	1	0.3	10
36	$D_1^R \xrightarrow{k_{W0}^2} D_{12}^R$	$a_{36} = k_{W0}^2 n_1^R$	k_{W0}^2	5	5	5
37	$D_{12}^R \xrightarrow{\bar{k}_E^R} D_1^R$	$a_{37} = \bar{k}_E^R n_{12}^R$	$\bar{k}_E^R$	4	4	4
38	$D_{12}^R \xrightarrow{\delta} D_1^R$	$a_{38} = \delta n_{12}^R$	δ	4	4	4
39	$D_1^R + D_2^R \xrightarrow{k_M} D_{12}^R + D_2^R$	$a_{39} = \frac{k_M}{\Omega} n_1^R n_2^R$	$\frac{k_M}{\Omega}$	0.2	0.2	0.2
40	$D_1^R + D_{12}^R \xrightarrow{k_M} D_{12}^R + D_{12}^R$	$a_{40} = \frac{k_M}{\Omega} n_1^R n_{12}^R$	$\frac{k_M}{\Omega}$	0.2	0.2	0.2
41	$D_1^R + D_1^R \xrightarrow{\bar{k}_M} D_{12}^R + D_1^R$	$a_{41} = \frac{\bar{k}_M}{\Omega} \frac{n_1^R(n_1^R-1)}{2}$	$\frac{\bar{k}_M}{\Omega}$	0.2	0.2	0.2
42	$D_1^R + D_{12}^R \xrightarrow{\bar{k}_M} D_{12}^R + D_{12}^R$	$a_{42} = \frac{\bar{k}_M}{\Omega} n_1^R n_{12}^R$	$\frac{\bar{k}_M}{\Omega}$	0.2	0.2	0.2
43	$D_{12}^R + D^A \xrightarrow{k_E^R} D_1^R + D^A$	$a_{43} = \frac{k_E^R}{\Omega} n_{12}^R n^A$	$\frac{k_E^R}{\Omega}$	1	0.3	10
44	$D_2^R \xrightarrow{k_{W0}^1} D_{12}^R$	$a_{44} = k_{W0}^1 n_2^R$	k_{W0}^1	5	5	5
45	$D_{12}^R \xrightarrow{k_T'} D_2^R$	$a_{45} = k_T' n_{12}^R$	k_T'	1.6, 0.8	1.6, 0.8	1.6, 0.8
46	$D_{12}^R \xrightarrow{\delta'} D_2^R$	$a_{46} = \delta' n_{12}^R$	δ'	1.6, 0.8	1.6, 0.8	1.6, 0.8
47	$D_2^R + D_2^R \xrightarrow{k_M'} D_{12}^R + D_2^R$	$a_{47} = \frac{k_M'}{\Omega} \frac{n_2^R(n_2^R-1)}{2}$	$\frac{k_M'}{\Omega}$	0.2	0.2	0.2
48	$D_2^R + D_{12}^R \xrightarrow{k_M'} D_{12}^R + D_{12}^R$	$a_{48} = \frac{k_M'}{\Omega} n_2^R n_{12}^R$	$\frac{k_M'}{\Omega}$	0.2	0.2	0.2
49	$D_{12}^R + D^A \xrightarrow{k_T'^*} D_2^R + D^A$	$a_{49} = \frac{k_T'^*}{\Omega} n_{12}^R n^A$	$\frac{k_T'^*}{\Omega}$	0.4, 0.2	0.12, 0.06	2, 1
50	$D^A \xrightarrow{\alpha_x} D^A + X$	$a_{50} = \alpha_x n^A$	α_x	0, 10	0, 10	0, 10
51	$X \xrightarrow{\gamma_x} \emptyset$	$a_{51} = \gamma_x n^X$	γ_x	1	1	1
52	$C_A \xrightarrow{\delta} D + X$	$a_{52} = \delta n^A$	δ	4	4	4

Table J: **Reactions and parameter values used to generate the plots in Fig E.**

**R**$_k$	**Reaction**	**Prop.Func.**(a_k)	**Param.**	**Value (h**$^{-1}$**)** Fig G left plots	**Value (h**$^{-1}$**)** Fig G central plots	**Value (h**$^{-1}$**)** Fig G right plots	**Value (h**$^{-1}$**)** Fig H left plots	**Value (h**$^{-1}$**)** Fig H right plots
1_i	$\mathrm{D^{i}} + \mathrm{i} \xrightarrow{a} \mathrm{C^{i}_{A}}$	$a_{1_i} = \frac{a}{\Omega} n^{D,i} n^{i}$	$\frac{a}{\Omega}$	10	10	10	10	10
2_i	$\mathrm{C^{i}_{A}} \xrightarrow{d} \mathrm{D^{i}} + \mathrm{i}$	$a_{2_i} = d n^{C,i}_{A}$	d	10	10	10	10	10
3_i	$\mathrm{D^{i}} + \mathrm{j} \xrightarrow{a} \mathrm{C^{i}_{R}}$	$a_{3_i} = \frac{a}{\Omega} n^{D,i} n^{j}$	$\frac{a}{\Omega}$	10	10	10	10	10
4_i	$\mathrm{C^{i}_{R}} \xrightarrow{d} \mathrm{D^{i}} + \mathrm{j}$	$a_{4_i} = d n^{C,i}_{R}$	d	10	10	10	10	10
5_i	$\mathrm{D^{i}} \xrightarrow{k^{A}_{W0}} \mathrm{D^{A,i}}$	$a_{5_i} = k^{A}_{W0} n^{D,i}$	k^{A}_{W0}	3.5	3.5	3.5	3.5	3.5
6_i	$\mathrm{C^{i}_{A}} \xrightarrow{k^{A}_{W0}} \mathrm{D^{A,i}} + \mathrm{i}$	$a_{6_i} = k^{A}_{W0} n^{C,i}_{A}$	k^{A}_{W0}	3.5	3.5	3.5	3.5	3.5
7_i	$\mathrm{C^{i}_{R}} \xrightarrow{k^{A}_{W0}} \mathrm{D^{A,i}} + \mathrm{j}$	$a_{7_i} = k^{A}_{W0} n^{C,i}_{R}$	k^{A}_{W0}	3.5	3.5	3.5	3.5	3.5
8_i	$\mathrm{C^{i}_{A}} \xrightarrow{k^{A}_{W}} \mathrm{D^{A,i}} + \mathrm{i}$	$a_{8_i} = k^{A}_{W} n^{C,i}_{A}$	k^{A}_{W}	300	300	300	300	300
9_i	$\mathrm{D^{A,i}} \xrightarrow{\bar{k}^{A}_{E}} \mathrm{D^{i}}$	$a_{9_i} = \bar{k}^{A}_{E} n^{A,i}$	$\bar{k}^{A}_{E}$	12, 5	12, 5	12, 5	12, 5	12, 5
10_i	$\mathrm{D^{A,i}} \xrightarrow{\delta} \mathrm{D^{i}}$	$a_{10_i} = \delta n^{A,i}$	δ	12, 5	12, 5	12, 5	12, 5	12, 5
11_i	$\mathrm{D^{i}} + \mathrm{D^{A,i}} \xrightarrow{k^{A}_{M}} \mathrm{D^{A,i}} + \mathrm{D^{A,i}}$	$a_{11_i} = \frac{k^{A}_{M}}{\Omega} n^{D,i} n^{A,i}$	$\frac{k^{A}_{M}}{\Omega}$	1	1	1	1	1
12_i	$\mathrm{C^{i}_{A}} + \mathrm{D^{A,i}} \xrightarrow{k^{A}_{M}} \mathrm{D^{A,i}} + \mathrm{D^{A,i}} + \mathrm{i}$	$a_{12_i} = \frac{k^{A}_{M}}{\Omega} n^{C,i}_{A} n^{A,i}$	$\frac{k^{A}_{M}}{\Omega}$	1	1	1	1	1
13_i	$\mathrm{C^{i}_{R}} + \mathrm{D^{A,i}} \xrightarrow{k^{A}_{M}} \mathrm{D^{A,i}} + \mathrm{D^{A,i}} + \mathrm{j}$	$a_{13_i} = \frac{k^{A}_{M}}{\Omega} n^{C,i}_{R} n^{A,i}$	$\frac{k^{A}_{M}}{\Omega}$	1	1	1	1	1
14_i	$\mathrm{D^{A,i}} + \mathrm{D^{R,i}_{1}} \xrightarrow{k^{A}_{E}} \mathrm{D^{i}} + \mathrm{D^{R,i}_{1}}$	$a_{14_i} = \frac{k^{A}_{E}}{\Omega} n^{A,i} n^{R,i}_{1}$	$\frac{k^{A}_{E}}{\Omega}$	0.2	1	10	1	1
15_i	$\mathrm{D^{A,i}} + \mathrm{D^{R,i}_{12}} \xrightarrow{k^{A}_{E}} \mathrm{D^{i}} + \mathrm{D^{R,i}_{12}}$	$a_{15_i} = \frac{k^{A}_{E}}{\Omega} n^{A,i} n^{R,i}_{12}$	$\frac{k^{A}_{E}}{\Omega}$	0.2	1	10	1	1
16_i	$\mathrm{D^{A,i}} + \mathrm{D^{R,i}_{2}} \xrightarrow{k^{A}_{E}} \mathrm{D^{i}} + \mathrm{D^{R,i}_{2}}$	$a_{16_i} = \frac{k^{A}_{E}}{\Omega} n^{A,i} n^{R,i}_{2}$	$\frac{k^{A}_{E}}{\Omega}$	0.2	1	10	1	1
17_i	$\mathrm{D^{A,i}} + \mathrm{D^{R,i}_{12}} \xrightarrow{k^{A}_{E}} \mathrm{D^{i}} + \mathrm{D^{R,i}_{12}}$	$a_{17_i} = \frac{k^{A}_{E}}{\Omega} n^{A,i} n^{R,i}_{12}$	$\frac{k^{A}_{E}}{\Omega}$	0.2	1	10	1	1
18_i	$\mathrm{D^{i}} \xrightarrow{k^{1}_{W0}} \mathrm{D^{R,i}_{1}}$	$a_{18_i} = k^{1}_{W0} n^{D,i}$	k^{1}_{W0}	3.5	3.5	3.5	3.5	3.5
19_i	$\mathrm{C^{i}_{A}} \xrightarrow{k^{1}_{W0}} \mathrm{D^{R,i}_{1}} + \mathrm{i}$	$a_{19_i} = k^{1}_{W0} n^{C,i}_{A}$	k^{1}_{W0}	3.5	3.5	3.5	3.5	3.5
20_i	$\mathrm{C^{i}_{R}} \xrightarrow{k^{1}_{W0}} \mathrm{D^{R,i}_{1}} + \mathrm{j}$	$a_{20_i} = k^{1}_{W0} n^{C,i}_{R}$	k^{1}_{W0}	3.5	3.5	3.5	3.5	3.5
21_i	$\mathrm{C^{i}_{R}} \xrightarrow{k^{1}_{W}} \mathrm{D^{R,i}_{1}} + \mathrm{j}$	$a_{21_i} = k^{1}_{W} n^{C,i}_{R}$	k^{1}_{W}	300	300	300	300	300
22_i	$\mathrm{D^{R,i}_{1}} \xrightarrow{k'_{T}} \mathrm{D^{i}}$	$a_{22_i} = k'_{T} n^{R,i}_{1}$	k'_{T}	7.2, 3	7.2, 3	7.2, 3	3.6, 1.5	1.2, 0.5
23_i	$\mathrm{D^{R,i}_{1}} \xrightarrow{\delta'} \mathrm{D^{i}}$	$a_{23_i} = \delta' n^{R,i}_{1}$	δ'	7.2, 3	7.2, 3	7.2, 3	3.6, 1.5	1.2, 0.5
24_i	$\mathrm{D^{i}} + \mathrm{D^{R,i}_{2}} \xrightarrow{k'_{M}} \mathrm{D^{R,i}_{1}} + \mathrm{D^{R,i}_{2}}$	$a_{24_i} = \frac{k'_{M}}{\Omega} n^{D,i} n^{R,i}_{2}$	$\frac{k'_{M}}{\Omega}$	0.2	0.2	0.2	0.2	0.2
25_i	$\mathrm{C^{i}_{R}} + \mathrm{D^{R,i}_{2}} \xrightarrow{k'_{M}} \mathrm{D^{R,i}_{1}} + \mathrm{D^{R,i}_{2}} + \mathrm{j}$	$a_{25_i} = \frac{k'_{M}}{\Omega} n^{C,i}_{R} n^{R,i}_{2}$	$\frac{k'_{M}}{\Omega}$	0.2	0.2	0.2	0.2	0.2
26_i	$\mathrm{C^{i}_{A}} + \mathrm{D^{R,i}_{2}} \xrightarrow{k'_{M}} \mathrm{D^{R,i}_{1}} + \mathrm{D^{R,i}_{2}} + \mathrm{i}$	$a_{26_i} = \frac{k'_{M}}{\Omega} n^{C,i}_{A} n^{R,i}_{2}$	$\frac{k'_{M}}{\Omega}$	0.2	0.2	0.2	0.2	0.2
27_i	$\mathrm{D^{i}} + \mathrm{D^{R,i}_{12}} \xrightarrow{k'_{M}} \mathrm{D^{R,i}_{1}} + \mathrm{D^{R,i}_{12}}$	$a_{27_i} = \frac{k'_{M}}{\Omega} n^{D,i} n^{R,i}_{12}$	$\frac{k'_{M}}{\Omega}$	0.2	0.2	0.2	0.2	0.2
28_i	$\mathrm{C^{i}_{R}} + \mathrm{D^{R,i}_{12}} \xrightarrow{k'_{M}} \mathrm{D^{R,i}_{1}} + \mathrm{D^{R,i}_{12}} + \mathrm{j}$	$a_{28_i} = \frac{k'_{M}}{\Omega} n^{C,i}_{R} n^{R,i}_{12}$	$\frac{k'_{M}}{\Omega}$	0.2	0.2	0.2	0.2	0.2
29_i	$\mathrm{C^{i}_{A}} + \mathrm{D^{R,i}_{12}} \xrightarrow{k'_{M}} \mathrm{D^{R,i}_{1}} + \mathrm{D^{R,i}_{12}} + \mathrm{i}$	$a_{29_i} = \frac{k'_{M}}{\Omega} n^{C,i}_{A} n^{R,i}_{12}$	$\frac{k'_{M}}{\Omega}$	0.2	0.2	0.2	0.2	0.2
30_i	$\mathrm{D^{R,i}_{1}} + \mathrm{D^{A,i}} \xrightarrow{k'^{*}_{T}} \mathrm{D^{i}} + \mathrm{D^{A,i}}$	$a_{30_i} = \frac{k'^{*}_{T}}{\Omega} n^{R,i}_{1} n^{A,i}$	$\frac{k'^{*}_{T}}{\Omega}$	0.12	0.6	6	0.3	0.1
31_i	$\mathrm{D^{i}} \xrightarrow{k^{2}_{W0}} \mathrm{D^{R,i}_{2}}$	$a_{31_i} = k^{2}_{W0} n^{D,i}$	k^{2}_{W0}	3.5	3.5	3.5	3.5	3.5
32_i	$\mathrm{C^{i}_{A}} \xrightarrow{k^{2}_{W0}} \mathrm{D^{R,i}_{2}} + \mathrm{i}$	$a_{32_i} = k^{2}_{W0} n^{C,i}_{A}$	k^{2}_{W0}	3.5	3.5	3.5	3.5	3.5
33_i	$\mathrm{C^{i}_{R}} \xrightarrow{k^{2}_{W0}} \mathrm{D^{R,i}_{2}} + \mathrm{j}$	$a_{33_i} = k^{2}_{W0} n^{C,i}_{R}$	k^{2}_{W0}	3.5	3.5	3.5	3.5	3.5
34_i	$\mathrm{C^{i}_{R}} \xrightarrow{k^{2}_{W}} \mathrm{D^{R,i}_{2}} + \mathrm{j}$	$a_{34_i} = k^{2}_{W} n^{C,i}_{R}$	k^{2}_{W}	300	300	300	300	300
35_i	$\mathrm{D^{R,i}_{2}} \xrightarrow{\bar{k}^{R}_{E}} \mathrm{D^{i}}$	$a_{35_i} = \bar{k}^{R}_{E} n^{R,i}_{2}$	$\bar{k}^{R}_{E}$	12, 5	12, 5	12, 5	12, 5	12, 5
36_i	$\mathrm{D^{R,i}_{2}} \xrightarrow{\delta} \mathrm{D^{i}}$	$a_{36_i} = \delta n^{R,i}_{2}$	δ	12, 5	12, 5	12, 5	12, 5	12, 5
37_i	$\mathrm{D^{i}} + \mathrm{D^{R,i}_{2}} \xrightarrow{k_{M}} \mathrm{D^{R,i}_{2}} + \mathrm{D^{R,i}_{2}}$	$a_{37_i} = \frac{k_{M}}{\Omega} n^{D,i} n^{R,i}_{2}$	$\frac{k_{M}}{\Omega}$	0.2	0.2	0.2	0.2	0.2
38_i	$\mathrm{C^{i}_{R}} + \mathrm{D^{R,i}_{2}} \xrightarrow{k_{M}} \mathrm{D^{R,i}_{2}} + \mathrm{D^{R,i}_{2}} + \mathrm{j}$	$a_{38_i} = \frac{k_{M}}{\Omega} n^{C,i}_{R} n^{R,i}_{2}$	$\frac{k_{M}}{\Omega}$	0.2	0.2	0.2	0.2	0.2
39_i	$\mathrm{C^{i}_{A}} + \mathrm{D^{R,i}_{2}} \xrightarrow{k_{M}} \mathrm{D^{R,i}_{2}} + \mathrm{D^{R,i}_{2}} + \mathrm{i}$	$a_{39_i} = \frac{k_{M}}{\Omega} n^{C,i}_{A} n^{R,i}_{2}$	$\frac{k_{M}}{\Omega}$	0.2	0.2	0.2	0.2	0.2

Table K: **Reactions and parameter values used to generate the plots in Figs G, H, with** $i, j = X, Z$ **and** $i \neq j$**.**

$\mathbf{R}_k$	**Reaction**	**Prop.Func.**(a_k)	**Param.**	**Value** $(\mathbf{h}^{-1})$ Fig G left plots	**Value** $(\mathbf{h}^{-1})$ Fig G central plots	**Value** $(\mathbf{h}^{-1})$ Fig G right plots	**Value** $(\mathbf{h}^{-1})$ Fig H left plots	**Value** $(\mathbf{h}^{-1})$ Fig H right plots
40_i	$\mathrm{D^i} + \mathrm{D^{R,i}_{12}} \xrightarrow{k_M} \mathrm{D^{R,i}_2} + \mathrm{D^{R,i}_{12}}$	$a_{40_i} = \frac{k_M}{\Omega} n^{D,i} n^{R,i}_{12}$	$\frac{k_M}{\Omega}$	0.2	0.2	0.2	0.2	0.2
41_i	$\mathrm{C^i_R} + \mathrm{D^{R,i}_{12}} \xrightarrow{k_M} \mathrm{D^{R,i}_2} + \mathrm{D^{R,i}_{12}} + \mathrm{j}$	$a_{41_i} = \frac{k_M}{\Omega} n^{C,i}_R n^{R,i}_{12}$	$\frac{k_M}{\Omega}$	0.2	0.2	0.2	0.2	0.2
42_i	$\mathrm{C^i_A} + \mathrm{D^{R,i}_{12}} \xrightarrow{k_M} \mathrm{D^{R,i}_2} + \mathrm{D^{R,i}_{12}} + \mathrm{i}$	$a_{42_i} = \frac{k_M}{\Omega} n^{C,i}_A n^{R,i}_{12}$	$\frac{k_M}{\Omega}$	0.2	0.2	0.2	0.2	0.2
43_i	$\mathrm{D^i} + \mathrm{D^{R,i}_1} \xrightarrow{\bar{k}_M} \mathrm{D^{R,i}_2} + \mathrm{D^{R,i}_1}$	$a_{43_i} = \frac{\bar{k}_M}{\Omega} n^{D,i} n^{R,i}_1$	$\frac{\bar{k}_M}{\Omega}$	0.2	0.2	0.2	0.2	0.2
44_i	$\mathrm{C^i_R} + \mathrm{D^{R,i}_1} \xrightarrow{\bar{k}_M} \mathrm{D^{R,i}_2} + \mathrm{D^{R,i}_1} + \mathrm{j}$	$a_{44_i} = \frac{\bar{k}_M}{\Omega} n^{C,i}_R n^{R,i}_1$	$\frac{\bar{k}_M}{\Omega}$	0.2	0.2	0.2	0.2	0.2
45_i	$\mathrm{C^i_A} + \mathrm{D^{R,i}_1} \xrightarrow{\bar{k}_M} \mathrm{D^{R,i}_2} + \mathrm{D^{R,i}_1} + \mathrm{i}$	$a_{45_i} = \frac{\bar{k}_M}{\Omega} n^{C,i}_A n^{R,i}_1$	$\frac{\bar{k}_M}{\Omega}$	0.2	0.2	0.2	0.2	0.2
46_i	$\mathrm{D^i} + \mathrm{D^{R,i}_{12}} \xrightarrow{\bar{k}_M} \mathrm{D^{R,i}_2} + \mathrm{D^{R,i}_{12}}$	$a_{46_i} = \frac{\bar{k}_M}{\Omega} n^{D,i} n^{R,i}_{12}$	$\frac{\bar{k}_M}{\Omega}$	0.2	0.2	0.2	0.2	0.2
47_i	$\mathrm{C^i_R} + \mathrm{D^{R,i}_{12}} \xrightarrow{\bar{k}_M} \mathrm{D^{R,i}_2} + \mathrm{D^{R,i}_{12}} + \mathrm{j}$	$a_{47_i} = \frac{\bar{k}_M}{\Omega} n^{C,i}_R n^{R,i}_{12}$	$\frac{\bar{k}_M}{\Omega}$	0.2	0.2	0.2	0.2	0.2
48_i	$\mathrm{C^i_A} + \mathrm{D^{R,i}_{12}} \xrightarrow{\bar{k}_M} \mathrm{D^{R,i}_2} + \mathrm{D^{R,i}_{12}} + \mathrm{i}$	$a_{48_i} = \frac{\bar{k}_M}{\Omega} n^{C,i}_A n^{R,i}_{12}$	$\frac{\bar{k}_M}{\Omega}$	0.2	0.2	0.2	0.2	0.2
49_i	$\mathrm{D^{R,i}_2} + \mathrm{D^{A,i}} \xrightarrow{k^R_E} \mathrm{D^i} + \mathrm{D^{A,i}}$	$a_{49_i} = \frac{k^R_E}{\Omega} n^{R,i}_2 n^{A,i}$	$\frac{k^R_E}{\Omega}$	0.2	1	10	1	1
50_i	$\mathrm{D^{R,i}_1} \xrightarrow{k^2_{W0}} \mathrm{D^{R,i}_{12}}$	$a_{50_i} = k^2_{W0} n^{R,i}_1$	k^2_{W0}	3.5	3.5	3.5	3.5	3.5
51_i	$\mathrm{D^{R,i}_{12}} \xrightarrow{\bar{k}^R_E} \mathrm{D^{R,i}_1}$	$a_{51_i} = \bar{k}^R_E n^{R,i}_{12}$	$\bar{k}^R_E$	12, 5	12, 5	12, 5	12, 5	12, 5
52_i	$\mathrm{D^{R,i}_{12}} \xrightarrow{\delta} \mathrm{D^{R,i}_1}$	$a_{52_i} = \delta n^{R,i}_{12}$	δ	12, 5	12, 5	12, 5	12, 5	12, 5
53_i	$\mathrm{D^{R,i}_1} + \mathrm{D^{R,i}_2} \xrightarrow{k_M} \mathrm{D^{R,i}_{12}} + \mathrm{D^{R,i}_2}$	$a_{53_i} = \frac{k_M}{\Omega} n^{R,i}_1 n^{R,i}_2$	$\frac{k_M}{\Omega}$	0.2	0.2	0.2	0.2	0.2
54_i	$\mathrm{D^{R,i}_1} + \mathrm{D^{R,i}_{12}} \xrightarrow{k_M} \mathrm{D^{R,i}_{12}} + \mathrm{D^{R,i}_{12}}$	$a_{54_i} = \frac{k_M}{\Omega} n^{R,i}_1 n^{R,i}_{12}$	$\frac{k_M}{\Omega}$	0.2	0.2	0.2	0.2	0.2
55_i	$\mathrm{D^{R,i}_1} + \mathrm{D^{R,i}_1} \xrightarrow{\bar{k}_M} \mathrm{D^{R,i}_{12}} + \mathrm{D^{R,i}_1}$	$a_{55_i} = \frac{\bar{k}_M}{\Omega} \frac{n^{R,i}_1 (n^{R,i}_1 - 1)}{2}$	$\frac{\bar{k}_M}{\Omega}$	0.2	0.2	0.2	0.2	0.2
56_i	$\mathrm{D^{R,i}_1} + \mathrm{D^{R,i}_{12}} \xrightarrow{\bar{k}_M} \mathrm{D^{R,i}_{12}} + \mathrm{D^{R,i}_{12}}$	$a_{56_i} = \frac{\bar{k}_M}{\Omega} n^{R,i}_1 n^{R,i}_{12}$	$\frac{\bar{k}_M}{\Omega}$	0.2	0.2	0.2	0.2	0.2
57_i	$\mathrm{D^{R,i}_{12}} + \mathrm{D^{A,i}} \xrightarrow{k^R_E} \mathrm{D^{R,i}_1} + \mathrm{D^{A,i}}$	$a_{57_i} = \frac{k^R_E}{\Omega} n^{R,i}_{12} n^{A,i}$	$\frac{k^R_E}{\Omega}$	0.2	1	10	1	1
58_i	$\mathrm{D^{R,i}_2} \xrightarrow{k^1_{W0}} \mathrm{D^{R,i}_{12}}$	$a_{58_i} = k^1_{W0} n^{R,i}_2$	k^1_{W0}	3.5	3.5	3.5	3.5	3.5
59_i	$\mathrm{D^{R,i}_{12}} \xrightarrow{k'_T} \mathrm{D^{R,i}_2}$	$a_{59_i} = k'_T n^{R,i}_{12}$	k'_T	7.2, 3	7.2, 3	7.2, 3	3.6, 1.5	1.2, 0.5
60_i	$\mathrm{D^{R,i}_{12}} \xrightarrow{\delta'} \mathrm{D^{R,i}_2}$	$a_{60_i} = \delta' n^{R,i}_{12}$	δ'	7.2, 3	7.2, 3	7.2, 3	3.6, 1.5	1.2, 0.5
61_i	$\mathrm{D^{R,i}_2} + \mathrm{D^{R,i}_2} \xrightarrow{k'_M} \mathrm{D^{R,i}_{12}} + \mathrm{D^{R,i}_2}$	$a_{61_i} = \frac{k'_M}{\Omega} \frac{n^{R,i}_2 (n^{R,i}_2 - 1)}{2}$	$\frac{k'_M}{\Omega}$	0.2	0.2	0.2	0.2	0.2
62_i	$\mathrm{D^{R,i}_2} + \mathrm{D^{R,i}_{12}} \xrightarrow{k'_M} \mathrm{D^{R,i}_{12}} + \mathrm{D^{R,i}_{12}}$	$a_{62_i} = \frac{k'_M}{\Omega} n^{R,i}_2 n^{R,i}_{12}$	$\frac{k'_M}{\Omega}$	0.2	0.2	0.2	0.2	0.2
63_i	$\mathrm{D^{R,i}_{12}} + \mathrm{D^{A,i}} \xrightarrow{k'^*_T} \mathrm{D^{R,i}_2} + \mathrm{D^{A,i}}$	$a_{63_i} = \frac{k'^*_T}{\Omega} n^R_{12} n^{A,i}$	$\frac{k'^*_T}{\Omega}$	0.12	0.6	6	0.3	0.1
64_i	$\mathrm{D^{A,i}} \xrightarrow{\alpha_i} \mathrm{D^{A,i}} + \mathrm{i}$	$a_{64_i} = \alpha_i n^{A,i}$	α_i	0, 10	0, 10	0, 10	0, 0.1, 10	0, 0.1, 10
65_i	$\mathrm{i} \xrightarrow{\gamma_i} \emptyset$	$a_{65_i} = \gamma_i n^i$	γ_i	1	1	1	1	1
66_i	$\mathrm{C^i_A} \xrightarrow{\delta} \mathrm{D^i} + \mathrm{i}$	$a_{66_i} = \delta n^{C,i}_A$	δ	12, 5	12, 5	12, 5	12, 5	12, 5
67_i	$\mathrm{C^i_R} \xrightarrow{\delta} \mathrm{D^i} + \mathrm{j}$	$a_{67_i} = \delta n^{C,i}_R$	δ	12, 5	12, 5	12, 5	12, 5	12, 5

Table L: **Reactions and parameter values used to generate the plots in Figs G, H, with $i, j = X, Z$ and $i \neq j$.**

References

[1] J. R. Norris. *Markov Chains*. Cambridge University Press, 1997.

[2] G. Felsenfeld and M. Groudine. “Controlling the double helix”. In: *Nature* 421 (2003).

[3] Cooper GM. *The Cell: A Molecular Approach*. Sunderland (MA): Sinauer Associates, 2000.
